# Reaction of Blatter and Verdazyl Radicals with Arynes: Synthesis and Investigation of N‐Chiral, Antiaromatic Triazines, and Tetrazinones

**DOI:** 10.1002/anie.202520021

**Published:** 2025-11-10

**Authors:** Lena Lezius, Elena S. Horst, Maximilian Scherübl, Jessika Lammert, Constantin G. Daniliuc, Tatsuya Mori, Shigehiro Yamaguchi, Armido Studer

**Affiliations:** ^1^ Organisch‐Chemisches Institut Universität Münster 48149 Münster Germany; ^2^ Department of Chemistry Graduate School of Science and Integrated Research Consortium on Chemical Sciences (IRCCS) Nagoya University Furo Chikusa Nagoya 464–8602 Japan; ^3^ Institute of Transformative Bio‐Molecules (WPI‐ITbM) Nagoya University Furo Chikusa Nagoya 464–8601 Japan

**Keywords:** Annulation, Antiaromaticity, Arynes, Chirality, Radicals

## Abstract

Arynes are versatile building blocks in organic chemistry. In contrast to their extensively examined ionic reactivity, radical reactions with arynes remain scarcely explored. In this study, we present a novel radical annulation reaction of stable Blatter and verdazyl radicals with arynes, which offers access to polycyclic triazines and tetrazinones. The N2‐ and N4‐annulated triazines derived from Blatter radicals exhibit distinct bending modes of the 8π‐electron triazine ring, yet retain a certain degree of antiaromaticity despite their nonplanar geometries, as revealed by NICS and ACID calculations. Notably, the N2‐annulated triazines feature a strongly pyramidalized nitrogen atom with N‐chirality. Optical resolution yielded pure enantiomers, from which the configurational stability was assessed to determine the N‐inversion barrier. Furthermore, one of the N2‐annulated triazine derivatives displayed intense fluorescence, highlighting its potential as a scaffold for light‐emitting materials, whereas H‐cell cycling studies of the N4‐annulated triazine derivatives demonstrated long‐term stability of the redox process underscoring their utility for battery applications.

## Graphical abstract

In this study a novel radical annulation reaction mode of Blatter and verdazyl radicals with arynes is demonstrated which offers access to antiaromatic and N‐chiral triazines and tetrazinones. The photophysical and electrochemical properties are investigated revealing their potential for application in materials science.

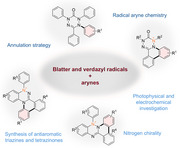

## Introduction

**Figure 1:**
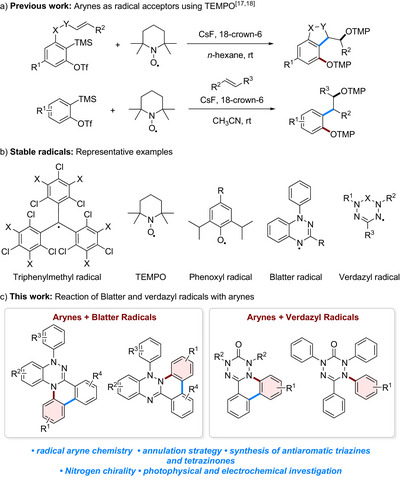
a) Methods for the use of arynes as radical acceptors, b) representative examples of stable radicals, and c) the herein reported reaction of Blatter and verdazyl radicals with arynes.

Arynes are versatile intermediates characterized by a strained triple bond embedded within the aromatic ring. They are highly reactive due to their low‐lying lowest unoccupied molecular orbital (LUMO), which results from the strained nature of the triple bond. As a consequence, they are electrophilic in nature and can therefore be used in different ionic‐type reactions like nucleophilic additions, insertion, pericyclic, metal‐catalyzed and multicomponent reactions.^[^ ^1^, ^2^, ^3^, ^4^, ^5^, ^6^ ^]^ In contrast, radical reactions with arynes have remained underdeveloped to date, as both species are highly reactive intermediates that exist only in low concentrations, making their coupling particularly challenging. Till now, only a few transformations are known in which radical intermediates in reactions with arynes were proposed.^[^ ^7^, ^8^, ^9^, ^10^, ^11^, ^12^, ^13^, ^14^, ^15^, ^16^ ^]^ In our group we overcame this concentration problem by using a stable radical as the reaction partner of the aryne, in particular TEMPO (2,2,6,6‐tetramethylpiperidine 1‐oxyl) (Figure 1a). Along these lines, we could realize a direct TEMPO‐trapping, a cyclization reaction and a three‐component reaction with activated alkenes where the aryne acts as a radical acceptor.^[^ ^17^, ^18^ ^]^ Motivated by these achievements, we envisioned to expand this kind of radical aryne chemistry to other types of stable heteroatom‐centered radicals.

Stable radicals have gained increased interest in recent years as they possess unique physicochemical properties which are interesting in terms of applications.^[^ ^19^, ^20^, ^21^, ^22^ ^]^ Since the report of the first persistent radical in 1900, the triphenylmethyl radical,^[^ ^23^ ^]^ various classes of stable radicals, centered on a range of elements, have been developed. A few representative examples of stable radicals are depicted in Figure 1b. We were particularly interested in transposing our radical aryne chemistry from O‐centered radicals to N‐centered stable radicals.^[^ ^24^ ^]^ Two of the most prominent representatives of N‐centered stable radicals are Blatter and verdazyl radicals whose characteristics like their magnetic and electronic properties have been studied.^[^ ^25^, ^26^, ^27^, ^28^, ^29^, ^30^, ^31^ ^]^ Inspired by their interesting properties we aimed to examine the reaction of Blatter and verdazyl radicals with arynes to access π‐conjugated systems in a novel annulation cascade (Figure 1c).

## Results and Discussion

**Scheme 1:**
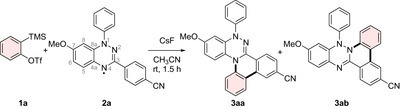
The first trial of the annulation reaction for the synthesis of **3aa** and **3ab** using aryne precursor **1a** (50 µmol, 1.0 equiv), stable radical **2a** (0.10 mmol, 2.0 equiv) and CsF (0.15 mmol, 3.0 equiv) in CH_3_CN (0.5 mL) at room temperature under argon atmosphere for 1.5 h. Under these reaction conditions **3aa** and **3ab** were obtained in 40% and 21% yield, respectively.

We started our investigations by using the aryne precursor **1a** (1.0 equiv), the Blatter radical **2a** (2.0 equiv) and CsF (3.0 equiv) in acetonitrile (CH_3_CN, 0.1 m) at room temperature for 1.5 h (Scheme 1). Under these conditions we observed the formation of two different triazine products **3aa** (40%) and **3ab** (21%) through reaction either at N4 or N2 of the Blatter radical with the aryne (for atom labels, see Scheme 1), which represents to our knowledge a new type of annulation cascade.

As the synthesized isomers should have interesting properties due to 8π‐electron antiaromatic character of the triazine rings as well as the N‐chirality (vide infra),^[^ ^32^, ^33^, ^34^ ^]^ the reaction toward **3aa** and **3ab** was further investigated. The details of the optimization can be found in Table S1 of the Supporting Information. It is noteworthy that in most test reactions a higher yield of **3aa** in comparison to **3ab** was obtained, which can be explained with the slightly higher spin density at the N4 atom of the Blatter radical calculated with DFT (for details see Supporting Information). Following optimization of solvent, reaction temperature, concentration, reactant ratios, and additives, products **3aa** and **3ab** were isolated in yields of 66% and 25%, respectively, (2.0 equiv of **1a**, 1.0 equiv of **2a**, and 6.0 equiv of CsF in CH_3_CN (0.5 mL) at room temperature under argon atmosphere for 1.5 h).

**Scheme 2:**
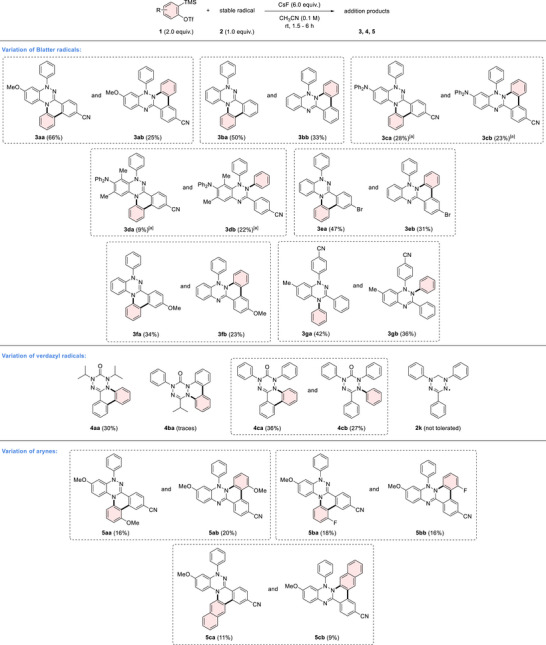
Reaction scope of the annulation reaction of Blatter and verdazyl radicals with arynes. Variation of the Blatter radical (top), the verdazyl radical (middle), and the aryne precursor (bottom). Reaction conditions: All reactions were performed with the aryne precursor **1** (0.10 mmol, 2.0 equiv), stable radical **2** (50 µmol, 1.0 equiv), and CsF (0.30 mmol, 6.0 equiv) in CH_3_CN (0.5 mL) at room temperature under argon atmosphere for 1.5 h to 6 h. Isolated yields are given. ^[a]^Reaction performed at 50 °C.

With the optimized conditions in hand, we then investigated the substrate scope of the annulation reaction by varying the stable radical and also the aryne precursor (Scheme 2). As in the case of **2a**, an unsubstituted Blatter radical **2b** worked well in the reaction with in situ generated benzyne to afford **3ba** and **3bb** in 83% total yield as a 1.5:1 mixture of separable regioisomers. A Blatter radical possessing a *N,N*‐diphenylaminyl substituent at C7 was employed in the reaction with benzyne to produce the benzannulated triazines **3ca** (28%) and **3cb** (23%). As expected, increased steric hindrance at the C6 and C8 positions of the Blatter radical significantly lowered reaction efficiency. The isomer resulting from reaction at the N4 position, **3da**, was obtained in only 9% yield, whereas no radical cyclization occurred following reaction at N2. Instead, the non‐fused N‐phenylation product **3db** was isolated in a yield of 22%. Comparison of different electronic substituents at the C3 position of the 3‐aryl ring revealed that yields were slightly higher when an electron‐withdrawing group was present (R═Br, **3ea** (47%) and **3eb** (31%); R═OMe, **3fa** (34%) and **3fb** (23%)). Further, we substituted the N1‐phenyl group in the parent Blatter radical by a *para*‐cyanophenyl group and found that the radical annulation did not occur. For both regioisomers ring fusion could not be achieved and the N‐phenylation products **3ga** (42%) and **3gb** (36%) were obtained exclusively.

Encouraged by several successful annulations of in situ generated benzyne with Blatter radicals, we decided to extend the studies toward 6‐oxoverdazyl radicals as the persistent N‐radical reaction component. Pleasingly, an oxoverdazyl radical possessing isopropyl groups as N‐substituents and a phenyl group at the C3 carbon atom afforded the tetrazinone **4aa** in 30% yield. However, the corresponding isomer with two phenyl groups at the N‐atoms along with an isopropyl group as C‐substituent provided only traces of the targeted **4ba** and its isolation was not possible. Thus, it seems that the radical annulation is favored through the 3‐aryl substituent in such oxoverdazyls. To further support that finding, we reacted the 1,3,5‐triphenylated 6‐oxoverdazyl radical with in situ generated benzyne and indeed observed formation of the annulation product **4ca** (36%) along with the simple N‐phenylation product **4cb** (27%). Unfortunately, the Kuhn verdazyl bearing a methylene group at the C6 position (see **2k**) was incompatible with the reaction conditions, likely due to side reactions involving the methylene group.^[^ ^35^ ^]^

Last, different aryne precursors were tested in the reaction with the Blatter radical **2a** under the optimized conditions. Both electron‐donating and electron‐withdrawing groups in the *ortho*‐position of the aryne were well tolerated, resulting in comparable overall product yields (R═OMe, **5aa** (16%) and **5ab** (20%); R═F, **5ba** (18%) and **5bb** (16%)). Interestingly, as in the ionic reaction with unsymmetrical arynes possessing *ortho*‐fluoro and *ortho*‐methoxy groups, only one regioisomer was formed also in the radical mode, indicating that the Garg/Houk distortion model also applies in the radical process.^[^ ^36^, ^37^, ^38^ ^]^ Moreover, with the 2‐naphthyne the regioisomeric products were formed in diminished yields (see **5ca** and **5cb**).

**Scheme 3:**
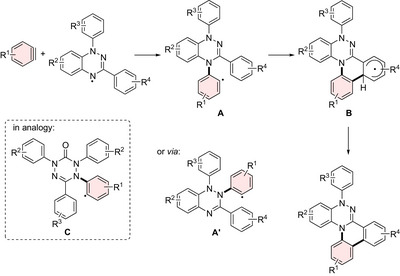
Proposed mechanism of the reaction of Blatter and verdazyl radicals with arynes.

The suggested underlying mechanism is depicted in Scheme 3. The aryne is first generated by fluoride‐induced removal of the TMS group and subsequent triflate β‐elimination.^[^ ^39^ ^]^ Then, the Blatter radical can add to the aryne to generate an aryl radical **A**. As the spin densities at N2 and N4 of Blatter radicals are generally similar,^[^ ^40^ ^]^ reaction at either nitrogen atom is feasible, leading to the formation of the aryl radicals **A** or **A’**, and eventually to distinct product isomers. The aryl radical **A** can then engage in a radical cyclization with the adjacent aryl ring to form a cyclohexadienyl radical **B**, which is finally oxidized to the isolated product. The analogous reaction with the aryl radical **A’** leads to the other isolated regioisomer. The Blatter radical, residual oxygen or the aryne can all likely act as the hydrogen atom abstractor in the final oxidative rearomatization step. Indeed, a lower yield was observed when the reaction mixture was degassed, suggesting that residual oxygen may play a role in the final rearomatization. Reactions with the verdazyl radicals should proceed in analogy. Thus, after addition of a verdazyl radical to the aryne, an aryl radical **C** is generated, which can undergo radical cyclization by reacting with the aryl rings of the tetrazinone, either at the nitrogen or carbon position. Oxidative rearomatization through hydrogen abstraction eventually leads to the isolated products. As for the other cascade, the persistent verdazyl radical, residual oxygen or the aryne can all act as the hydrogen atom abstractor in the final oxidative rearomatization step.

**Figure 2:**
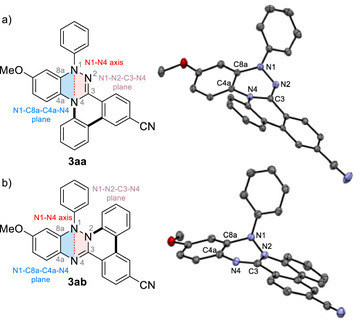
Depiction of N1‐N4 axis and mean N1‐C8a‐C4a‐N4 and N1‐N2‐C3‐N4 planes and *ORTEP* diagrams of a) **3aa** and b) **3ab** with the thermal ellipsoids at 50% probability. Hydrogen atoms are omitted for clarity. Single crystals were obtained from a racemic mixture. In **3aa**, selected bond lengths are N1–N2 1.418(2) Å, N2–C3 1.290(2) Å, and C3–N4 1.401(1) Å, with the sum of the bond angles at N1 of 353.1°. In **3ab**, selected bond lengths are N1–N2 1.418(2) Å, N2–C3 1.387(2) Å, and C3–N4 1.305(2) Å, with the sum of the bond angles at N1 of 337.2°.

Both types of Blatter annulation products possess formally 8π‐electron triazine cores, whose antiaromatic character can influence their overall structures. In general, 8π‐electron frameworks tend to adopt distorted or nonplanar geometries to mitigate the instability associated with antiaromaticity.^[^ ^41^, ^42^, ^43^, ^44^, ^45^ ^]^ Therefore, the nature of the distortion in these annulated products is of interest. X‐ray crystallographic analysis revealed that representative compounds **3aa** and **3ab** both exhibit significant deviation from planarity, but in largely different ways (Figure 2).

In N4‐annulated compound **3aa**, the triazine skeleton is distinctly puckered along the N1–N4 axis, with a dihedral angle between the mean planes defined by N1–C8a–C4a–N4 and N1–N2–C3–N4 of 38°, whereas the N1 atom retains near‐planarity with a sum of the bond angles around the N1 atom of 353°. In contrast, N2‐annulated compound **3ab** also features a puckered triazine ring along the N1–N4 axis, with dihedral angles of 34°, but the N1 atom adopts a highly pyramidalized geometry. The sum of the bond angles around N1 atom is 337°, accompanied by a phenyl group oriented nearly perpendicular to the triazine ring. For the crystal structures of other derivatives (**3ca**, **3cb**, **3da**, **3db**, and **4ca**), see the Supporting Information.^[^ ^46^ ^]^

**Figure 3:**
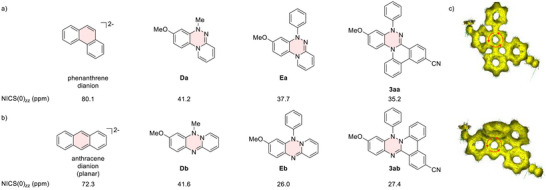
Assessment of the antiaromaticity of a) **3aa** and model compounds, and b) **3ab** and model compounds. NICS(0)_zz_ (ppm) values were calculated at the GIAO‐HF/6‐31+G(d,p) level; the evaluated ring is highlighted. c) ACID plots of **3aa** (top) and **3ab** (bottom).

To elucidate the origins of the distinct distortion modes, the structures of **3aa** and **3ab** were investigated computationally, together with their model compounds, including tricyclic cores **Da** and **Db**, and their N‐phenylated derivatives **Ea** and **Eb** (Figure 3), in conjunction with an assessment of the antiaromaticity of the triazine ring in these systems. The optimized structures of **3aa** and **3ab**, obtained at the CAM‐B3LYP/6‐31G(d) level of theory, were in good agreement with their corresponding crystal structures. Specifically, in the optimized geometries, the dihedral angles of the puckered central triazine rings are 37.3° for **3aa** and 32.9° for **3ab**, respectively.

Within the series of the N4‐annulated compounds, the tricyclic phenanthrene‐type framework **Da** already adopts a puckered conformation in the central ring with a dihedral angle of 32.1° (Figure S59). Notably, while the phenanthrene dianion, isoelectronic with **Da**, also adopts a nonplanar conformation in the optimized structure, the dihedral angle in the central ring, measured in the same manner as in the triazine ring of **Da**, is only 8.9°.^[^ ^47^, ^48^ ^]^ In addition, introducing a phenyl group at the N1 atom in **Ea** does not significantly alter the puckered geometry of the triazine ring, and the N1 atom remains nearly planar. These results therefore suggest that the distorted structure observed in **3aa** is an intrinsic feature of the triazine‐centered tricyclic phenanthrene‐type framework.

To gain deeper insight into the influence of triazine‐ring antiaromaticity, nuclear‐independent chemical shift (NICS) calculations were performed at the GIAO‐HF/6‐31+G(d,p) level of theory (Figure 3a). Because the triazine ring adopts a nonplanar geometry, the NICS(1)_zz_ values differ between its convex and concave faces. Therefore, in this study, we evaluated NICS(0)_zz_ values using a dummy point located at the center of triazine rings.^[^ ^49^, ^50^ ^]^ In the puckered tricyclic core **Da**, the NICS(0)_zz_ value of triazine ring is 41.2 ppm, significantly reduced from that of the isoelectronic phenanthrene dianion (80.1 ppm). Introduction of phenyl group at the N1 atom in **Ea** and fusion of two benzene rings in **3aa** further decrease the NICS(0)_zz_ values to 37.7 ppm and 35.2 ppm, respectively. Despite these pronounced decreases in NICS(0)_zz_ values, anisotropy of the induced current density (ACID) calculation still exhibited a counterclockwise paratropic ring current, confirming that a certain degree of antiaromaticity remains in the triazine ring (Figure 3c). It is noteworthy that, whereas phenanthrene dianion exhibits a global paratropic ring current (Figure S59), in **3aa** the current is more localized in the central triazine ring. This difference likely underlies their distinct nonplanar structures, highlighting the role of the triazine core in enforcing the nonplanar puckered geometry.

In the series of N2‐annulated compounds (Figure 3b), the triazine ring in tricyclic anthracene‐type core **Db** also adopts a puckered conformation, with a dihedral angle of 33.6° and a NICS(0)_zz_ value of 41.6 ppm, comparable to that of **Da**. This is in contrast to the fact that its isoelectronic anthracene dianion takes a planar conformation in the optimized structure. Notably, phenyl substitution at the N1 atom in **Eb** induces significant pyramidalization, with the sum of the bond angles around the N1 atom measuring 337.1°, resulting in a nearly perpendicular orientation of the phenyl group. The fact that **Eb** exhibits such a distorted geometry, similar to that of **3ab**, despite lacking a benzene‐fused structure on the N2‐containing terminal hexagon, indicates that this is an inherent structural feature of the N1‐phenyl triazine‐centered anthracene‐type tricyclic framework. As a consequence of this structural distortion, the NICS(0)_zz_ value decreased to 26.0 ppm in **Eb** and a comparable value (27.4 ppm) is retained in **3ab**, where ACID calculation also revealed a weak paratropic ring current (Figure 3c). In addition, the different substitution patterns in **3ca**, **3cb**, **3da** and **3db** did not have a significant influence on the respective NICS(0)_zz_ values (Figure S60).

In contrast to the puckered geometry of the triazine ring observed in the Blatter N2‐ or N4‐annulation products, the tetrazinone core of the verdazyl annulation product **4ca** adopts a distinct nonplanar conformation, characterized by pronounced pyramidalization at the N2 atom seen in the crystal structure (Figure S8). Considering the carbonyl carbon as a carbocation equivalent, the tetrazinone ring can also be viewed as an 8π‐electron system. Indeed, the calculated NICS(0)_zz_ value for the tetrazinone core in **4ca** is 28.5 ppm, only slightly higher than that for the Blatter annulation product **3ab**. This suggests that the tetrazinone ring may retain its antiaromatic character even when the carbonyl group is incorporated into the ring framework.

**Figure 4:**
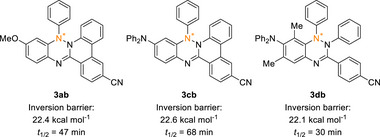
Inversion barriers of **3ab**, **3cb,** and **3db**, estimated from racemization half‐lives of their separated enantiomers.

For the Blatter N2‐annulation products, pronounced pyramidalization at the N1 atom, combined with unsymmetrical substitution, gives rise to chirality at the nitrogen center. Because the separation of N‐chiral enantiomers is generally challenging due to the low activation barrier for pyramidal inversion at nitrogen, considerable effort has been devoted to increasing this barrier, through steric congestion, ring strain, or incorporation of electronegative substituents. Nevertheless, reliable strategies for installing chiral nitrogen centers remain underdeveloped.^[^ ^32^, ^33^, ^51^, ^52^, ^53^, ^54^, ^55^ ^]^ We envisioned that the antiaromatic character of the triazine ring might enhance the inversion barrier sufficiently to allow separation of N‐chiral enantiomers. To test this hypothesis, we tried to separate the Blatter N2‐annulation products using chiral HPLC (for details, see Supporting Information). The enantiomers of **3ab** were successfully resolved using a chiralcel OJ‐RH reversed phase HPLC column. Monitoring the enantiomeric excess of the isolated enantiomers in solution over time revealed a racemization half‐life of 47 min, corresponding to an inversion barrier of 22.4 kcal mol^−1^ (Figure 4). We also achieved separation of the enantiomers of **3cb** and **3db**, which exhibited comparable inversion barriers, 22.6 and 22.1 kcal mol^−1^ (half‐lives: 68 and 30 min), respectively. However, while the verdazyl annulation product **4ca** also features chirality at the N1 atom, its enantiomers could not be separated.

**Figure 5:**
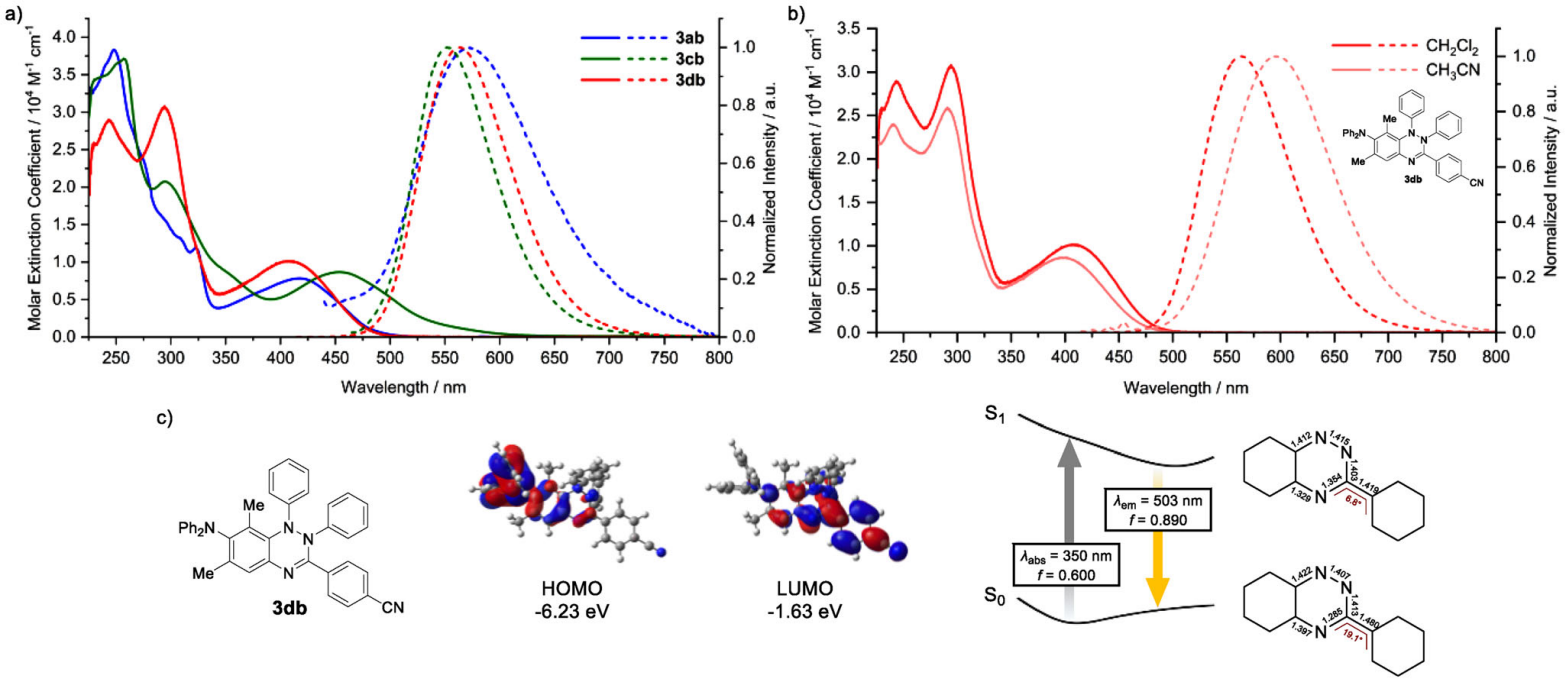
a) UV–vis absorption (solid lines) and fluorescence (dashed lines) spectra of **3ab** (blue), **3cb** (green), and **3db** (red) in CH_2_Cl_2_. b) UV–vis (solid lines) and fluorescence (dashed lines) spectra of **3db** in CH_2_Cl_2_ and CH_3_CN. c) Optimized structure of **3db** in the S_1_ state, together with its molecular orbitals relevant to the electronic transition.

Among the compounds examined, whereas the Blatter N4‐annulation products and the verdazyl annulation products did not exhibit notable photophysical properties, the Blatter N2‐annulation products displayed distinct emission in the visible region. Specifically, in CH_2_Cl_2_, **3aa** showed only a weak absorption tail extending to 600 nm, and the verdazyl annulation product **4ca** exhibited a weak absorption band with a maximum wavelength (*λ*_abs_) at 355 nm; both compounds were essentially non‐emissive or only weakly emissive, respectively (Table S2). In contrast, the Blatter N2‐annulation product **3ab** exhibited *λ*_abs_ = 418 nm with a molar absorption coefficient (*ε*) of 0.78 × 10^4^ M^−1^cm^−1^ and showed weak green emission with a maximum (*λ*_em_) at 544 nm (Figure 5a). Introduction of an electron‐donating *N,N*‐diphenylamino group at the 7‐position in **3cb** led to a red‐shift in the absorption spectrum to *λ*_abs_ = 453 nm, whereas its emission band shifted only slightly to 553 nm, accompanied by a low fluorescence quantum yield (*Φ*_F_) of 0.01. By contrast, the N2‐phenyl non‐fused derivative **3db** exhibited intense emission at 564 nm with a significantly higher *Φ*_F_ of 0.43, whereas its absorption band was blue‐shifted to 407 nm. The Stokes shift of this compound amounts to 6840 cm^−1^ in CH_2_Cl_2_, and it displayed solvatochromism in its fluorescence spectra (*λ*_em_ = 599 nm in CH_3_CN, Figure 5b). These observations suggest substantial structural reorganization in the excited state.

To gain further insight into the excited‐state behavior in **3db**, geometry optimization was performed in the lowest singlet excited state (S_1_) at the CAM‐B3LYP/6‐31G(d) level, incorporating CH_2_Cl_2_ as the solvent via the conductor‐like polarizable continuum model (CPCM). The emission was assigned to a HOMO–LUMO transition in the S_1_‐optimized geometry, with the HOMO predominantly localized on the diphenylaminophenyl–imino moiety and the LUMO on the cyanophenyl–imino–phenyl moiety, imparting a distinct intramolecular charge‐transfer character (Figure 5c). The calculations indicated that excitation from S_0_ to S_1_ induces notable structural changes. In the imino‐phenyl moiety, the C═N bond is elongated from 1.285 to 1.354 Å, whereas the adjacent C─N and C─C bonds are shortened from 1.397 to 1.329 Å and from 1.480 to 1.419 Å, respectively. In addition, the torsion angle of the imino‐phenyl moiety decreases from 19.1° to 6.8°, resulting in planarization. Notably, the oscillator strength for this transition (*f *= 0.890) was enhanced compared to that in the ground state (*f* = 0.600 in S_0_), which likely accounts for the high fluorescence quantum yield.

Blatter and verdazyl radicals are well known for their intriguing electrochemical properties, typically exhibiting reversible oxidation and reduction, making them promising candidates for flow battery applications.^[^ ^26^, ^27^, ^35^, ^56^, ^57^, ^58^, ^59^ ^]^ Motivated by this, we examined the electrochemical behavior of the Blatter and verdazyl annulation products. Cyclic voltammetry measurements were conducted for a series of compounds, including **3aa**, **3ab**, **3ca**, **3cb**, **4ca**, and **4cb**, in CH_3_CN using *n*‐Bu_4_NPF_6_ (0.1 M) as the supporting electrolyte (for all data, see Tables S3 and S4).

**Figure 6:**
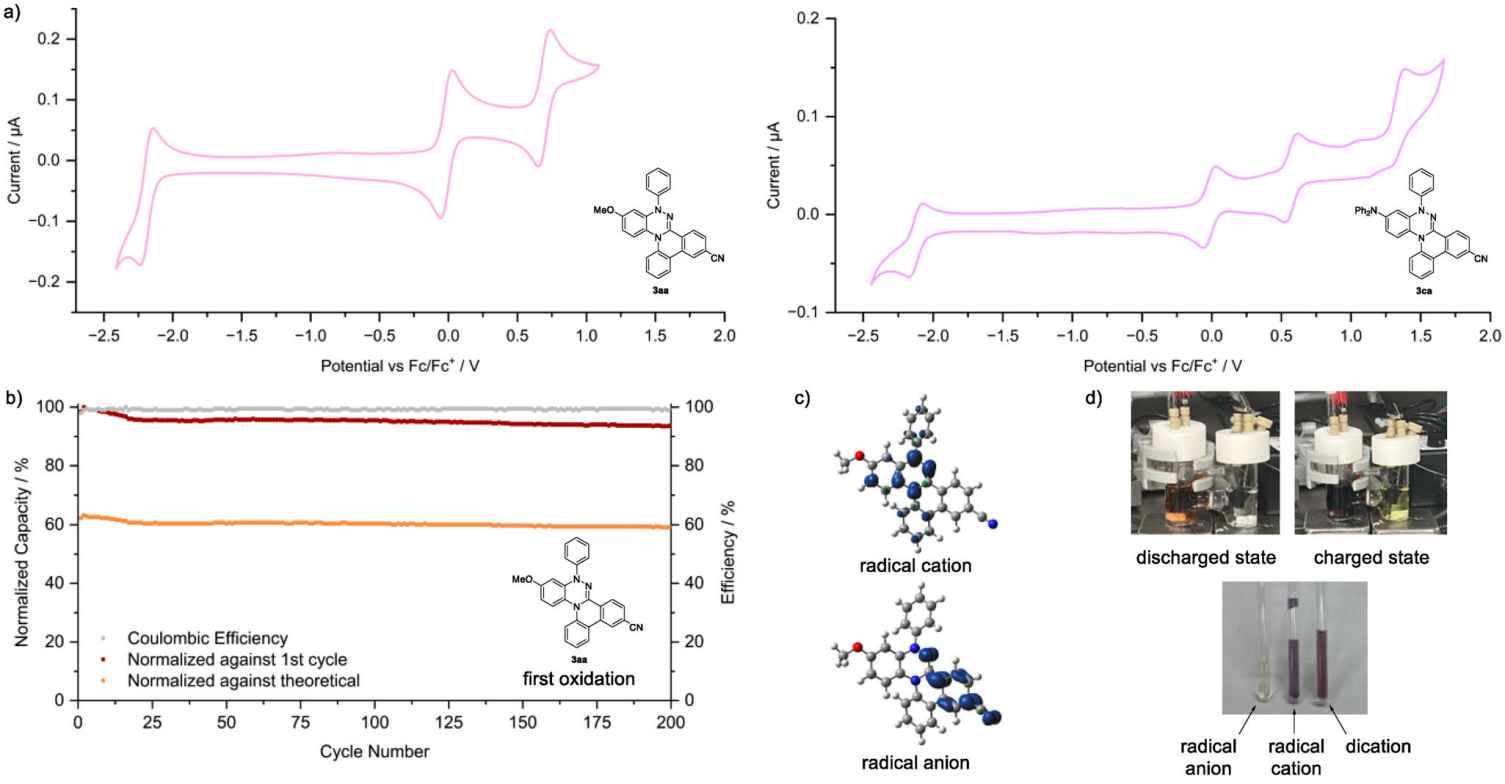
a) Cyclic voltammograms (0.5 mM in 0.1 m NBu_4_PF_6_/CH_3_CN) of **3aa** and **3ca** with a scan rate of 200 mV s^−1^. b) Plot of discharge capacity (normalized to theoretical capacity and the first discharge cycle) and coulombic efficiency versus cycle number for H‐cell cycling of the first oxidation of **3aa** (0.5 mm in 0.1 m NBu_4_PF_6_/CH_3_CN). c) Spin densities of the radical cation and the radical anion of **3aa**, calculated on the CAM‐UB3LYP/6‐31G(d) level of theory including CH_3_CN using the CPCM. d) Visual appearance of discharged and charged state during H‐cell cycling of first oxidation of **3aa** (top, left half‐cell) and electrochemically synthesized redox states of **3aa** (bottom).

Among the Blatter N4‐annulated, Blatter N2‐annulated, and verdazyl annulated products, a marked difference was observed in their oxidation potentials, reflecting the different HOMO characteristics revealed by DFT calculations (Figure S58). Specifically, N4‐annulated **3aa** exhibited two reversible oxidations (*E*_1/2,ox1_ = +0.03 V, *E*_1/2,ox2_ = +0.74 V versus Fc/Fc⁺) and one reversible reduction (*E*_1/2,red_ = −2.15 V versus Fc/Fc⁺) (Figure 6a). The first oxidation potential of **3aa** was significantly cathodically shifted compared to those of N2‐annulated **3ab** (*E*_1/2,ox1_ = +0.50 V versus Fc/Fc⁺) and verdazyl annulation product **4ca** (*E*_1/2,ox1_ = +0.64 V versus Fc/Fc⁺). Another notable feature of **3aa** was the large separation between its first and second oxidation potentials, much greater than those observed for **3ab**. Replacing the methoxy group in **3aa** with a diphenylamino group to give **3ca** had little influence on the overall electrochemical profile, which still showed two reversible oxidations and one reversible reduction (*E*_1/2,ox1_ = −0.01 V, *E*_1/2,ox2_ = +0.57 V, *E*_1/2,red_ = −2.13 V versus Fc/Fc⁺). However, **3ca** also displayed an additional irreversible oxidation, attributable to oxidation of the diphenylamine moiety.

The ability of **3aa** to exhibit two reversible oxidations and one reversible reduction within a wide electrochemical window is particularly compelling for potential battery applications; therefore, we subsequently focused on exploring its utilization. The long‐term reversibility of the oxidations and reduction of **3aa** was studied with H‐cell cyclings. All cyclings were performed with a constant current of 0.5 mA over 200 cycles. The H‐cell cycling of the first oxidation of **3aa** seemed promising for battery application as 95% of capacity referred to the first discharge cycle could be retained after 200 cycles which corresponds to a retention of 59% of the theoretical capacity (Figure 6b). However, during the H‐cell cycling of the second oxidation the capacity decreased fast with only 7% of capacity referred to the first discharge cycle retained after 200 cycles and also the cycling of the reduction failed. During the reduction cycling the first charging process lasted longer than the theoretical value and afterwards the first discharging process ended fast (see Supporting Information). Probably the reduction event below −2.4 V, which is occurring at a potential close to the reversible one, took place, leading to a diminished cycling performance. Instead, next the reversibility of the reduction of **3ca** was tested in the H‐cell as for this compound, the additional reduction is better separated from the reversible one. But, also the H‐cell cycling of this reduction performed poorly as only 10% of capacity referred to the first discharge cycle could be retained after 200 cycles. Despite this, the performance of the first oxidation of **3aa** makes it a promising candidate as a catholyte in electrochemical application. However, there is still potential for future improvement of the reversibility of the oxidations and the reduction by varying functional groups.

The different obtained redox states of **3aa** could further also be isolated. The radical cation of **3aa** which is stable under air could be generated by chemical oxidation with NOBF_4_ or Na_2_S_2_O_8_ (for details see Supporting Information). The other redox states were only accessible by electrochemical oxidation or reduction and the presence of radical or non‐radical species was confirmed by EPR signals of the radicals and an ^1^H‐NMR of the dicationic species. Interestingly, analysis of the hyperfine couplings of the EPR signals of the radical cation and the radical anion of **3aa** suggests that the radical anion is more localized than the radical cation which is also supported by DFT calculation of the respective spin densities (Figure 6c). Another intriguing aspect that caught our attention was the electrochromism of **3aa** which could potentially make it useful for application as sensors, photocatalysts or similar. As observed during the H‐cell cycling as well as the isolation of the redox states the radical cation and the dication of **3aa** show a violet and dark purple color whereas the radical anion appears light red as the neutral molecule (Figure 6d).

## Conclusion

A novel annulation reaction of Blatter and verdazyl radicals with arynes was developed which proceeds via radical addition of the stable radical to the aryne, radical cyclization and subsequent oxidation. This constitutes a new radical reaction mode of arynes. During the annulation reaction of the aryne with a Blatter radical two isomers after reaction at N2 or N4 of the triazine core were observed, for the reaction with verdazyl radicals the annulation with the arene attached to carbon in the tetrazinone ring was favored. Different electron‐donating and electron‐withdrawing groups at the Blatter radical and the aryne reaction component with varying steric hindrance were tolerated. The synthesized triazines and tetrazinones are of particular interest as they are antiaromatic and chiral at nitrogen. They possess moderate antiaromaticity as determined through calculation of the NICS(0)_zz_ values and the enantiomers of the triazines could be separated by chiral HPLC. However, with inversion barriers in the range of 22 to 23 kcal mol^−1^ the enantiomers are not long‐term stable and racemization occurs at room temperature. In addition, the obtained different isomers show distinctly unique characteristics. One triazine isomer possesses interesting photophysical properties as it shows strong absorption in the visible region and emission, whereas the other triazine isomer is interesting in terms of electrochemical application due to obtained reversible reduction and oxidation. Taken together, a novel synthesis of antiaromatic and N‐chiral triazines and tetrazinones which are promising compounds for materials science was developed.

## Conflict of Interests

The authors declare no conflict of interest.

## Supporting information



Supporting Information

Supporting Information

## Data Availability

The data that support the findings of this study are available in the Supporting Information of this article.

## References

[1] N. Kim , M. Choi , S.‐E. Suh , D. M. Chenoweth , Chem. Rev. 2024, 124, 11435–11522, 10.1021/acs.chemrev.4c00296. 39383091

[2] H. H. Wenk , M. Winkler , W. Sander , Angew. Chem. Int. Ed. 2003, 115, 502–528 10.1002/anie.200390151 12569480

[3] R. Sanz , Org. Prep. Proced. Int. 2008, 40, 215–291, 10.1080/00304940809458089.

[4] C. M. Gampe , E. M. Carreira , Angew. Chem. Int. Ed. 2012, 124, 3766–3778 10.1002/anie.201107485 22422638

[5] A. Bhunia , S. R. Yetra , A. T. Biju , Chem. Soc. Rev. 2012, 41, 3140, 10.1039/c2cs15310f. 22278415

[6] J. Shi , L. Li , Y. Li , Chem. Rev. 2021, 121, 3892–4044, 10.1021/acs.chemrev.0c01011. 33599472

[7] P. G. Gassman , G. D. Richmond , J. Am. Chem. Soc. 1970, 92, 2090–2096, 10.1021/ja00710a049.

[8] S. Zhou , G. M. Anderson , B. Mondal , E. Doni , V. Ironmonger , M. Kranz , T. Tuttle , J. A. Murphy , Chem. Sci. 2014, 5, 476–482, 10.1039/C3SC52315B.

[9] S. Yamabe , T. Minato , A. Ishiwata , O. Irinamihira , T. Machiguchi , J. Org. Chem. 2007, 72, 2832–2841, 10.1021/jo062256e. 17367189

[10] U. N. Rao , E. Biehl , J. Org. Chem. 2002, 67, 3409–3411, 10.1021/jo016407j. 12003553

[11] V. Usieli , S. Sarel , J. Org. Chem. 1973, 38, 1703–1708, 10.1021/jo00949a019.

[12] K. Okuma , S. Sonoda , Y. Koga , K. Shioji , J. Chem. Soc., Perkin Trans. 1 1999, 2997–3000, 10.1039/a904204k.

[13] X. Yang , G. C. Tsui , Chem. Sci. 2018, 9, 8871–8875, 10.1039/C8SC03754J. 30627405 PMC6296300

[14] U. N. Rao , R. Sathunuru , J. A. Maguire , E. Biehl , J. Heterocycl. Chem. 2004, 41, 13–21.

[15] Y. Hu , J. Ma , L. Li , Q. Hu , S. Lv , B. Liu , S. Wang , Chem. Commun. 2017, 53, 1542–1545, 10.1039/C6CC09732D. 28094359

[16] K. Koyamada , K. Miyamoto , M. Uchiyama , Nat. Synth. 2024, 3, 1083–1090, 10.1038/s44160-024-00572-y.

[17] M. Scherübl , C. G. Daniliuc , A. Studer , Angew. Chem. Int. Ed. 2021, 133, 711–715 10.1002/anie.202012654 PMC7839731 33038065

[18] D. Bhattacharya , M. Scherübl , C. G. Daniliuc , A. Studer , Chem. Sci. 2024, 15, 13712–13716, 10.1039/D4SC04369C. 39211489 PMC11351772

[19] T. Kubo , M. Abe , Chem. Rev. 2024, 124, 4541–4542, 10.1021/acs.chemrev.3c00893. 38654681

[20] C. Shu , Z. Yang , A. Rajca , Chem. Rev. 2023, 123, 11954–12003, 10.1021/acs.chemrev.3c00406. 37831948

[21] K. Hatakeyama‐Sato , K. Oyaizu , Chem. Rev. 2023, 123, 11336–11391, 10.1021/acs.chemrev.3c00172. 37695670

[22] Z. X. Chen , Y. Li , F. Huang , Chem. 2021, 7, 288–332, 10.1016/j.chempr.2020.09.024.

[23] M. Gomberg , J. Am. Chem. Soc. 1900, 22, 757–771, 10.1021/ja02049a006.

[24] S. Gao , F. Li , Adv. Funct. Mat. 2023, 33, 2304291, 10.1002/adfm.202304291.

[25] Y. Ji , L. Long , Y. Zheng , Mater. Chem. Front. 2020, 4, 3433–3443, 10.1039/D0QM00122H.

[26] F. J. M. Rogers , P. L. Norcott , M. L. Coote , Org. Biomol. Chem. 2020, 18, 8255–8277, 10.1039/D0OB01394C. 33001120

[27] M. Duggin , A. C. Bissember , R. O. Fuller , Chem. Asian J. 2025, 20, e202401550. 39843396 10.1002/asia.202401550

[28] G. N. Lipunova , T. G. Fedorchenko , O. N. Chupakhin , Russ. Chem. Rev. 2013, 82, 701–734, 10.1070/RC2013v082n08ABEH004341.

[29] G. N. Lipunova , T. G. Fedorchenko , A. V. Shchepochkin , O. N. Chupakhin , Russ. Chem. Bull. 2025, 74, 328–353, 10.1007/s11172-025-4526-5.

[30] B. D. Koivisto , R. G. Hicks , Coord. Chem. Rev. 2005, 249, 2612–2630, 10.1016/j.ccr.2005.03.012.

[31] G. N. Lipunova , T. G. Fedorchenko , A. N. Tsmokalyuk , O. N. Chupakhin , Russ. Chem. Bull. 2020, 69, 1203–1222, 10.1007/s11172-020-2892-6.

[32] H. Kessler , Naturwissenschaften 1971, 58, 46–51, 10.1007/BF00620801.

[33] J. M. Lehn , in Dynamic Stereochemistry, Springer, Berlin Heidelberg, Berlin, Heidelberg, 1970, pp. 311–377, 10.1007/BFb0050818.

[34] A. B. Buda , T. A. der Heyde , K. Mislow , Angew. Chem. Int. Ed. 1992, 104, 989–1007

[35] J. S. Steen , F. de Vries , J. Hjelm , E. Otten , ChemPhysChem. 2023, 24, e202200779. 36317641 10.1002/cphc.202200779

[36] E. Picazo , K. N. Houk , N. K. Garg , Tetrahedron Lett. 2015, 56, 3511–3514, 10.1016/j.tetlet.2015.01.022. 26034336 PMC4448725

[37] F. M. Bickelhaupt , K. N. Houk , Angew. Chem. Int. Ed. 2017, 129, 10070–10086 10.1002/anie.201701486 PMC5601271 28447369

[38] J. M. Medina , J. L. Mackey , N. K. Garg , K. N. Houk , J. Am. Chem. Soc. 2014, 136, 15798–15805, 10.1021/ja5099935. 25303232 PMC4221504

[39] Y. Himeshima , T. Sonoda , H. Kobayashi , Chem. Lett. 1983, 12, 1211–1214, 10.1246/cl.1983.1211.

[40] F. A. Neugebauer , I. Umminger , Chem. Ber. 1980, 113, 1205–1225, 10.1002/cber.19801130402.

[41] C. Yuan , S. Saito , C. Camacho , S. Irle , I. Hisaki , S. Yamaguchi , J. Am. Chem. Soc. 2013, 135, 8842–8845, 10.1021/ja404198h. 23721361

[42] Z. Zhang , Y.‐S. Wu , K.‐C. Tang , C.‐L. Chen , J.‐W. Ho , J. Su , H. Tian , P.‐T. Chou , J. Am. Chem. Soc. 2015, 137, 8509–8520, 10.1021/jacs.5b03491. 26075574

[43] M. Ueda , K. Jorner , Y. M. Sung , T. Mori , Q. Xiao , D. Kim , H. Ottosson , T. Aida , Y. Itoh , Nat. Commun. 2017, 8, 346, 10.1038/s41467-017-00382-1. 28839142 PMC5570949

[44] R. Kotani , L. Liu , P. Kumar , H. Kuramochi , T. Tahara , P. Liu , A. Osuka , P. B. Karadakov , S. Saito , J. Am. Chem. Soc. 2020, 142, 14985–14992, 10.1021/jacs.0c05611. 32786754

[45] Y. Chen , K.‐H. Chang , F.‐Y. Meng , S.‐M. Tseng , P.‐T. Chou , Angew. Chem. Int. Ed. 2021, 60, 7205–7212, 10.1002/anie.202015274. 33381896

[46] Deposition numbers 2452331 (for **3aa**), 2452332 (for **3ab**), 2451504 (for **3ca**), 2451505 (for **3cb**), 2451506 (for **3da**), 2451507 (for **3db**) and 2451508 (for **4ca**). These data are provided free of charge by the joint Cambridge Crystallographic Data Centre and Fachinformationszentrum Karlsruhe Access Structures service.

[47] R. Frim , A. Mannschreck , M. Rabinovitz , Angew. Chem. Int. Ed. 1990, 29, 919–921

[48] A. Ioffe , A. Ayalon , M. Rabinovitz , J. Chem. Soc. Perkin Trans. 1994, 2, 1115, 10.1039/p29940001115.

[49] Y. Chen , S.‐M. Tseng , K.‐H. Chang , P.‐T. Chou , J. Am. Chem. Soc. 2022, 144, 1748–1757, 10.1021/jacs.1c11231. 35067055

[50] Y. Yoneda , T. Konishi , K. Suga , S. Saito , H. Kuramochi , J. Am. Chem. Soc. 2025, 147, 12051–12060, 10.1021/jacs.4c18623. 40059351 PMC11987032

[51] S. K. Zaitseva Valentin , Synthesis 2024, 57, 1237–1254.

[52] N. Vystavkin , C. Teskey , Nachr. Chem. 2023, 71, 54–56, 10.1002/nadc.20234134055.

[53] C. Lee , J. M. Weber , L. E. Rodriguez , R. Y. Sheppard , L. M. Barge , E. L. Berger , A. S. Burton , Symmetry 2022, 14, 460–508.

[54] F. A. Davis , R. H. Jr. Jenkins , in Asymmetric Synthesis, Academic Press, San Diego, 1984, pp. 313–353.

[55] A. Rauk , L. C. Allen , K. Mislow , Angew. Chem. Int. Ed. 1970, 82, 400–414

[56] G. D. Charlton , S. M. Barbon , J. B. Gilroy , C. A. Dyker , J. Ener. Chem. 2019, 34, 52–56, 10.1016/j.jechem.2018.09.020.

[57] A. Saal , L. Elbinger , K. Schreyer , X. Fataj , C. Friebe , U. S. Schubert , ACS Appl. Ener. Mater. 2022, 5, 15019–15028, 10.1021/acsaem.2c02559.

[58] J. S. Steen , J. L. Nuismer , V. Eiva , A. E. T. Wiglema , N. Daub , J. Hjelm , E. Otten , J. Am. Chem. Soc. 2022, 144, 5051–5058, 10.1021/jacs.1c13543. 35258956 PMC8949756

[59] A. Korshunov , M. J. Milner , M. Grünebaum , A. Studer , M. Winter , I. Cekic‐Laskovic , J. Mater. Chem. A 2020, 8, 22280–22291, 10.1039/D0TA07891C.

