# Supplementary material for: Reaction of Blatter and Verdazyl Radicals with Arynes: Synthesis and Investigation of N‐Chiral, Antiaromatic Triazines, and Tetrazinones
Source: Angew Chem Int Ed Engl. 2025 Nov 10;65(1):e20021. doi: 10.1002/anie.202520021 (PMC12759216; doi:10.1002/anie.202520021)
Supplement: Supplementary file 1 — Supporting Information [file ANIE-65-e20021-s001.pdf]

Reaction of Blatter and Verdazyl Radicals with Arynes: Synthesis and Investigation of N-Chiral, Antiaromatic Triazines, and Tetrazinones

Lena Lezius[a], Elena S. Horst[a], Maximilian Scherübl[a], Jessika Lammert[a], Constantin G. Daniliuc[a], Tatsuya Mori[b], Shigehiro Yamaguchi*[b] and Armido Studer*[a]

[a] University of Münster, Germany; [b] Nagoya University, Japan

Supporting Information

Table of Contents

1. General Information 1
2. Reaction Optimization 2
3. Synthesis and Characterization of the Starting Materials 4
4. Synthesis and Characterization of the Products 19
5. X-Ray Analysis 34
6. Photophysical Measurements 42
7. Electrochemical Measurements 46
   7.1 Cyclic Voltammetry 46
   7.2 H-Cell Cyclings 49
   7.3 Synthesis and Characterization of Different Redox States of 3aa 52
   7.4 Spectroelectrochemical Measurements 55
8. Investigation of Inversion Barriers and Half-life Times 58
   8.1 Separation of the Enantiomers on the Chiral HPLC 58
   8.2 Calculation of Inversion Barriers and Half-life Times 60
9. DFT Calculations 68
10. NMR Spectra 80
11. References 106

1. General Information

All Reactions involving air- or moisture-sensitive reagents or intermediates were carried out under an Argon atmosphere using Schlenk techniques. Glassware was dried at 105 °C for at least 12 h before use. All commercially available chemicals were purchased from ABCR, Acros Organics, Alfa Aesar, BLDpharm, Fluorochem, Merck, Sigma Aldrich and TCI and used without further purification. Solvents for reactions were dried using standard methods (THF over K, Et_2O over a Na/K-alloy, DCM over P_2O_5) and distilled prior to use. Alternatively, solvents dried over a molecular sieve and stored under an Argon atmosphere were purchased from Acros Organics („Extra Dry over Molecular Sieve, AcroSeal"). Solvents for extractions and column chromatography were distilled before use. Thin layer chromatography (TLC) was performed with Silica 60 F254 glass plates purchased from Merck. Compounds were detected by excitation with UV light (λ = 254 nm) or by immersion into a $KMnO_4$ solution (1.5 g $KMnO_4$, 5.0 g $NaHCO_3$ in 200 mL water). Column chromatography was performed using Merck Silica Gel 60 (40-63 μm) or Acros Organics Silica Gel (35-70 μm) with an air-pressure of up to 0.5 bar. Purification control by Reversed Phase HPLC was performed with Agilent Series 1200 HPLC device with an Agilent Eclipse XDB-C18 column (4.6 x 150 mm x 5 µm) using a CH_3CN/H_2O mixture as solvent.^{1}H-, ^{13}C- and ^{19}F-NMR-spectra were recorded on a Bruker Avance II 300, a Bruker Neo 400, an Agilent DD2 500 or an Agilent DD2 600 at 300 K (unless otherwise stated). The chemical shifts δ (ppm) were referenced to the solvent residual peak of the deuterated solvent ($CDCl_3$ (^{1}H-NMR: δ = 7.26 ppm; ^{13}C-NMR: δ = 77.2 ppm), CD_2Cl_2 (^{1}H-NMR: δ = 5.32 ppm, ^{13}C-NMR: δ = 53.8 ppm), Acetone-d_6 (^{1}H-NMR: δ = 2.05 ppm; ^{13}C: δ = 29.84 ppm), DMSO-d_6 (^{1}H-NMR: δ = 2.50 ppm; ^{13}C-NMR: δ = 39.52 ppm). The multiplicities are marked as s (singlet), d (dublet), t (triplet), q (quartet), p (pentet), h (heptet) and m (multiplet) or a combination of the before mentioned. Coupling constants *J* are reported in Hertz (Hz). HRMS (ESI) spectra were recorded with a Thermo Fisher Scientific LTQ XL Orbitrap or a Thermo Fisher Scientific Orbitrap. MassLinx 4.0 of Water-Micromass was used for data analysis. Note for NMR spectra: The signals pointing down at 110 ppm are measurement artefacts. All given HR masses were checked using an HRMS checker.[1] IR-spectra were recorded with a JASCO-FT-IR-4X spectrometer. The wave numbers ($\tilde{\nu}$) of the absorption bands are given in cm^{-1}. Melting points were measured with a TA Instruments Q20 DSC device.

2. Reaction Optimization

General Information: All solid compounds were dried *in vacuo* overnight. Afterwards the aryne precursor **1a** and dry solvent were added and the reaction mixture was stirred at room temperature or 50 °C for 1.5 . The solvent was removed under reduced pressure and the crude product was purified by column chromatography (SiO_2, Et_2O/pentane).

Results: We optimized the annulation reaction starting with aryne precursor **1a**, Blatter radical **2a** and CsF for the synthesis of **3aa** and **3ab** (Table S1, entry 1). Performing the reaction at 50 °C did not improve the yield (entry 2) and in other solvents no distinct product formation was observed (entries 3 – 5). Lowering the equivalents of the Blatter radical led to a comparable yield of both isomers (entry 6), with a lower reaction concentration the total yield dropped to 38%. The best results with a total yield of around 90% were obtained with a higher amount of CsF (4.0 equiv.) together with 18-crown-6 (3.0 equiv.) as an additive (entry 8) or by increasing the equivalents of the aryne precursor and CsF to 2.0 equiv. and 6.0 equiv. (entry 9). Thereby, the selectivity was higher without the addition of the crown ether. With even more equivalents of CsF and 18-crown-6 as an additive the yield decreased (entry 10) and also adding DDQ as an external oxidant turned out to be less efficient (entry 11). When the reaction mixture was degassed with three freeze-pump-thaw cycles a lower total yield of 72% was obtained suggesting that residual oxygen takes part in the reaction (entry 12). It is striking that in most test reactions a higher yield of **3aa** in comparison to **3ab** was obtained which can be explained with the larger steric hindrance at N2 of the triazine core of the Blatter radical. As the total yield of the test reactions in entries 8 and 9 was similar we decided to proceed with the conditions used in entry 9 where no additional reagent was needed.

Table S1. Optimization of the reaction conditions.[a]

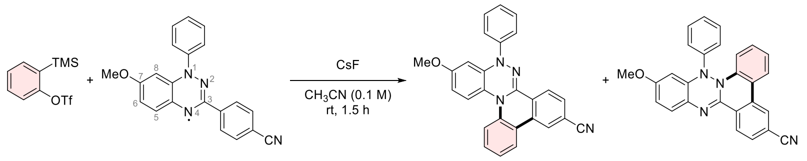

Entry	Equiv. Aryne	Equiv. Blatter	Equiv. CsF	Deviation from standard conditions	Yield 3aa[b]	Yield 3ab[b]
1	1.0	2.0	3.0	/	40%	21%
2	1.0	2.0	3.0	50 °C	37%	18%
3	1.0	2.0	3.0	THF	traces	/
4	1.0	2.0	3.0	Et_2O	/	/
5	1.0	2.0	3.0	Toluene	/	/
6	1.0	1.0	3.0	/	39%	25%
7	1.0	1.0	3.0	CH_3CN (0.05 M)	26%	12%
8	1.0	1.0	4.0	18-crown-6 (3.0 equiv.)	**56%**	**34%**
9	2.0	1.0	6.0	/	**66%**	**25%**
10	2.0	1.0	6.0	18-crown-6 (3.0 equiv.)	47%	18%
11	2.0	1.0	6.0	DDQ (1.0 equiv.)	23%	27%
12	2.0	1.0	6.0	Degassed	51%	21%

[a] Unless otherwise noted, all reactions were performed with **1a**, **2a** and CsF on a 50 μmol scale of the limiting reagent in CH_3CN (0.5 mL) at room temperature under argon atmosphere for 1.5 h. [b] Isolated yields are given.

3. Synthesis and Characterization of the Starting Materials

The stable radicals **2a**, **2c**, **2d, 2h, 2i** and **2k** were synthesized in this study and the procedures are shown below. All other Blatter and verdazyl radicals were used from previous projects of our group.[2,3] Also, aryne precursor **1b** was used from a previous project.[4]

Overview of used starting materials:

Aryne precursors:

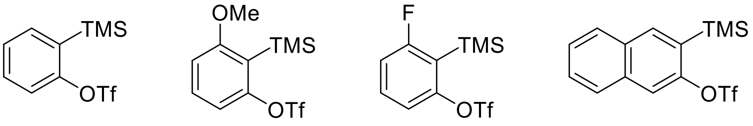

1a **1b** **1c** **1d**

Blatter radicals:

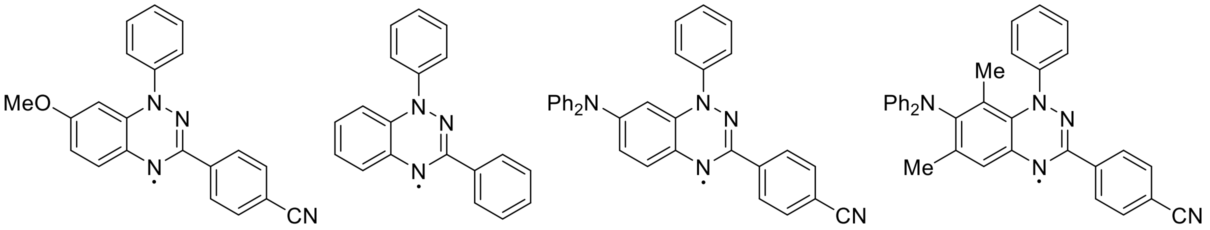

2a **2b** **2c** **2d**

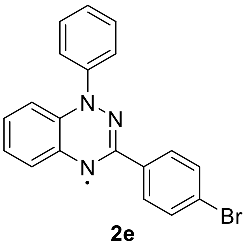

2e

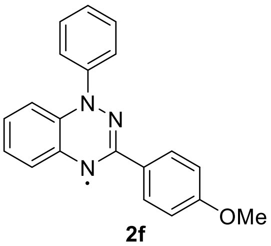

2f

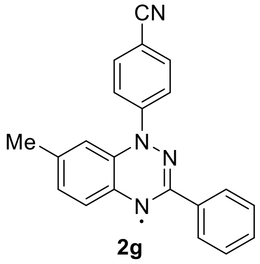

2g

Verdazyl radicals:

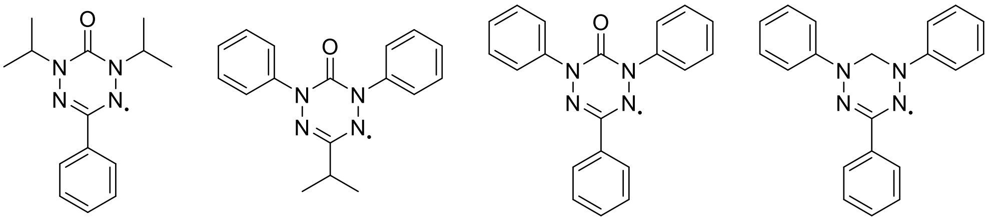

2h **2i** **2j** **2k**

Figure S1. Overview of used starting materials.

2-(Trimethylsilyl)phenyl trifluoromethanesulfonate (**1a**)

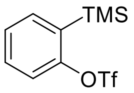

1a

According to a literature procedure,[4] 1-bromophenol (3.2 mL, 30 mmol, 1.0 equiv.) and bis(trimethylsilyl)amine (9.3 mL, 45 mmol, 1.5 equiv.) were suspended in dry THF (60 mL) and the mixture was stirred at 80 °C for 18 h. The volatile components were removed *in vacuo* and the residue was dissolved in dry THF (70 mL). Afterwards, the mixture was cooled to -78 °C and at that temperature *n*BuLi (1.6 M in hexanes, 20.6 mL, 33.0 mmol, 1.1 equiv.) was added dropwise. After 30 min, trifluoromethanesulfonic anhydride (7.1 mL, 45 mmol, 1.5 equiv.) was added slowly and the mixture was stirred for an additional hour. The reaction was quenched by addition of sat. aq. NH_4Cl solution (8 mL) and diluted with water (130 mL). The aqueous phase was extracted with Et_2O (3 x 130 mL) and the combined organic phases were washed with brine (130 mL) and dried over $MgSO_4$. The solvent was removed under reduced pressure and the crude product was purified by column chromatography (SiO_2, pentane). The title compound was obtained as a colorless liquid (4.29 g, 13.4 mmol, 44%).

^{1}H-NMR (300 MHz, $CDCl_3$): δ (ppm) = 7.54 (dd, *J* = 7.5, 2.0 Hz, 1H), 7.49 – 7.40 (m, 1H), 7.38 – 7.31 (m, 2H), 0.37 (s, 9H).

^{19}F-NMR {^{13}C} (282 MHz, $CDCl_3$): δ (ppm) = -73.95.

^{13}C-NMR (101 MHz, $CDCl_3$): δ (ppm) = 155.3, 136.4, 132.7, 131.4, 127.6, 119.7 (q, *J* = 1.6 Hz), 118.7 (q, *J* = 319.9 Hz), -0.7.

Data are in accordance with those reported in literature.[4]

3-Fluoro-2-(trimethylsilyl)phenyl trifluoromethanesulfonate (**1c**)

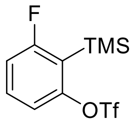

1c

According to a literature procedure,[4] 2-bromo-3-fluorophenol (0.50 mL, 4.6 mmol, 1.0 equiv.) and bis(trimethylsilyl)amine (1.4 mL, 6.9 mmol, 1.5 equiv.) were dissolved in dry THF (10 mL) and the mixture was refluxed for 18 h. Afterwards, the mixture was concentrated *in vacuo* and the residue was dissolved in dry THF (15 mL). The reaction mixture was cooled to -78 °C and at that temperature *n*BuLi (1.6 M in hexanes, 3.2 mL, 5.1 mmol, 1.1 equiv.) was added dropwise. After stirring for 30 min, triflic anhydride (1.2 mL, 6.9 mmol, 1.5 equiv.) was added slowly and the mixture was stirred for an additional hour. The reaction was quenched by addition of sat. aq. NH_4Cl solution (15 mL), diluted with water (50 mL) and extracted with Et_2O (3 x 50 mL). The combined organic phases were washed with brine (50 mL), dried over $MgSO_4$ and concentrated under reduced pressure. After purification by column chromatography (SiO_2, pentane) the title compound was obtained as a colorless liquid (0.34 g, 1.1 mmol, 24%).

^{1}H-NMR (300 MHz, CDCl$_3$): δ (ppm) = 7.46 – 7.36 (m, 1H), 7.15 (d, *J* = 8.3 Hz, 1H), 7.03 (tq, *J* = 8.3, 0.9 Hz, 1H), 0.54 – 0.36 (m, *J* = 1.5 Hz, 9H).

^{19}F-NMR (282 MHz, CDCl$_3$): δ (ppm) = -73.04, -93.61.

^{13}C-NMR (101 MHz, CDCl$_3$): δ (ppm) = 167.4 (d, *J* = 245.6 Hz), 154.3 (d, *J* = 14.9 Hz), 132.1 (d, *J* = 10.3 Hz), 120.5 (d, *J* = 34.1 Hz), 118.7 (q, *J* = 320.5 Hz), 116.7 – 115.8 (m), 115.2 (d, *J* = 27.3 Hz), 0.5 (d, *J* = 3.6 Hz).

Data are in accordance with those reported in literature.[4]

3-(Trimethylsilyl)naphthalen-2-yl trifluoromethanesulfonate (**1d**)

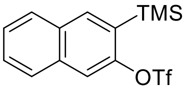

1d

According to a literature procedure,[5] 3-bromonaphthalen-2-ol (1.12 g, 5.00 mmol, 1.0 equiv.) and bis(trimethylsilyl)amine (1.6 mL, 7.5 mmol, 1.5 equiv.) were dissolved in dry THF (10 mL) and the mixture was refluxed at 80 °C for 18 h. Afterwards, the reaction mixture was concentrated *in vacuo* and the residue was dissolved in dry THF (12 mL). The mixture was cooled to -78 °C and at that temperature *n*BuLi (2.3 M in hexanes, 2.4 mL, 5.5 mmol, 1.1 equiv.) was added dropwise. After stirring for 30 min, triflic anhydride (1.3 mL, 7.5 mmol, 1.5 equiv.) was added slowly and the mixture was stirred at -78 °C for an additional hour. The solvent was removed under reduced pressure and after purification by column chromatography (SiO_2, hexane) the title compound was obtained as a yellow liquid (0.74 g, 2.1 mmol, 42%).

^{1}H-NMR (400 MHz, CDCl$_3$): δ (ppm) = 8.02 (s, 1H), 7.86 (ddd, *J* = 14.5, 6.9, 2.0 Hz, 2H), 7.82 (s, 1H), 7.60 – 7.52 (m, 2H), 0.45 (s, 9H).

^{19}F-NMR (282 MHz, CDCl$_3$): δ (ppm) = -73.73.

^{13}C-NMR (101 MHz, CDCl$_3$): δ (ppm) = 152.7, 137.7, 134.3, 131.9, 131.2, 128.1, 127.92, 127.90, 127.1, 118.7 (q, *J* = 320.1 Hz), 116.6 (q, *J* = 1.5 Hz), -0.58.

Data are in accordance with literature.[5]

4-Cyanobenzoyl chloride (**S1**)

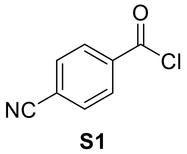

S1

According to a literature procedure,[6] 4-cyanobenzoic acid (11.8 g, 80.0 mmol, 1.0 equiv.) was suspended in dry dichloromethane (130 mL) and at 0 °C DMF (0.62 mL, 8.0 mmol, 0.10 equiv.) and oxalyl chloride (8.2 mL, 96 mmol, 1.2 equiv.) were added. The mixture was allowed to warm to room

temperature and stirred for 3 h. The solvent was removed under reduced pressure and the crude acid chloride was used in the next step without further purification.

4-Cyano-*N′*-phenylbenzohydrazide (**S2**)

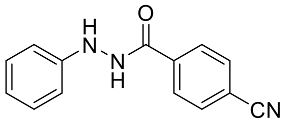

S2

According to a literature procedure,[3] phenylhydrazine (7.1 mL, 72 mmol, 1.0 equiv.) was dissolved in dry THF (75 mL) and the solution was cooled to 0 °C. At this temperature NEt_3 (12 mL, 86 mmol, 1.2 equiv.) and a suspension of the acid chloride **S1** in dry THF (25 mL) were added. The mixture was allowed to warm to room temperature and stirred at that temperature for 24 h. After that time, water (50 mL) and a sat. aq. $NaHCO_3$ solution (50 mL) were added and the mixture was extracted with Et_2O (3 x 100 mL). The combined organic phases were washed with sat. aq. $NaHCO_3$ solution (100 mL) and dried over $MgSO_4$. The solvent was removed under reduced pressure and after recrystallization from MeOH the title product was obtained as an orange solid (7.4 g, 31 mmol, 43%).

^{1}H-NMR (300 MHz, Acetone-d$_6$): δ (ppm) = 9.90 (s, 1H), 8.21 – 8.13 (m, 2H), 7.97 – 7.88 (m, 2H), 7.26 (s, 1H), 7.22 – 7.15 (m, 2H), 6.99 – 6.93 (m, 2H), 6.81 (td, *J* = 7.3, 1.1 Hz, 1H).

^{13}C-NMR (100 MHz, Acetone-d$_6$): δ (ppm) = 166.2, 150.3, 138.2, 133.4, 129.7, 129.2, 120.8, 118.8, 115.9, 114.1.

Data are in accordance with those reported in literature.[3]

N′-(2-Amino-5-methoxyphenyl)-4-cyano-N'-phenylbenzohydrazide (**S3**)

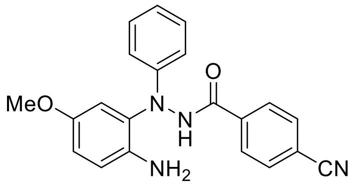

S3

According to a literature procedure,[3] 2-iodo-4-methoxyaniline (5.50 g, 22.1 mmol, 1.0 equiv.), hydrazide **S2** (5.24 g, 22.1 mmol, 1.0 equiv.), K_2CO_3 (3.36 g, 24.3 mmol, 1.1 equiv.) and CuI (0.252 mg, 1.33 mmol, 6.0 mol%) were suspended in dry DMSO (11 mL) and the mixture was stirred at 70 °C for 24 h. Afterwards, the reaction mixture was diluted with ethyl acetate (10 mL) and water (10 mL). The phases were separated and the organic phase was washed with brine (3 x 20 mL) and dried over $MgSO_4$. The solvent was removed under reduced pressure and after purification by column chromatography (SiO_2, acetone/dichloromethane = 3:97 – 25:75) the product was obtained as a brown solid (1.65 g, 4.60 mmol, 21%).

^{1}H-NMR (300 MHz, Acetone-d$_6$): δ (ppm) = 10.32 (s, 1H), 8.21 – 8.11 (m, 2H), 7.99 – 7.93 (m, 2H), 7.24 – 7.16 (m, 2H), 6.90 (d, *J* = 2.8 Hz, 1H), 6.87 – 6.71 (m, 5H), 4.82 (s, 2H), 3.65 (s, 3H).

^{13}C-NMR (101 MHz, Acetone-d$_6$): δ (ppm) = 166.6, 153.1, 148.5, 141.7, 137.9, 133.6, 131.4, 129.8, 129.4, 120.4, 118.8, 117.7, 116.3, 115.9, 114.5, 114.3, 56.1.

Data are in accordance with those reported in literature.[3]

<u>3-(4-Cyanophenyl)-7-methoxy-1-phenyl-1,4-dihydrobenzo[1,2,4]-triazin-4-yl radical (**2a**)</u>

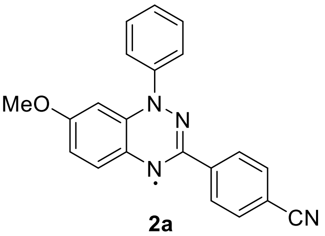

2a

According to a literature procedure,[3] the hydrazide **S3** (1.65 g, 4.60 mmol, 1.0 equiv.) was dissolved in conc. acetic acid (18 mL) and stirred at 130 °C for 4 h. After that time, the solution was diluted with ethyl acetate (20 mL) and sat. aq. NaOH solution (3 x 20 mL). The phases were separated, the organic phase was dried over $MgSO_4$ and the solvent was removed under reduced pressure. After purification by column chromatography (SiO_2, Et_2O/pentane = 50:50) and subsequent recrystallization from EtOH, the title compound was obtained as a brown solid (0.632 g, 1.86 mmol, 40%).

HRMS (ESI): m/z calculated for $[C_{21}H_{15}N_4O]^+$: 339.1240, found: 339.1240.

RP-HPLC:

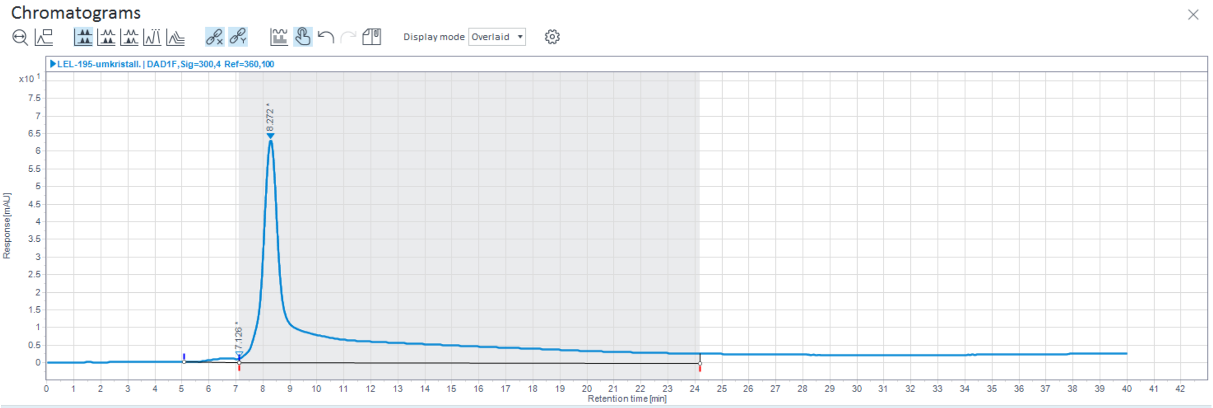

Injection Results

#	Signal description	RT (min)	Area	Area%	Height	Width
1	DAD1F,Sig=300,4 Ref=360,100	7.126	77.920	1.117	1.182	1.099
2	DAD1F,Sig=300,4 Ref=360,100	8.272	6895.480	98.883	63.209	1.407

<u>4-Nitro-*N,N*-diphenylaniline (**S4**)</u>

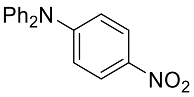

S4

According to a literature procedure,[7] a solution of diphenylamine (6.77 g, 40.0 mmol, 1.0 equiv.) in dry DMF (15 mL) was cooled to 0 °C. Then, a suspension of NaH (60% in paraffin oil, 2.40 g, 60.0 mmol, 1.5 equiv.) in DMF (15 mL) was added dropwise. The mixture was allowed to warm to room temperature and stirring was continued for 1 h. Afterwards, the mixture was cooled to 0 °C and 1-fluoro-4-nitrobenzene (5.1 mL, 48 mmol, 1.2 equiv.) was added dropwise. The mixture was heated to

100 °C and stirred at that temperature for 3 h. The reaction mixture was allowed to cool to room temperature and was slowly added to water at 0 °C under vigorous stirring. The formed precipitate was collected by filtration and washed with water. After recrystallization from MeOH, the title compound was obtained as an orange solid (7.6 g, 26 mmol, 66%)**^{1}H-NMR (399 MHz, $CDCl_3$):** δ (ppm) = 8.08 – 8.00 (m, 2H), 7.42 – 7.33 (m, 4H), 7.24 – 7.16 (m, 6H), 6.95 – 6.89 (m, 2H).

^{13}C-NMR (100 MHz, $CDCl_3$): δ (ppm) = 153.6, 145.8, 140.3, 130.1, 126.7, 125.9, 125.6, 118.2.

Data are in accordance with those reported in literature.[7]

N^1,N^1-Diphenylbenzene-1,4-diamine (**S5**)

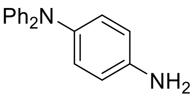

S5

According to a literature procedure,[7] a suspension of aniline **S4** (3.05 g, 10.5 mmol, 1.0 equiv.) and Pd/C (0.11 g, 1.1 mmol, 10 mol%) in EtOH (20 mL) was heated to reflux. The suspension was removed from the heat source and hydrazine hydrate (1.07 mL, 22.1 mmol, 2.1 equiv.) was added dropwise. Afterwards, the mixture was again heated to reflux for 2 h. The suspension was filtrated while it was still hot and the solvent was removed under reduced pressure. The title compound was obtained as an orange solid (2.7 g, 10.5 mmol, quant.) without further purification.

^{1}H-NMR (300 MHz, $CDCl_3$): δ (ppm) = 7.26 – 7.14 (m, 4H), 7.12 – 7.00 (m, 4H), 7.00 – 6.87 (m, 4H), 6.72 – 6.59 (m, 2H), 3.96 – 3.23 (m, 2H).

^{13}C-NMR (101 MHz, $CDCl_3$): δ (ppm) = 148.3, 141.4, 140.0, 129.2, 127.6, 122.9, 121.8, 117.0.

Data are in accordance with those reported in literature.[7]

(*Z*)-4-Cyano-*N*-phenylbenzohydrazonoyl chloride (**S6**)

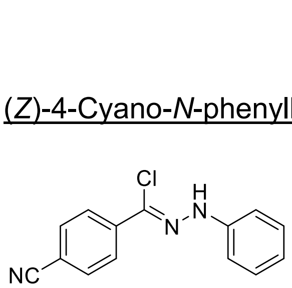

S6

According to a literature procedure,[8] 4-cyanobenzaldehyde (2.62 g, 20.0 mmol, 1.0 equiv.) and phenylhydrazine (2.0 mL, 20 mmol, 1.0 equiv.) were dissolved in dry EtOH (50 mL) and the mixture was stirred at room temperature for 3 h. Afterwards, the solvent was removed under reduced pressure to obtain crude (*E*)-4-((2-phenylhydrazine-ylidene)methyl)benzonitrile. *N*-Chlorosuccinimide (4.01 g, 30.0 mmol, 1.5 equiv.) was dissolved in dry dichloromethane (200 mL), cooled to 0 °C and stirred for 5 min. Then a solution of Me_2S (3.0 mL, 40 mmol, 2.0 equiv.) in dry dichloromethane (40 mL) was added slowly and after stirring for 15 min the reaction mixture was cooled to -40 °C. At that temperature a solution of crude (*E*)-4-((2-phenylhydrazineylidene)methyl)benzonitrile in dry

dichloromethane (60 mL) was added slowly to the reaction mixture and the mixture was stirred at -40 °C for 1.5 h. Then, the mixture was warmed to 0 °C and stirred at that temperature for further 3 h. The solvent was removed under reduced pressure and after purification by column chromatography (SiO_2, Et_2O/pentane = 20:80 -60:40) the title compound was obtained as an orange solid (1.8 g, 7.2 mmol,16%).

^{1}H-NMR (300 MHz, $CDCl_3$): δ (ppm) = 8.21 (s, 1H), 8.05 – 7.98 (m, 2H), 7.72 – 7.65 (m, 2H), 7.34 (dd, *J* = 8.6, 7.2 Hz, 2H), 7.25 – 7.17 (m, 2H), 7.00 (tt, *J* = 7.2, 1.2 Hz, 1H).

^{13}C-NMR (101 MHz, $CDCl_3$): δ (ppm) = 142.5, 138.4, 132.1, 129.4, 126.4, 122.4, 122.0, 118.5, 113.7, 112.0.

Data are in accordance with those reported in literature.[8]

(*Z*)-4-Cyano-*N''*-(4-(diphenylamino)phenyl)-*N'*-phenylbenzimidohydrazide (**S7**)

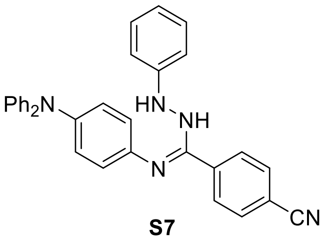

S7

Similar to a literature procedure,[9] diamine **S5** (0.74 g, 2.9 mmol, 1.00 equiv.) and hydrazonoyl chloride **S6** (0.91 g, 3.6 mmol, 1.25 equiv.) were dissolved in dry THF (30 mL). Then, NEt_3 (1.2 mL, 8.6 mmol, 3.0 equiv.) was added and the mixture was refluxed for 24 h. After that time the reaction mixture was allowed to cool to room temperature, filtered and the solvent of the filtrate was removed under reduced pressure. The crude product was used in the next step without further purification.

7-Diphenylamino-3-(4-cyanophenyl)-1-phenyl-1,4-dihydrobenzo[1,2,4]-triazin-4-yl radical (**2c**)

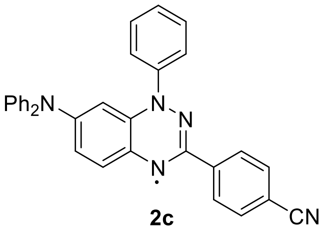

2c

Similar to a literature procedure,[9] imidohydrazide **S7** (from the previous step) was dissolved in dry dichloromethane (20 mL). Then, 1,8-diazabicyclo(5.4.0)undec-7-ene (0.85 mL, 5.7 mmol, 2.0 equiv.) and Pd/C (5 w%, 0.10 g, 5 mg/mL) were added and the suspension was stirred at room temperature for 24 h exposed to air. The mixture was filtered and the solvent of the filtrate was removed under reduced pressure. Purification by column chromatography (SiO_2, Et_2O/pentane =20:80 – 60:40) gave the title compound as a dark green solid (0.51 g, 1.1 mmol, 37%).

HRMS (ESI): m/z calculated for $[C_{32}H_{22}N_5]^+$: 476.1870, found: 476.1870.

RP-HPLC:

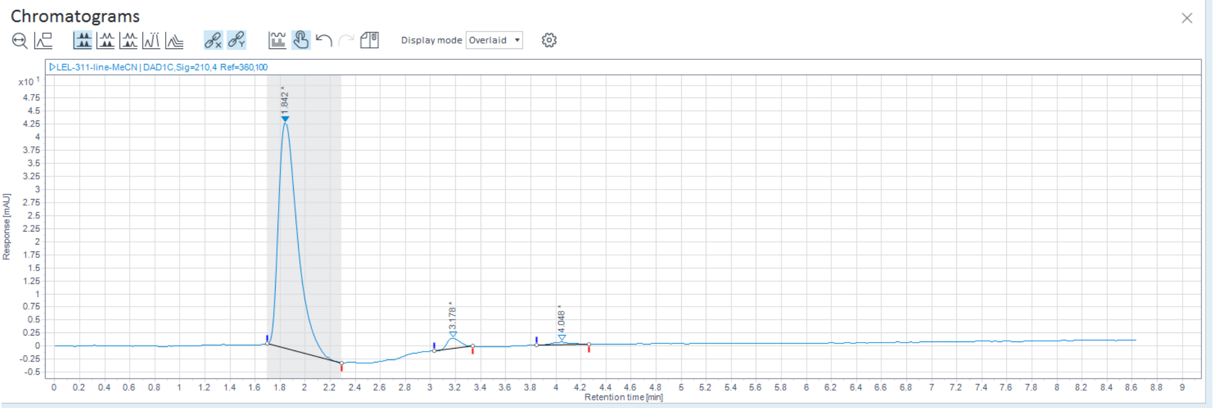

Injection Results

Peaks | Summary

#	Signal description	RT (min)	Area	Area%	Height	Width
1	DAD1C,Sig=210,4 Ref=360,100	1.842	453.204	95.992	43.254	0.158
2	DAD1C,Sig=210,4 Ref=360,100	3.178	13.782	2.919	1.963	0.110
3	DAD1C,Sig=210,4 Ref=360,100	4.048	5.140	1.089	0.495	0.139

Melting point: 223-229 °C.

IR (neat): $\tilde{\nu}$ (cm^{-1}) = 3040, 2227, 1584, 1486, 1412, 1388, 1354, 1315, 1272, 1244, 1174, 1124, 1077, 1017, 962, 895, 848, 792, 751, 692, 641, 628, 617, 599, 551, 526, 511, 499, 485.

2,6-Dimethyl-4-nitro-*N*,*N*-diphenylaniline (**S8**)

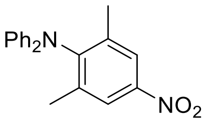

S8

Similar to a literature procedure,[7] a solution of diphenylamine (4.23 g, 25.0 mmol) in dry DMF (60 mL) was cooled to 0 °C. Then, Cs_2CO_3 (24.4 g, 75.0 mmol, 3.0 equiv.) was added in small portions. The mixture was allowed to warm to room temperature and stirred at room temperature for 1.5 h. 2-Fluoro-1,3-dimethyl-5-nitrobenzene (5.07 g, 30.0 mmol, 1.2 equiv.) was added portionwise and the mixture was stirred at 120 °C for 20 h. The mixture was allowed to cool to room temperature and diluted with water (100 mL). It was extracted with ethyl acetate (3 x 100 mL), the combined organic phases were dried over $MgSO_4$ and the solvent was removed under reduced pressure. After drying *in vacuo* and recrystallization from MeOH the title compound was obtained as a brown solid (3.81 g, 12.0 mmol, 48%).

^{1}H-NMR (300 MHz, $CDCl_3$): δ (ppm) = 8.03 (s, 2H), 7.31 – 7.21 (m, 4H), 7.06 – 6.89 (m, 6H), 2.10 (s, 6H).

^{13}C-NMR (101 MHz, $CDCl_3$): δ (ppm) = 149.2, 145.9, 145.4, 139.6, 129.6, 124.6, 122.3, 120.5, 19.4.

HRMS (ESI): m/z calculated for $[C_{20}H_{18}N_2O_2Na]^+$: 341.1260, found: 341.1260.

Melting point: 246-248 °C.

IR (neat): $\tilde{\nu}$ (cm^{-1}) = 3065, 1582, 1490, 1325, 1308, 1281, 1254, 1180, 1163, 1096, 1027, 955, 923, 894, 880, 825, 777, 747, 735, 690, 617, 569, 501.

2,6-Dimethyl-*N^1,N^1*-diphenylbenzene-1,4-diamine (**S9**)

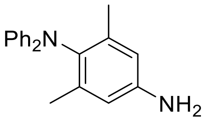

S9

Similar to a literature procedure,[7] a suspension of aniline **S8** (3.18 g, 10.0 mmol, 1.0 equiv.) and Pd/C (5 w%, 0.22 g, 2.0 mmol, 20 mol%) in EtOH (30 mL) was heated to reflux. The suspension was removed from the heat source and hydrazine hydrate (1.0 mL, 21 mmol, 2.1 equiv.) was added dropwise. Then, the mixture was again heated to reflux for 3 h. The mixture was filtered while it was still hot and the solvent was removed under reduced pressure. Purification by column chromatography (SiO_2, dichloromethane/pentane = 50:50 -70:30) gave the title compound as a beige solid (2.6 g, 9.0 mmol, 90%).

^{1}H-NMR (300 MHz, CDCl$_3$): 7.22 – 7.15 (m, 4H), 7.01 – 6.95 (m, 4H), 6.90 – 6.83 (m, 2H), 6.69 – 6.58 (m, 2H), 5.57 (br s, 2H), 1.96 (s, 6H).

^{13}C-NMR (101 MHz, CDCl$_3$): δ (ppm) = 146.5, 144.5, 139.2, 134.7, 129.1, 120.6, 119.5, 116.0, 18.8.

HRMS (ESI): m/z calculated for $[C_{20}H_{20}N_2H]^+$: 289.1699, found: 289.1703.

Melting point: 141-143 °C.

IR (neat): $\tilde{\nu}$ (cm^{-1}) = 3449, 3365, 3005, 2946, 1699, 1622, 1583, 1485, 1434, 1375, 1325, 1308, 1292, 1257, 1196, 1175, 1153, 1078, 1026, 994, 917, 886, 848, 752, 736, 691, 625, 563, 512, 498.

(*Z*)-4-Cyano-*N''*-(4-(diphenylamino)-3,5-dimethylphenyl)-*N'*-phenylbenzimidohydrazide (**S10**)

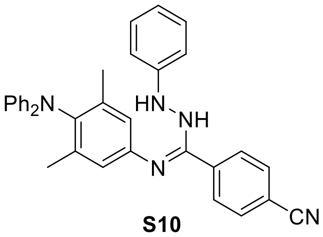

S10

Similar to a literature procedure,[9] hydrazonoyl chloride **S6** (0.22 g, 0.87 mmol, 1.25 equiv.) and diamine **S9** (0.20 g, 0.69 mmol, 1.0 equiv.) were dissolved in dry THF (8 mL). Then, NEt_3 (0.29 mL, 2.1 mmol, 3.0 equiv.) was added and the mixture was refluxed for 24 h. After that time the reaction mixture was cooled to room temperature, filtered and the solvent of the filtrate was removed under reduced pressure. The crude product was used in the next step without further purification.

7-Diphenylamino-3-(4-cyanophenyl)-6,8-dimethyl-1-phenyl-1,4-dihydrobenzo[1,2,4]-triazin-4-yl radical (**2d**)

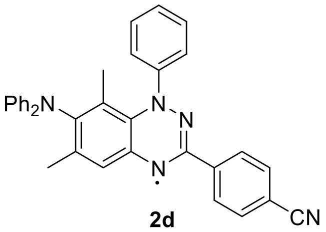

2d

Similar to a literature procedure,[9] imidohydrazide **S10** (from previous the step) was suspended in dichloromethane (5 mL). Then, 1,8-diazabicyclo(5.4.0)undec-7-ene (0.21 mL, 1.4 mmol, 2.0 equiv.) and Pd/C (5 w%, 25 mg, 5 mg/mL) were added and the suspension was stirred at room temperature for 24 h exposed to air. After that time the mixture was filtered and the solvent of the filtrate was removed under reduced pressure. Purification by column chromatography (SiO_2, Et_2O/pentane = 15:85 – 25:75) gave the title compound as a brown solid (0.17 g, 0.34 mmol, 49%). The radical was sufficiently pure to be used in the next step.

HRMS (ESI): m/z calculated for $[C_{34}H_{26}N_5]^+$: 504.2183, found: 504.2182.

RP-HPLC:

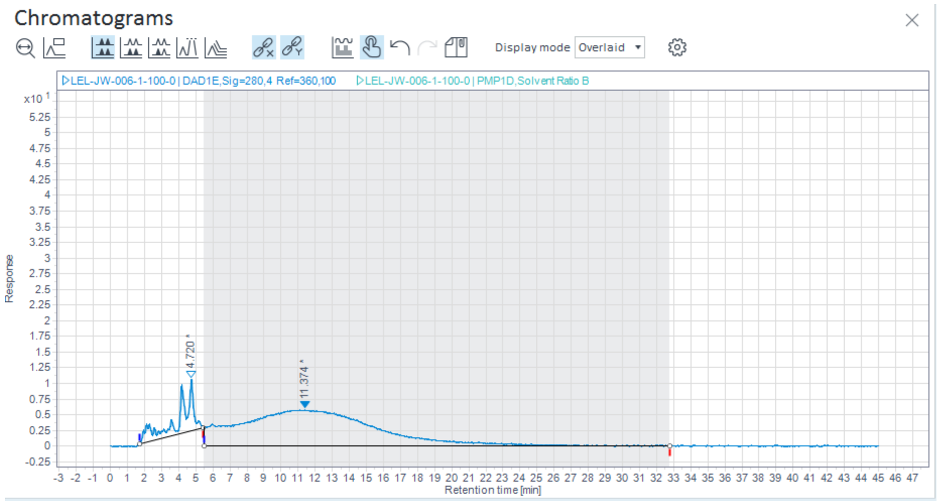

Injection Results

Peaks | Summary

#	Signal description	RT (min)	Area	Area%	Height	Width
1	DAD1E,Sig=280,4 Ref=360,100	4.720	449.629	12.204	8.233	0.677
2	DAD1E,Sig=280,4 Ref=360,100	11.374	3234.669	87.796	5.794	6.527

Melting point: 208-212 °C.

IR (neat): $\tilde{\nu}$ (cm^{-1}) = 2922, 2220, 1700, 1586, 1486, 1469, 1407, 1359, 1309, 1292, 1255, 1216, 1181, 1108, 1029, 957, 891, 853, 782, 768, 748, 692, 664, 637, 599, 566, 548, 516, 500.

Tert-butyl 2-(propan-2-ylidene)hydrazine-1-carboxylate (**S11**)

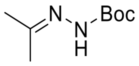

S11

According to a literature procedure,[10] *tert*-butyl carbazate (4.0 g, 30 mmol, 1.0 equiv.) was dissolved in dry acetone (30 mL). Then, $MgSO_4$ (0.73 g, 6.0 mmol, 20 mol%) was added to the solution and the mixture was refluxed for

3 h. Afterwards, the reaction mixture was cooled to room temperature, filtered and the solvent was removed under reduced pressure to give the product as a white solid (5.2 g, 30 mmol, quantitative).

^{1}H NMR (300 MHz, $CDCl_3$): δ(ppm) = 7.40 (s, 1H), 2.00 (s, 3H), 1.78 (s, 3H), 1.47 (s, 9H).

^{13}C NMR (76 MHz, $CDCl_3$): δ(ppm) = 153.0, 145.0, 81.0, 28.3, 25.5, 16.1.

Data are in accordance with those reported in the literature.[10]

Tert-butyl 2-isopropylhydrazine-1-carboxylate (**S12**)

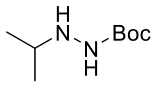

S12

According to a literature procedure,[10] hydrazine **S11** (5.2 g, 30 mmol, 1.0 equiv.) and Bromocresol green (1 spatula tip) were dissolved in dry THF (160 mL). Then, sodium cyanoborohydride (1.9 g, 30 mmol, 1.0 equiv.) was added resulting in a blue color of the reaction mixture. Afterwards, a solution of *p*-toluene sulfonic acid monohydrate (5.7 g, 30 mmol, 1.0 equiv.) in dry THF (12 mL) was added dropwise, while keeping the color of the reaction green. After complete addition, the solution was concentrated under reduced pressure and the residue was diluted with ethyl acetate (150 mL) and brine (150 mL) and the phases were separated. The organic phase was washed with sat. aq. $NaHCO_3$ solution (150 mL) and brine (150 mL), dried over $MgSO_4$ and concentrated under reduced pressure. Afterwards, the residue was dissolved in EtOH (120 mL) and aq. NaOH-solution (1 M, 8 mL) and stirred at room temperature for 1 h. After removal of EtOH, the residue was dissolved in ethyl acetate (150 mL) followed by water (150 mL) and the aqueous layer was extracted with ethyl acetate (2 x 150 mL). The combined organic phases were dried with $MgSO_4$ and concentrated under reduced pressure. The residue was purified by column chromatography (SiO_2, dichloromethane/EtOH = 95:5) and the title compound was obtained as a white solid (4.07 g, 23.4 mmol, 78%).

^{1}H NMR (400 MHz, $CDCl_3$): δ (ppm) = 5.99 (s, 1H), 4.45 – 3.53 (s, 1H), 3.13 (h, *J* = 6.5 Hz, 1H), 1.45 (s, 9H), 1.00 (d, *J* = 6.2 Hz, 6H).

^{13}C NMR (101 MHz, $CDCl_3$): δ (ppm) = 157.0, 80.5, 51.0, 28.5, 20.7.

Data are in accordance with those reported in the literature.[10]

Di-*tert*-butyl 2,2'-carbonylbis(2-isopropylhydrazine-1-carboxylate) (**S13**)

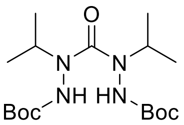

S13

According to a literature procedure,[11] hydrazine **S12** (3.6 g, 21 mmol, 2.0 equiv.) was dissolved in dry toluene (50 mL). Then, NEt_3 (2.9 mL, 21 mmol, 2.0 equiv.) was added and the solution was cooled to 0 °C. Afterwards, a solution of triphosgene (1.02 g, 3.44 mmol, 0.33 equiv.). in toluene (30 mL) was added dropwise over 1 h. The precipitated solid was removed by filtration and washed with toluene. The filtrate was concentrated under reduced pressure and purified by recrystallization from heptane. The product was obtained as a white, crystalline solid (2.5 g, 6.4 mmol, 62%).

^{1}H NMR (400 MHz, DMSO-d$_6$): δ (ppm) = 8.48 (s, 2H), 4.02 (hept, *J* = 6.5 Hz, 2H), 1.39 (s, 18H), 1.03 – 0.97 (m, 12H).

^{13}C NMR (101 MHz, DMSO-d$_6$): δ (ppm) = 161.0, 155.2, 78.8, 50.7, 28.0, 19.9, 19.1.

Data are in accordance with those reported in the literature.[11]

2,4-Diisopropylcarbohydrazide bis-hydrochloride (**S14**)

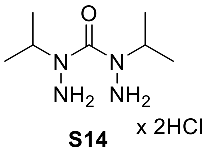

S14

According to a literature procedure,[11] bishydrazine **S13** (1.5 g, 4.0 mmol) was dissolved in dry EtOH (25 mL). After the addition of conc. hydrochloric acid (13 mL), the reaction mixture was refluxed for 30 min. Afterwards, the reaction mixture was cooled to room temperature and concentrated under reduced pressure. The crude product was purified by recrystallization from *n*-butanol and the product was obtained as a white solid (0.64 g, 2.6 mmol, 65%).

^{1}H NMR (300 MHz, DMSO-d$_6$): δ (ppm) = 7.79 (s, 6H), 4.24 (h, *J* = 6.6 Hz, 2H), 1.12 (d, *J* = 6.7 Hz, 12H).

^{13}C NMR (76 MHz, DMSO-d$_6$): δ (ppm) = 160.3, 50.9, 18.5.

Data are in accordance with those reported in the literature.[11]

2,4-Diisopropyl-6-phenyl-1,2,4,5-tetrazinan-3-one (**S15**)

Similar to a literature procedure,[11] carbohydrazide **S14** (0.25 g, 2.0 mmol, 1.0 equiv.) was dissolved in MeOH (2 mL). After the addition of benzaldehyde (0.10 mL, 1.0 mmol, 1.0 equiv.) and sodium acetate (0.16 g, 2.0 mmol, 2.0 equiv.), the reaction was stirred at room

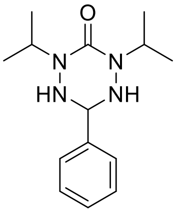

S15

temperature for 16 h. Afterwards, the formed solid was collected by filtration and washed with water and cold MeOH to give the tetrazinanone as a white solid (0.19 g, 0.72 mmol, 72%).

^{1}H NMR (400 MHz, DMSO-d$_6$): δ (ppm) = 7.59 – 7.51 (m, 2H), 7.44 – 7.31 (m, 3H), 4.96 (d, *J* = 11.6 Hz, 2H), 4.57 – 4.43 (m, *J* = 6.6, 6.1 Hz, 2H), 4.35 (t, *J* = 11.5 Hz, 1H), 1.04 (dd, *J* = 9.0, 6.6 Hz, 12H).

^{13}C NMR (101 MHz, DMSO-d$_6$): δ (ppm) = 153.4, 136.5, 128.3, 128.2, 126.7, 71.7, 46.7, 19.5, 18.4.

Data are in accordance with those reported in the literature.[12]

1,5-Diisopropyl-3-phenyl-6-oxo-verdazyl (**2h**)

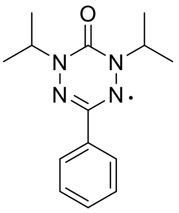

2h

Similar to a literature procedure,[11] the tetrazinanone **S15** (0.19 g, 0.72 mmol, 1.0 equiv.) was suspended in dichloromethane (10 mL). Then benzoquinone (0.13 g, 1.2 mmol, 1.7 eq) was added and the reaction was stirred at 60 °C for 4 h. The reaction mixture was allowed to cool to room temperature and the solvent was removed under reduced pressure. Purification by column chromatography (SiO_2, dichloromethane) delivered the product as an orange solid (0.16 g, 0.60 mmol, 82%).

EA (%) for $C_{14}H_{19}N_4O$: Calculated: C: 64.84, H: 7.38, N: 21.60; found: C: 64.79, H: 7.40, N: 21.48.

HRMS (ESI): m/z calculated for $[C_{14}H_{19}N_4ONa]^+$: 282.1451, found: 282.1451.

N,1-diphenylhydrazine-1-carbohydrazide (**S16**)

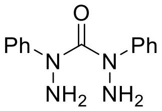

S16

According to a literature procedure,[2] K_3PO_4 (11.0 g, 52.0 mmol, 2.6 equiv.), CuI (0.19 g, 1.0 mmol, 5.0 mol%), 1,10-phenanthroline (0.36 g, 2.0 mmol, 10 mol%) and hydrazinecarbohydrazide (1.80 g, 20.0 mmol, 1.0 equiv.) were dissolved in dry DMF (40 mL) followed by addition of iodobenzene (5.2 mL, 46 mmol, 2.3 equiv.). The reaction mixture was stirred at 90 °C for 2 days. Afterwards, ethyl acetate (50 mL) was added and the mixture was filtered through silica. After addition of water (100 mL), the phases were separated and the aqueous layer was extracted with ethyl acetate (2 x 100 mL). The combined organic phases were washed with water (100 mL) followed by brine (100 mL) and dried over $MgSO_4$. After purification by column chromatography

(EtOAc/pentane = 85:15 – 90:10) the title compound was obtained as a yellow solid (1.5 g, 6.2 mmol, 31%).

^{1}H NMR (300 MHz, CDCl$_3$): δ (ppm) = 7.24 – 7.10 (m, 8H), 7.08 – 6.99 (m, 2H), 4.46 (s, 4H).

^{13}C NMR (76 MHz, CDCl$_3$): δ (ppm) = 162.0, 144.8, 128.9, 125.5, 123.8.

Data are in accordance with those reported in the literature.[2]

6-Isopropyl-2,4-diphenyl-1,2,4,5-tetrazinan-3-one (**S17**)

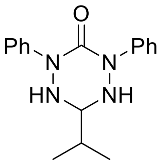

S17

According to a literature procedure,[13] carbohydrazide **S16** (0.85 g, 3.5 mmol, 1.0 equiv.) was added to a dried Schlenk tube under argon and dissolved in MeOH (25 mL). Afterwards, a solution of isobutyraldehyde (0.35 mL, 3.85 mmol, 1.1 equiv.) in MeOH (15 mL) was added over 10 min. After stirring for 3 h, the formed precipitate was filtered off and washed with cold MeOH. The title compound was obtained as a white solid without further purification (0.81 g, 2.7 mmol, 78%).

^{1}H NMR (300 MHz, CDCl$_3$): δ (ppm) = 7.67 – 7.54 (m, 4H), 7.33 (s, 4H), 7.10 (td, *J* = 7.2, 1.2 Hz, 2H), 4.36 (s, 2H), 4.01 (d, *J* = 7.0 Hz, 1H), 1.83 (h, *J* = 6.8 Hz, 1H), 1.11 (d, *J* = 6.7 Hz, 6H).

^{13}C NMR (76 MHz, CDCl$_3$): δ (ppm) = 157.0, 142.3, 128.5, 124.2, 121.1, 77.4, 30.5, 18.9.

Data are in accordance with those reported in the literature.[13]

3-Isopropyl-1,5-diphenyl-6-oxo-verdazyl (**2i**)

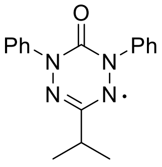

2i

According to a literature procedure,[13] tetrazinanone **S17** (0.42 g, 1.5 mmol, 1.0 equiv.) was dissolved in dichloromethane (25 mL). Then, benzoquinone (0.28 g, 2.6 mmol, 1.7 equiv.) was added and the resulting brown solution was stirred at 60 °C for 1 h. Afterwards, the mixture was stirred at 30 °C for further 3 h. After purification by column chromatography (SiO$_2$, DCM/pentane = 25:75 – 90:10) the title compound was obtained a red solid (0.22 g, 0.75 mmol, 50%).

EA (%) for $C_{17}H_{17}N_4O$: Calculated: C: 69.61, H: 5.84, N: 19.10; found: C: 69.17, H: 5.88, N: 19.07.

HRMS (ESI): m/z calculated for $[C_{17}H_{17}N_4ONa]^+$: 316.1295, found: 316.1293.

1,3,5-Triphenylverdazyl (**2k**)

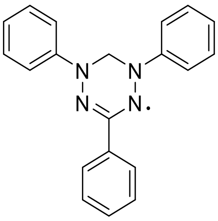

2k

According to a literature procedure,[14] 1,3,5-triphenylformazan (0.20 g, 0.65 mmol, 1.0 equiv.) was dissolved in DMF (11 mL). Then, aq. formaldehyde solution (37%, 2.2 mL, 10.4 mmol, 16 equiv.) and aq. NaOH solution (1 M, 3.1 mL, 3.1 mmol, 4.8 equiv.) were added and the reaction mixture was stirred at room temperature under air for 4 h in which the reaction mixture turned green. Afterwards, Et_2O (60 mL) was added and the organic phase was washed with water (3 x 60 mL) followed by brine (60 mL) and dried over $MgSO_4$. The solvent was removed under reduced pressure and after recrystallization from MeOH the title compound was obtained as a dark green solid (0.12 g, 0.37 mmol, 57%).

HRMS (ESI): m/z calculated for $[C_{20}H_{17}N_4]^+$: 313.1448, found: 313.1448.

RP-HPLC:

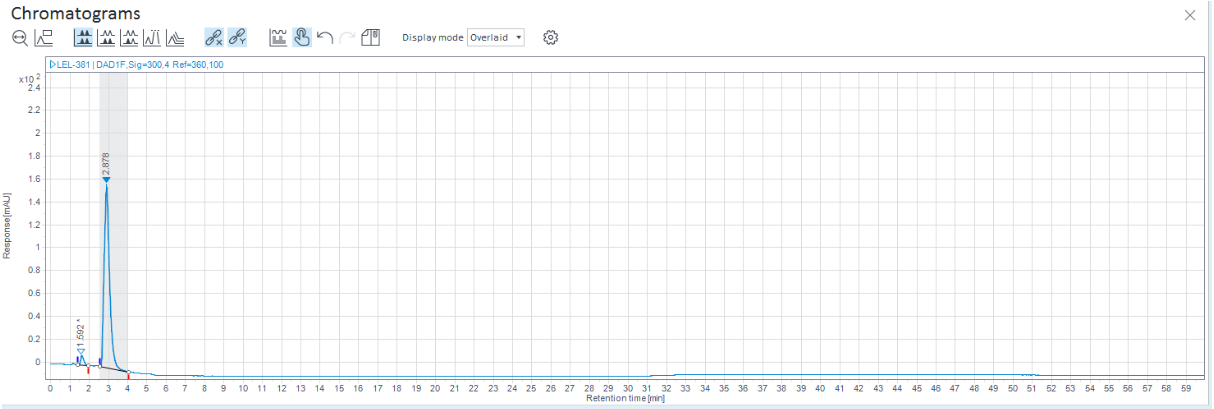

Injection Results

Peaks Summary

#	Signal description	RT (min)	Area	Area%	Height	Width
1	DAD1F,Sig=300,4 Ref=360,100	1.592	97.333	3.029	8.401	0.175
2	DAD1F,Sig=300,4 Ref=360,100	2.878	3116.514	96.971	160.561	1.498

4. Synthesis and Characterization of the Products

General procedure: The stable radical (50 µmol, 1.0 equiv.) and CsF (45.6 mg, 0.30 mmol, 6.0 equiv.) were dried *in vacuo* overnight. Afterwards, the aryne precursor (0.10 mmol, 2.0 equiv.) and dry acetonitrile (0.5 mL) were added and the reaction mixture was stirred at room temperature or 50 °C for 1.5 to 6 h. The solvent was removed under reduced pressure and the crude product was purified by column chromatography (SiO_2, Et_2O/pentane).

<u>8-Methoxy-6-phenyl-6H-benzo[5,6][1,2,4]triazino[4,3-f]phenanthridine-2-carbonitrile (**3aa**)</u>

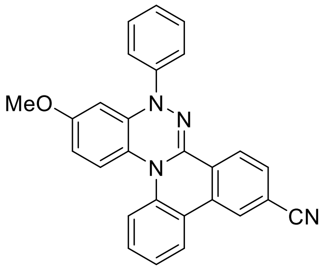

3aa

The reaction was performed according to the general procedure using aryne precursor **1a** (24 µL) and Blatter radical **2a** (17.0 mg). After purification by column chromatography (SiO_2, Et_2O/pentane = 10:90 – 15:85), the product **3aa** was obtained as a red solid (13.6 mg, 32.8 µmol, 66%) along with **3ab** (yellow solid, 5.2 mg, 13 µmol, 25%).

^{1}H-NMR (599 MHz, CD2Cl$_2$): 8.28 (dd, *J* = 8.2, 0.6 Hz, 1H), 8.25 (dt, *J* = 1.6, 0.5 Hz, 1H), 7.92 – 7.89 (m, 1H), 7.63 (dd, *J* = 8.4, 1.2 Hz, 1H), 7.61 – 7.58 (m, 3H), 7.46 – 7.43 (m, 2H), 7.38 (ddd, *J* = 8.4, 7.2, 1.4 Hz, 1H), 7.21 – 7.15 (m, 2H), 7.10 (d, *J* = 8.8 Hz, 1H), 6.47 (dd, *J* = 8.8, 2.7 Hz, 1H), 6.29 (d, *J* = 2.6 Hz, 1H), 3.65 (s, 3H).

^{13}C-NMR (151 MHz, CD_2Cl_2): δ (ppm) = 159.0, 143.8, 143.0, 136.6, 133.0, 131.0, 130.3, 130.2, 129.5, 127.1, 126.1, 124.8, 124.5, 123.7, 123.1, 122.2, 120.8, 118.9, 117.9, 116.9, 114.6, 107.8, 101.8, 55.9.

HRMS (ESI): m/z calculated for $[C_{27}H_{18}N_4ONa]^+$: 437.1373, found: 437.1378.

Melting point: 230-237 °C.

IR (neat): $\tilde{\nu}$ (cm^{-1}) = 2919, 2230, 1701, 1627, 1588, 1488, 1443, 1413, 1367, 1329, 1281, 1266, 1230, 1199, 1155, 1120, 1098, 1047, 1028, 905, 887, 848, 831, 791, 761, 742, 694, 654, 641, 614, 568, 531, 491, 476.

12-Methoxy-10-phenyl-10H-benzo[5,6][1,2,4]triazino[2,3-f]phenanthridine-3-carbonitrile (**3ab**)

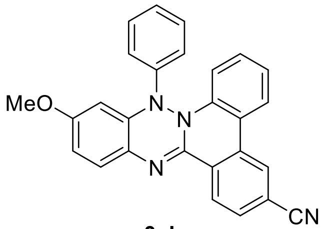

3ab

^{1}H-NMR (599 MHz, CD_2Cl_2): δ (ppm) = 8.56 (dd, *J* = 8.2, 0.6 Hz, 1H), 8.50 – 8.48 (m, 1H), 8.12 (dd, *J* = 8.0, 1.3 Hz, 1H), 7.98 (dd, *J* = 8.4, 1.2 Hz, 1H), 7.73 (dd, *J* = 8.2, 1.5 Hz, 1H), 7.52 (ddd, *J* = 8.5, 7.2, 1.3 Hz, 1H), 7.31 – 7.27 (m, 2H), 7.12 – 7.08 (m, 2H), 7.04 (d, *J* = 2.8 Hz, 1H), 6.97 – 6.92 (m, 2H), 6.82 – 6.79 (m, 2H), 3.90 (s, 3H).

^{13}C-NMR (151 MHz, CD_2Cl_2): δ (ppm) = 158.6, 149.9, 148.0, 140.1, 135.2, 134.9, 132.1, 131.6, 131.5, 130.7, 129.4, 127.3, 127.2, 126.2, 124.0, 123.8, 123.3, 119.2, 118.9, 117.0, 115.1, 114.3, 113.4, 111.1, 56.2.

HRMS (ESI): m/z calculated for $[C_{27}H_{18}N_4OH]^+$: 415.1553, found: 415.1553.

Melting point: 200-205 °C.

IR (neat): $\tilde{\nu}$ (cm^{-1}) = 2921, 2850, 2227, 1724, 1613, 1586, 1561, 1535, 1484, 1441, 1410, 1349, 1315, 1275, 1227, 1205, 1173, 1146, 1119, 1099, 1025, 964, 895, 860, 843, 825, 792, 771, 739, 689, 636, 621, 601, 567, 536, 516, 494.

6-Phenyl-6H-benzo[5,6][1,2,4]triazino[4,3-f]phenanthridine (**3ba**)

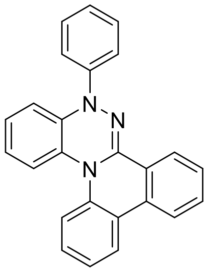

3ba

The reaction was performed according to the general procedure using aryne precursor **1a** (24 µL) and Blatter radical **2b** (14.2 mg). After purification by column chromatography (SiO_2, Et_2O/pentane = 5:95 – 15:85), the product **3ba** was obtained as an orange solid (9.0 mg, 25 µmol, 50%) along with **3bb** (green solid, 5.9 mg, 16 µmol, 33%).

^{1}H-NMR (599 MHz, CD_2Cl_2): δ (ppm) = 8.25 (ddd, *J* = 8.0, 1.4, 0.6 Hz, 1H), 8.04 – 7.99 (m, 2H), 7.67 (dd, *J* = 8.2, 1.2 Hz, 1H), 7.65 – 7.61 (m, 2H), 7.58 – 7.54 (m, 1H), 7.46 – 7.42 (m, 2H), 7.40 (ddd, *J* = 8.2, 7.2, 1.2 Hz, 1H), 7.34 (ddd, *J* = 8.5, 7.2, 1.4 Hz, 1H), 7.22 (dd, *J* = 7.8, 1.6 Hz, 1H), 7.19 – 7.14 (m, 2H), 6.97 – 6.91 (m, 2H), 6.79 – 6.76 (m, 1H).

^{13}C-NMR (151 MHz, CD_2Cl_2): δ (ppm) = 145.2, 144.3, 141.5, 136.1, 132.1, 131.7, 131.2, 129.4, 129.0, 128.6, 126.6, 126.1, 125.4, 124.4, 124.2, 123.6, 123.3, 122.8, 122.7, 121.8, 117.5, 117.4, 114.5.

HRMS (ESI): m/z calculated for $[C_{25}H_{17}N_3Na]^+$: 382.1315, found: 382.1313.

Melting point: 257-262 °C.

IR (neat): $\tilde{\nu}$ (cm^{-1}) = 3064, 2920, 2850, 1937, 1730, 1630, 1582, 1486, 1439, 1360, 1328, 1295, 1262, 1216, 1185, 1119, 1105, 1077, 1043, 1028, 982, 952, 900, 876, 803, 739, 721, 694, 666, 634, 610, 579, 563, 503.

10-Phenyl-10H-benzo[5,6][1,2,4]triazino[2,3-f]phenanthridine (**3bb**)

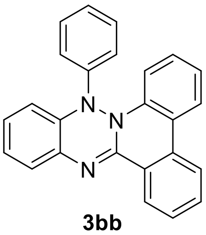

3bb

^{1}H-NMR (599 MHz, CD_2Cl_2): δ (ppm) = 8.56 (ddd, *J* = 8.0, 1.5, 0.6 Hz, 1H), 8.27 (ddt, *J* = 8.2, 1.1, 0.6 Hz, 1H), 8.24 – 8.21 (m, 1H), 8.01 (ddd, *J* = 8.3, 1.3, 0.5 Hz, 1H), 7.73 (ddd, *J* = 8.1, 7.2, 1.4 Hz, 1H), 7.57 (ddd, *J* = 8.1, 7.2, 1.1 Hz, 1H), 7.51 – 7.46 (m, 2H), 7.35 – 7.32 (m, 2H), 7.30 – 7.26 (m, 2H), 7.10 – 7.05 (m, 2H), 6.95 – 6.91 (m, 1H), 6.78 – 6.75 (m, 2H).

^{13}C-NMR (151 MHz, CD_2Cl_2): δ (ppm) = 151.2, 150.1, 142.5, 139.2, 134.4, 131.9, 131.7, 130.4, 129.3, 128.6, 128.5, 128.0, 126.5, 125.6, 125.1, 125.0, 123.64, 123.60, 123.2, 122.6, 120.8, 116.7, 113.5.

HRMS (ESI): m/z calculated for $[C_{25}H_{17}N_3Na]^+$: 382.1315, found: 382.1314.

Melting point: Decomposition above 165 °C.

IR (neat): $\tilde{\nu}$ (cm^{-1}) = 3388, 3186, 3070, 2920, 2850, 1645, 1588, 1563, 1523, 1488, 1470, 1452, 1435, 1350, 1277, 1198, 1155, 1119, 1031, 963, 939, 874, 838, 813, 749, 722, 691, 674, 615, 585, 468.

8-(Diphenylamino)-6-phenyl-6H-benzo[5,6][1,2,4]triazino[4,3-f]phenanthridine-2-carbonitrile (**3ca**)

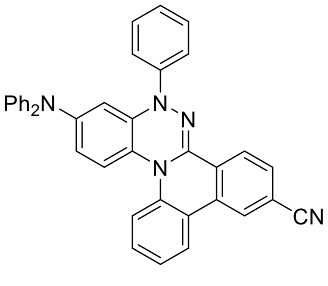

3ca

The reaction was performed according to the general procedure using aryne precursor **1a** (24 µL) and Blatter radical **2c** (23.8 mg). After purification by column chromatography (SiO_2, Et_2O/pentane = 5:95 – 10:90), the product **3ca** was obtained as a red solid (7.6 mg, 14 µmol, 28%) along with **3cb** (orange solid, 6.3 mg, 11 µmol, 23%).

^{1}H-NMR (599 MHz, CD_2Cl_2): δ (ppm) = 8.31 (d, *J* = 7.8 Hz, 1H), 8.26 (s, 1H), 7.92 (d, *J* = 8.0 Hz, 1H), 7.67 (d, J = 8.3 Hz, 1H), 7.61 (dt, *J* = 8.3, 1.6 Hz, 1H), 7.48 (d, *J* = 7.7 Hz, 2H), 7.39 (t, J = 7.8 Hz, 1H), 7.29 – 7.26 (m, 2H), 7.25 – 7.21 (m, 4H), 7.17 (t, *J* = 7.7 Hz, 1H), 7.06 – 6.98 (m, 8H), 6.59 (d, *J* = 8.6 Hz, 1H), 6.56 – 6.52 (m, 1H).

^{13}C-NMR (151 MHz, CD_2Cl_2): δ (ppm) = 147.7, 146.9, 144.1, 143.4, 142.0, 136.5, 133.0, 131.1, 130.3, 130.2, 129.6, 129.3, 127.2, 126.1, 125.8, 124.53, 124.46, 124.1, 123.4, 120.90, 120.86, 119.1, 118.9, 118.0, 117.2, 114.7, 110.6, 110.5.

HRMS (ESI): m/z calculated for $[C_{38}H_{25}N_5Na]^+$: 574.2002, found: 574.2000.

Melting point: 288-291 °C.

IR (neat): $\tilde{\nu}$ (cm^{-1}) = 2921, 2848, 2229, 1629, 1586, 1486, 1446, 1413, 1328, 1273, 1214, 1111, 1027, 963, 869, 834, 809, 771, 745, 692, 650, 631, 621, 564, 531, 515, 475.

<u>12-(Diphenylamino)-10-phenyl-10H-benzo[5,6][1,2,4]triazino[2,3-f]phenanthridine-3-carbonitrile (**3cb**)</u>

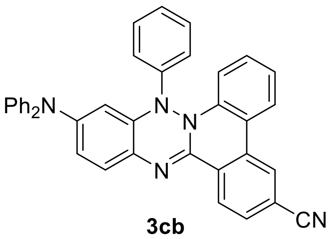

3cb

^{1}H-NMR (599 MHz, CD_2Cl_2): δ (ppm) = 8.58 (dd, *J* = 8.3, 0.6 Hz, 1H), 8.51 – 8.48 (m, 1H), 8.12 (dd, *J* = 8.1, 1.3 Hz, 1H), 7.86 – 7.83 (m, 1H), 7.74 (dd, *J* = 8.3, 1.5 Hz, 1H), 7.46 (ddd, *J* = 8.4, 7.2, 1.3 Hz, 1H), 7.34 – 7.30 (m, 4H), 7.27 (ddd, *J* = 8.4, 7.2, 1.3 Hz, 1H), 7.23 (d, *J* = 8.5 Hz, 1H), 7.19 – 7.16 (m, 5H), 7.12 – 7.05 (m, 5H), 6.98 – 6.95 (m, 1H), 6.76 – 6.73 (m, 2H).

^{13}C-NMR (151 MHz, CD_2Cl_2): δ (ppm) = 149.6, 148.4, 148.0, 146.4, 139.9, 136.7, 134.9, 132.1, 131.6, 131.5, 130.7, 129.8, 129.4, 127.32, 127.29, 126.0, 124.8, 123.88, 123.85, 123.75, 123.6, 123.4, 120.6, 119.2, 118.9, 116.7, 115.1, 113.5.

HRMS (ESI): m/z calculated for $[C_{38}H_{25}N_5Na]^+$: 574.2002, found: 574.2003.

Melting point: Decomposition above 148 °C.

IR (neat): $\tilde{\nu}$ (cm^{-1}) = 3056, 2923, 2852, 2228, 2161, 2028, 1978, 1589, 1561, 1525, 1489, 1442, 1412, 1353, 1324, 1277, 1227, 1161, 1029, 959, 894, 838, 746, 690, 635, 620, 588, 541, 516, 494.

<u>8-(Diphenylamino)-7,9-dimethyl-6-phenyl-6H-benzo[5,6][1,2,4]triazino[4,3-f]phenanthridine-2-carbonitrile (**3da**)</u>

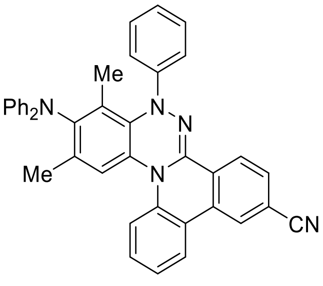

3da

The reaction was performed according to the general procedure using aryne precursor **1a** (24 µL) and Blatter radical **2d** (25.2 mg). After purification by column chromatography (SiO_2, Et_2O/pentane = 5:95 – 7:93), the product **3da** was obtained as a red solid (2.6 mg, 4.5 µmol, 9%) along with **3db** (yellow solid, 6.5 mg, 11 µmol, 22%).

^{1}H-NMR (599 MHz, CD_2Cl_2): δ (ppm) = 8.55 (dd, *J* = 8.2, 0.6 Hz, 1H), 8.34 (d, *J* = 1.5 Hz, 1H), 7.98 (dd, *J* = 8.0, 1.4 Hz, 1H), 7.80 (dd, *J* = 8.3, 1.1 Hz, 1H), 7.70 (dd, *J* = 8.2, 1.5 Hz, 1H), 7.43 (ddd, *J* = 8.4, 7.2, 1.4 Hz, 1H), 7.29 (t, *J* = 7.7 Hz, 2H), 7.26 – 7.23 (m, 1H), 7.21 – 7.15 (m, 5H), 7.09 – 7.04 (m, 4H), 6.98 (dd, *J* = 16.1, 7.8 Hz, 3H), 6.88 (t, *J* = 7.3 Hz, 1H), 6.80 (tt, *J* = 7.3, 1.2 Hz, 1H), 1.96 (s, 3H), 1.68 (s, 3H).

^{13}C-NMR (151 MHz, CD_2Cl_2): δ (ppm) = 152.1, 145.7, 142.2, 135.5, 135.4, 135.2, 132.6, 132.2, 131.7, 130.7, 129.8, 129.5, 129.1, 128.7, 126.7, 126.0, 124.1, 123.6, 121.2, 121.1, 120.4, 119.64, 119.58, 118.3, 118.0, 116.9, 114.9, 113.4, 18.6, 15.1.

HRMS (ESI): m/z calculated for $[C_{40}H_{29}N_5Na]^+$: 602.2315, found: 602.2315.

Melting point: 263-267 °C.

IR (neat): $\tilde{\nu}$ (cm^{-1}) = 2920, 2855, 2229, 1729, 1590, 1490, 1444, 1412, 1360, 1292, 1209, 1154, 1102, 1028, 985, 884, 835, 744, 692, 640, 606, 561, 538, 510.

4-(7-(Diphenylamino)-6,8-dimethyl-1,2-diphenyl-1,2-dihydrobenzo[e][1,2,4]triazin-3-yl)benzonitrile (**3db**)

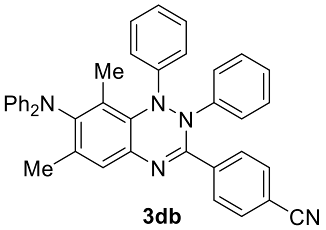

^{1}H-NMR (599 MHz, CD_2Cl_2): δ (ppm) = 8.16 – 8.13 (m, 2H), 7.72 – 7.67 (m, 2H), 7.41 (p, *J* = 0.6 Hz, 1H), 7.28 – 7.21 (m, 6H), 7.17 – 7.14 (m, 2H), 7.12 – 7.10 (m, 2H), 7.06 – 6.98 (m, 4H), 6.97 – 6.92 (m, 5H), 6.88 (tt, *J* = 7.4, 1.1 Hz, 1H), 2.08 (s, 3H), 1.86 (s, 3H).

^{13}C-NMR (151 MHz, CD_2Cl_2): δ (ppm) = 153.7, 148.5, 146.6, 146.1, 145.4, 142.6, 139.4, 137.6, 137.4, 133.1, 132.9, 132.1, 129.6, 129.5, 129.3, 126.0, 124.1, 123.6, 121.7, 121.6, 120.4, 120.1, 118.9, 118.8, 116.4, 114.7, 19.1, 13.6.

HRMS (ESI): m/z calculated for $[C_{40}H_{31}N_5Na]^+$: 604.2472, found: 604.2471.

Melting point: Decomposition above 150 °C.

IR (neat): $\tilde{\nu}$ (cm^{-1}) = 3032, 2922, 2855, 2228, 1588, 1535, 1489, 1410, 1291, 1174, 1150, 1081, 1028, 879, 849, 749, 690, 578, 512.

2-Bromo-6-phenyl-6H-benzo[5,6][1,2,4]triazino[4,3-f]phenanthridine (**3ea**)

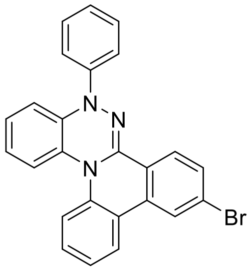

3ea

The reaction was performed according to the general procedure using aryne precursor **1a** (24 µL) and Blatter radical **2e** (18.2 mg). After purification by column chromatography (SiO_2, Et_2O/pentane = 5:95), the product **3ea** was obtained as an orange solid (10.3 mg, 23.5 µmol, 47%) along with **3eb** (green solid, 6.8 mg, 16 µmol, 31%).

^{1}H-NMR (599 MHz, CD_2Cl_2): δ (ppm) = 8.13 (d, *J* = 1.9 Hz, 1H), 8.10 (d, *J* = 8.5 Hz, 1H), 7.92 (dd, *J* = 8.0, 1.5 Hz, 1H), 7.66 (dd, *J* = 8.3, 1.2 Hz, 1H), 7.62 – 7.58 (m, 2H), 7.50 (dd, *J* = 8.5, 1.9 Hz, 1H), 7.46 – 7.41 (m, 2H), 7.36 (ddd, *J* = 8.4, 7.2, 1.4 Hz, 1H), 7.21 (dd, *J* = 7.7, 1.7 Hz, 1H), 7.19 – 7.16 (m, 2H), 6.97 – 6.91 (m, 2H), 6.76 – 6.72 (m, 1H).

^{13}C-NMR (151 MHz, CD_2Cl_2): δ (ppm) = 144.5, 144.1, 141.6, 136.5, 134.1, 131.6, 131.4, 129.8, 129.5, 127.1, 126.3, 125.73, 125.68, 125.6, 124.5, 124.4, 123.7, 123.4, 121.9, 121.6, 117.5, 117.4, 114.6.

HRMS (ESI): m/z calculated for $[C_{25}H_{16}BrN_3]^+$: 437.0522, found: 437.0521.

Melting point: 247-250 °C.

IR (neat): $\tilde{\nu}$ (cm^{-1}) = 3059, 2923, 2852, 2161, 2020, 1978, 1631, 1589, 1485, 1440, 1405, 1360, 1325, 1289, 1214, 1186, 1122, 1079, 1046, 1011, 984, 931, 903, 868, 827, 774, 740, 699, 665, 612, 601, 582, 551, 521, 473.

3-Bromo-10-phenyl-10H-benzo[5,6][1,2,4]triazino[2,3-f]phenanthridine (**3eb**)

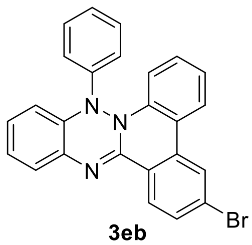

3eb

^{1}H-NMR (599 MHz, CD_2Cl_2): δ (ppm) = 8.43 (d, *J* = 8.6 Hz, 1H), 8.40 – 8.38 (m, 1H), 8.14 (dd, *J* = 8.0, 1.4 Hz, 1H), 8.01 – 7.99 (m, 1H), 7.67 (dd, *J* = 8.5, 1.9 Hz, 1H), 7.52 – 7.47 (m, 2H), 7.37 – 7.32 (m, 2H), 7.31 – 7.26 (m, 2H), 7.09 – 7.06 (m, 2H), 6.96 – 6.92 (m, 1H), 6.76 – 6.73 (m, 2H).

^{13}C-NMR (151 MHz, CD_2Cl_2): δ (ppm) = 150.6, 150.0, 142.2, 139.6, 134.3, 133.4, 131.7, 131.1, 129.4, 128.6, 128.4, 126.93, 126.89, 125.625, 125.617, 125.3, 125.1, 123.8, 123.7, 123.4, 119.6, 116.7, 113.6.

HRMS (ESI): m/z calculated for $[C_{25}H_{16}BrN_3H]^+$: 438.0600, found: 438.0603.

Melting point: Decomposition above 174 °C.

IR (neat): $\tilde{\nu}$ (cm^{-1}) = 3063, 2919, 2850, 2161, 2035, 1712, 1584, 1555, 1531, 1487, 1472, 1436, 1403, 1344, 1322, 1268, 1198, 1156, 1117, 1079, 1013, 958, 939, 889, 872, 842, 823, 761, 746, 690, 636, 619, 599, 579, 542, 522, 498, 469.

2-Methoxy-6-phenyl-6H-benzo[5,6][1,2,4]triazino[4,3-f]phenanthridine (**3fa**)

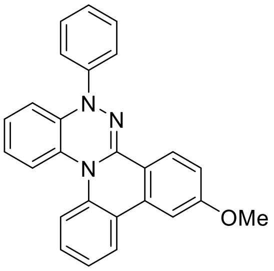

3fa

The reaction was performed according to the general procedure using aryne precursor **1a** (24 µL) and Blatter radical **2f** (15.7 mg). After purification by column chromatography (SiO_2, Et_2O/pentane = 20:80), the product **3fa** was obtained as an orange solid (6.6 mg, 17 µmol, 34%) along with **3fb** (green solid, 4.4 mg, 11 µmol, 23%).

^{1}H-NMR (500 MHz, CD_2Cl_2, 183 K): δ (ppm) = 8.14 (d, *J* = 8.8 Hz, 1H), 7.96 (d, *J* = 7.2 Hz, 1H), 7.63 (d, *J* = 8.0 Hz, 1H), 7.58 (d, *J* = 7.1 Hz, 2H), 7.45 – 7.36 (m, 3H), 7.37 – 7.29 (m, 1H), 7.18 (t, *J* = 8.6 Hz, 2H), 7.10 (s, 1H), 7.00 – 6.88 (m, 3H), 6.83 (d, *J* = 7.2 Hz, 1H), 3.89 (s, 3H).

^{13}C-NMR (126 MHz, CD_2Cl_2, 183 K): δ (ppm) = 160.8, 144.4, 142.6, 139.7, 135.0, 132.5, 130.7, 128.6, 128.5, 126.1, 125.1, 123.5, 122.83, 122.77, 122.3, 121.3, 120.0, 118.3, 116.8, 116.6, 114.9, 113.4, 105.0, 55.3.

HRMS (ESI): m/z calculated for $[C_{26}H_{19}N_3ONa]^+$: 412.1420, found: 412.1420.

Melting point: 264-268 °C.

IR (neat): $\tilde{\nu}$ (cm^{-1}) = 3059, 2919, 2849, 2161, 2036, 1730, 1631, 1607, 1583, 1486, 1446, 1361, 1328, 1296, 1265, 1211, 1174, 1120, 1106, 1040, 1017, 982, 889, 843, 817, 767, 738, 695, 639, 617, 606, 553, 527, 494.

3-Methoxy-10-phenyl-10H-benzo[5,6][1,2,4]triazino[2,3-f]phenanthridine (**3fb**)

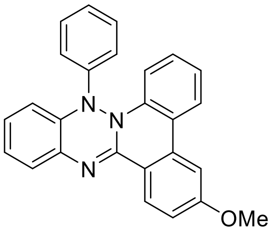

3fb

^{1}H-NMR (599 MHz, CD_2Cl_2): δ (ppm) = 8.50 (d, *J* = 8.8 Hz, 1H), 8.18 – 8.15 (m, 1H), 7.99 (dd, *J* = 8.3, 1.2 Hz, 1H), 7.66 (d, *J* = 2.4 Hz, 1H), 7.49 – 7.45 (m, 2H), 7.35 – 7.28 (m, 2H), 7.29 – 7.23 (m, 2H), 7.14 (dd, *J* = 8.9, 2.5 Hz, 1H), 7.09 – 7.05 (m, 2H), 6.95 – 6.90 (m, 1H), 6.77 – 6.73 (m, 2H), 4.00 (s, 3H).

^{13}C-NMR (151 MHz, CD_2Cl_2): δ (ppm) = 163.0, 151.1, 150.2, 142.8, 139.4, 134.4, 133.5, 130.5, 129.3, 128.6, 128.5, 125.5, 124.8, 124.5, 123.7, 123.6, 123.1, 121.4, 120.6, 116.7, 116.5, 113.6, 105.6, 56.1.

HRMS (ESI): m/z calculated for $[C_{26}H_{19}N_3ONa]^+$: 412.1420, found: 412.1420.

Melting point: 172-176 °C.

IR (neat): $\tilde{\nu}$ (cm^{-1}) = 3072, 2920, 2850, 2161, 2035, 1607, 1586, 1560, 1534, 1488, 1469, 1448, 1434, 1343, 1324, 1289, 1220, 1175, 1155, 1116, 1032, 1018, 960, 940, 889, 863, 828, 781, 765, 741, 690, 639, 619, 580, 541, 481, 467.

4-(7-Methyl-3,4-diphenylbenzo[e][1,2,4]triazin-1(4H)-yl)benzonitrile (**3ga**)

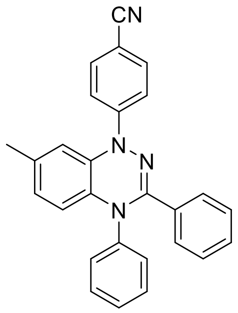

3ga

The reaction was performed according to the general procedure using aryne precursor **1a** (24 µL) and Blatter radical **2g** (16.2 mg). After purification by column chromatography (SiO_2, Et_2O/pentane = 10:90 – 20:80), the product **3ga** was obtained as a light brown solid (8.4 mg, 21 µmol, 42%) along with **3gb** (green solid, 7.2 mg, 18 µmol, 36%).

^{1}H-NMR (599 MHz, CD_2Cl_2): δ (ppm) = 8.10 – 8.06 (m, 2H), 7.57 – 7.53 (m, 2H), 7.49 – 7.45 (m, 2H), 7.45 – 7.41 (m, 2H), 7.41 – 7.37 (m, 2H), 7.19 – 7.14 (m, 4H), 7.03 – 6.97 (m, 3H), 2.38 (s, 3H).

^{13}C-NMR (151 MHz, CD_2Cl_2): δ (ppm) = 155.9, 153.0, 145.4, 138.0, 136.7, 134.8, 133.9, 132.0, 131.7, 129.6, 129.3, 128.8, 128.5, 125.9, 124.8, 124.3, 119.5, 119.0, 115.4, 105.6, 21.5.

HRMS (ESI): m/z calculated for $[C_{27}H_{20}N_4Na]^+$: 423.1580, found: 423.1579.

Melting point: Decomposition above 153 °C.

IR (neat): $\tilde{\nu}$ (cm^{-1}) = 3065, 2923, 2857, 2222, 2164, 2032, 1978, 1620, 1597, 1549, 1489, 1447, 1316, 1276, 1239, 1173, 1132, 1022, 930, 824, 789, 748, 688, 614, 541, 461.

4-(7-Methyl-2,3-diphenylbenzo[e][1,2,4]triazin-1(2H)-yl)benzonitrile (**3gb**)

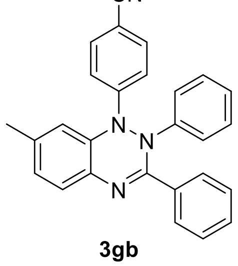

3gb

^{1}H-NMR (599 MHz, CD_2Cl_2): δ (ppm) = 7.46 – 7.44 (m, 2H), 7.36 – 7.33 (m, 1H), 7.24 – 7.17 (m, 8H), 7.11 (d, J = 8.0 Hz, 1H), 6.89 – 6.78 (m, 4H), 6.28 (s, 1H), 2.34 (s, 3H).

^{13}C-NMR (151 MHz, CD_2Cl_2): δ (ppm) = 169.3, 152.8, 148.9, 144.6, 139.5, 136.2, 133.5, 131.09, 131.07, 131.01, 130.2, 129.42, 129.35, 128.2, 122.0, 120.7, 120.3, 116.3, 112.8, 100.7, 21.1.

HRMS (ESI): m/z calculated for $[C_{27}H_{20}N_4Na]^+$: 423.1580, found: 423.1580.

Melting point: Decomposition above 196 °C.

IR (neat): $\tilde{\nu}$ (cm^{-1}) = 3277, 3056, 2922, 2855, 2214, 2164, 2033, 1981, 1600, 1497, 1311, 1289, 1266, 1222, 1170, 955, 825, 753, 709, 691, 645, 603, 578, 543, 474.

2,4-Diisopropyl-2H-[1,2,4,5]tetrazino[1,6-f]phenanthridin-3(4H)-one (**4aa**)

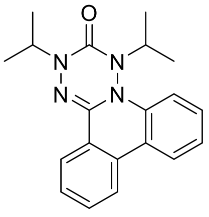

4aa

The reaction was performed according to the general procedure using aryne precursor **1a** (24 µL) and verdazyl radical **2h** (13.0 mg). After purification by column chromatography (SiO_2, Et_2O/pentane = 5:95 – 10:90) the product was obtained as a yellow solid (5.0 mg, 15 µmol, 30%).

^{1}H-NMR (599 MHz, CD_2Cl_2): δ (ppm) = 8.13 (dd, *J* = 7.9, 1.4 Hz, 1H), 8.07 (dd, *J* = 8.1, 1.0 Hz, 1H), 8.00 (dd, *J* = 7.9, 1.4 Hz, 1H), 7.62 (dd, *J* = 8.2, 1.2 Hz, 1H), 7.59 (ddd, *J* = 8.4, 7.2, 1.4 Hz, 1H), 7.46 (ddd, *J* = 8.1, 7.2, 1.1 Hz, 1H), 7.39 (ddd, *J* = 8.4, 7.2, 1.4 Hz, 1H), 7.13 (ddd, *J* = 8.2, 7.2, 1.2 Hz, 1H), 4.71 (h, *J* = 6.7 Hz, 1H), 3.58 (h, *J* = 6.7 Hz, 1H), 1.39 (d, *J* = 6.5 Hz, 3H), 1.33 (d, *J* = 6.8 Hz, 3H), 1.21 (d, *J* = 6.8 Hz, 3H), 1.12 (d, *J* = 6.6 Hz, 3H).

^{13}C-NMR (151 MHz, CD_2Cl_2): δ (ppm) = 155.1, 144.1, 138.2, 131.0, 130.9, 129.7, 128.4, 126.3, 124.7, 123.6, 122.7, 122.1, 120.8, 112.8, 59.3, 48.6, 21.9, 20.3, 20.2, 20.0.

HRMS (ESI): m/z calculated for $[C_{20}H_{22}N_4ONa]^+$: 357.1686, found: 357.1685.

Melting point: 167-172 °C.

IR (neat): $\tilde{\nu}$ (cm^{-1}) = 2971, 2932, 2164, 1672, 1626, 1603, 1488, 1442, 1367, 1303, 1216, 1154, 1129, 1105, 1070, 1043, 954, 880, 808, 762, 743, 722, 700, 648, 615, 595, 566.

4-Isopropyl-2-phenylbenzo[c][1,2,4,5]tetrazino[1,2-a]cinnolin-1(2H)-one (**4ba**)

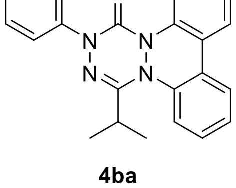

4ba

The reaction was performed according to the general procedure using aryne precursor **1a** (24 µL) and verdazyl radical **2i** (14.7 mg). After several purification attempts by column chromatography (SiO_2, Et_2O/pentane) and preparative HPLC the product could not be obtained cleanly. However, characteristic signals could be found in ^{1}H-NMR and HRMS suggesting the formation of at least traces of the desired product. The recorded analytics are shown below.

^{1}H-NMR (599 MHz, CD_2Cl_2):

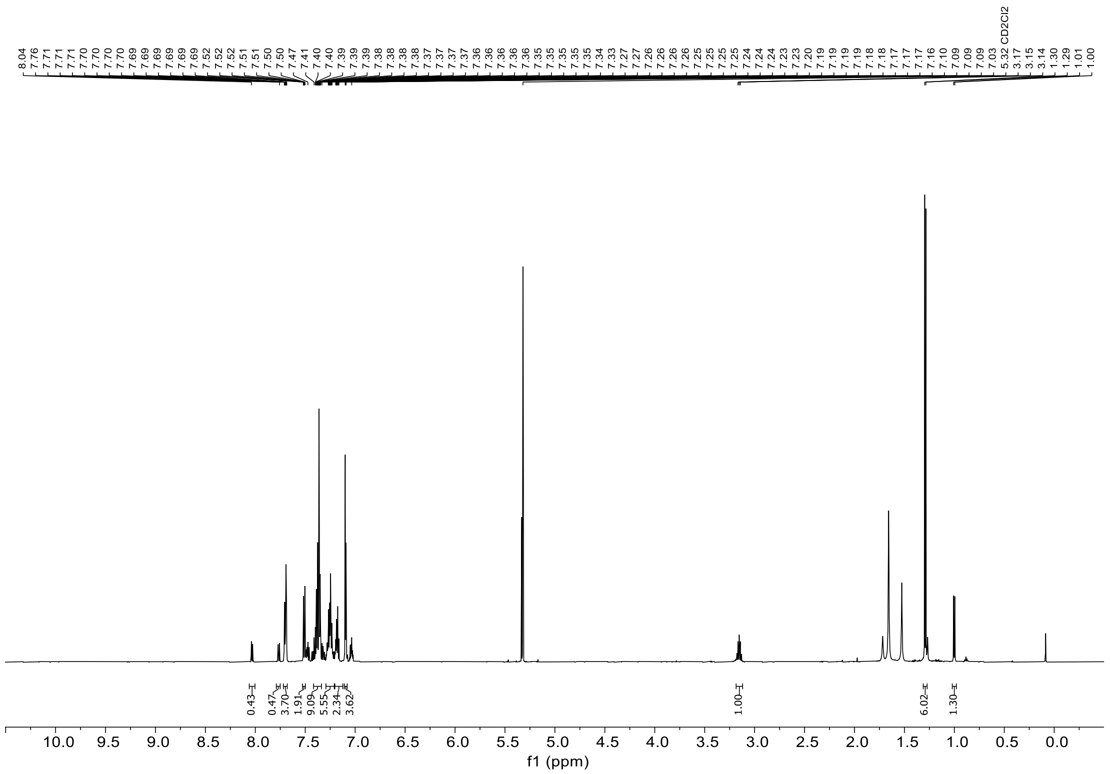

^{13}C-NMR (151 MHz, CD_2Cl_2):

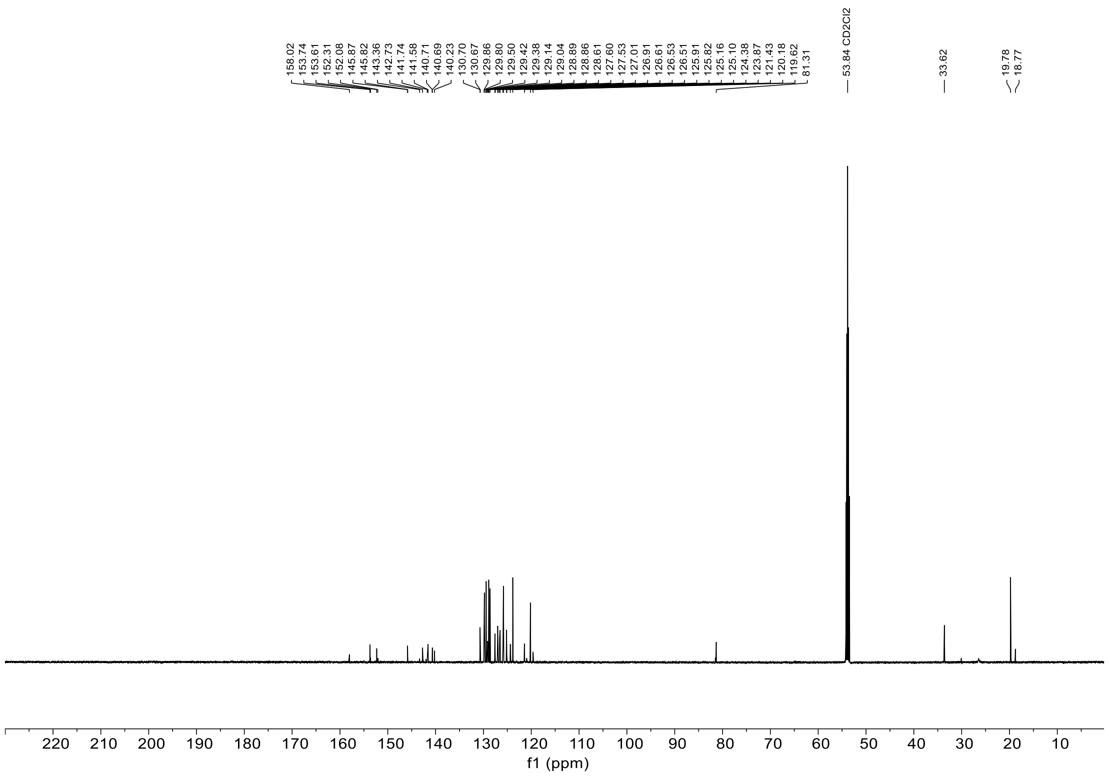

HRMS (ESI): m/z calculated for $[C_{23}H_{20}N_4ONa]^+$: 391.1529, found: 391.1532.

2,4-Diphenyl-2H-[1,2,4,5]tetrazino[1,6-f]phenanthridin-3(4H)-one (**4ca**)

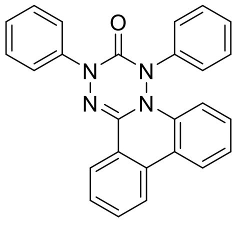

4ca

The reaction was performed according to the general procedure using aryne precursor **1a** (24 µL) and verdazyl radical **2j** (16.4 mg). After purification by column chromatography (SiO_2, Et_2O/pentane = 5:95 – 10:90), the product **4ca** was obtained as a light orange solid (7.3 mg, 18 µmol, 36%) along with **4cb** (orange solid, 5.4 mg, 13 µmol, 27%).

^{1}H-NMR (600 MHz, CD_2Cl_2): δ (ppm) = 8.29 (ddd, *J* = 7.9, 1.5, 0.6 Hz, 1H), 8.20 (ddd, *J* = 8.6, 1.1, 0.6 Hz, 1H), 8.11 (dd, *J* = 8.0, 1.4 Hz, 1H), 7.73 – 7.68 (m, 3H), 7.55 (ddd, *J* = 8.2, 7.2, 1.1 Hz, 1H), 7.45 – 7.40 (m, 3H), 7.32 (ddd, *J* = 8.4, 7.2, 1.4 Hz, 1H), 7.29 – 7.22 (m, 5H), 7.18 (ddd, *J* = 8.2, 7.2, 1.3 Hz, 1H), 7.13 – 7.10 (m, 1H).

^{13}C-NMR (151 MHz, CD_2Cl_2): δ (ppm) = 152.4, 145.3, 142.0, 141.4, 135.9, 131.8, 131.1, 130.1, 129.5, 128.90, 128.88, 126.5, 126.1, 125.1, 124.9, 124.2, 123.8, 123.12, 123.06, 121.0, 120.9, 113.1.

HRMS (ESI): m/z calculated for $[C_{26}H_{18}N_4ONa]^+$: 425.1373, found: 425.1373.

Melting point: 148-155 °C.

IR (neat): $\tilde{\nu}$ (cm^{-1}) = 3383, 3066, 2164, 1984, 1696, 1600, 1484, 1442, 1376, 1340, 1235, 1172, 1092, 1067, 1004, 825, 759, 742, 718, 690, 647, 617, 588, 559, 513.

1,2,4,6-Tetraphenyl-1,4-dihydro-1,2,4,5-tetrazin-3(2H)-one (**4cb**)

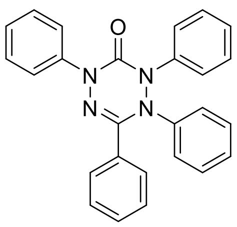

4cb

^{1}H-NMR (599 MHz, CD_2Cl_2): δ (ppm) = 8.09 – 8.06 (m, 2H), 7.84 – 7.80 (m, 2H), 7.58 – 7.55 (m, 2H), 7.54 – 7.48 (m, 3H), 7.44 – 7.39 (m, 4H), 7.30 – 7.26 (m, 1H), 7.26 – 7.23 (m, 2H), 7.20 (tt, *J* = 7.4, 1.1 Hz, 1H), 7.10 – 7.06 (m, 1H), 7.03 – 6.99 (m, 2H).

^{13}C-NMR (151 MHz, CD_2Cl_2): δ (ppm) = 153.2, 149.1, 145.9, 142.3, 140.8, 131.7, 131.5, 129.8, 129.6, 129.0, 127.6, 126.8, 125.1, 124.4, 124.3, 119.0, 116.7.

HRMS (ESI): m/z calculated for $[C_{26}H_{20}N_4ONa]^+$: 427.1529, found: 427.1531.

Melting point: 256-259 °C.

IR (neat): $\tilde{\nu}$ (cm^{-1}) = 3387, 3064, 2928, 2848, 2164, 1694, 1592, 1488, 1448, 1286, 1204, 1164, 1090, 1069, 1028, 902, 839, 750, 687, 610, 589, 567, 508.

8,15-Dimethoxy-6-phenyl-6H-benzo[5,6][1,2,4]triazino[4,3-f]phenanthridine-2-carbonitrile (**5aa**)

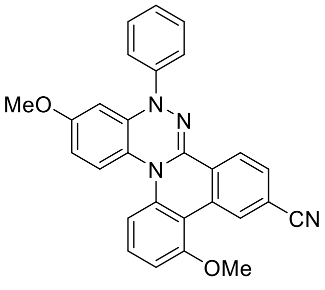

5aa

The reaction was performed according to the general procedure using aryne precursor **1b** (32.8 mg) and Blatter radical **2a** (17.0 mg). After purification by column chromatography (SiO_2, Et_2O/pentane = 15:85 – 20:80), the product **5aa** was obtained as a red solid (3.6 mg, 8.1 µmol, 16%) along with **5ab** (yellow solid, 4.5 mg, 10 µmol, 20%).

^{1}H-NMR (599 MHz, CD_2Cl_2): δ (ppm) = 9.15 (dd, *J* = 1.6, 0.6 Hz, 1H), 8.29 (d, *J* = 8.2 Hz, 1H), 7.58 (m, 3H), 7.44 (br t, *J* = 7.7 Hz, 2H), 7.32 (br d, *J* = 8.3 Hz, 1H), 7.30 – 7.27 (br m, 1H), 7.18 (br s, 1H), 7.06 (br d, *J* = 8.8 Hz, 1H), 6.78 – 6.72 (br m, 1H), 6.48 (br s, 1H), 6.29 (br s, 1H), 4.03 (s, 3H), 3.65 (s, 3H).

^{13}C-NMR (151 MHz, CD_2Cl_2): δ (ppm) = 137.0, 132.9, 130.4, 130.1, 129.5, 125.4, 122.1, 119.5, 113.9, 105.8, 101.7, 56.3, 55.9.

As the sample is dynamic not all ^{13}C-signals could be detected.

HRMS (ESI): m/z calculated for $[C_{28}H_{20}N_4O_2Na]^+$: 467.1478, found: 467.1478.

Melting point: 270-273 °C.

IR (neat): $\tilde{\nu}$ (cm^{-1}) = 2921, 2850, 2226, 2161, 1724, 1586, 1493, 1454, 1404, 1339, 1301, 1251, 1229, 1210, 1190, 1159, 1130, 1077, 1024, 1000, 954, 904, 832, 793, 758, 718, 695, 614, 594, 565, 523, 499.

5,12-Dimethoxy-10-phenyl-10H-benzo[5,6][1,2,4]triazino[2,3-f]phenanthridine-3-carbonitrile (**5ab**)

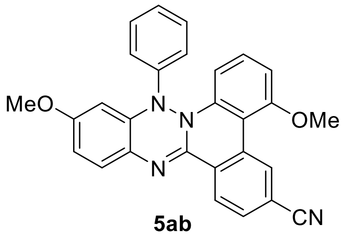

5ab

^{1}H-NMR (599 MHz, CD_2Cl_2): δ (ppm) = 9.43 (dd, *J* = 1.5, 0.6 Hz, 1H), 8.54 (dd, *J* = 8.2, 0.6 Hz, 1H), 7.70 (ddd, *J* = 8.2, 5.7, 1.3 Hz, 2H), 7.45 (t, *J* = 8.3 Hz, 1H), 7.30 (d, *J* = 8.7 Hz, 1H), 7.09 (dd, *J* = 9.0, 7.3 Hz, 2H), 7.02 (d, *J* = 2.8 Hz, 1H), 6.96 – 6.92 (m, 2H), 6.86 (d, *J* = 7.8 Hz, 1H), 6.78 (dt, *J* = 8.0, 1.0 Hz, 2H), 4.09 (s, 3H), 3.89 (s, 3H).

^{13}C-NMR (151 MHz, CD_2Cl_2): δ (ppm) = 158.4, 134.7, 132.8, 131.8, 131.6, 131.5, 131.4, 129.9, 129.4, 126.3, 126.1, 123.8, 119.5, 116.7, 114.5, 114.3, 112.7, 111.0, 106.4, 106.2, 105.7, 56.4, 56.2.

Due to the low amount of the compound not all ^{13}C-signals could be detected.

HRMS (ESI): m/z calculated for $[C_{28}H_{20}N_4O_2Na]^+$: 467.1478, found: 467.1479.

Melting point: 274-278 °C.

IR (neat): $\tilde{\nu}$ (cm^{-1}) = 3389, 3195, 2919, 2849, 2228, 1981, 1642, 1618, 1592, 1488, 1452, 1439, 1403, 1345, 1262, 1208, 1125, 1034, 788, 660, 466.

15-Fluoro-8-methoxy-6-phenyl-6H-benzo[5,6][1,2,4]triazino[4,3-f]phenanthridine-2-carbonitrile (**5ba**)

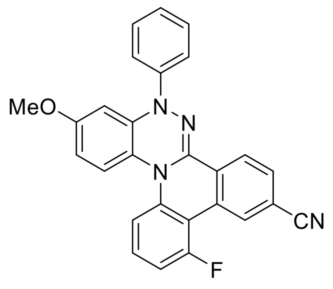

5ba

The reaction was performed according to the general procedure using aryne precursor **1c** (31.6 mg) and Blatter radical **2a** (17.0 mg). After purification by column chromatography (SiO_2, Et_2O/pentane = 5:95 – 20:80), the product **5ba** was obtained as a red solid (3.9 mg, 9.0 µmol, 18%) along with **5bb** (green solid, 3.4 mg, 7.9 µmol, 16%).

^{1}H-NMR (599 MHz, CD_2Cl_2): δ (ppm) = 8.64 (ddd, *J* = 2.9, 1.6, 0.6 Hz, 1H), 8.30 (dd, *J* = 8.2, 0.6 Hz, 1H), 7.64 (dd, *J* = 8.2, 1.5 Hz, 1H), 7.60 – 7.54 (m, 2H), 7.48 – 7.42 (m, 3H), 7.33 (td, *J* = 8.3, 5.8 Hz, 1H), 7.20 (tt, *J* = 7.4, 1.2 Hz, 1H), 7.06 (d, *J* = 8.8 Hz, 1H), 6.88 (ddd, *J* = 12.9, 8.2, 1.1 Hz, 1H), 6.49 (dd, *J* = 8.8, 2.7 Hz, 1H), 6.27 (d, *J* = 2.6 Hz, 1H), 3.65 (s, 3H).

^{13}C-NMR (151 MHz, CD_2Cl_2): δ (ppm) = 161.9 (d, *J* = 251.3 Hz), 159.2, 143.6, 143.4, 143.1, 138.6 (d, *J* = 6.1 Hz), 131.8, 131.6, 131.3 (d, *J* = 1.3 Hz), 130.8 (d, *J* = 5.6 Hz), 130.7, 130.3 (d, *J* = 4.0 Hz), 129.6, 125.8, 125.0, 123.1, 122.4, 119.0, 118.6, 114.7 (d, *J* = 1.8 Hz), 112.1 (d, *J* = 3.2 Hz), 110.3 (d, *J* = 24.4 Hz), 108.0, 101.8, 55.9.

^{19}F-NMR {^{13}C} (563 MHz, CD_2Cl_2): δ (ppm) = -111.48 (ddd, *J* = 12.9, 5.9, 2.9 Hz).

HRMS (ESI): m/z calculated for $[C_{27}H_{17}FN_4ONa]^+$: 455.1279, found: 455.1281.

Melting point: 272-276 °C.

IR (neat): $\tilde{\nu}$ (cm^{-1}) = 2921, 2848, 2228, 1730, 1587, 1493, 1450, 1339, 1272, 1210, 1159, 1122, 1053, 962, 913, 898, 837, 784, 758, 718, 692, 654, 613, 582, 523, 499.

5-Fluoro-12-methoxy-10-phenyl-10H-benzo[5,6][1,2,4]triazino[2,3-f]phenanthridine-3-carbonitrile (**5bb**)

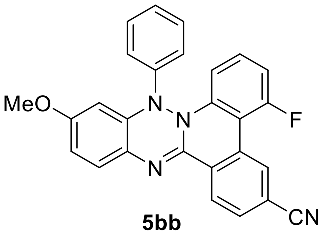

5bb

^{1}H-NMR (599 MHz, CD_2Cl_2): δ (ppm) = 8.88 (ddd, *J* = 3.9, 1.6, 0.6 Hz, 1H), 8.57 (dd, *J* = 8.2, 0.6 Hz, 1H), 7.84 (dt, *J* = 8.3, 0.9 Hz, 1H), 7.76 (dd, *J* = 8.3, 1.5 Hz, 1H), 7.46 (td, *J* = 8.3, 5.7 Hz, 1H), 7.32 (d, *J* = 8.7 Hz, 1H), 7.14 – 7.09 (m, 2H), 7.04 – 6.99 (m, 2H), 6.99 – 6.96 (m, 1H), 6.94 (dd, *J* = 8.7, 2.8 Hz, 1H), 6.82 – 6.78 (m, 2H), 3.90 (s, 3H).

^{13}C-NMR (151 MHz, CD_2Cl_2): δ (ppm) = 161.8 (d, *J* = 252.0 Hz), 158.8, 149.6, 147.6, 141.9 (d, *J* = 5.9 Hz), 134.8 (d, *J* = 13.6 Hz), 132.0, 131.8, 131.7, 131.6 (d, *J* = 8.2 Hz), 131.0 (d, *J* = 1.3 Hz), 129.5, 129.4 (d, *J* = 4.3 Hz), 126.9, 126.4, 124.2, 118.9, 117.0, 115.2 (d, *J* = 2.0 Hz), 114.3, 111.0, 110.8, 110.7, 109.1 (d, *J* = 2.9 Hz), 56.2.

^{19}F-NMR {^{13}C} (563 MHz, CD_2Cl_2): δ (ppm) = -111.17 (ddd, *J* = 13.6, 5.7, 3.9 Hz).

HRMS (ESI): m/z calculated for $[C_{27}H_{17}FN_4ONa]^+$: 455.1279, found: 455.1278.

Melting point: 271-275 °C.

IR (neat): $\tilde{\nu}$ (cm^{-1}) = 3376, 3184, 2920, 2850, 2229, 1978, 1647, 1617, 1589, 1488, 1447, 1403, 1341, 1311, 1279, 1257, 1224, 1201, 1177, 1146, 1096, 1031, 957, 884, 866, 792, 651.

3-Methoxy-5-phenyl-5H-benzo[b]benzo[5,6][1,2,4]triazino[4,3-f]phenanthridine-9-carbonitrile (**5ca**)

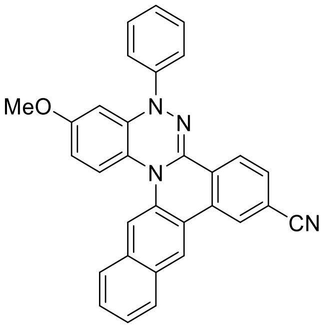

5ca

The reaction was performed according to the general procedure using aryne precursor **1d** (34.8 mg) and Blatter radical **2a** (17.0 mg). After purification by column chromatography (SiO_2, Et_2O/pentane = 5:95 – 25:75), the product **5ca** was obtained as a red solid (2.5 mg, 5.4 µmol, 11%) along with **5cb** (brown solid, 2.0 mg, 4.3 µmol, 9%).

^{1}H-NMR (599 MHz, CD_2Cl_2): δ (ppm) = 8.47 (d, *J* = 1.5 Hz, 1H), 8.42 (s, 1H), 8.34 (dd, *J* = 8.2, 0.6 Hz, 1H), 7.98 (s, 1H), 7.88 (d, *J* = 8.2 Hz, 1H), 7.72 (d, *J* = 8.4 Hz, 1H), 7.64 – 7.60 (m, 3H), 7.49 – 7.45 (m, 3H), 7.42 – 7.40 (m, 1H), 7.31 (d, *J* = 8.8 Hz, 1H), 7.21 (d, *J* = 7.3 Hz, 1H), 6.52 (dd, *J* = 8.8, 2.7 Hz, 1H), 6.32 (d, *J* = 2.7 Hz, 1H), 3.68 (s, 3H).

^{13}C-NMR (151 MHz, CD_2Cl_2): δ (ppm) = 158.8, 143.7, 143.1, 143.0, 134.5, 134.3, 132.8, 131.1, 130.6, 129.9, 129.6, 128.7, 128.1, 127.6, 127.0, 126.2, 125.5, 125.0, 124.4, 123.8, 122.4, 121.6, 118.9, 118.0, 114.6, 111.9, 107.9, 101.8, 56.0.

HRMS (ESI): m/z calculated for $[C_{31}H_{20}N_4ONa]^+$: 487.1529, found: 487.1532.

Melting point: 300-302 °C.

IR (neat): $\tilde{\nu}$ (cm^{-1}) = 3056, 2921, 2851, 2226, 2164, 1726, 1625, 1589, 1501, 1452, 1410, 1360, 1327, 1303, 1279, 1202, 1162, 1120, 1050, 983, 954, 884, 828, 795, 746, 697, 664, 632, 582, 476.

2-Methoxy-17-phenyl-17H-benzo[b]benzo[5,6][1,2,4]triazino[2,3-f]phenanthridine-8-carbonitrile (**5cb**)

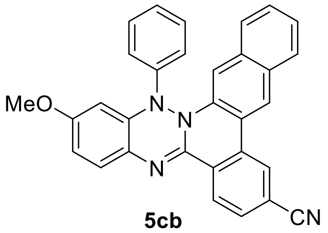

5cb

^{1}H-NMR (599 MHz, CD_2Cl_2): δ (ppm) = 8.67 (d, *J* = 1.5 Hz, 1H), 8.64 (s, 1H), 8.57 (dd, *J* = 8.2, 0.6 Hz, 1H), 8.31 (s, 1H), 7.98 (d, *J* = 8.2 Hz, 1H), 7.85 (d, *J* = 8.2 Hz, 1H), 7.76 (dd, *J* = 8.2, 1.5 Hz, 1H), 7.54 – 7.52 (m, 1H), 7.47 – 7.45 (m, 1H), 7.34 (d, *J* = 8.7 Hz, 1H), 7.14 (d, *J* = 2.8 Hz, 1H), 7.11 – 7.08 (m, 2H), 6.97 – 6.94 (m, 2H), 6.91 – 6.88 (m, 2H), 3.93 (s, 3H).

^{13}C-NMR (151 MHz, CD_2Cl_2): δ (ppm) = 158.8, 149.7, 147.8, 137.7, 135.2, 135.1, 134.9, 132.2, 132.0, 131.1, 129.9, 129.5, 128.8, 128.3, 127.7, 127.43, 127.37, 126.4, 125.6, 124.0, 123.9, 119.9, 118.9, 117.0, 115.2, 114.3, 111.2, 108.9, 56.3.

HRMS (ESI): m/z calculated for $[C_{31}H_{20}N_4ONa]^+$: 487.1529, found: 487.1529.

Melting point: 271-276 °C.

IR (neat): $\tilde{\nu}$ (cm^{-1}) = 3059, 2920, 2850, 2228, 1712, 1618, 1577, 1490, 1447, 1409, 1362, 1305, 1276, 1202, 1169, 1106, 1032, 963, 881, 864, 842, 814, 743, 688, 627, 604, 570, 514, 477.

5. X-Ray Analysis

X-ray crystal structure analysis of 3aa:

Red block single crystals of compound **3aa** were obtained by slow diffusion of methanol into its hexane solution. Intensity data were collected at 100 K on synchrotron radiation (λ = 0.4140 Å) at the BL02B1 beamline in SPring-8 (JASRI). A total of 46426 reflections were measured with the maximum 2θ angle of 31°, of which 9255 were independent reflections (R_{int} = 0.0711). The structure was solved by direct methods (SHELXT–2018/2)[15] and refined by full-matrix least squares procedures on F_2 for all reflections (SHELXL–2018/1).[16] All non-hydrogen atoms were refined anisotropically and all hydrogen atoms were placed using AFIX instructions. The crystal data are as follows: $C_{27}H_{18}N_4O$; FW = 828.91, triclinic, P–1, a = 11.3176(4) Å, b = 14.0090(5) Å, c = 14.7329(7) Å, α = 65.151(4), β = 72.404(4), γ = 79.946(3), V = 2017.39(16) Å^3, Z = 2, D_c = 1.365 g cm^{-3}. The refinement converged to R_1 = 0.0433 ($I > 2\sigma(I)$), wR_2 = 0.1130 (all data), and GOF = 1.012. Crystallographic data have been deposited at the Cambridge Crystallographic Data Centre with the deposition number CCDC 2452331. This data can be obtained free of charge from The Cambridge Crystallographic Data Centre at www.ccdc.cam.ac.uk/data_request/cif.

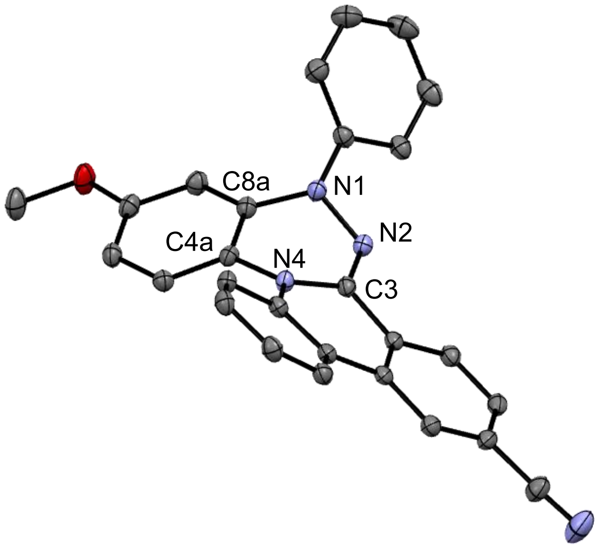

Figure S2. *ORTEP* diagram of **3aa** with the thermal ellipsoids at 50 % probability. Single crystals were obtained from a racemic mixture. Selected bond lengths are N1–N2 1.418(2) Å, N2–C3 1.290(2) Å, and C3–N4 1.401(1) Å, with the sum of the bond angles at N1 of 353.1˚.

X-ray crystal structure analysis of 3ab:

Yellow block single crystals of compound **3ab** were obtained by slow diffusion of hexane into its chloroform solution. Intensity data were collected at 100 K on synchrotron radiation (λ = 0.4140 Å) at the BL02B1 beamline in SPring-8 (JASRI). A total of 23006 reflections were measured with the maximum 2θ angle of 31°, of which 4587 were independent reflections (R_{int}

= 0.0642). The structure was solved by direct methods (SHELXT–2018/2)[15] and refined by full-matrix least squares procedures on F_2 for all reflections (SHELXL–2018/1).[16] All non-hydrogen atoms were refined anisotropically and all hydrogen atoms were placed using AFIX instructions. The crystal data are as follows: $C_{27}H_{18}N_4O$; FW = 414.45, triclinic, P–1,a = 8.2817(6) Å, b = 10.3713(8) Å, c = 12.5543(10) Å, α = 68.087(7), β = 87.668(6), γ = 87.377(6), V = 999.04(14) Å^3, Z = 2, D_c = 1.378 g cm^{-3}. The refinement converged to R_1 = 0.0516 (I > 2σ(I)), wR_2 = 0.1349 (all data), and GOF = 1.073. Crystallographic data have been deposited at the Cambridge Crystallographic Data Centre with the deposition number CCDC 2452332. This data can be obtained free of charge from The Cambridge Crystallographic Data Centre at www.ccdc.cam.ac.uk/data_request/cif.

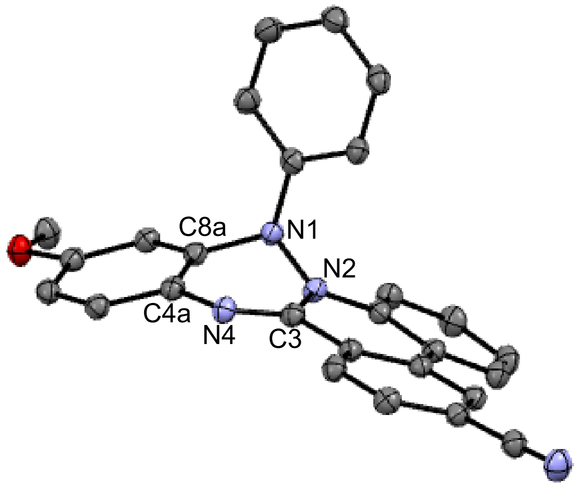

Figure S3. *ORTEP* diagram of **3ab** with the thermal ellipsoids at 50 % probability. Single crystals were obtained from a racemic mixture. Selected bond lengths are N1–N2 1.418(2) Å, N2–C3 1.387(2) Å, and C3–N4 1.305(2) Å, with the sum of the bond angles at N1 of 337.2˚.

General Information for X-ray analysis of 3ca, **3cb**, **3da**, **3db and 4ca:** X-Ray diffraction data for compounds **3ca**, **3cb**, **3da**, **3db** and **4ca** were collected with a Bruker D8 Venture Photon III Diffractometer. Programs used: data collection: *APEX6* Version 2024.9-0[17]; cell refinement: *SAINT* Version 8.41; data reduction: *SAINT* Version 8.41; absorption correction, *SADABS* Version 2016/2; structure solution *SHELXT*-Version 2018-3 [15], structure refinement *SHELXL*- Version 2019-2 [16] and graphics, *XP* [18]. *R*-values are given for observed reflections, and *w*R^2 values are given for all reflections.

Exceptions and special features: For compound **3cb** a badly disordered pentane molecule was found in the asymmetrical unit and could not be satisfactorily refined. The program SQUEEZE (Spek, A.L. (**2015**). Acta Cryst. C71, 9-18) was therefore used to remove mathematically the effect of the solvent. The quoted formula and derived parameters are not included the squeezed solvent molecule.

X-ray crystal structure analysis of 3ca (stu10595): An orange, prism-like specimen of $C_{38}H_{25}N_5$, approximate dimensions 0.072 mm x 0.129 mm x 0.191 mm, was used for the X-ray crystallographic analysis. The crystals were crystallised from DCM and pentane. The X-ray intensity data were measured on a single crystal diffractometer Bruker D8 Venture Photon III system equipped with a micro focus tube Cu ImS (CuKα, λ = 1.54178 Å) and a MX mirror monochromator. A total of 1323 frames were collected. The total exposure time was 15.43 hours. The frames were integrated with the Bruker SAINT software package using a wide-frame algorithm. The integration of the data using a triclinic unit cell yielded a total of 19179 reflections to a maximum θ angle of 66.57° (0.84 Å resolution), of which 4789 were independent (average redundancy 4.005, completeness = 98.0%, R_{int} = 3.35%, R_{sig} = 3.18%) and 4317 (90.14%) were greater than $2\sigma(F^2)$. The final cell constants of a = 9.2000(2) Å, b = 10.8477(3) Å, c = 14.9603(4) Å, α = 83.2120(10)°, β = 82.6630(10)°, γ = 70.0220(10)°, volume = 1387.23(6) Å^3, are based upon the refinement of the XYZ-centroids of 9914 reflections above 20 $\sigma(I)$ with 8.701° < 2θ < 133.0°. Data were corrected for absorption effects using the multi-scan method (SADABS). The ratio of minimum to maximum apparent transmission was 0.857. The calculated minimum and maximum transmission coefficients (based on crystal size) are 0.8910 and 0.9570. The structure was solved and refined using the Bruker SHELXTL Software Package, using the space group P–1, with Z = 2 for the formula unit, $C_{38}H_{25}N_5$. The final anisotropic full-matrix least-squares refinement on F^2 with 388 variables converged at R_1 = 4.00%, for the observed data and wR_2 = 10.88% for all data. The goodness-of-fit was 1.031. The largest peak in the final difference electron density synthesis was 0.307 e$^-$/Å^3 and the largest hole was −0.165 e$^-$/Å^3 with an RMS deviation of 0.037 e$^-$/Å^3. On the basis of the final model, the calculated density was 1.321 g/cm^3 and F(000), 576 e$^-$. CCDC number: 2451504.

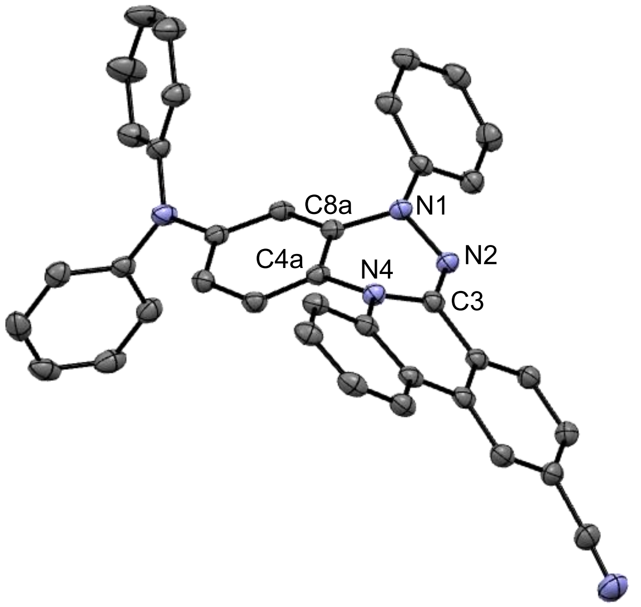

Figure S4. *ORTEP* diagram of **3ca** with the thermal ellipsoids at 50 % probability. Single crystals were obtained from a racemic mixture. Selected bond lengths are N1–N2 1.423 Å, N2–C3 1.284 Å, and C3–N4 1.401 Å, with the sum of the bond angles at N1 of 346.3°.

X-ray crystal structure analysis of 3cb (stu10625): A red, prism-like specimen of $C_{38}H_{25}N_5$, approximate dimensions 0.047 mm x 0.076 mm x 0.100 mm, was used for the X-ray crystallographic analysis. The crystals were crystallised from DCM and pentane. The X-ray intensity data were measured on a single crystal diffractometer Bruker D8 Venture Photon III system equipped with a micro focus tube Cu ImS (CuKα, λ = 1.54178 Å) and a MX mirror monochromator. A total of 1947 frames were collected. The total exposure time was 23.30 hours. The frames were integrated with the Bruker SAINT software package using a wide-frame algorithm. The integration of the data using a monoclinic unit cell yielded a total of 59301 reflections to a maximum θ angle of 66.67° (0.84 Å resolution), of which 5521 were independent (average redundancy 10.741, completeness = 99.7%, R_{int} = 7.14%, R_{sig} = 3.03%) and 4443 (80.47%) were greater than $2\sigma(F^2)$. The final cell constants of a = 8.1087(2) Å, b = 13.5789(3) Å, c = 28.4513(7) Å, β = 93.0860(10)°, volume = 3128.15(13) Å^3, are based upon the refinement of the XYZ-centroids of 9549 reflections above 20 $\sigma(I)$ with 6.222° < 2θ < 133.1°. Data were corrected for absorption effects using the multi-scan method (SADABS). The ratio of minimum to maximum apparent transmission was 0.905. The calculated minimum and maximum transmission coefficients (based on crystal size) are 0.9470 and 0.9750. The structure was solved and refined using the Bruker SHELXTL Software Package, using the space group $P2_1/n$, with Z = 4 for the formula unit, $C_{38}H_{25}N_5$. The final anisotropic full-matrix least-squares refinement on F^2 with 388 variables converged at R_1 = 3.51%, for the observed data and wR_2 = 9.08% for all data. The goodness-of-fit was 1.024. The largest peak in the final difference electron density synthesis was 0.172 e$^-$/Å^3 and the largest hole was -0.165 e$^-$/Å^3 with an RMS deviation of 0.034 e$^-$/Å^3. On the basis of the final model, the calculated density was 1.171 g/cm^3 and F(000), 1152 e$^-$. CCDC number: 2451505.

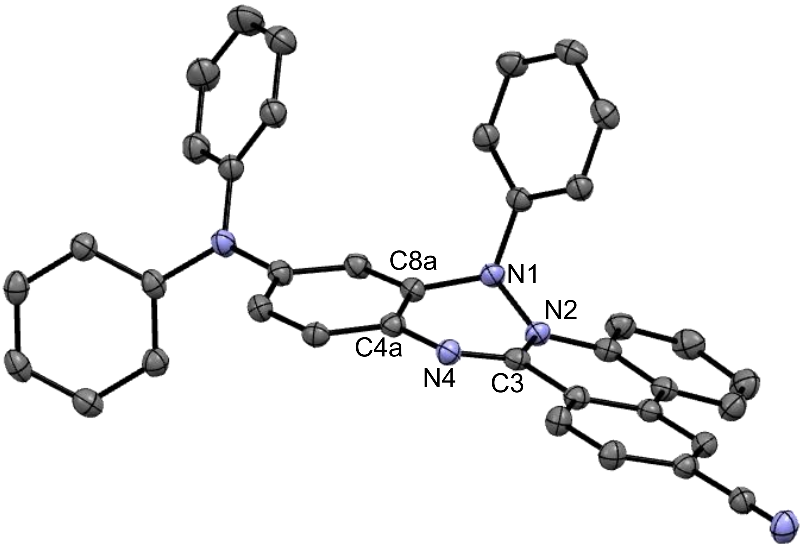

Figure S5. *ORTEP* diagram of **3cb** with the thermal ellipsoids at 50 % probability. Single crystals were obtained from a racemic mixture. Selected bond lengths are N1–N2 1.427 Å, N2–C3 1.384 Å, and C3–N4 1.296 Å, with the sum of the bond angles at N1 of 337.4˚.

X-ray crystal structure analysis of 3da (stu10703): A red, prism-like specimen of $C_{40}H_{29}N_5$, approximate dimensions 0.040 mm x 0.070 mm x 0.080 mm, was used for the X-ray crystallographic analysis. The crystals were crystallised from DCM and pentane. The X-ray intensity data were measured on a single crystal diffractometer Bruker D8 Venture Photon III system equipped with a micro focus tube Cu ImS (CuKα, λ = 1.54178 Å) and a MX mirror monochromator. A total of 1638 frames were collected. The total exposure time was 15.02 hours. The frames were integrated with the Bruker SAINT software package using a wide-frame algorithm. The integration of the data using a monoclinic unit cell yielded a total of 49984 reflections to a maximum θ angle of 66.76° (0.84 Å resolution), of which 5361 were independent (average redundancy 9.324, completeness = 99.6%, R_{int} = 15.70%, R_{sig} = 8.44%) and 3765 (70.23%) were greater than $2\sigma(F^2)$. The final cell constants of a = 29.562(2) Å, b = 8.0249(6) Å, c = 29.206(3) Å, β = 119.080(4)°, volume = 6055.2(9) Å^3, are based upon the refinement of the XYZ-centroids of 7328 reflections above 20 $\sigma(I)$ with 6.843° < 2θ < 132.4°. Data were corrected for absorption effects using the multi-scan method (SADABS). The ratio of minimum to maximum apparent transmission was 0.583. The calculated minimum and maximum transmission coefficients (based on crystal size) are 0.9540 and 0.9770. The structure was solved and refined using the Bruker SHELXTL Software Package, using the space group *C*2/*c*, with Z = 8 for the formula unit, $C_{40}H_{29}N_5$. The final anisotropic full-matrix least-squares refinement on F^2 with 408 variables converged at R_1 = 5.82%, for the observed data and wR_2 = 16.74% for all data. The goodness-of-fit was 1.010. The largest peak in the final difference electron density synthesis was 0.255 e$^-$/Å^3 and the

largest hole was −0.306 e⁻/Å^3 with an RMS deviation of 0.062 e⁻/Å^3. On the basis of the final model, the calculated density was 1.272 g/cm^3 and *F*(000), 2432 e⁻. CCDC number: 2451506.

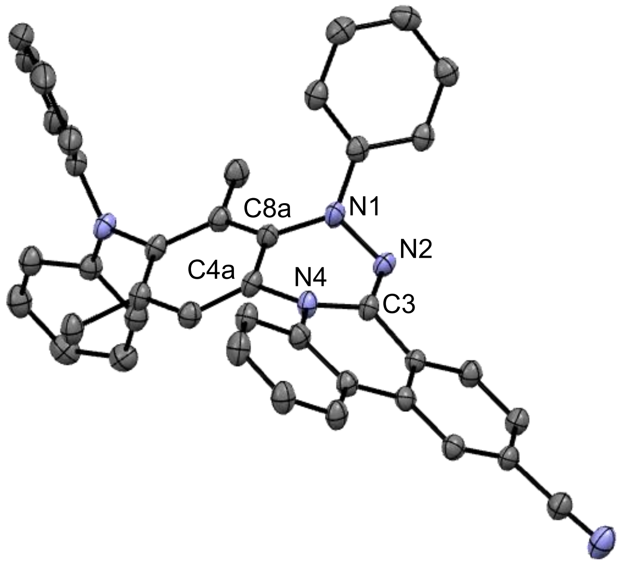

Figure S6. *ORTEP* diagram of **3da** with the thermal ellipsoids at 50 % probability. Single crystals were obtained from a racemic mixture. Selected bond lengths are N1–N2 1.417 Å, N2–C3 1.291 Å, and C3–N4 1.400 Å, with the sum of the bond angles at N1 of 360.0˚.

X-ray crystal structure analysis of 3db (stu10801): A yellow, prism shaped specimen of $C_{40}H_{31}N_5$, approximate dimensions 0.055 × 0.085 × 0.088 mm^3, was used for the X-ray crystallographic analysis. The crystals were crystallised from CH_2Cl_2 and pentane. The X-ray intensity data of stu10801 were measured on a Bruker D8 VENTURE KAPPA diffractometer system equipped with a microfocus sealed tube (λ = 0.71073 Å) and a multilayer mirror monochromator. A total of 1497 frames were collected. The total exposure time was 14.71 hours. The frames were integrated with the SAINT V8.41 package using a narrow-frame algorithm. The integration of the data using a triclinic unit cell yielded a total of 39332 reflections to a maximum θ angle of 26.80° (0.79 Å resolution), of which 6573 were independent (average redundancy 5.98, completeness = 100.0%, R_{int} = 7.13%, R_{sig} = 4.81%) and 4963 (75.5%) were greater than $2\sigma(F^2)$. The final cell constants of a = 9.9113(10) Å, b = 11.6082(12) Å, c = 14.8379(13) Å, volume = 1539.9(3) Å^3, are based upon the refinement of the XYZ-centroids of 8433 reflections above 20 σ(*I*) with 2.26° < 2θ < 26.72°. Data were corrected for absorption effects using the Multi-Scan method in SADABS 2016/2. The calculated minimum and maximum transmission coefficients (based on crystal size) are 0.993 and 0.996. The structure was solved by SHELXT 2018/2 and refined using the SHELXL-2019/2 Software, in the space group *P*–1 (2), with *Z* = 2 for the formula unit $C_{40}H_{31}N_5$. The final anisotropic full-matrix least-squares refinement on F^2 with 408 variables against 6573 data points converged at R_1 = 4.29%, for the observed data and wR_2 = 11.80% for all data. The

goodness-of-fit on F^2 was 1.03. The largest peak in the final difference electron density synthesis was 0.22 e⁻/Å^3 and the deepest hole was −0.25 e⁻/Å^3 with an RMS deviation of 0.045 e⁻/Å^3. On the basis of the final model, the calculated density was 1.25 g/cm^3 and F(000), 612 e⁻. CCDC number: 2451507.

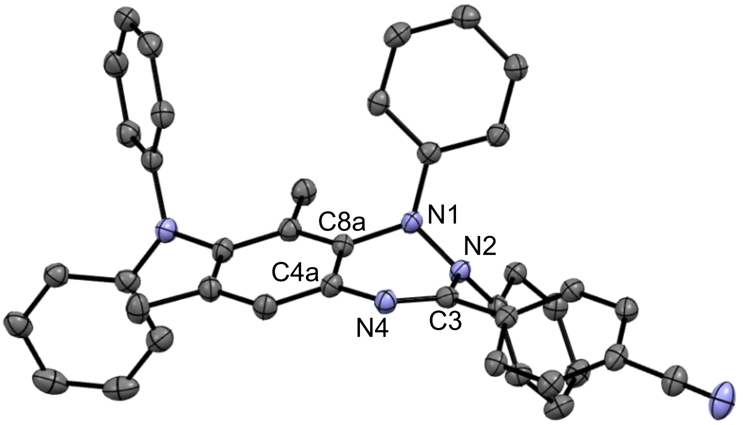

Figure S7. *ORTEP* diagram of **3db** with the thermal ellipsoids at 50 % probability. Single crystals were obtained from a racemic mixture. Selected bond lengths are N1–N2 1.418 Å, N2–C3 1.408 Å, and C3–N4 1.288 Å, with the sum of the bond angles at N1 of 344.0˚ and N2 of 355.3˚.

X-ray crystal structure analysis of 4ca (stu10576): A yellow, prism-like specimen of $C_{26}H_{18}N_4O$, approximate dimensions 0.069 mm x 0.133 mm x 0.214 mm, was used for the X-ray crystallographic analysis. The crystals were crystallised from DCM and pentane. The X-ray intensity data were measured on a single crystal diffractometer Bruker D8 Venture Photon III system equipped with a micro focus tube Cu ImS (CuKα, λ = 1.54178 Å) and a MX mirror monochromator. A total of 1948 frames were collected. The total exposure time was 19.51 hours. The frames were integrated with the Bruker SAINT software package using a wide-frame algorithm. The integration of the data using a monoclinic unit cell yielded a total of 68720 reflections to a maximum θ angle of 66.70° (0.84 Å resolution), of which 6849 were independent (average redundancy 10.034, completeness = 99.2%, R_{int} = 4.16%, R_{sig} = 1.98%) and 6086 (88.86%) were greater than 2σ(F^2). The final cell constants of a = 31.3055(5) Å, b = 10.6982(2) Å, c = 23.3792(4) Å, β = 96.1320(10)°, volume = 7785.2(2) Å^3, are based upon the refinement of the XYZ-centroids of 9933 reflections above 20 $\sigma(I)$ with 7.606° < 2θ < 133.2°. Data were corrected for absorption effects using the multi-scan method (SADABS). The ratio of minimum to maximum apparent transmission was 0.905. The calculated minimum and maximum transmission coefficients (based on crystal size) are 0.8670 and 0.9540. The structure was solved and refined using the Bruker SHELXTL Software Package, using the space group $C2/c$, with Z = 16 for the formula unit, $C_{26}H_{18}N_4O$. The final anisotropic full-matrix least-squares refinement on F^2 with 559 variables converged at R_1 = 3.16%, for the observed data and wR_2 = 8.56% for all data. The goodness-of-fit was 1.038. The largest peak in the final difference electron density synthesis was 0.197 e⁻/Å^3 and the largest hole was –0.187 e⁻/Å^3 with an RMS deviation

of 0.033 e⁻/Å^3. On the basis of the final model, the calculated density was 1.373 g/cm^3 and *F*(000), 3360 e⁻. CCDC number: 2451508.

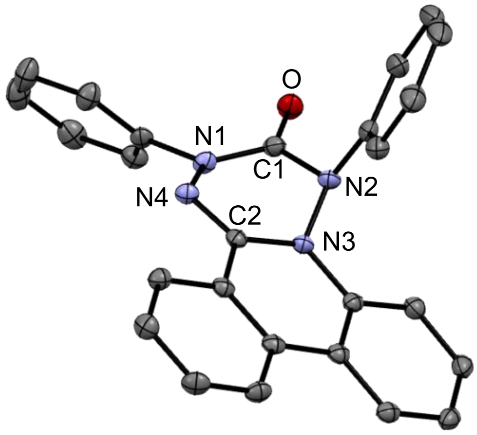

Figure S8. *ORTEP* diagram of **4ca** with the thermal ellipsoids at 50 % probability. Single crystals were obtained from a racemic mixture. Selected bond lengths are C1–O 1.361 Å, N1–C1 1.361 Å, C1–N2 1.430 Å, N2–N3 1.412 Å, N3–C2 1.384 Å, C2–N4 1.292 Å, and N4–N1 1.415 Å, with the sum of the bond angles at N1 of 359.2˚ and N2 of 341.7˚.

6. Photophysical Measurements

General Information: The spectroscopic measurements were conducted under ambient conditions using a 30 µM solution of the respective compound in spectroscopic grade CH_2Cl_2 or CH_3CN in quartz cuvettes with 1 cm path length. The UV/vis absorption spectra were recorded with a JASCO V-770 or a Shimadzu UV-3600 Plus spectrophotometer, the fluorescence spectra were recorded with a JASCO FP-8500 or a JASCO FP-8550 spectrofluorometer. The recorded spectra are shown below. Quantum yields were measured in solutions in spectroscopic grade CH_2Cl_2 with a Hamamatsu Absolute PL Quantum Yield spectrometer C11347-01 calibrated integrating sphere system (λ_{ex} = 300 nm).

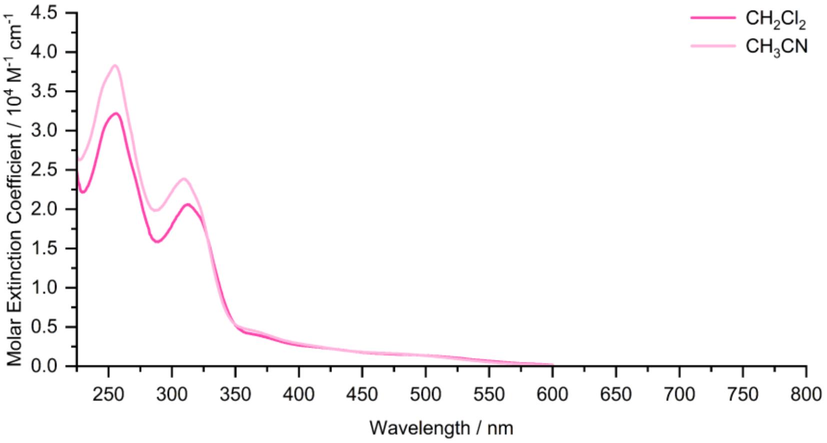

Figure S9. UV/vis absorption spectra of **3aa**.

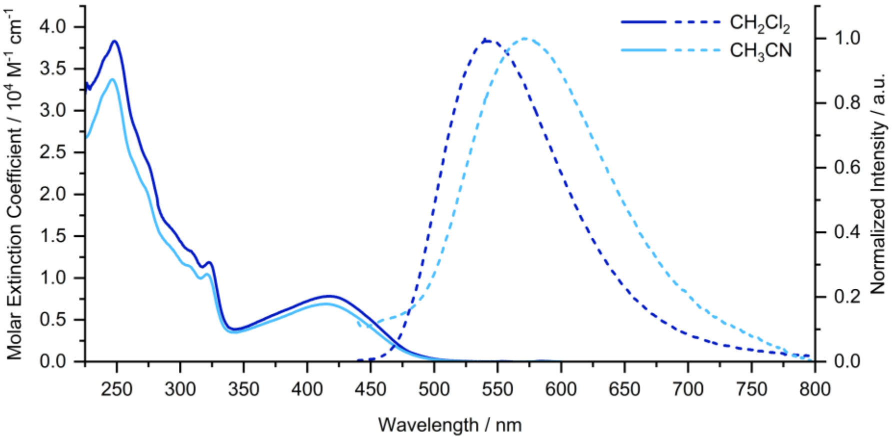

Figure S10. UV/vis absorption and fluorescence spectra of **3ab**.

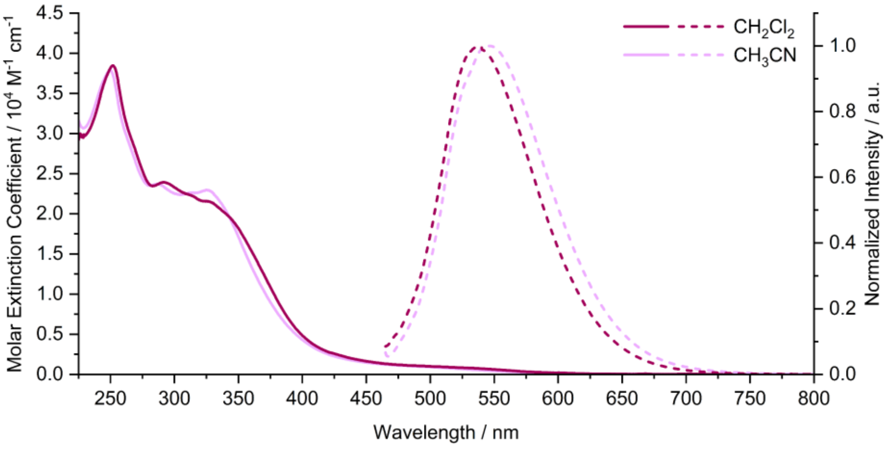

Figure S11. UV/vis absorption and fluorescence spectra of **3ca**.

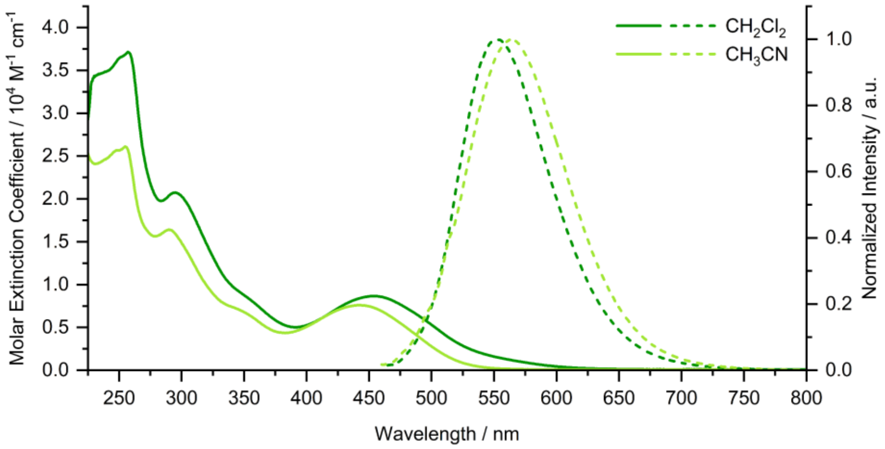

Figure S12. UV/vis absorption and fluorescence spectra of **3cb**.

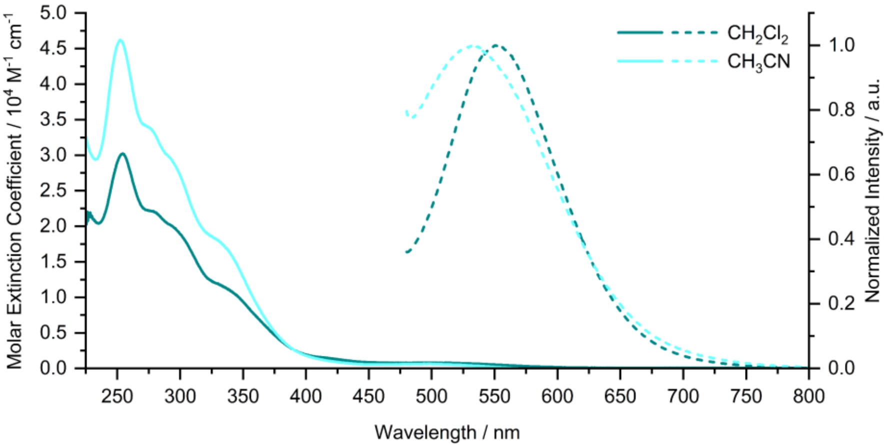

Figure S13. UV/vis absorption and fluorescence spectra of **3da**.

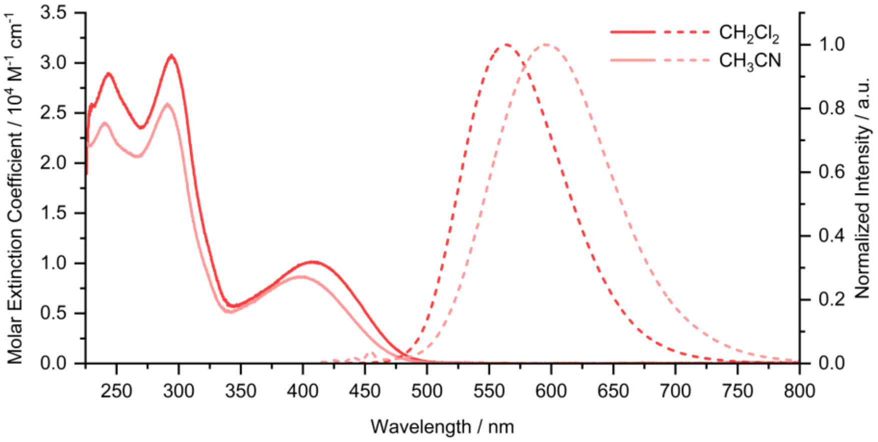

Figure S14. UV/vis absorption and fluorescence spectra of **3db**.

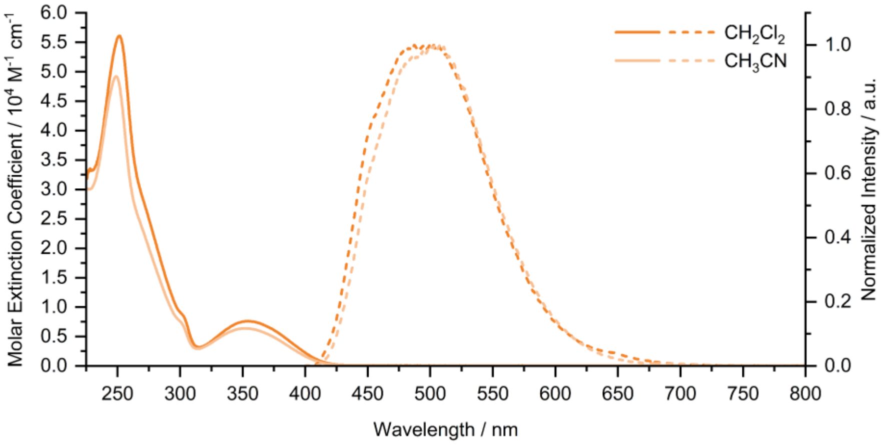

Figure S15. UV/vis absorption and fluorescence spectra of **4ca**.

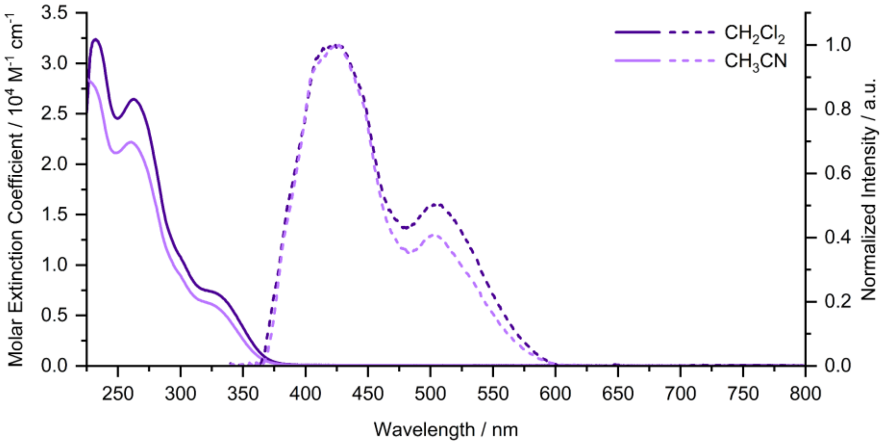

Figure S16. UV/vis absorption and fluorescence spectra of **4cb**.

Table S2. Photophysical properties of **3aa**, **3ab**, **3ca**, **3cb**, **3da**, **3db**, **4ca** and **4cb** in CH_2Cl_2.

Compound	λ_{abs} / nm	ε / $10^4 M^{-1} cm^{-1}$	λ_{em} / nm	Φ_F
**3aa**	500	0.15	n.d[a]	n.d.[a]
**3ab**	418	0.78	544	< 0.01
**3ca**	475	0.12	539	< 0.01
**3cb**	453	0.87	553	0.01
**3da**	496	0.08	552	< 0.01
**3db**	407	1.02	564	0.43
**4ca**	355	0.35	492	< 0.01
**4cb**	326	0.73	422	< 0.01

[a] Not detected.

7. Electrochemical Measurements

7.1 Cyclic Voltammetry

General Information: Cyclic voltammetry was conducted with a Metrohm Autolab using a 0.5 mM solution of the respective compound and 0.1 M Bu_4NPF_6 in CH_3CN in an argon filled glove box with a scan rate of 200 mV/s. A Pt wire working electrode, a Pt crucible counter electrode and a Ag wire reference electrode were used in a three-electrode-setup. Acetonitrile was degassed with three freeze-pump-thaw cycles and stored in a glove box over a molecular sieve (3 Å) and the supporting electrolyte NBu_4PF_6 (Sigma Aldrich, electrochemical grade) was stored in a glove box before use. All voltammograms were referenced to Fc/Fc^+ using $Me_{10}Fc$ as an internal standard with a conversion factor of -0.499 for $Me_{10}Fc/Me_{10}Fc^+$ vs Fc/Fc^+. The recorded voltammograms are shown below. Sweep directions are indicated with arrows.

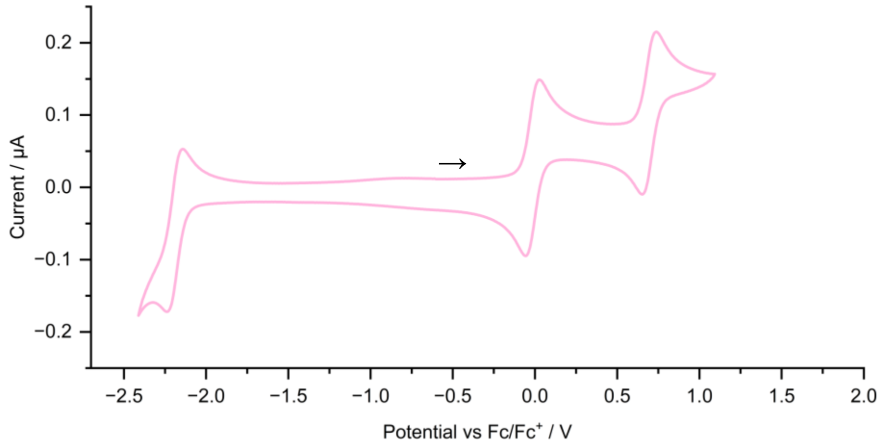

Figure S17. Cyclic voltammogram of **3aa** (0.5 mM in 0.1 M Bu_4NPF_6/CH_3CN), second scan.

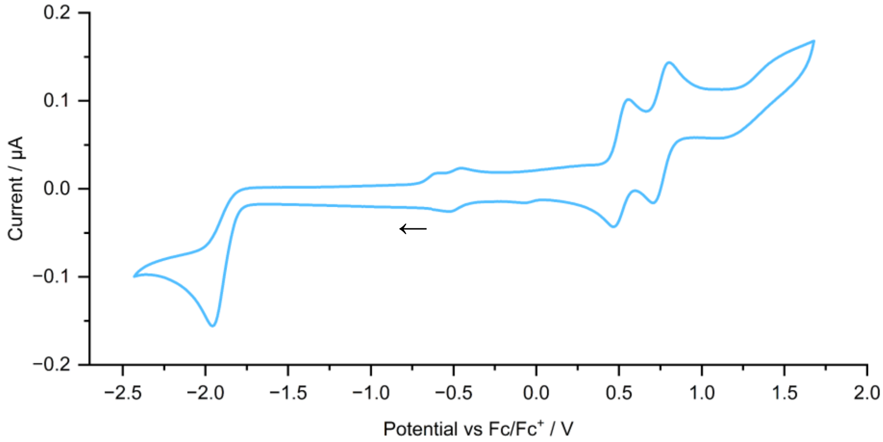

Figure S18. Cyclic voltammogram of **3ab** (0.5 mM in 0.1 M Bu_4NPF_6/CH_3CN), second scan.

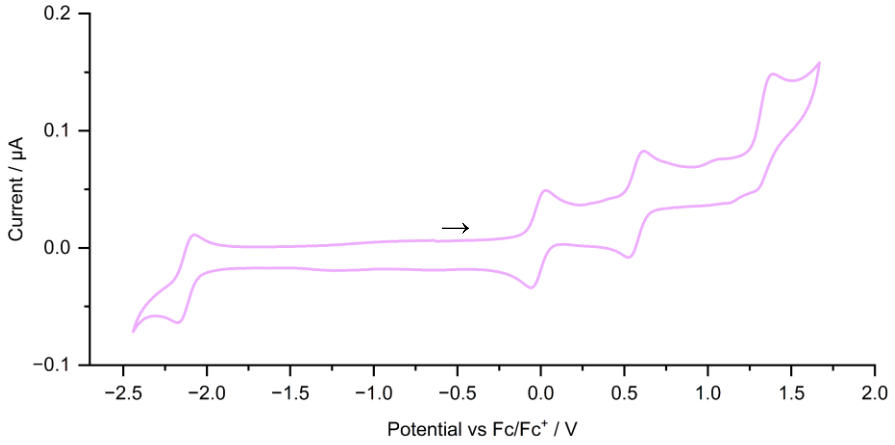

Figure S19. Cyclic voltammogram of **3ca** (0.5 mM in 0.1 M Bu_4NPF_6/CH_3CN), second scan.

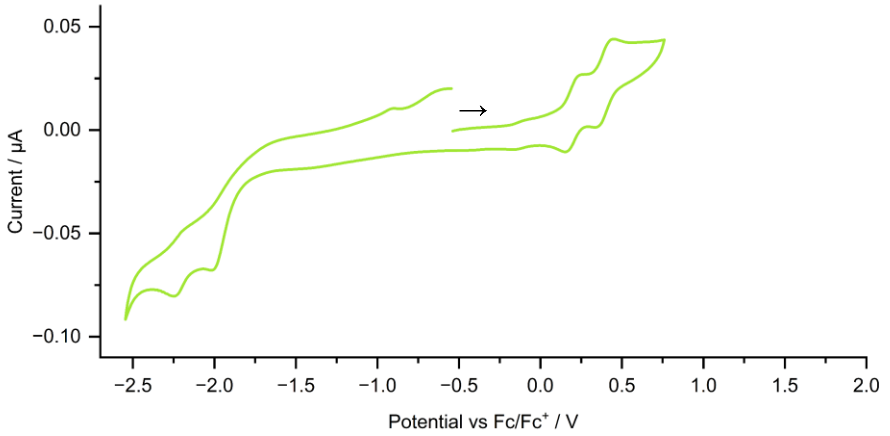

Figure S20. Cyclic voltammogram of **3cb** (0.5 mM in 0.1 M Bu_4NPF_6/CH_3CN), first scan.

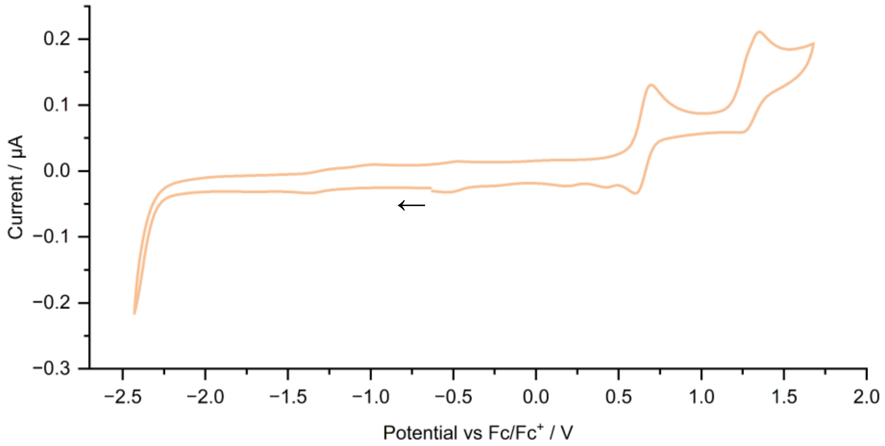

Figure S21. Cyclic voltammogram of **4ca** (0.5 mM in 0.1 M Bu_4NPF_6/CH_3CN), second scan.

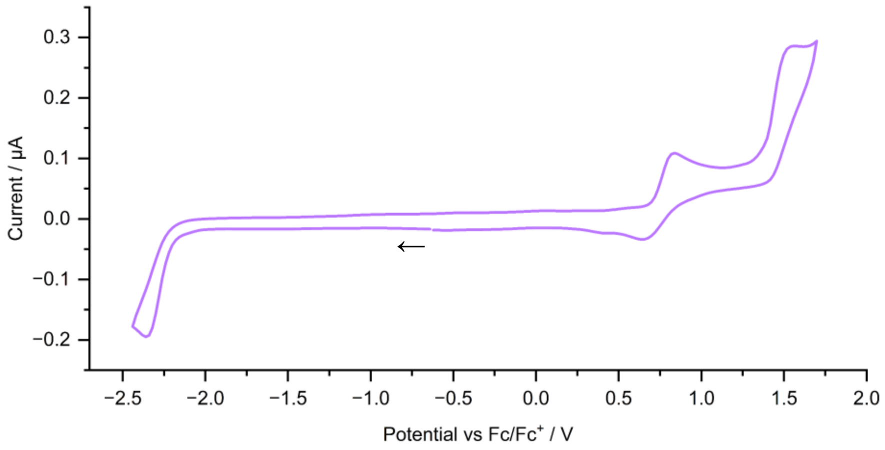

Figure S22. Cyclic voltammogram of **4cb** (0.5 mM in 0.1 M Bu_4NPF_6/CH_3CN), second scan.

Table S3. Overview of reduction potentials of **3aa**, **3ab**, **3ca**, **3cb**, **4ca** and **4cd** in CH_3CN with a scan rate of 200 mV/s.

Compound	Potential of first reduction ($E_{1/2,red1}$ or $E_{p,red1}$) vs Fc/Fc^+ / V[a]	Peak separation (ΔE_p) for reversible peaks / mV	Potential of second reduction ($E_{p,red2}$) vs Fc/Fc^+ / V
**3aa**	-2.15	81	/
**3ab**	-1.95	/	/
**3ca**	-2.13	81	/
**3cb**	-2.01	/	-2.25
**4ca**	/	/	/
**4cb**	-2.34	/	/

[a] $E_{1/2,red1}$ was used for reversible peaks, $E_{p,red1}$ for irreversible ones.

Table S4. Overview of oxidation potentials of **3aa**, **3ab**, **3ca**, **3cb**, **4ca** and **4cd** in CH_3CN with a scan rate of 200 mV/s.

Compound	Potential of first oxidation ($E_{1/2,ox1}$ or $E_{p,ox1}$) vs Fc/Fc^+ / V [a]	Peak separation (ΔE_p) for reversible peaks / mV	Potential of second oxidation ($E_{1/2,ox2}$ or $E_{p,ox2}$) vs Fc/Fc^+ / V [a]	Peak separation (ΔE_p) for reversible peaks / mV	Potential of third oxidation ($E_{p,ox3}$) vs Fc/Fc^+ / V
**3aa**	+0.03	81	+0.74	86	/
**3ab**	+0.50	71	+0.74	71	/
**3ca**	-0.01	81	+0.57	91	+1.39
**3cb**	+0.34	70	+0.54	91	/
**4ca**	+0.64	91	+1.35	/	/
**4cb**	+0.84	/	+1.54	/	/

[a] $E_{1/2,red1}$ was used for reversible peaks, $E_{p,red1}$ for irreversible ones.

7.2 H-Cell Cyclings

General Information: Galvanostatic cycling experiments were carried out in a customized H-Cell with a glass frit as a separator (Adams and Chittenden, P5) in a three-electrode setup in an argon-filled glove box. Carbon felt (≈ 1 cm x 1 cm) attached to a Ti wire was used as working and counter electrodes and the potential of the working electrode was controlled by an Ag/Ag^+ ($AgNO_3$ and NBu_4ClO_4 in CH_3CN) reference electrode. The working side was filled with **3aa** or **3ca** (6.5 mL, 0.5 mM solution in 0.1 M Bu_4NPF_6/CH_3CN) and the counter side with ferrocene (6.5 mL, 1 mM solution in 0.1 M Bu_4NPF_6/CH_3CN) or 2-(2,6-diisopropylphenyl)isoindoline-1,3-dione (6.5 mL, 1 mM (1.5 mM for second oxidation) solution in 0.1 M Bu_4NPF_6/CH_3CN) for the reductive and oxidative cycling, respectively. Both chambers were stirred continuously and a constant current of 0.5 mA was applied. The plots of normalized discharge capacity to the initial charging cycle vs the cycle number and of potentials vs Ag/Ag^+ vs time for the first cycles of the failed cyclings are shown below.

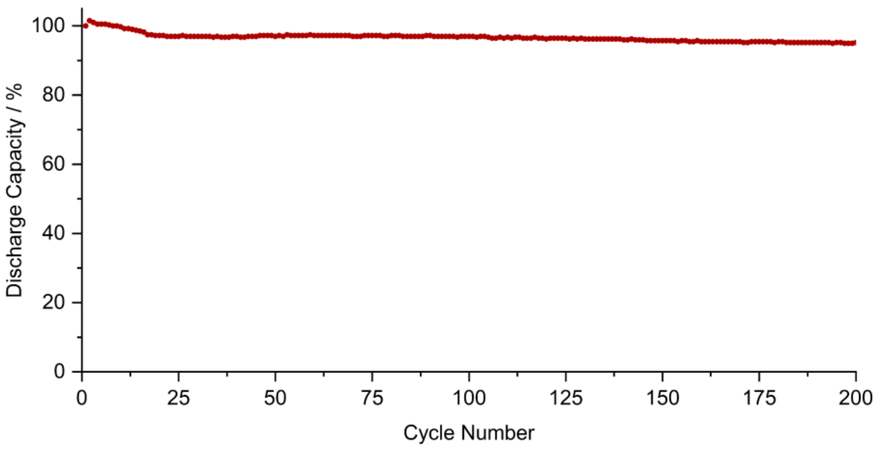

Figure S23. Plot of discharge capacity normalized to the initial charging cycle vs cycle number for H-cell cycling of the first oxidation of **3aa**.

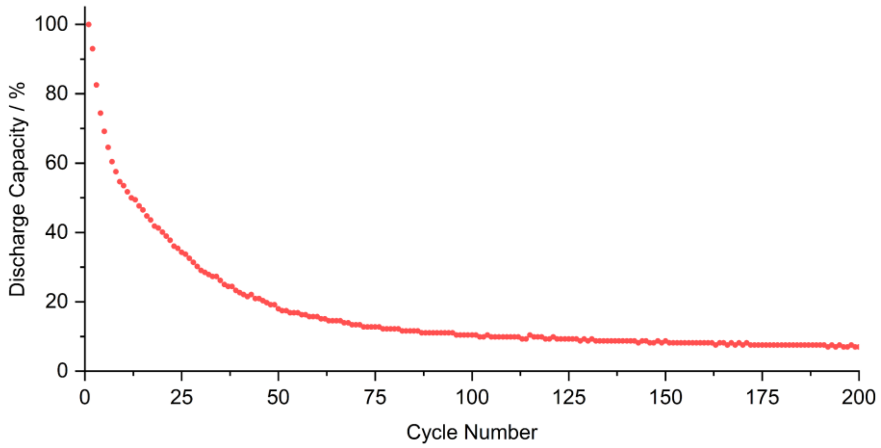

Figure S24. Plot of discharge capacity normalized to the initial charging cycle vs cycle number for H-cell cycling of the second oxidation of **3aa**.

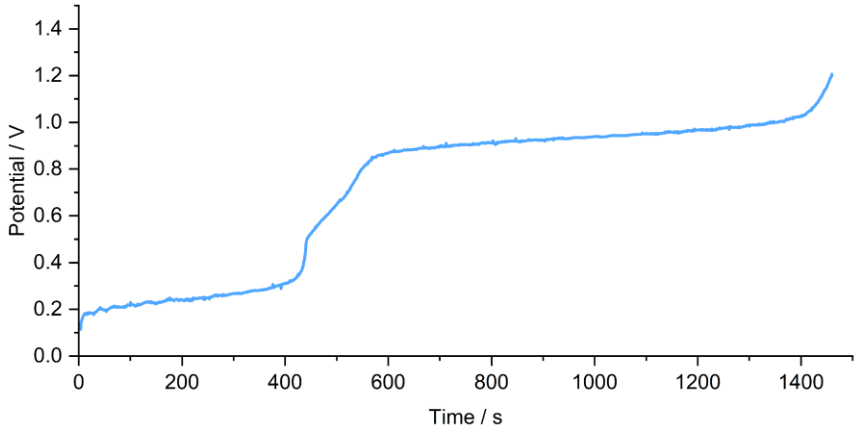

Figure S25. Plot of potential vs Ag/Ag$^+$ vs time for the first charging cycle of the second oxidation of **3aa**.

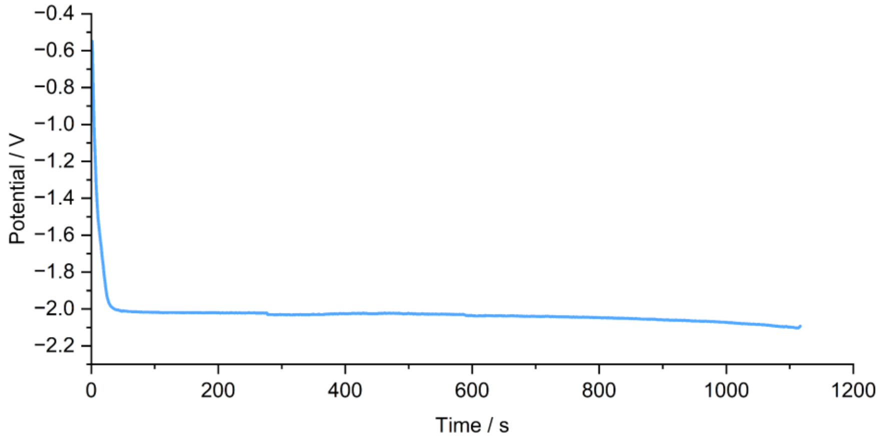

Figure S26. Plot of potential vs Ag/Ag$^+$ vs time for the first charging cycle of the reduction of **3aa**.

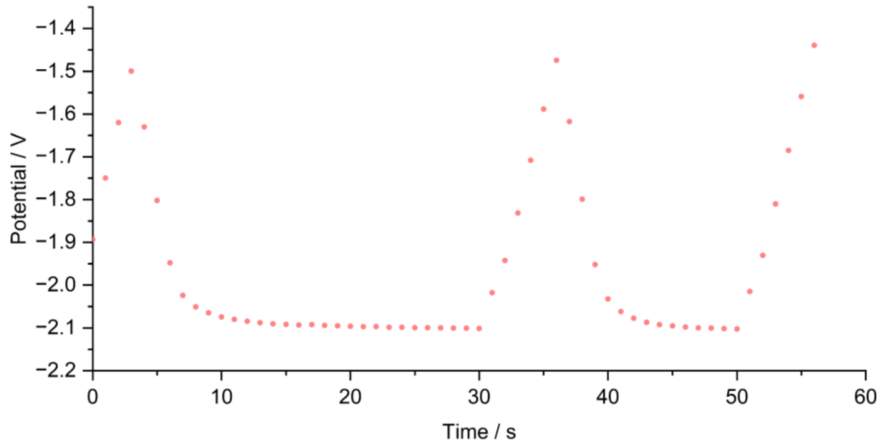

Figure S27. Plot of potential vs Ag/Ag$^+$ vs time for the following cycles of the reduction of **3aa**.

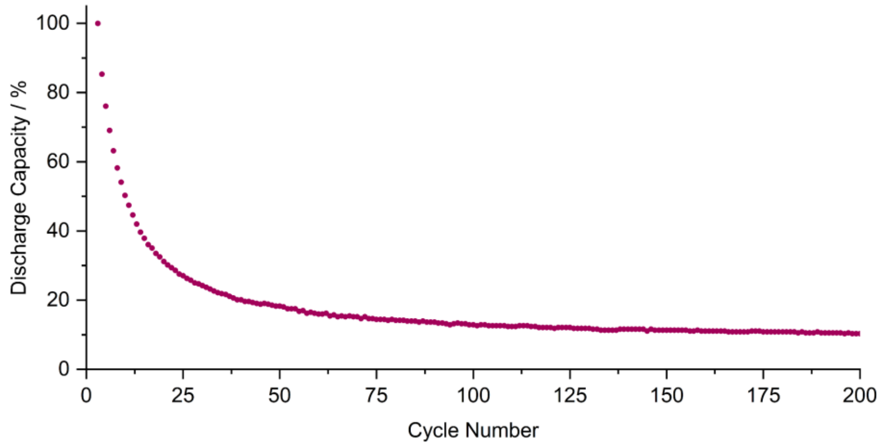

Figure S28. Plot of discharge capacity normalized to the initial charging cycle vs cycle number for H-cell cycling of the reduction of **3ca**.

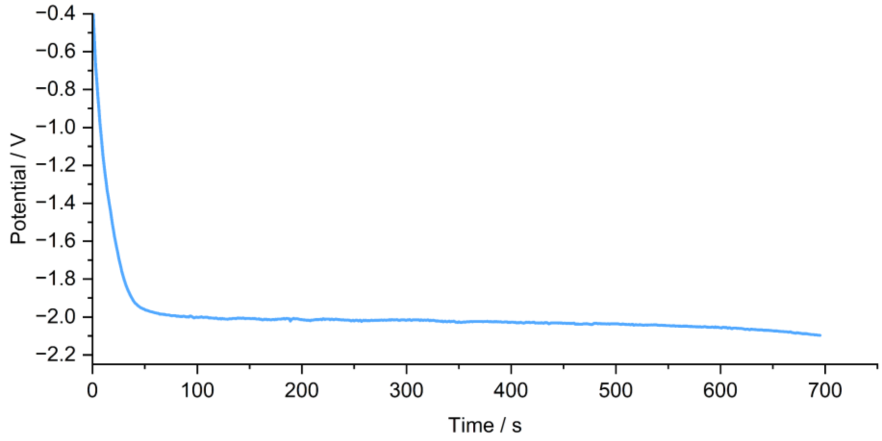

Figure S29. Plot of potential vs Ag/Ag$^+$ vs time for the first charging cycle of the reduction of **3ca**.

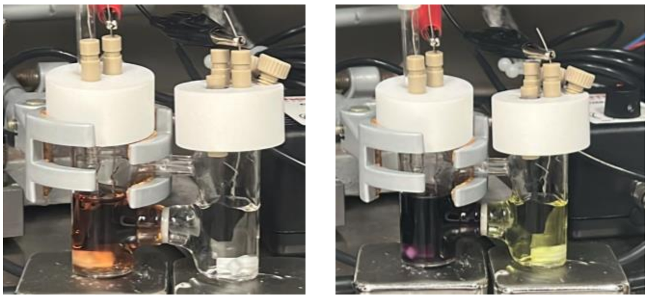

Figure S1. Visual appearance of **3aa** (left picture, left half-cell) and **3aa**$^{•+}$ (right picture, left half-cell) during H-cell cycling.

7.3 Synthesis and Characterization of Different Redox States of 3aa

Synthesis:

Route A: A solution of **3aa** (6.2 mg, 15 µmol, 1.0 equiv.) in CH_3CN (4 mL) and a solution of $Na_2S_2O_8$ (3.6 mg, 15 µmol, 1.0 equiv.) in water (2 mL) were degassed by bubbling argon through the solutions for 10 min. Then, the $Na_2S_2O_8$ solution was transferred into the solution of **3aa** and the resulting mixture was stirred at 80 °C for 1.5 h, whereby a color change from light red to dark purple was observed after 5 min. The formation of the radical cation was confirmed by HRMS.

HRMS (ESI): m/z calculated for $[C_{27}H_{18}N_4O]^{•+}$: 414.1475, found: 414.1466.

Route B: **3aa** (3.1 mg, 7.7 µmol, 1.0 equiv.) was dissolved in CH_2Cl_2 (0.5 mL) and the solution was cooled to 0 °C. Then, $NOBF_4$ (2.2 mg, 18.8 µmol, 2.5 equiv.) was added and the mixture was stirred at 0 °C for 1.5 h during which the mixture turned dark purple. Afterwards, Et_2O (2 mL) was added and the formed dark purple precipitate was collected by filtration. The formation of the radical cation was confirmed by HRMS.

HRMS (ESI): m/z calculated for $[C_{27}H_{18}N_4O]^{•+}$: 414.1475, found: 414.1477.

Unfortunately, oxidation to the dication was not possible using these chemical methods.

Electrochemical synthesis: The electrochemical oxidations and reduction were performed in a customized H-Cell using the same procedure as described in chapter 7.2. The working side of the H-Cell was filled with **3aa** (6.5 mL, 0.5 mM solution in 0.1 M Bu_4NPF_6/CD_3CN for oxidation 0.5 mM solution in 0.1 M Bu_4NPF_6/CH_3CN for reduction) and the counter side with 2-(2,6-diisopropylphenyl)isoindoline-1,3-dione (6.5 mL, 2 mM solution in 0.1 M NBu_4PF_6/CD_3CN) for oxidation or ferrocene (6.5 mL, 1 mM solution in 0.1 M Bu_4NPF_6/CH_3CN) for reduction. For analysis an aliquot of the respective solution was removed after the charging was completed.

The measured EPRs of the radical anion and the radical cation and ^{1}H-NMR of the dication of **3aa** are shown below.

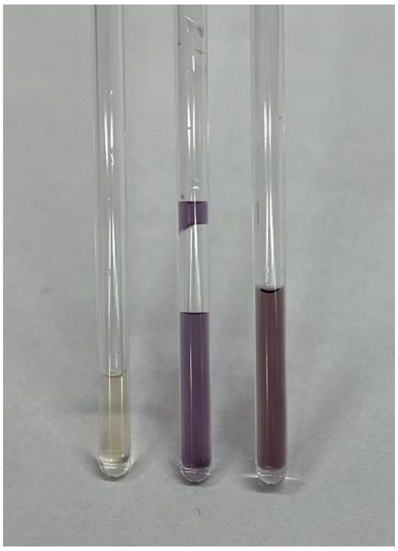

Figure S31. Appearance of different redox states of **3aa**. Left: radical anion, middle: radical cation, right: dication.

EPR Measurements:

The liquid-state EPR experiments were performed on a Bruker EMXnano spectrometer operating at Xband (9,6 GHz) in the range from 0–6000 G with a microwave power of 0.32 mW. For the measurements 1000 or 2000 data points were recorded using 170 to 1000 scans. The modulation frequency was set to 100 kHz and a modulation amplitude of 1.0 G and a time constant of 0.16 ms were used. The resulting data were processed and simulated using the fitting program SpinFit by BRUKER and the simulation software Easyspin.

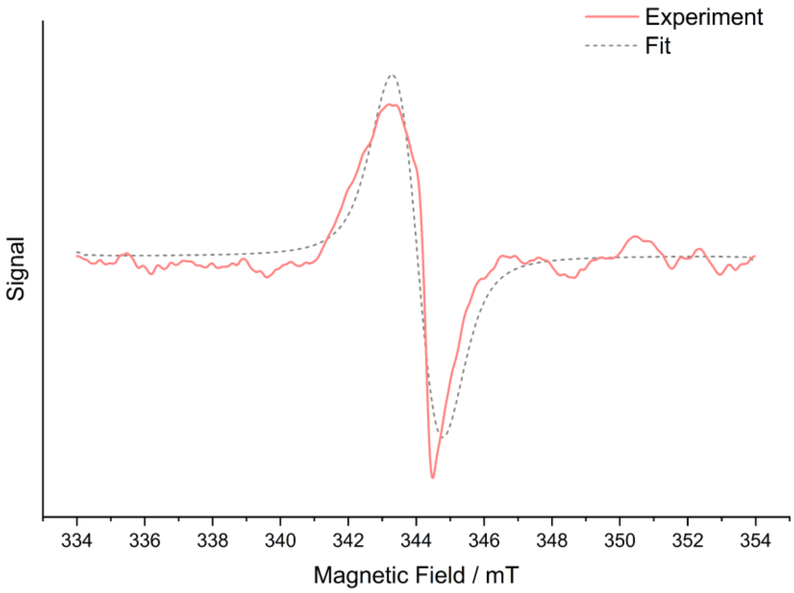

Figure S32. EPR spectrum of the radical anion of **3aa**.

Experimental parameters for the best fit with the experimental spectrum for the radical anion:

S = 0.5

Nuclei for hyperfine coupling: N (n = 1)

g-factor = 2.0029

A_N = 2.3784

lw_N = 1.2549

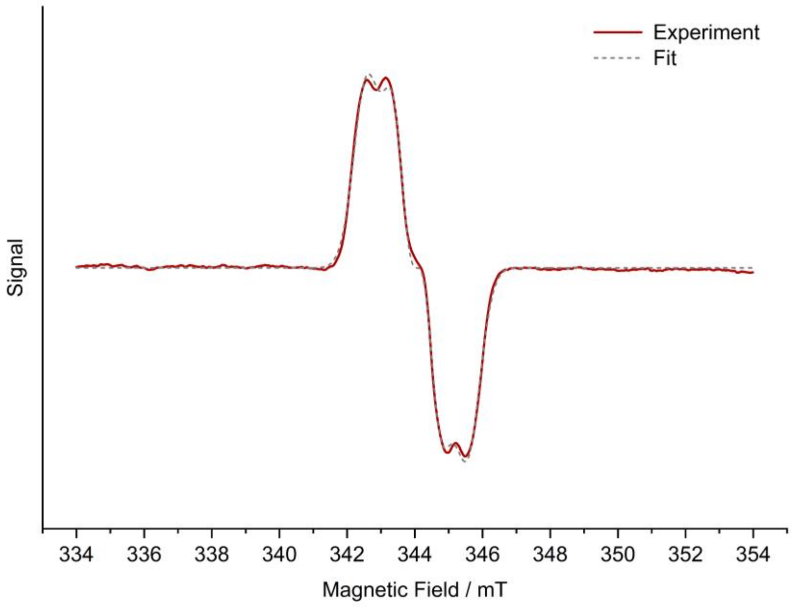

Figure S33. EPR spectrum of the radical cation of **3aa**.

Experimental parameters for the best fit with the experimental spectrum for the radical cation:

S = 0.5

Nuclei for hyperfine coupling: N (n = 1), H (n = 1)

g-factor = 2.0037

A_N = 22.4093

A_H = 20.8676

lw_N = 0.9986

lw_H = 0.0103

NMR of the dication of 3aa in comparison to the neutral state:

^{1}H-NMR (500 MHz for dication of **3aa**, 600 MHz for **3aa**, CD_3CN):

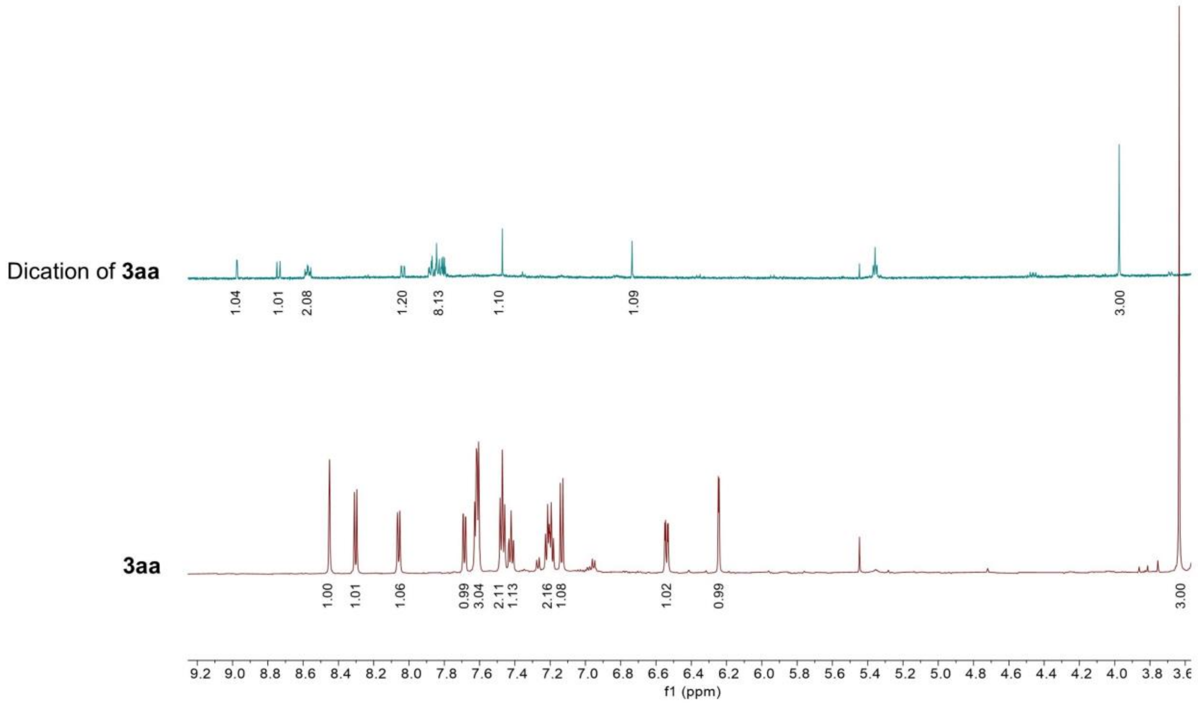

7.4 Spectroelectrochemical Measurements

General Information: The spectroelectrochemical measurements were carried out using an AVA-Light DH-S-BAL light source, an AVASpec2024 spectrometer and a Metrohm PGSTAT204 potentiostat. Acetonitrile was degassed via three freeze-pump-thaw cycles before use, dried over molecular sieves and constantly stored in the glove box. The respective compound was dissolved in the electrolyte (3.5 mL 0.1 M Bu_4NPF_6/CH_3CN) to give a concentration of 0.5 mM. A platinum gauze electrode was used as a working electrode and the potential was monitored with a pseudo-Ag/AgCl reference electrode (previously referenced to ferrocene in the same electrolyte). Glassy carbon press-fitted into PEEK was used as a counter electrode. The scan rate was set to 50 mV/s and the UV-vis absorption was measured every 0.0244 V.

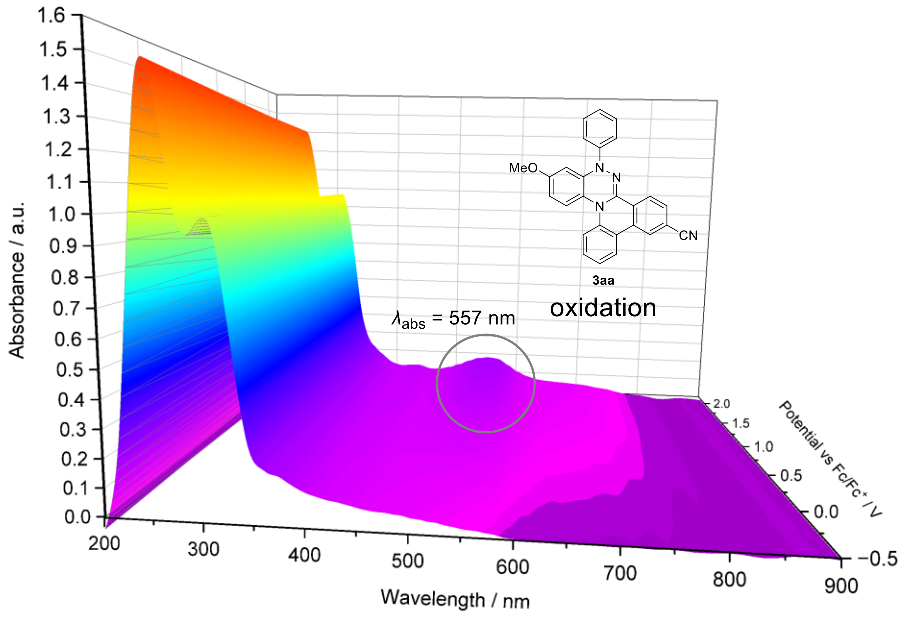

Figure S34. Spectroelectrochemical measurement of the oxidation of **3aa**.

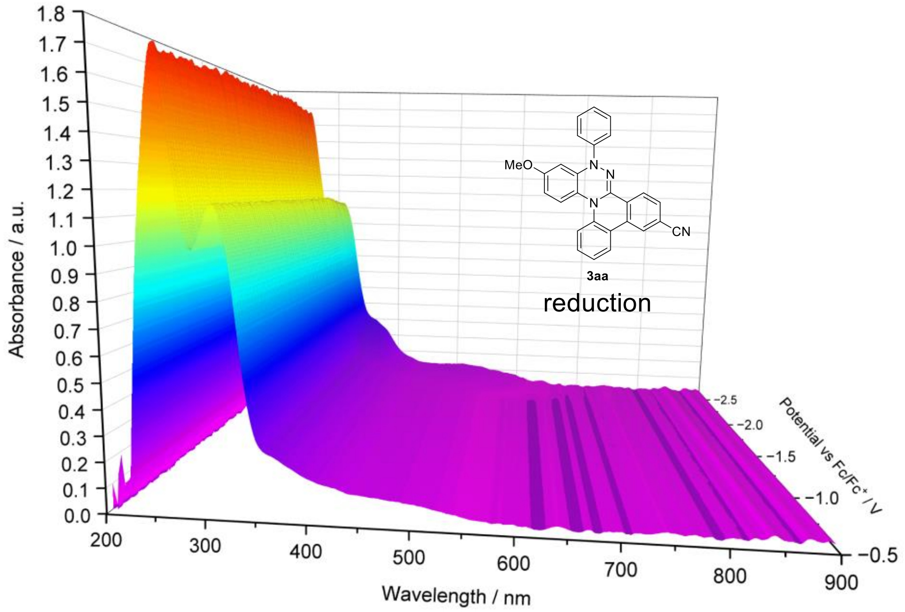

Figure S35. Spectroelectrochemical measurement of the reduction of **3aa**.

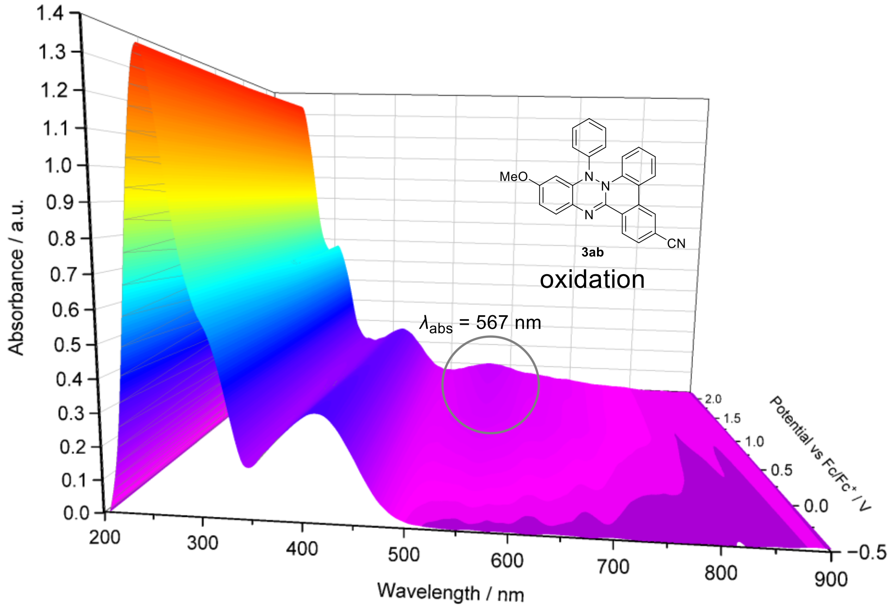

Figure S36. Spectroelectrochemical measurement of the oxidation of **3ab**.

8. Investigation of Inversion Barriers and Half-life Times

8.1 Separation of the Enantiomers on the Chiral HPLC

General Information: The separations on the reversed phase HPLC were performed on an Agilent Series 1200 HPLC device with a Chiralcel OJ-RH column (4.6 x 150 mm x 5 µm, Daicel Chemical Industries, LTD.) using a CH_3CN/H_2O solvent mixture. The separations on the normal phase HPLC were performed on a Hewlett Packard Series 1100 HPLC device with Chiralpak AD-H column (4.6 x 250 mm x 5 µm, Daicel Chemical Industries, LTD.) column with a cyclohexane/isopropanol solvent mixture. Both peaks were detected by UV-detection (210 nm, 230 nm, 250 nm, 260 nm, 280 nm).

<u>Separation of the enantiomers of **3ab:**</u>

Separation conditions: Reversed phase HPLC, Chiralcel OJ-RH column, 60:40 MeCN/H_2O, 0.5 mL/min

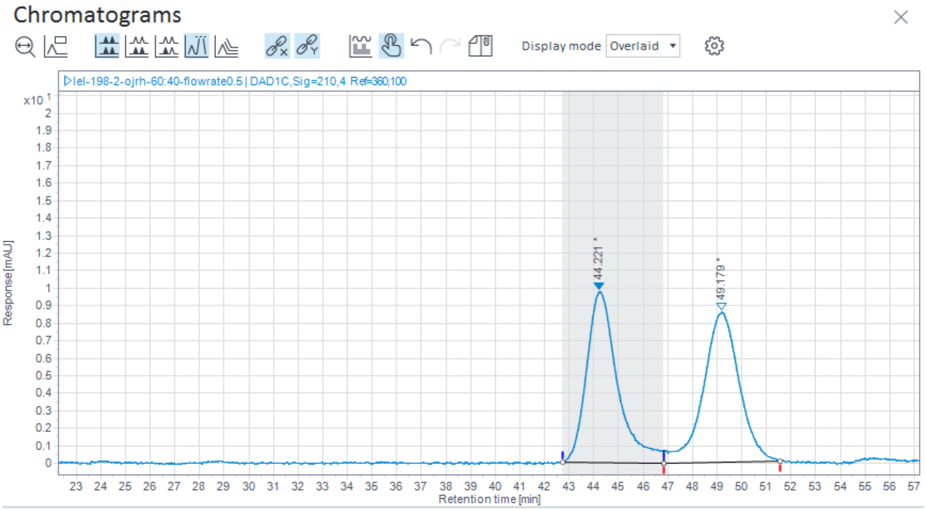

Injection Results

Peaks Summary

#	Signal description	RT (min)	Area	Area%	Height	Width
1	DAD1C,Sig=210,4 Ref=360,100	44.221	856.820	49.733	9.791	1.037
2	DAD1C,Sig=210,4 Ref=360,100	49.179	866.020	50.267	8.588	1.188

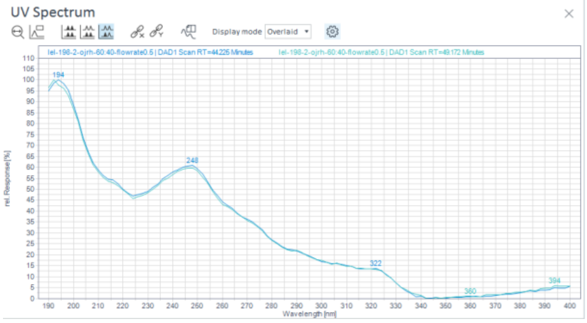

Figure S2. Details of the HPLC separation of the enantiomers of **3ab**.

Separation of the enantiomers of **3cb**:

Separation conditions: Normal phase HPLC, Chiralpak AD-H column, 98:2 cyclohexane/iPrOH, 1 mL/min

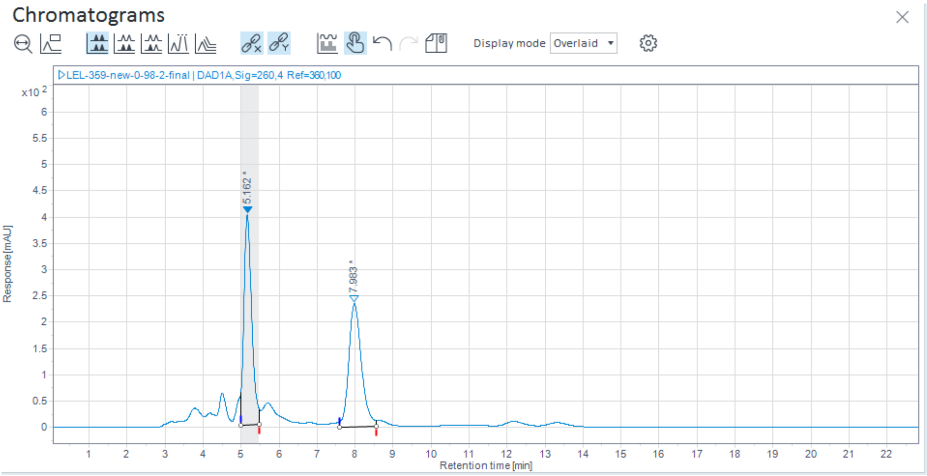

Injection Results

#	Signal description	RT (min)	Area	Area%	Height	Width
1	DAD1A,Sig=260,4 Ref=360,100	5.162	5647.331	50.654	398.923	0.217
2	DAD1A,Sig=260,4 Ref=360,100	7.983	5501.558	49.346	234.743	0.357

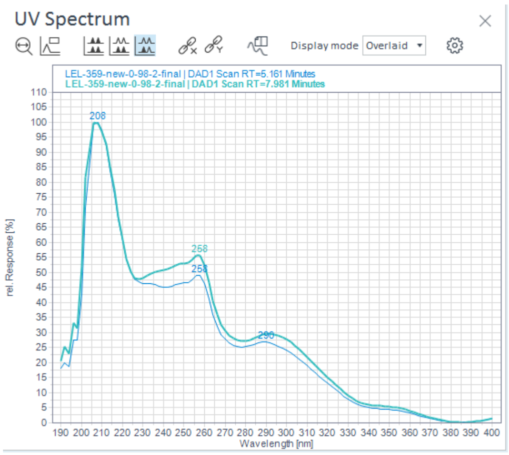

Figure S3. Details of the HPLC separation of the enantiomers of **3cb**.

Separation of the enantiomers of **3db**:

Separation conditions: Normal phase HPLC, Chiralpak AD-H column, 99.5:0.5 cyclohexane/iPrOH, 1 mL/min

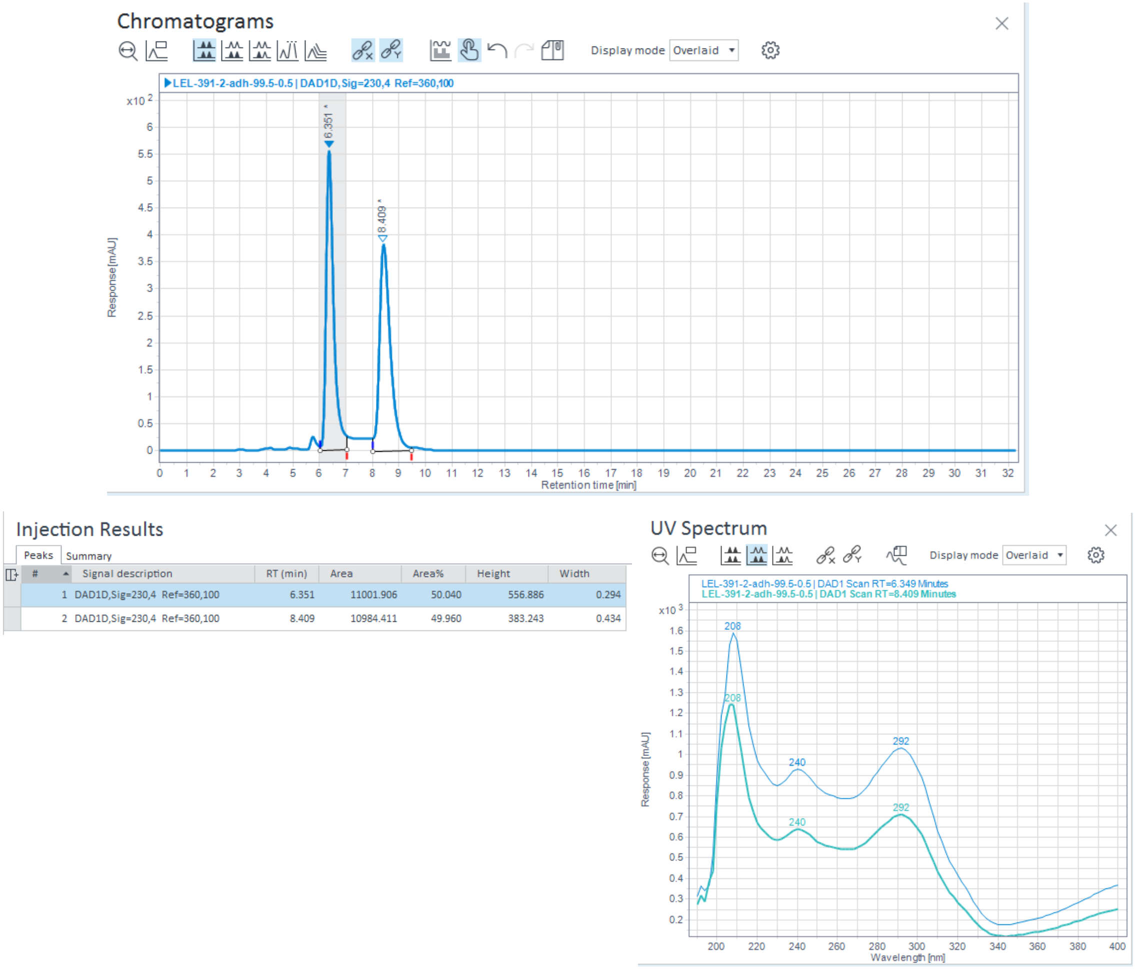

Figure S4. Details of the HPLC separation of the enantiomers of **3db**.

8.2 Calculation of Inversion Barriers and Half-Lives

General procedure: The separation of the enantiomers on the respective HPLC was performed three times with an injection volume of 50 µL and the enantiomers were collected separately. After each collection the solvent was removed *in vacuo* directly. The combined fractions of the respective enantiomers were dissolved again and the *ee*s of the dissolved compounds were measured after certain time intervals. With these results Eyring plots (ln(ee_0/ee_t) vs time) were created and the half-life time and the inversion barrier were calculated from the slope of the line of the Eyring plots (k_r) using the following equations.

k_r : slope of line in Eyring plot; $t_{1/2}$: half-lives; $\Delta G^{\neq}$: inversion barrier

$$k_e = \frac{k_r}{2}$$

$$t_{1/2} = \frac{\ln 2}{k_r}$$

Eyring equation: $k_e = \frac{k_B T}{h} e^{-\frac{\Delta G^{\neq}}{RT}}$

$$\Leftrightarrow \Delta G^{\neq} = -RT \ln \frac{k_e h}{k_B T}$$

For every compound the measurement was performed twice and the half-life times and inversion barriers of **3ab**, **3cb** and **3db** were calculated as the average of the different measurements. The obtained ees after certain time intervals and Eyring plots are shown below.

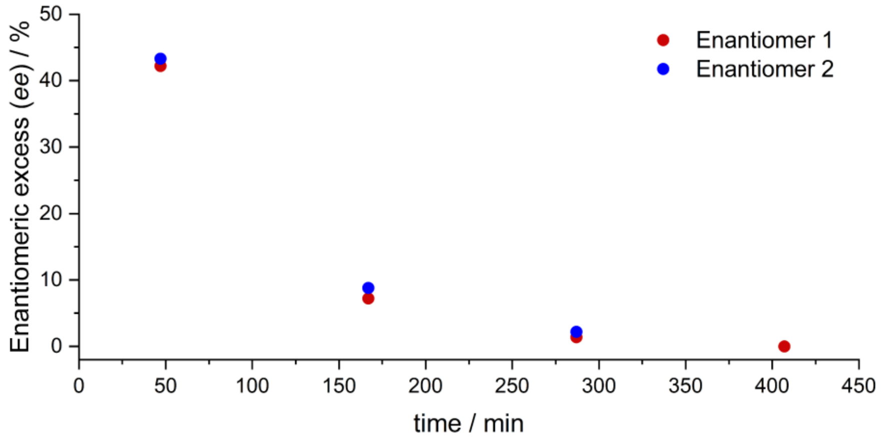

Figure S40. Measured *ee*s of **3ab** after storing the respective enantiomer in solution, measurement 1.

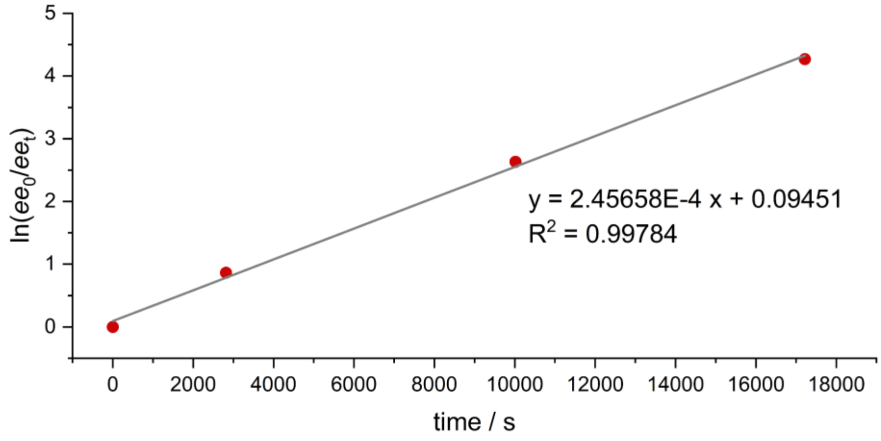

Figure S41. Eyring plot of the measured *ee*s after certain time intervals of enantiomer 1 of **3ab**, measurement 1.

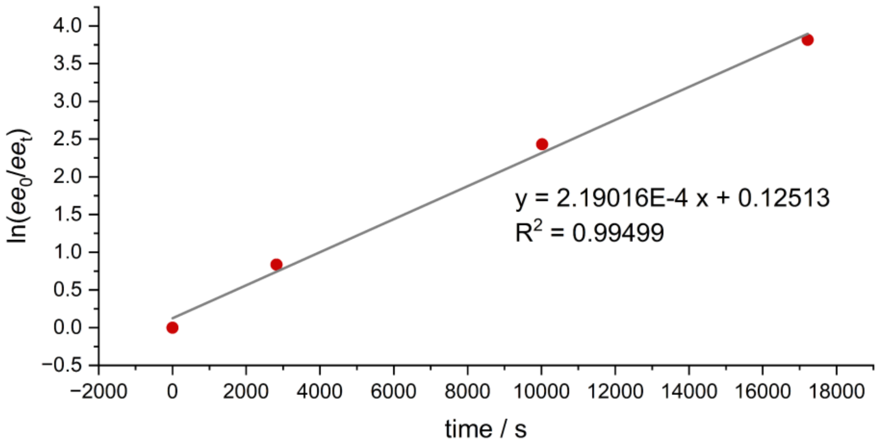

Figure S42. Eyring plot of the measured *ee*s after certain time intervals of enantiomer 2 of **3ab**, measurement 1.

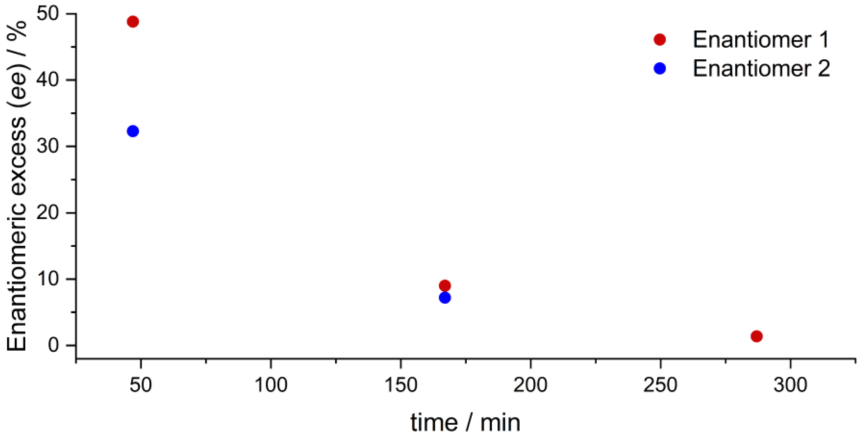

Figure S43. Measured *ee*s of **3ab** after storing the respective enantiomer in solution, measurement 2. As for enantiomer 2 only two data points could be recorded it is not considered for the calculation of the half-life time and the inversion barrier.

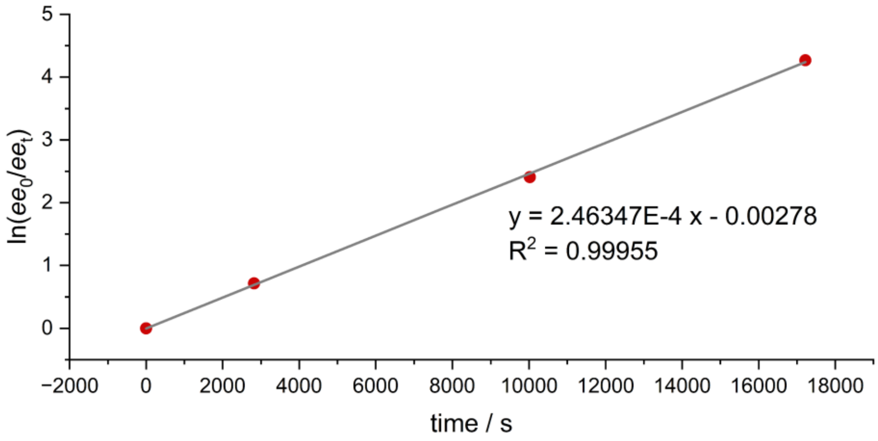

Figure S44. Eyring plot of the measured *ee*s after certain time intervals of enantiomer 1 of **3ab**, measurement 2.

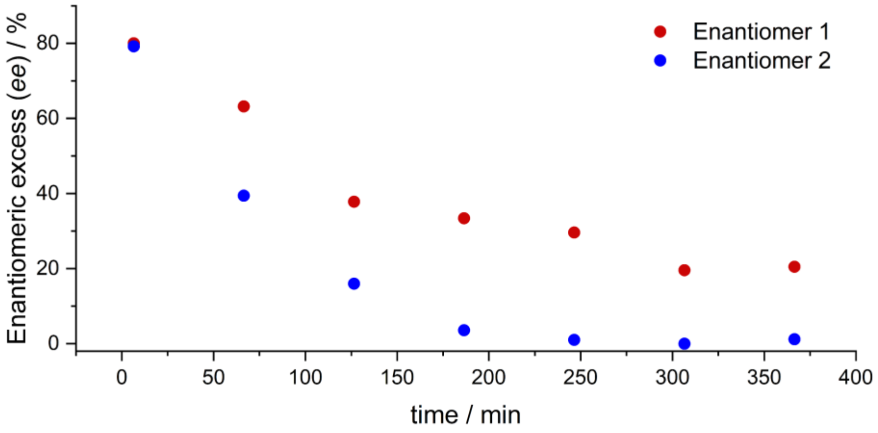

Figure S45. Measured *ee*s of **3cb** after storing the respective enantiomer in solution, measurement 1.

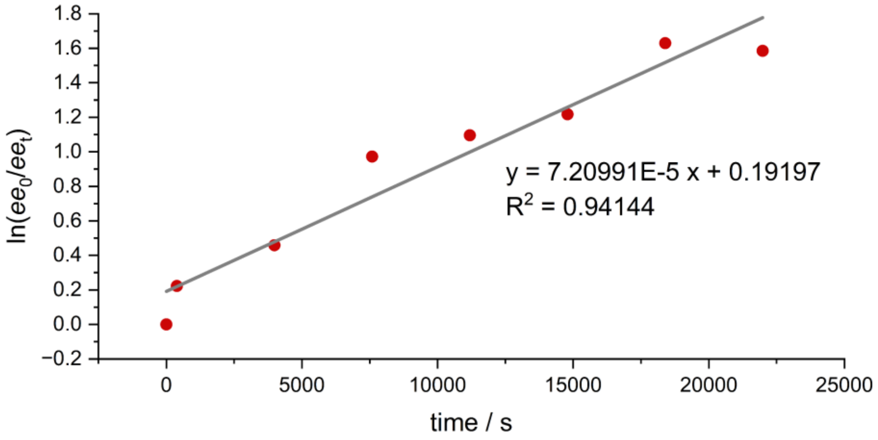

Figure S46. Eyring plot of the measured *ee*s after certain time intervals of enantiomer 1 of **3cb**, measurement 1.

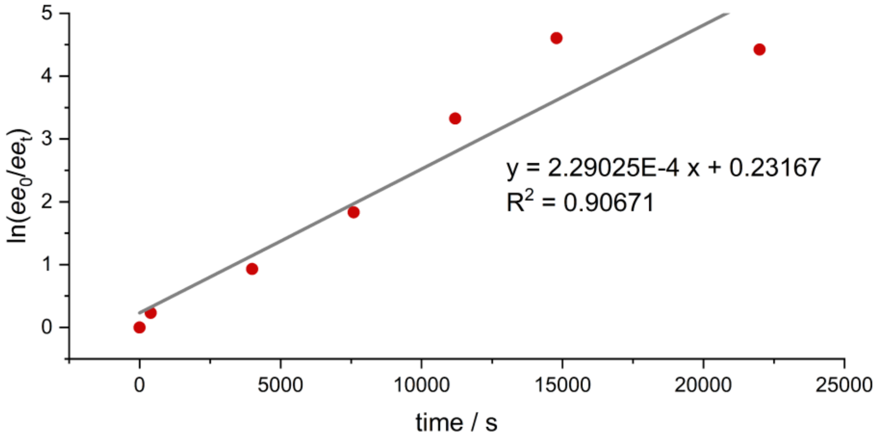

Figure S47. Eyring plot of the measured *ee*s after certain time intervals of enantiomer 2 of **3cb**, measurement 1.

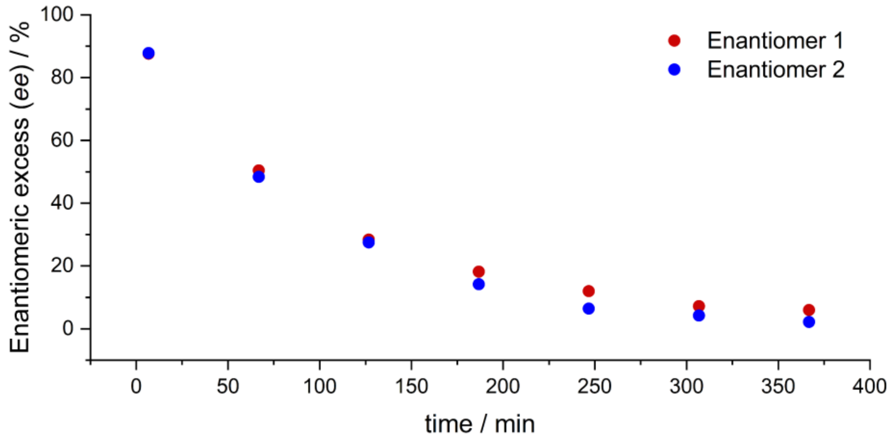

Figure S48. Measured *ee*s of **3cb** after storing the respective enantiomer in solution, measurement 2.

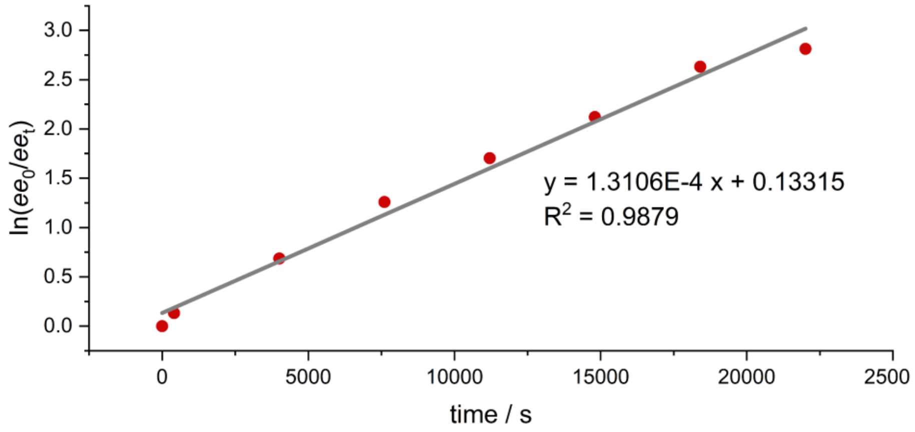

Figure S49. Eyring plot of the measured *ee*s after certain time intervals of enantiomer 1 of **3cb**, measurement 2.

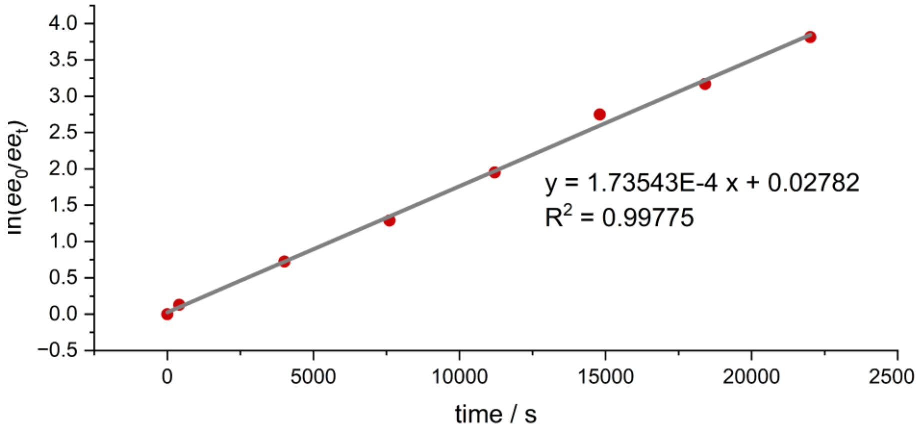

Figure S50. Eyring plot of the measured *ee*s after certain time intervals of enantiomer 2 of **3cb**, measurement 2.

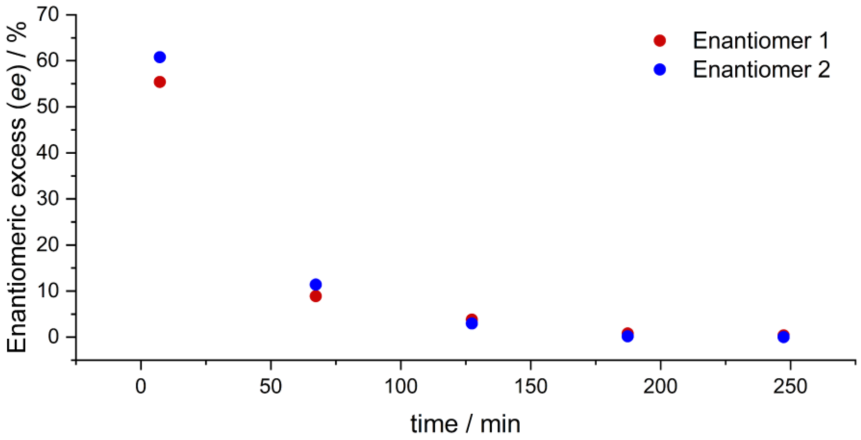

Figure S51. Measured *ee*s of **3db** after storing the respective enantiomer in solution, measurement 1.

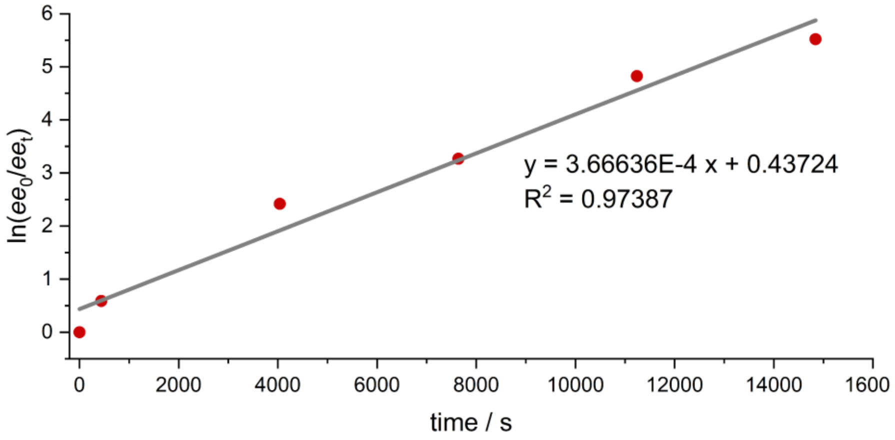

Figure S52. Eyring plot of the measured *ee*s after certain time intervals of enantiomer 1 of **3db**, measurement 1.

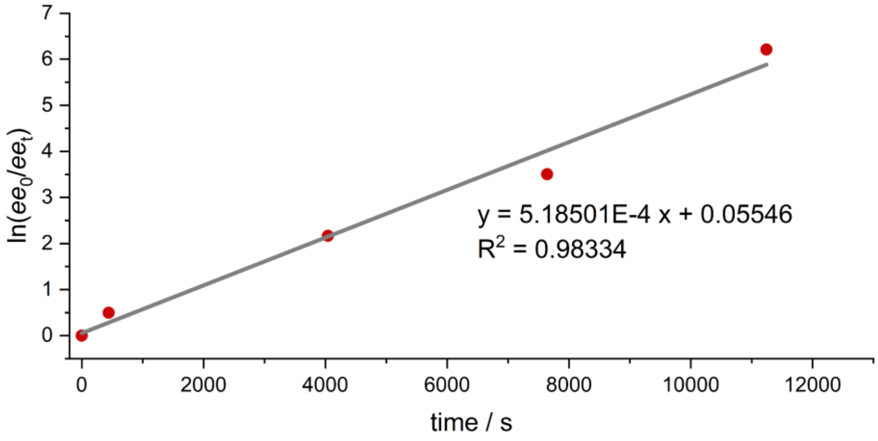

Figure 53. Eyring plot of the measured *ee*s after certain time intervals of enantiomer 2 of **3db**, measurement 1.

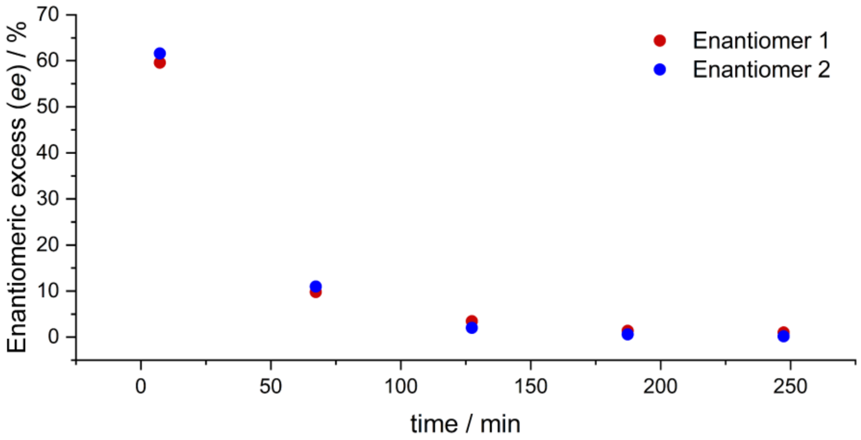

Figure S54. Measured *ee*s of **3db** after storing the respective enantiomer in solution, measurement 2.

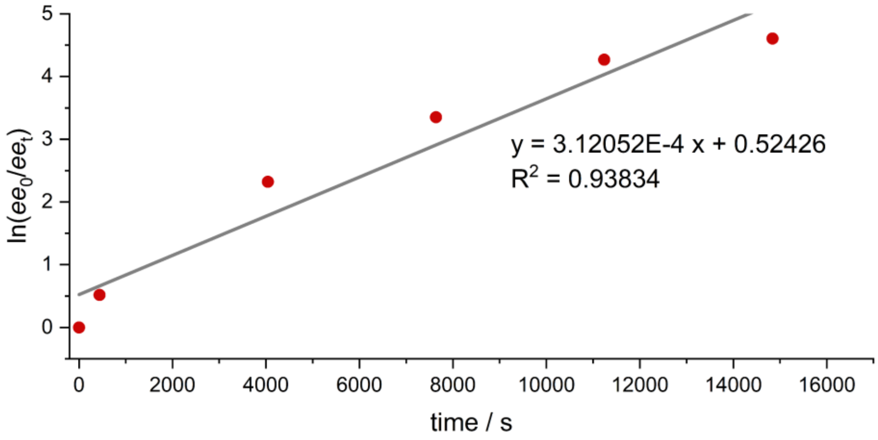

Figure S55. Eyring plot of the measured *ee*s after certain time intervals of enantiomer 1 of **3db**, measurement 2.

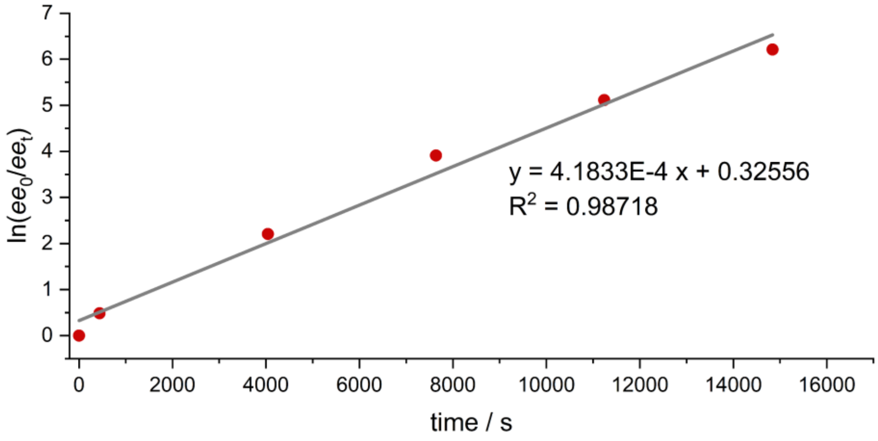

Figure 56. Eyring plot of the measured *ee*s after certain time intervals of enantiomer 2 of **3db**, measurement 2.

Table S5. Summary of calculated half-lives and inversion barriers of **3ab**, **3cb** and **3db** in the different measurements.

	**Slope in Eyring plot**	**$t_{1/2}$ / min**	**Inversion barrier / J**	**Inversion barrier / kcal/mol**
**3ab** measurement 1 enantiomer 1	2.45658E-4	47.02657	93701.61229	22.39525
**3ab** measurement 1 enantiomer 2	2.19016E-4	52.74707	93981.39761	22.46212
**3ab** measurement 2 enantiomer 1	2.46347E-4	46.89504	93694.78607	22.39362
**3cb** measurement 1 enantiomer 1	7.20991E-5	160.2302	96689.43294	23.10935
**3cb** measurement 1 enantiomer 2	2.29025E-4	50.44189	93872.48577	22.43609
**3cb** measurement 2 enantiomer 1	1.3106E-4	88.14629	95232.89956	22.76123
**3cb** measurement 2 enantiomer 2	1.73543E-4	66.56825	94548.59271	22.59768
**3db** measurement 1 enantiomer 1	3.66636E-4	31.50933	92725.66657	22.16199
**3db** measurement 1 enantiomer 2	5.18501E-4	22.28048	91880.98323	21.96011
**3db** measurement 2 enantiomer 1	4.1833E-4	27.61565	92404.19082	22.08516
**3db** measurement 2 enantiomer 2	3.12052E-4	37.02092	93118.55037	22.25589

For the calculation of the average inversion barriers and half-lives of 3cb the values of measurement 1, enantiomer 1 were omitted as these are outliers.

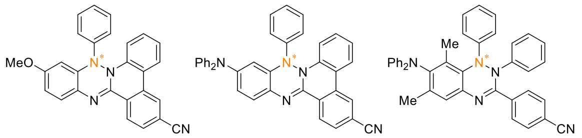

3ab
Inversion barrier:
22.4 kcal/mol
$t_{1/2}$ = 47 min

3cb
Inversion barrier:
22.6 kcal/mol
$t_{1/2}$ = 68 min

3db
Inversion barrier:
22.1 kcal/mol
$t_{1/2}$ = 30 min

Figure S57. Calculated average inversion barriers and half-life times of **3ab**, **3cb** and **3db**.

9. DFT Calculations

General Information: All calculations were performed with Gaussian 16 Revision B.01.[19] Geometries for the calculation of absorption properties were optimized at the state were optimized at the CAM-B3LYP/6-31G(d) level of theory including CH_2Cl_2 as a solvent using the conductor-like polarizable continuum model (CPCM). The optimized structures were confirmed as potential energy minima by vibrational frequency calculations as no imaginary frequencies were obtained. These optimized geometries were employed for the TD-DFT calculations and anisotropy of current induced density (ACID) plots[20] at the same level of theory. For the estimation of the nucleus independent chemical shift (NICS),[21–23] GIAO calculations were performed at the HF/6-31+G(d,p) level with ghost atoms placed at the centers of the triazine or tetrazine rings. For the calculation of the spin densities of the radical anion and the radical cation of **3aa** and of the Blatter radical **2a** the geometries were optimized at the CAM-UB3LYP/6-31G(d) level of theory including CH_3CN using the CPCM. These optimized structures were also confirmed as potential energy minima by vibrational frequency calculations. The cartesian coordinates of all optimized structures are shown below.

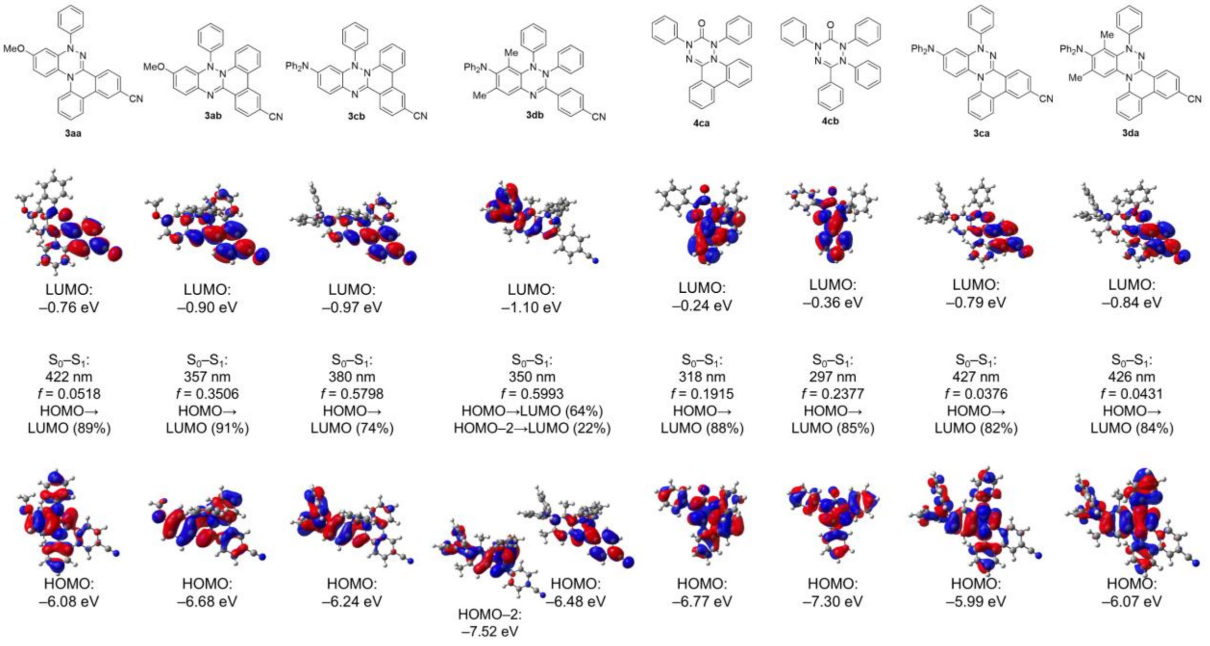

Figure S58. Kohn-Sham molecular orbitals and electronic transitions of **3aa**, **3ab**, **3cb**, **3db**, **4ca**, **4cb**, **3ca** and **3da** calculated on the CAM-B3LYP/6-31G(d) level of theory including CH_2Cl_2 as a solvent using the CPCM.

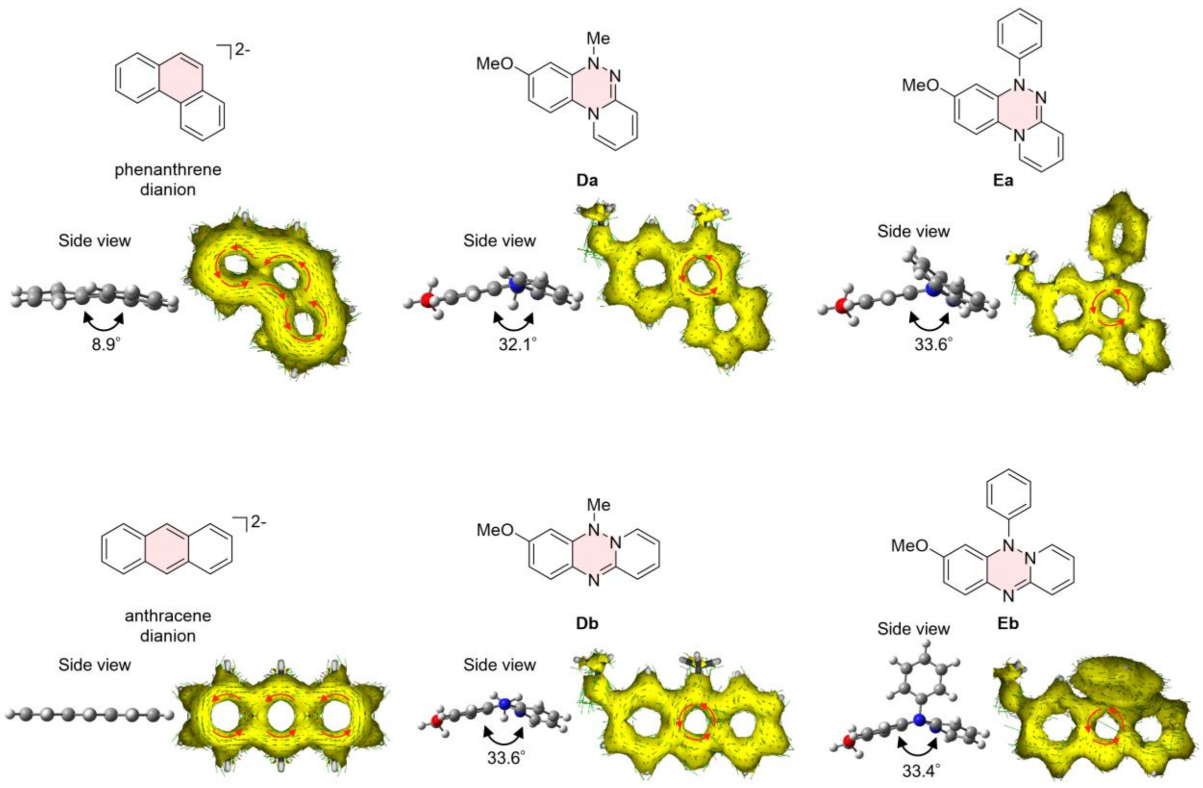

Figure S59. Optimized structures and ACID plots of model compounds calculated at the CAM-B3LYP/6-31G(d) level of theory.

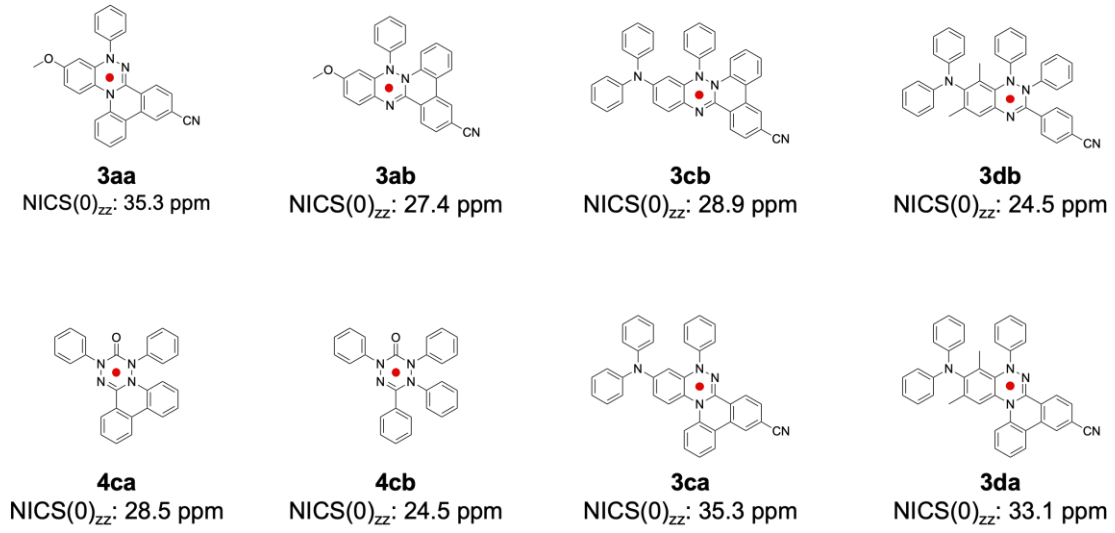

Figure S60. NICS(0)$_{zz}$ values for a series of derivatives calculated at the GIAO-HF/6-31+G(d,p) level of theory.

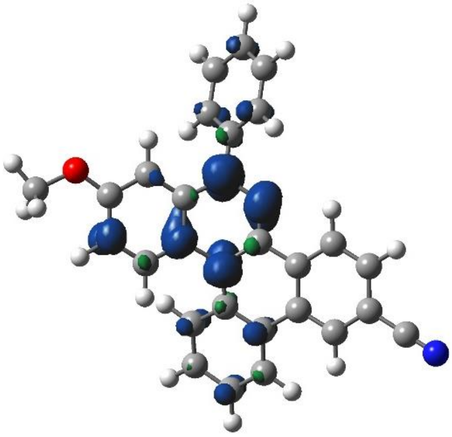

Figure S61. Calculated spin density of the radical cation of **3aa** including CH_3CN as a solvent using the CPCM with an isovalue of 0.004.

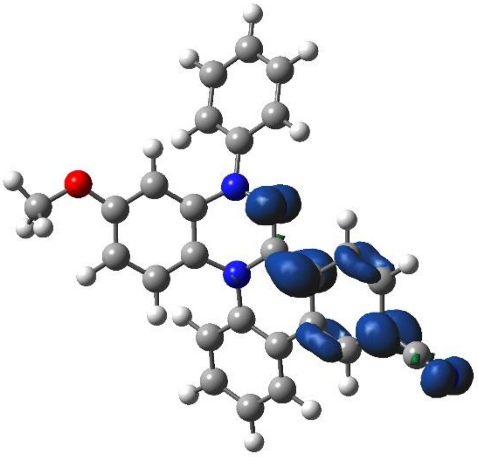

Figure S62. Calculated spin density of the radical anion of **3aa** in CH_3CN including CH_3CN as a solvent using the CPCM with an isovalue of 0.004.

Spin densities at the Nitrogen atoms of Blatter radical **2a** in CH_3CN:

N1: 0.275999

N2: 0.310594

N4: 0.352161

Cartesian coordinates of the optimized structures:

Table S6. Cartesian coordinates of the optimized structure for **3aa** in S_0 calculated at the CAM-B3LYP/6-31G(d) level of theory including CH_2Cl_2 as a solvent using the CPCM.

atom	X	Y	Z	atom	X	Y	Z
C	3.257786	-2.075324	-1.275179	C	4.544489	3.597075	1.301959
C	2.179047	-2.953378	-1.389228	C	4.629576	2.297811	1.79419
C	0.937084	-2.589249	-0.894609	C	3.63778	1.369669	1.506592
C	0.77532	-1.377999	-0.231321	O	4.435488	-2.510779	-1.790715
C	1.845203	-0.489422	-0.141516	C	5.563178	-1.656603	-1.708702
C	3.087199	-0.827809	-0.676631	C	-5.94074	2.186765	-1.128505

N	-0.472634	-0.895092	0.266808	N	-7.015937	2.528824	-1.384269
C	-0.66822	0.474216	0.069483	H	2.328173	-3.910953	-1.874434
N	0.30254	1.309179	0.09919	H	0.094728	-3.260842	-1.009559
N	1.546671	0.764656	0.440157	H	3.90166	-0.120876	-0.617149
C	-1.514835	-1.71523	0.718847	H	-0.213063	-3.289532	1.391945
C	-2.842993	-1.257484	0.633954	H	-2.037784	-4.707059	2.232114
C	-3.107741	0.059668	0.034357	H	-4.398056	-3.920023	2.086338
C	-2.033899	0.927436	-0.221648	H	-4.888038	-1.726471	1.114237
C	-1.23975	-2.957538	1.300753	H	-5.25163	-0.149986	-0.128253
C	-2.269626	-3.748491	1.77941	H	-3.702138	3.617506	-1.458884
C	-3.589358	-3.308508	1.701474	H	-1.398414	2.843844	-0.935062
C	-3.861208	-2.072214	1.142544	H	1.617605	3.307636	-0.40579
C	-4.397343	0.496532	-0.281863	H	3.374732	4.966162	0.126481
C	-4.605533	1.76536	-0.811526	H	5.315531	4.323791	1.536199
C	-3.529082	2.631372	-1.044468	H	5.465643	2.005448	2.421899
C	-2.251343	2.202269	-0.751766	H	3.692928	0.365976	1.914748
C	2.555018	1.726664	0.69788	H	6.389306	-2.204835	-2.160858
C	2.465695	3.029121	0.20609	H	5.400325	-0.725599	-2.262635
C	3.455911	3.955238	0.514274	H	5.810435	-1.420612	-0.667887

Table S7. Cartesian coordinates of the optimized structure for **3ab** in S_0 calculated at the CAM-B3LYP/6-31G(d) level of theory including CH_2Cl_2 as a solvent using the CPCM.

atom	X	Y	Z	atom	X	Y	Z
C	-4.178933	-1.698369	-0.660177	C	0.775099	0.954978	-1.304729
C	-3.659255	-2.881656	-0.118086	C	2.880241	1.520145	-2.318643
C	-2.369243	-2.921564	0.374261	C	2.247197	2.434521	-3.14136
C	-1.565313	-1.776625	0.351528	C	0.869531	2.62201	-3.033145
C	-2.111314	-0.600106	-0.161061	C	0.133169	1.889893	-2.118707
C	-3.396249	-0.54552	-0.688501	C	6.189306	-1.321672	0.621803
N	-0.223227	-1.84335	0.738589	N	7.340749	-1.401256	0.700169
C	0.545822	-0.918299	0.270892	H	-4.290117	-3.763734	-0.106926
N	0.045585	0.198493	-0.380263	H	-1.95557	-3.842475	0.771454
N	-1.275867	0.562901	-0.081869	H	-3.7628	0.390342	-1.090328
C	2.583539	-1.952187	1.211408	H	1.928474	-2.584711	1.797663
C	2.007208	-1.012969	0.350374	H	4.406092	-2.784475	1.980887
C	3.953264	-2.064248	1.3098	H	4.852525	0.334488	-0.923211
C	4.759904	-1.225636	0.526785	H	-3.465653	0.897488	1.435922
C	4.19589	-0.299178	-0.341489	H	-3.706173	2.396637	3.35689
C	2.805997	-0.180277	-0.452904	H	-1.785641	3.771017	4.138109
C	-1.396547	1.393218	1.085285	H	0.388085	3.600569	2.941522
C	-2.617641	1.488701	1.7567	H	0.637949	2.112181	1.018611
C	-2.749658	2.340628	2.846429	H	-7.030021	-0.879584	-2.00133
C	-1.67727	3.108451	3.285885	H	-6.081628	0.207512	-0.953723
C	-0.464046	3.012311	2.615399	H	-5.454832	-0.259647	-2.561091
C	-0.318655	2.166201	1.521913	H	3.950577	1.382266	-2.417177
O	-5.451205	-1.769962	-1.126945	H	2.823628	3.002056	-3.863645
C	-6.023419	-0.602194	-1.689576	H	0.36465	3.340567	-3.670496
C	2.167445	0.769511	-1.374927	H	-0.937011	2.021287	-2.024911

Table S8. Cartesian coordinates of the optimized structure for **3ca** in S_0 calculated at the CAM-B3LYP/6-31G(d) level of theory including CH_2Cl_2 as a solvent using the CPCM.

atom	X	Y	Z	atom	X	Y	Z
N	-1.792465	1.648332	0.34267	H	-3.649919	1.987813	1.994448
N	-0.521119	1.726559	-0.234457	C	-7.733618	-0.115533	2.021823
N	-1.945014	-0.410023	-0.768483	C	-2.783409	-1.261888	-1.502007
N	-8.802364	-0.274217	2.435414	C	-4.14999	-1.33925	-1.175845
N	3.57263	-0.950002	0.197457	C	-4.974315	-2.158433	-1.955996
C	-2.474815	0.612226	0.024916	H	-6.034779	-2.213083	-1.739155
C	0.204871	0.514424	-0.286283	C	-4.472472	-2.893056	-3.015836
C	-0.548973	-0.62668	-0.575049	H	-5.133914	-3.521148	-3.602409
C	0.05406	-1.8756	-0.539593	C	-3.118249	-2.802706	-3.330208
H	-0.533938	-2.766816	-0.72302	H	-2.712827	-3.356199	-4.170903
C	1.420671	-1.989389	-0.314967	C	-2.281416	-1.988913	-2.586594
H	1.891219	-2.964675	-0.347162	H	-1.236011	-1.897497	-2.853586
C	2.183829	-0.853567	-0.04135	C	4.449911	0.049707	-0.297123

C	1.555348	0.39515	0.010424	C	5.482643	0.542746	0.504624
H	2.132084	1.272428	0.270054	H	5.605404	0.159526	1.511883
C	0.12057	2.979797	-0.079365	C	6.345416	1.5157	0.015604
C	1.141855	3.333489	-0.966571	H	7.143294	1.887138	0.651308
H	1.437748	2.642437	-1.748703	C	6.181455	2.0249	-1.268822
C	1.770254	4.565979	-0.851728	H	6.852155	2.790463	-1.645052
H	2.564935	4.822989	-1.545241	C	5.148654	1.540983	-2.066252
C	1.376161	5.471712	0.129066	H	5.013538	1.92233	-3.073758
H	1.862777	6.438112	0.211144	C	4.294219	0.553772	-1.591686
C	0.346496	5.125696	0.99698	H	3.501363	0.167866	-2.223457
H	0.025479	5.822875	1.765026	C	4.097075	-2.044247	0.933118
C	-0.279079	3.887582	0.902042	C	5.259958	-2.689724	0.504571
H	-1.081243	3.619808	1.57711	H	5.753769	-2.353514	-0.400779
C	-3.84259	0.43794	0.529374	C	5.780126	-3.752023	1.233117
C	-4.6621	-0.54528	-0.047808	H	6.685319	-4.241787	0.887472
C	-5.949404	-0.716477	0.469167	C	5.141099	-4.198332	2.385765
H	-6.605999	-1.479934	0.072553	H	5.545695	-5.033084	2.94881
C	-6.407212	0.083806	1.510217	C	3.977955	-3.562481	2.809153
C	-5.5883	1.074951	2.066812	H	3.472305	-3.895108	3.710473
H	-5.953981	1.692286	2.878807	C	3.462286	-2.487259	2.096629
C	-4.310626	1.239854	1.574179	H	2.563772	-1.98521	2.43894

Table S9. Cartesian coordinates of the optimized structure for **3cb** in S_0 calculated at the CAM-B3LYP/6-31G(d) level of theory including CH_2Cl_2 as a solvent using the CPCM.

atom	X	Y	Z	atom	X	Y	Z
C	2.627206	-0.736557	-0.486957	H	-5.958362	-2.451163	-2.744431
C	2.220317	-1.670031	-1.448813	H	-6.509265	-0.85935	1.195481
C	0.887142	-1.783428	-1.807478	H	1.275374	2.429964	-0.867931
C	-0.07404	-0.943872	-1.241695	H	1.014851	4.601855	-1.962197
C	0.349619	0.013365	-0.313435	H	-1.239872	5.615843	-2.24426
C	1.669376	0.098891	0.094419	H	-3.230769	4.387423	-1.400755
N	-1.429364	-1.12307	-1.526654	H	-2.984122	2.213453	-0.311661
C	-2.259023	-0.64262	-0.660922	H	-5.606446	-0.281589	2.91893
N	-1.854819	0.192992	0.36641	H	-4.57386	0.84917	4.826889
N	-0.66204	0.905765	0.173943	H	-2.244056	1.709148	4.649324
C	-4.209419	-1.626209	-1.81369	H	-0.972316	1.40164	2.545675
C	-3.682369	-0.995874	-0.681655	N	3.987206	-0.625854	-0.113537
C	-5.542501	-1.969286	-1.8675	C	4.764165	-1.790284	0.104021
C	-6.361081	-1.682721	-0.765084	C	6.093896	-1.845458	-0.324572
C	-5.843554	-1.069148	0.368599	C	4.2131	-2.901045	0.750077
C	-4.489872	-0.719663	0.43548	C	6.855207	-2.985526	-0.101219
C	-0.836086	2.155173	-0.516517	H	6.527275	-0.991228	-0.833187
C	0.283595	2.848218	-0.984062	C	4.976044	-4.04406	0.952261
C	0.132062	4.082159	-1.602683	H	3.18495	-2.864306	1.093402
C	-1.127257	4.650977	-1.761201	C	6.301955	-4.093494	0.533288
C	-2.237563	3.963889	-1.288268	H	7.886469	-3.010225	-0.439892
C	-2.100331	2.72632	-0.668002	H	4.531176	-4.897485	1.454817
C	-3.896893	-0.0883	1.622104	H	6.897397	-4.985281	0.699435
C	-2.572618	0.379402	1.554164	C	4.566431	0.659494	0.044475
C	-4.592763	0.089552	2.82485	C	5.389956	0.937966	1.138295
C	-4.012253	0.725093	3.907503	C	4.315617	1.666408	-0.892424
C	-2.706719	1.205161	3.807099	C	5.954748	2.198719	1.285698
C	-1.987366	1.037861	2.637249	H	5.584374	0.162168	1.871028
C	-7.754786	-2.025897	-0.80518	C	4.868115	2.930486	-0.727333
N	-8.877217	-2.303635	-0.840142	H	3.689997	1.451014	-1.752372
H	2.96058	-2.315891	-1.907649	C	5.693547	3.203588	0.359235
H	0.570098	-2.52341	-2.534733	H	6.592901	2.39901	2.140871
H	1.956239	0.824372	0.847074	H	4.662942	3.70159	-1.463553
H	-3.545449	-1.835048	-2.643289	H	6.129923	4.189442	0.482093

Table S10. Cartesian coordinates of the optimized structure for **3da** in S_0 calculated at the CAM-B3LYP/6-31G(d) level of theory including CH_2Cl_2 as a solvent using the CPCM.

atom	X	Y	Z	atom	X	Y	Z
N	1.886199	1.339619	-1.10282	H	6.005823	0.135088	-3.391614
N	1.93439	-0.108713	0.746884	C	4.35979	0.337923	-2.029138
N	-3.558469	-0.895544	-0.108164	H	3.734387	0.890744	-2.718761
N	8.743775	-1.654218	-2.176632	C	7.692787	-1.28112	-1.869262
C	2.512989	0.486074	-0.379297	C	2.741442	-0.632037	1.773318
C	0.532413	-0.336265	0.642389	C	4.095942	-0.910023	1.519791
C	-0.10594	-1.4688	1.12407	C	4.886704	-1.381805	2.574275
H	0.448325	-2.208585	1.688814	H	5.939598	-1.576861	2.407972
C	-1.472223	-1.64624	0.919914	C	4.361328	-1.587038	3.837481
C	-2.161649	-0.714755	0.131849	H	4.996311	-1.95457	4.636088
C	-1.50924	0.387857	-0.446848	C	3.01983	-1.296958	4.076626
C	-0.166573	0.605347	-0.113362	H	2.598629	-1.429433	5.067726
C	-2.174886	-2.831169	1.525136	C	2.218022	-0.814156	3.057055
H	-2.41643	-3.584794	0.768251	H	1.184532	-0.558494	3.254695
H	-3.118976	-2.533458	1.990177	C	-3.985957	-1.974999	-0.913464
H	-1.548704	-3.304866	2.284595	C	-5.204796	-2.621855	-0.673961
C	-2.227012	1.276088	-1.42234	H	-5.83989	-2.286634	0.138141
H	-1.521105	1.717287	-2.128706	C	-5.600148	-3.691094	-1.465871
H	-2.75318	2.098757	-0.927258	H	-6.54995	-4.176623	-1.26283
H	-2.968607	0.699243	-1.978261	C	-4.786056	-4.15387	-2.496009
C	0.284309	3.025898	-0.567655	H	-5.096584	-4.994439	-3.107819
C	-0.725625	3.485011	0.287584	C	-3.567358	-3.525559	-2.725223
H	-1.256066	2.784708	0.923195	H	-2.917239	-3.871164	-3.523289
C	-1.038266	4.837518	0.336645	C	-3.170239	-2.442802	-1.949142
H	-1.824295	5.17114	1.007623	H	-2.22072	-1.956372	-2.143887
C	-0.350516	5.759009	-0.445742	C	-4.462469	0.041264	0.444803
H	-0.596624	6.814712	-0.400469	C	-4.180803	0.636552	1.679108
C	0.665489	5.302946	-1.280754	H	-3.283117	0.352304	2.217298
H	1.220374	6.006149	-1.894958	C	-5.042438	1.585016	2.217477
C	0.987813	3.954393	-1.346394	H	-4.803494	2.033532	3.177026
H	1.784144	3.609247	-1.992973	C	-6.20495	1.948617	1.546928
C	3.864048	0.047966	-0.754724	H	-6.879384	2.684407	1.972461
C	4.635194	-0.668359	0.173524	C	-6.488791	1.357473	0.3192
C	5.902773	-1.106529	-0.220226	H	-7.385595	1.637267	-0.225322
H	6.520436	-1.685776	0.45382	C	-5.625478	0.421976	-0.235088
C	6.388261	-0.816342	-1.490375	H	-5.849446	-0.013983	-1.202059

Table S11. Cartesian coordinates of the optimized structure for **3db** in S_0 calculated at the CAM-B3LYP/6-31G(d) level of theory including CH_2Cl_2 as a solvent using the CPCM.

atom	X	Y	Z	atom	X	Y	Z
N	-0.890766	0.71426	0.71394	H	-1.649619	5.941175	1.263175
N	-1.689915	-0.290676	-1.687692	C	1.472396	0.194625	0.195042
N	-2.068387	-0.050893	0.624942	H	-6.158768	-1.255418	-3.366758
C	-0.329219	-0.302556	-1.372419	N	-9.249095	-0.476707	-1.955792
C	0.594694	-0.814367	-2.280162	C	-0.445731	4.397757	0.365962
H	2.796163	0.148472	1.884583	C	-1.496637	4.874187	1.1396
C	-1.101617	2.119986	0.819824	H	0.227628	5.092581	-0.126369
C	-2.228882	-1.069508	1.607694	N	3.776724	-0.292229	-0.445238
C	-2.513391	-0.229319	-0.703753	C	4.391461	-3.808057	0.565778
C	-6.704361	-0.384104	-1.47178	H	5.806532	0.372826	1.183485
C	-4.439732	-0.804844	-2.16274	C	4.442509	-1.517611	-0.216302
C	2.940174	-1.347313	-2.993492	H	3.83858	-4.624761	1.019988
C	-3.971806	-0.288729	-0.950089	C	5.69317	3.46369	-0.21985
C	-4.884792	0.17893	-0.002549	C	5.777152	-1.702728	-0.597881
C	0.119052	0.206617	-0.149057	C	3.756135	-2.587735	0.367007
C	-2.743085	-2.322196	1.274324	H	2.72089	-2.460993	0.664152
C	-2.351212	3.963103	1.754017	C	6.407773	-2.919699	-0.378853
H	0.566654	2.679055	-0.425214	C	5.72178	-3.982809	0.202001
C	-0.247517	3.030939	0.197169	H	6.317726	-0.890761	-1.070863
H	-1.76687	-1.545279	4.932198	C	4.438304	0.955081	-0.374937
C	-5.79643	-0.853366	-2.427545	C	6.108925	2.413262	0.593473
H	-2.720699	-3.765618	4.351727	H	6.179341	4.431862	-0.160084

C	-2.929261	-3.280395	2.26542	C	4.023659	2.012723	-1.191711
H	-1.462915	0.172336	3.185904	C	5.485344	1.174687	0.528441
C	-8.113191	-0.435174	-1.739523	H	-2.818507	1.896339	2.097774
C	-6.246034	0.133737	-0.258062	C	4.643095	3.25418	-1.106585
H	-6.953642	0.501514	0.476017	H	3.214072	1.858804	-1.896881
C	-2.155662	2.597275	1.604305	H	6.919335	2.56053	1.301018
H	-3.177088	4.316072	2.363865	H	4.304258	4.059969	-1.750711
C	-2.58274	-3.011614	3.583887	H	7.443593	-3.040565	-0.681419
H	0.233741	-1.195427	-3.230049	C	1.926094	0.70581	1.532198
C	-1.876401	-0.796453	2.930848	H	2.212316	1.762374	1.482764
H	-3.723443	-1.16505	-2.891085	H	1.12174	0.613647	2.264828
C	-2.047969	-1.767556	3.907589	H	3.782771	-0.660783	-3.116526
H	-3.332292	-4.2509	1.993768	H	3.357177	-2.309933	-2.67982
C	1.951601	-0.820632	-1.988742	H	6.217215	-4.93433	0.36467
H	2.461991	-1.487509	-3.965566	H	-4.527231	0.590942	0.932898
C	2.380211	-0.309451	-0.7482	H	-2.987751	-2.559714	0.245317

Table S12. Cartesian coordinates of the optimized structure for **3db** in S_1 calculated at the CAM-B3LYP/6-31G(d) level of theory including CH_2Cl_2 as a solvent using the CPCM.

atom	X	Y	Z	atom	X	Y	Z
N	-0.912914	0.486667	0.700075	H	-1.799625	5.698265	1.226279
N	-1.60769	-0.937136	-1.53775	C	1.457833	0.151346	0.172501
N	-2.091985	-0.294527	0.671156	H	-5.985717	-1.08682	-3.623502
C	-0.318063	-0.802016	-1.24517	N	-9.096372	-0.010818	-2.446087
C	0.682642	-1.397522	-2.064215	C	-0.646735	4.177918	0.22901
H	2.799705	0.428292	1.82728	C	-1.617682	4.634938	1.111051
C	-1.146635	1.891198	0.816039	H	-0.067059	4.883268	-0.358292
C	-2.306329	-1.143919	1.779263	N	3.7841	-0.195412	-0.464944
C	-2.540231	-0.560661	-0.630877	C	5.038003	-3.543804	0.500423
C	-6.616845	-0.220673	-1.74871	H	5.676057	0.719079	1.20477
C	-4.350245	-0.874799	-2.267052	C	4.6576	-1.298259	-0.300707
C	3.025931	-1.769531	-2.801936	H	4.673121	-4.426757	1.01536
C	-3.910723	-0.449858	-0.983586	C	5.174464	3.771894	-0.198976
C	-4.880584	0.088418	-0.096427	C	5.962342	-1.266825	-0.805894
C	0.117636	-0.02433	-0.119622	C	4.202332	-2.443465	0.359251
C	-3.151167	-2.255362	1.668654	H	3.196198	-2.462425	0.763904
C	-2.35695	3.709695	1.842844	C	6.792978	-2.365668	-0.646111
H	0.334067	2.475051	-0.635295	C	6.336606	-3.511091	0.002795
C	-0.41232	2.815602	0.072394	H	6.314831	-0.386843	-1.331938
H	-1.448452	-1.462387	5.046723	C	4.270743	1.132674	-0.374638
C	-5.66821	-0.757481	-2.639685	C	5.716097	2.786549	0.623544
H	-2.960217	-3.427411	4.854916	H	5.525685	4.796006	-0.129298
C	-3.379697	-3.06448	2.772397	C	3.734196	2.119423	-1.207549
H	-1.031601	-0.018125	3.099956	C	5.266848	1.476701	0.546007
C	-7.982599	-0.105256	-2.132794	H	-2.701482	1.639223	2.285553
C	-6.197964	0.200375	-0.470912	C	4.180961	3.430873	-1.111093
H	-6.923013	0.623177	0.216419	H	2.977141	1.849906	-1.936007
C	-2.124207	2.348638	1.704459	H	6.486625	3.042217	1.343873
H	-3.122078	4.047875	2.534612	H	3.75788	4.186629	-1.765122
C	-2.777545	-2.789475	3.9967	H	7.801718	-2.331973	-1.045185
H	0.34788	-1.9645	-2.926439	C	1.872432	0.850556	1.436509
C	-1.694965	-0.868628	3.008049	H	2.034235	1.922522	1.283856
H	-3.624051	-1.300815	-2.948216	H	1.093177	0.740401	2.193125
C	-1.932967	-1.68927	4.102212	H	3.795457	-1.032741	-3.048621
H	-4.031989	-3.925774	2.667567	H	3.538144	-2.647349	-2.397354
C	2.018587	-1.207459	-1.833628	H	6.989283	-4.369853	0.119298
H	2.527706	-2.067481	-3.726918	H	-4.571647	0.436397	0.882178
C	2.419147	-0.414825	-0.709593	H	-3.616263	-2.491325	0.719823

Table S13. Cartesian coordinates of the optimized structure for **4ca** in S_0 calculated at the CAM-B3LYP/6-31G(d) level of theory including CH_2Cl_2 as a solvent using the CPCM.

atom	X	Y	Z	atom	X	Y	Z
N	1.190114	-1.025498	-0.541326	C	-0.017264	3.657571	-0.274273
N	1.981551	-0.057524	0.08917	C	-0.534813	4.662532	-1.082257
C	-0.070162	-0.95609	-0.301221	C	-1.473469	4.370237	-2.066359
N	-0.5954	0.100848	0.409415	C	-1.888726	3.055497	-2.242144
N	0.111117	1.30343	0.373844	C	-1.366489	2.036786	-1.453039
C	1.514325	1.167843	0.486254	O	2.213983	2.087908	0.864287
C	-1.005863	-1.992803	-0.738435	H	0.389879	-2.876102	-2.102203
C	-2.291117	-2.032302	-0.169223	H	-1.189235	-4.644752	-2.848551
C	-2.663117	-1.045362	0.858488	H	-3.479448	-4.721986	-1.882747
C	-1.800016	0.033219	1.121178	H	-4.175475	-3.085433	-0.211159
C	-0.611638	-2.935682	-1.69243	H	-4.528106	-1.948482	1.438621
C	-1.49317	-3.916927	-2.103837	H	-5.114555	-0.230463	3.081218
C	-2.778053	-3.959792	-1.559142	H	-3.579085	1.689029	3.474839
C	-3.169225	-3.033351	-0.608917	H	-1.45443	1.846318	2.215593
C	-3.85188	-1.116336	1.594381	H	4.177635	1.470644	-0.278749
C	-4.185564	-0.149206	2.527392	H	6.516857	0.72223	-0.027065
C	-3.325194	0.924508	2.747944	H	7.018274	-1.619314	0.624252
C	-2.134335	1.020688	2.047771	H	5.149287	-3.209444	1.014258
C	3.350362	-0.443118	0.240273	H	2.806165	-2.449804	0.765507
C	4.393387	0.451091	0.007409	H	0.702554	3.881794	0.501139
C	5.707841	0.021633	0.15413	H	-0.208688	5.686111	-0.926927
C	5.989571	-1.290952	0.515838	H	-1.882901	5.161323	-2.685889
C	4.942925	-2.181534	0.733411	H	-2.622415	2.811312	-3.003766
C	3.626167	-1.761346	0.600122	H	-1.694613	1.015265	-1.604106
C	-0.423689	2.338147	-0.472699				

Table S14. Cartesian coordinates of the optimized structure for **4cb** in S_0 calculated at the CAM-B3LYP/6-31G(d) level of theory including CH_2Cl_2 as a solvent using the CPCM.

atom	X	Y	Z	atom	X	Y	Z
N	-1.106793	0.815964	-0.798912	C	2.168082	-4.397803	-0.956697
N	-1.688301	-0.316213	-0.250704	C	3.264742	-3.901298	-1.65103
C	0.12715	1.004015	-0.518244	C	3.407564	-2.525768	-1.801716
N	0.816966	0.048249	0.265999	C	2.463041	-1.655361	-1.273869
N	0.403885	-1.254667	-0.032365	O	-1.47746	-2.582229	0.071924
C	-0.96616	-1.483207	-0.044956	H	-0.930509	3.128152	-1.780656
C	0.847109	2.185713	-1.0274	H	0.193099	2.2958	1.631822
C	0.144039	3.213046	-1.666726	H	2.781444	1.50544	-0.375481
C	0.695799	1.538358	2.221661	H	3.978009	3.485985	-1.234692
C	1.051278	0.315768	1.653557	H	2.725253	5.298363	-2.374475
C	2.231061	2.294567	-0.874932	H	0.265301	5.11488	-2.644907
C	2.902473	3.411897	-1.357903	H	0.69517	2.740542	3.99259
C	2.200038	4.427022	-1.99659	H	1.807306	1.014959	5.391526
C	0.818629	4.323803	-2.14919	H	2.420182	-1.168452	4.37427
C	0.977982	1.78557	3.561204	H	1.956004	-1.607464	1.996957
C	1.596371	0.81977	4.345367	H	-3.428187	-2.16135	-1.207889
C	1.938029	-0.402781	3.774748	H	-5.893368	-1.984319	-1.136819
C	1.67728	-0.656496	2.434931	H	-6.97418	0.001125	-0.11653
C	-3.11857	-0.281223	-0.216899	H	-5.566813	1.815498	0.831248
C	-3.899881	-1.297429	-0.761229	H	-3.096727	1.621234	0.772472
C	-5.285579	-1.190606	-0.714175	H	0.373627	-3.925169	0.138204
C	-5.892032	-0.075917	-0.145082	H	2.047299	-5.468202	-0.822458
C	-5.104261	0.940911	0.385262	H	4.004117	-4.57815	-2.066441
C	-3.719664	0.837508	0.355745	H	4.258045	-2.120543	-2.340861
C	1.363401	-2.16104	-0.580593	H	2.574666	-0.586029	-1.400834
C	1.220355	-3.538653	-0.41034				

Table S15. Cartesian coordinates of the optimized structure for **phenanthrene dianion** in S_0 calculated at the CAM-B3LYP/6-31G(d) level of theory.

atom	X	Y	Z	atom	X	Y	Z
C	-3.569451	-0.353965	-0.050811	C	3.569453	-0.353964	0.05082
C	-2.907533	-1.552198	0.234363	C	2.890937	0.835488	0.225841
C	-1.482252	-1.507099	0.267818	H	-4.662479	-0.350185	-0.138114
C	-0.745878	-0.363423	0.050817	H	-3.446134	-2.485932	0.389535
C	-1.443221	0.926094	-0.089405	H	-0.940208	-2.434672	0.459376
C	-2.890939	0.835486	-0.225831	H	-3.443746	1.753548	-0.438121
C	0.745878	-0.363419	-0.050829	H	1.253981	3.045856	0.167789
C	1.443219	0.926093	0.0894	H	-1.253983	3.045856	-0.167793
C	0.724287	2.093666	0.075737	H	0.940211	-2.434671	-0.459383
C	-0.724289	2.093665	-0.075743	H	3.446137	-2.485931	-0.38953
C	1.482254	-1.507099	-0.267827	H	4.66248	-0.350184	0.138132
C	2.907534	-1.552197	-0.234357	H	3.443741	1.753548	0.438142

Table S16. Cartesian coordinates of the optimized structure for **anthracene dianion** in S_0 calculated at the CAM-B3LYP/6-31G(d) level of theory.

atom	X	Y	Z	atom	X	Y	Z
C	-3.730383	-0.689268	-0.000001	C	3.730383	-0.689268	-0.000001
C	-3.730383	0.689268	-0.000002	C	2.496645	-1.386142	-0.000001
C	-2.496645	1.386142	0	H	-4.669006	-1.249254	-0.000002
C	-1.253255	0.728734	0.000002	H	-4.669006	1.249254	-0.000003
C	-1.253255	-0.728734	0	H	-2.498655	2.478854	0
C	-2.496645	-1.386142	-0.000001	H	-2.498654	-2.478854	0
C	0	1.392822	0.000001	H	0	2.486153	-0.000001
C	1.253255	0.728735	0	H	0	-2.486153	0.000001
C	1.253255	-0.728735	0.000002	H	2.498655	2.478854	0.000001
C	0	-1.392822	0.000003	H	4.669006	1.249254	0
C	2.496645	1.386142	0	H	4.669006	-1.249254	-0.000004
C	3.730383	0.689268	-0.000001	H	2.498655	-2.478854	0

Table S17. Cartesian coordinates of the optimized structure for **Da** in S_0 calculated at the CAM-B3LYP/6-31G(d) level of theory.

atom	X	Y	Z	atom	X	Y	Z
C	2.618677	-0.795974	0.096637	C	4.786263	0.180484	0.138999
C	1.852982	-1.95233	0.179177	H	2.349033	-2.903648	0.327415
C	0.468576	-1.863289	0.089005	H	-0.130047	-2.760292	0.199291
C	-0.143423	-0.642972	-0.137042	H	2.595473	1.353311	-0.094453
C	0.619996	0.528933	-0.192565	H	-1.914112	-2.391573	-0.987501
C	2.005313	0.449949	-0.062788	H	-4.35234	-2.314912	-0.516482
N	-1.554263	-0.4785	-0.260578	H	-5.279378	-0.278967	0.673833
C	-2.061881	0.762008	0.177148	H	-3.803217	1.672478	1.073863
N	-1.351324	1.830093	0.197422	H	5.811216	-0.178974	0.233531
N	-0.07026	1.725419	-0.39059	H	4.676055	0.708768	-0.815873
C	-2.390834	-1.533385	-0.530721	H	4.570834	0.874853	0.960261
C	-3.711469	-1.481741	-0.259878	C	0.652633	2.970423	-0.270217
C	-4.234982	-0.304822	0.379729	H	1.107337	3.106392	0.722999
C	-3.443796	0.762918	0.608902	H	1.435548	3.018098	-1.031462
O	3.96412	-0.962978	0.197101	H	-0.056745	3.777986	-0.444318

Table S18. Cartesian coordinates of the optimized structure for **Db** in S_0 calculated at the CAM-B3LYP/6-31G(d) level of theory.

atom	X	Y	Z	atom	X	Y	Z
C	2.885402	-0.36697	-0.167081	C	-2.614233	1.36772	-0.483858
C	2.608026	-1.73008	-0.170118	C	-0.319767	2.339741	0.805444
C	1.311055	-2.175048	0.042598	H	3.422747	-2.424058	-0.342214
C	0.272292	-1.279626	0.275745	H	1.076952	-3.233917	0.033892
C	0.574941	0.087547	0.292597	H	2.063722	1.619132	0.063989
C	1.860484	0.557265	0.058746	H	-3.611937	-2.266696	0.27844

N	-1.04087	-1.735051	0.432704	H	-5.298871	-0.665646	-0.58741
C	-1.980583	-0.875152	0.21656	H	5.583055	1.407716	-0.589934
N	-1.720642	0.498513	0.060809	H	3.975479	1.889533	-1.190168
N	-0.508959	0.922632	0.666904	H	4.294293	1.828399	0.566903
C	-3.362866	-1.238276	0.048376	H	-4.583733	1.685137	-1.190481
C	-4.270388	-0.354271	-0.434758	H	-2.260208	2.373301	-0.660043
O	4.185122	-0.017076	-0.385999	H	-1.196709	2.779619	1.285618
C	4.511143	1.351439	-0.39758	H	0.534447	2.487074	1.467967
C	-3.886711	0.979581	-0.758603	H	-0.119682	2.871129	-0.139896

Table S19. Cartesian coordinates of the optimized structure for **Ea** in S_0 calculated at the CAM-B3LYP/6-31G(d) level of theory.

atom	X	Y	Z	atom	X	Y	Z
C	0.801596	2.810726	-0.194529	C	3.947717	-2.695248	0.246175
C	-0.502597	3.285747	-0.079487	C	2.993038	-3.114921	-0.67283
C	-1.545536	2.384842	0.076219	C	1.745496	-2.50739	-0.732644
C	-1.288605	1.026122	0.183923	H	-0.681046	4.35272	-0.133719
C	0.016234	0.544988	0.060642	H	-2.565527	2.750244	0.109671
C	1.061208	1.441478	-0.153909	H	2.062864	1.058744	-0.278285
N	-2.310473	0.04172	0.32563	H	-3.649335	1.283017	1.318731
C	-2.03936	-1.200018	-0.280594	H	-5.615045	-0.171663	0.875504
N	-0.843673	-1.62969	-0.467084	H	-5.285572	-2.221661	-0.574836
N	0.17037	-0.847965	0.123704	H	-2.994459	-2.862541	-1.273385
C	-3.576127	0.36271	0.753096	H	3.702443	4.22254	-0.604343
C	-4.635834	-0.431426	0.495509	H	3.436908	2.763297	0.386631
C	-4.431392	-1.612554	-0.297206	H	3.232917	2.690037	-1.38594
C	-3.191677	-1.972213	-0.689566	H	2.132549	-0.279314	1.79565
C	1.437965	-1.465036	0.142093	H	4.3599	-1.337817	1.864797
O	1.76573	3.752118	-0.361425	H	4.921295	-3.172897	0.284864
C	3.102051	3.318971	-0.497435	H	3.220549	-3.924585	-1.359752
C	2.386169	-1.059675	1.086309	H	0.994667	-2.833173	-1.439906
C	3.634235	-1.664163	1.125897				

Table S20. Cartesian coordinates of the optimized structure for **Eb** in S_0 calculated at the CAM-B3LYP/6-31G(d) level of theory.

atom	X	Y	Z	atom	X	Y	Z
C	3.267691	-0.517179	-0.03437	C	-1.699481	3.880817	-0.666823
C	3.141824	-1.253547	-1.218371	C	-0.340552	3.619756	-0.535774
C	1.895632	-1.576642	-1.716836	C	0.106943	2.376666	-0.112409
C	0.731171	-1.161804	-1.058545	H	4.048599	-1.571759	-1.720721
C	0.882633	-0.394513	0.096806	H	1.790824	-2.169118	-2.619075
C	2.126624	-0.087434	0.6358	H	2.172174	0.488971	1.550868
N	-0.520621	-1.590236	-1.498918	H	-2.944052	-2.634178	-1.745238
C	-1.466064	-1.553878	-0.61507	H	-4.507059	-2.904446	0.158841
N	-1.296685	-0.91006	0.620518	H	5.787829	0.554782	1.720015
N	-0.330534	0.105013	0.688163	H	4.27805	1.474325	1.485155
C	-2.734099	-2.218006	-0.767627	H	4.263323	-0.043555	2.426377
C	-3.573993	-2.36481	0.285973	H	-3.894562	-1.981454	2.428329
O	4.540478	-0.265913	0.377587	H	-1.796169	-0.588756	2.596967
C	4.711792	0.469568	1.566596	H	-2.904545	0.855201	0.269128
C	-3.24703	-1.831479	1.574593	H	-3.673199	3.05257	-0.473739
C	-2.117022	-1.098104	1.697015	H	-2.04438	4.853991	-0.999819
C	-0.808959	1.367476	0.18808	H	0.387624	4.390295	-0.769468
C	-2.173414	1.624104	0.055781	H	1.168942	2.186988	-0.0266
C	-2.60742	2.87408	-0.369831				

Table S21. Cartesian coordinates of the optimized structure for **3aa**$^{\bullet+}$ in S_0 calculated at the CAM-UB3LYP/6-31G(d) level of theory including CH_3CN as a solvent using the CPCM.

atom	X	Y	Z	atom	X	Y	Z
C	3.56670252	-1.83890626	-0.87286145	H	5.07242649	-4.09112634	-0.87396456
C	2.56660804	-2.82170011	-0.97790861	C	-3.09404934	-0.12803349	-0.02716653

N	1.50207873	1.03138453	0.18742300	H	3.01533824	5.29231484	-0.49984355
C	-0.69667972	0.45171457	0.04759372	H	3.46760091	1.02317888	1.98063505
H	-1.68408611	-4.80237971	2.14906498	C	-4.41008520	0.24257838	-0.32309281
C	-3.69582440	-2.38498858	0.89267685	C	3.38588728	1.95355929	1.42993178
C	1.91672147	-0.25790616	-0.12528181	C	2.40593619	2.10969084	0.45349645
H	4.76787354	-3.58989662	-2.56709508	C	-4.70239531	1.54656738	-0.69685263
C	3.23166692	-0.55294096	-0.46871514	H	4.77984820	5.03968329	1.22374694
H	0.03276532	-3.20629257	1.43783111	H	-1.59543707	2.88823825	-0.58392982
C	-1.00903514	2.99210946	-1.24249111	C	-3.69369694	2.51880547	-0.78078756
C	0.92161956	-1.25651752	-0.16624866	C	4.24100403	3.01418191	1.70001924
C	1.26248923	-2.52403998	-0.63701278	H	-5.21393785	-0.47936492	-0.26919141
N	0.21858005	1.38491211	0.06482626	C	-2.39302824	2.16060017	-0.50778227
H	1.48378001	3.40110880	-0.99326087	H	5.00451765	2.90257092	2.46205166
H	-4.74808146	-2.14653534	0.80381981	C	3.11850169	4.35806794	0.04134282
H	3.99789450	0.20955387	-0.45459175	C	-1.37985186	-1.79952731	0.61297768
C	-6.06020019	1.90446173	-0.99963636	C	-2.73608550	-1.45707789	0.46923954
N	-0.39192044	-0.88022775	0.18011023	C	-1.98042626	-3.88339729	1.65606575
H	-4.09353371	-4.28863410	1.78302533	H	2.79678205	-3.81300946	-1.34429179
C	-3.32961157	-3.59019891	1.46056079	H	0.50044395	-3.28108927	-0.76001870
C	-2.08705670	0.84190058	-0.14690633	O	4.85763307	-2.03981109	-1.17608801
C	2.25849912	3.30591598	-0.24199408	C	5.26841299	-3.32626349	-1.63094798
C	4.11008200	4.21414191	1.00779296	H	6.34083247	-3.24842884	-1.80103600
H	-3.93840427	3.53423497	-1.06784341	N	-7.15221195	2.19577248	-1.24412607

Table S22. Cartesian coordinates of the optimized structure for **3aa**$^{\bullet-}$ in S_0 calculated at the CAM-UB3LYP/6-31+G(d) level of theory including CH_3CN as a solvent using the CPCM.

atom	X	Y	Z	atom	X	Y	Z
C	3.32721311	-1.94782305	-1.18185725	H	4.70575294	-4.26881200	-1.44410731
C	2.29275848	-2.87657598	-1.12961710	C	-3.07019650	-0.03874565	0.01342311
N	1.57140235	0.99528976	0.31188603	H	3.03303776	5.33388082	0.24448633
C	-0.66654665	0.59713466	0.05252425	H	3.88411116	0.64960326	1.57242579
H	-1.67596657	-4.42145355	2.79051258	C	-4.36002498	0.25450073	-0.36452253
C	-3.67468005	-2.12653448	1.30105846	C	3.70581863	1.67180984	1.25798893
C	1.90852227	-0.28559976	-0.15575818	C	2.52547892	1.99099355	0.56974463
H	4.15094024	-3.71652746	-3.04917633	C	-4.70275666	1.49504776	-0.97180479
C	3.12543190	-0.64542573	-0.71923900	H	5.13843096	4.75175089	1.43215896
H	0.04587587	-2.91613220	1.87228453	H	-1.59765848	2.91260876	-0.95498247
C	-1.00318594	-2.70804913	1.69633871	C	-3.66443726	2.46401163	-1.15528436
C	0.86386922	-1.22224359	-0.09027278	C	4.63649909	2.65705347	1.55484785
C	1.05706600	-2.49406714	-0.60585657	H	-5.15423673	-0.46498919	-0.19749360
N	0.29691511	1.47304783	-0.08672086	C	-2.38157650	2.18044961	-0.79775380
H	1.37656357	3.57878491	-0.29351096	H	5.54212820	2.38427941	2.08871375
H	-4.72661490	-1.89087254	1.18433067	C	3.23166371	4.30468443	0.52998487
H	3.93689029	0.06627048	-0.80157194	C	-1.35933685	-1.58173064	0.94648410
C	-6.02477855	1.76491896	-1.36200784	C	-2.71710225	-1.26913469	0.74409528
N	-0.35660416	-0.73321415	0.44172340	C	-1.97336971	-3.55032878	2.21526818
H	-4.09050752	-3.90554218	2.42495983	H	2.42213490	-3.88780081	-1.49265465
C	-3.32032878	-3.26099649	2.01367297	H	0.24681666	-3.21308954	-0.58586667
C	-2.02056991	0.91197091	-0.23886343	O	4.57013577	-2.20339405	-1.68028343
C	2.29474748	3.32605181	0.21814180	C	4.81844464	-3.49262775	-2.20941978
C	4.41074687	3.98299783	1.19304749	H	5.84991833	-3.48217045	-2.56186426
H	-3.91004348	3.42756552	-1.59212991	N	-7.12526725	1.99049717	-1.68694948

Table S23. Cartesian coordinates of the optimized structure for **2a** in S_0 calculated at the CAM-UB3LYP/6-31+G(d) level of theory including CH_3CN as a solvent using the CPCM.

atom	X	Y	Z	atom	X	Y	Z
N	-0.90248655	0.49869121	0.04804945	H	5.47233512	-2.20276667	0.08554015
N	0.67518859	-1.74837275	0.11349684	H	-5.62142420	-0.66841222	0.59610200
N	0.44901976	0.61850709	0.01684807	C	-2.89702847	4.19993597	0.07302188
C	-0.68892220	-1.86764462	0.07321854	C	6.82950347	0.09709146	-0.01431810
C	-1.28278890	-3.14214305	0.06350831	C	4.58391516	1.08239651	-0.03518509
H	-0.62728908	-4.00460690	0.11450904	H	5.02832649	2.07036793	-0.07702864
C	-1.60162614	1.74602061	0.05237220	C	-1.31536378	2.68418263	-0.93507779

H	2.56702746	1.80701023	-0.04572066	H	-0.58630791	2.44608544	-1.70107733
C	1.15057809	-0.50851913	0.05369218	C	-3.17283903	3.25863901	1.05951533
C	5.40282722	-0.05058590	0.00251298	H	-3.40473581	5.15885738	0.08012625
C	3.45634206	-1.46253667	0.07241131	H	-3.88919042	3.48221259	1.84309814
H	3.00226023	-2.44440429	0.11338575	N	7.98066264	0.21627880	-0.02784206
C	2.63036290	-0.33716143	0.03582275	H	-3.54896309	-0.00284182	-0.16696042
C	3.20734495	0.93451735	-0.01801996	H	-5.49754026	-0.71109046	-1.18759806
C	-1.53451597	-0.73763254	0.01612487	C	-2.64615106	-3.29077760	-0.01875535
H	-6.68909661	-1.73925659	-0.34944854	H	-3.11064538	-4.27019899	-0.02795247
C	-1.96472144	3.91198302	-0.91975801	C	-3.47165947	-2.15429180	-0.10519270
H	-1.74413622	4.64407969	-1.68963154	O	-4.79552436	-2.40606178	-0.20160790
C	-2.52110426	2.03062924	1.05815603	C	-2.92259535	-0.87882724	-0.09157729
H	-2.71596002	1.30178607	1.83728752	C	-5.69066641	-1.30774399	-0.29008569
C	4.83458251	-1.32654142	0.05667458				

10. NMR Spectra

Note: The signals pointing down at 110 ppm are measurement artefacts.

2,6-Dimethyl-4-nitro-*N,N*-diphenylaniline (**S8**)

^{1}H-NMR (400 MHz, $CDCl_3$)

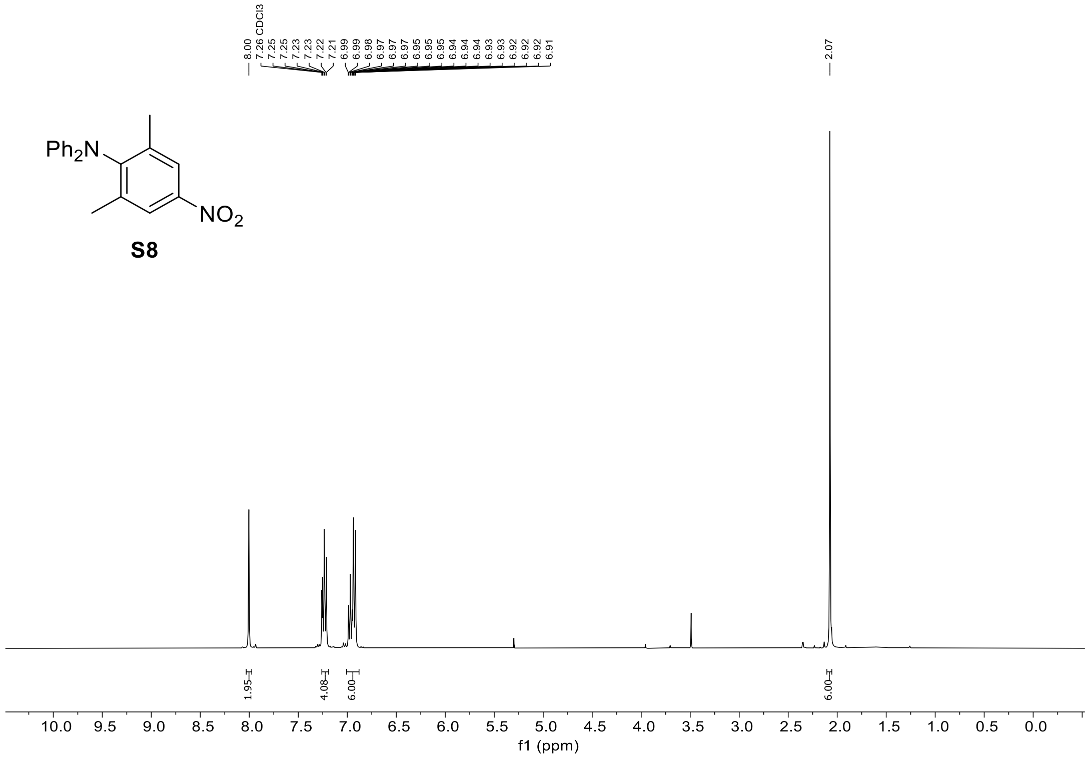

^{13}C-NMR (101 MHz, $CDCl_3$)

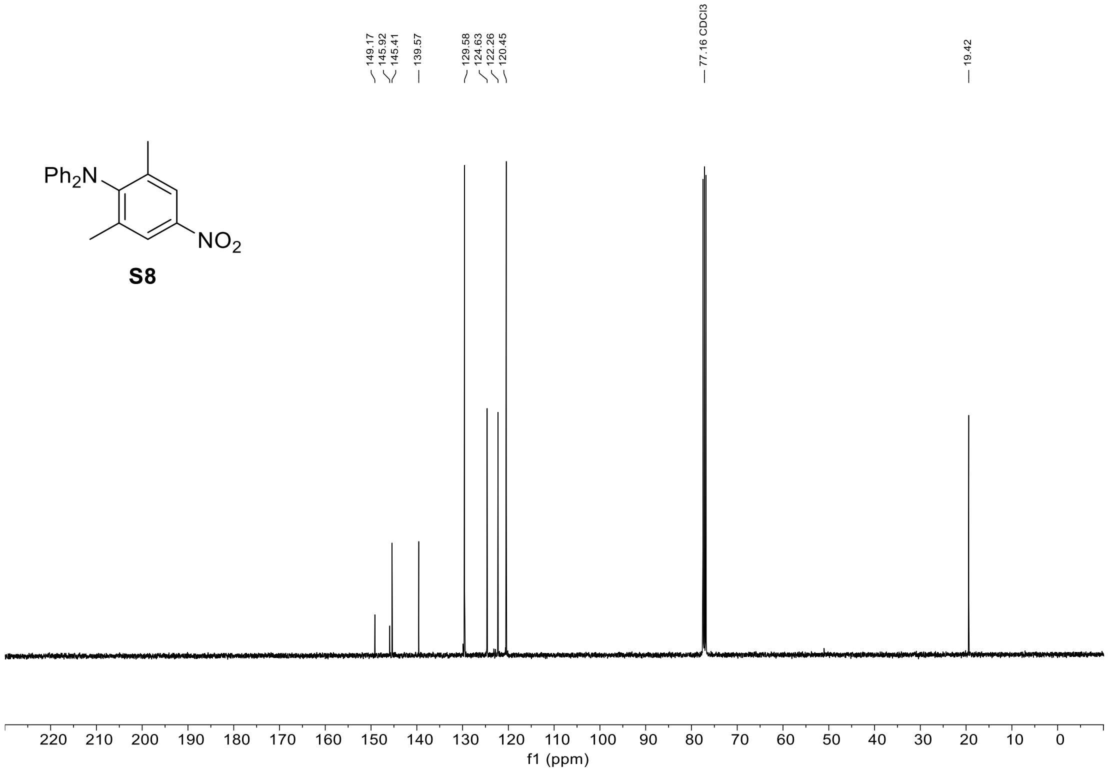

2,6-Dimethyl-N^1,N^1-diphenylbenzene-1,4-diamine (**S9**)

^{1}H-NMR (400 MHz, $CDCl_3$)

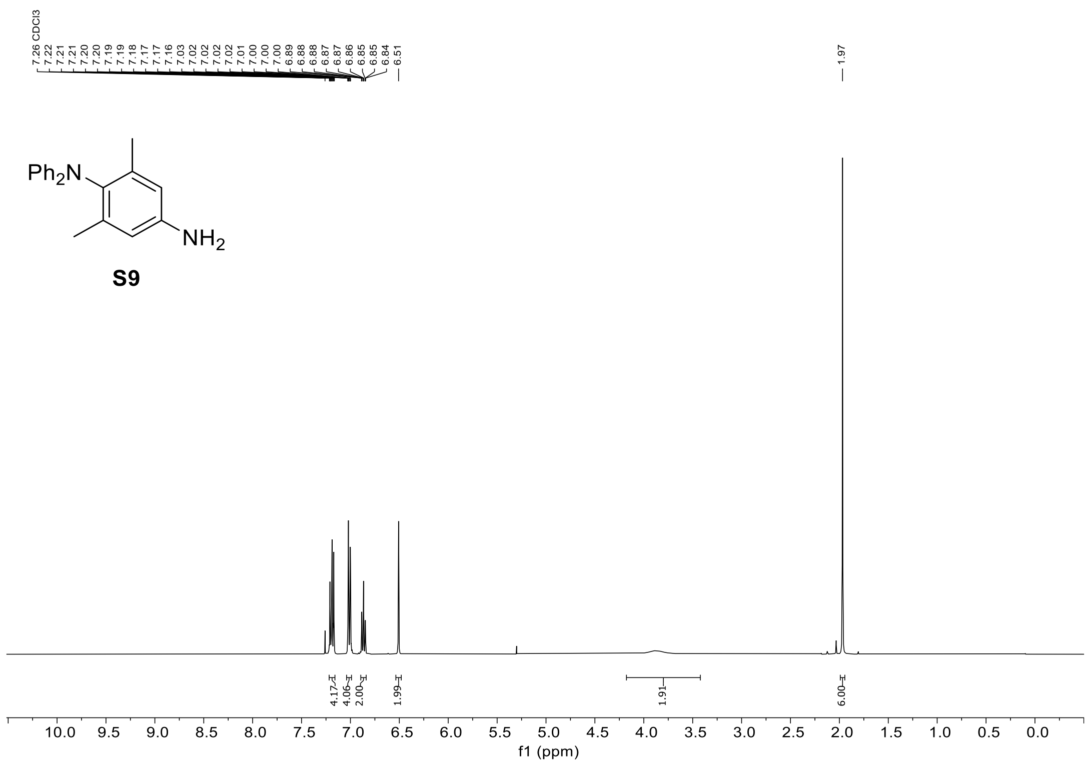

^{13}C-NMR (101 MHz, $CDCl_3$)

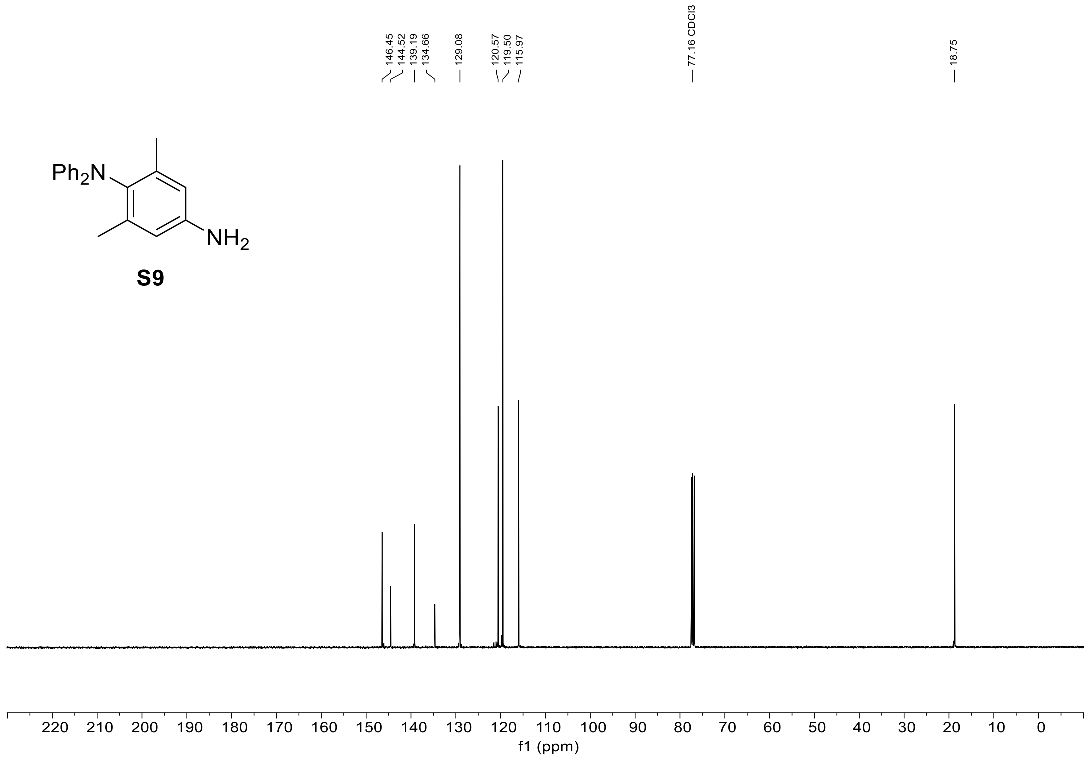

8-Methoxy-6-phenyl-6H-benzo[5,6][1,2,4]triazino[4,3-f]phenanthridine-2-carbonitrile (**3aa**)

^{1}H-NMR (599 MHz, CD_2Cl_2)

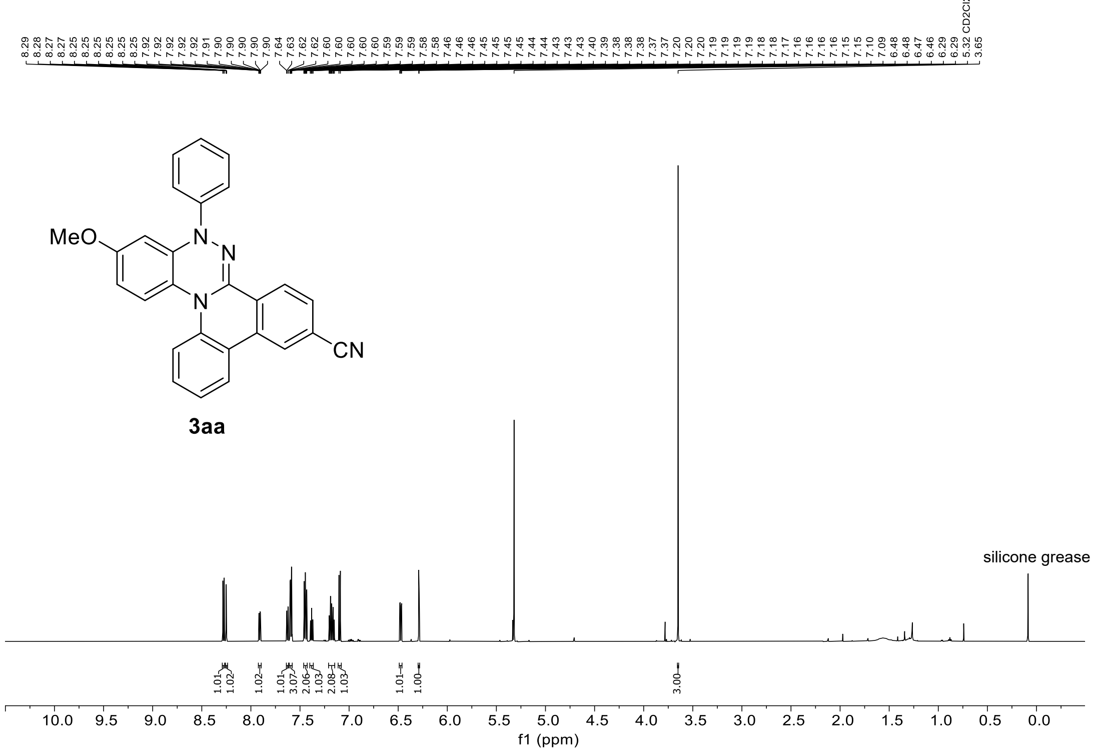

^{13}C-NMR (151 MHz, CD_2Cl_2)

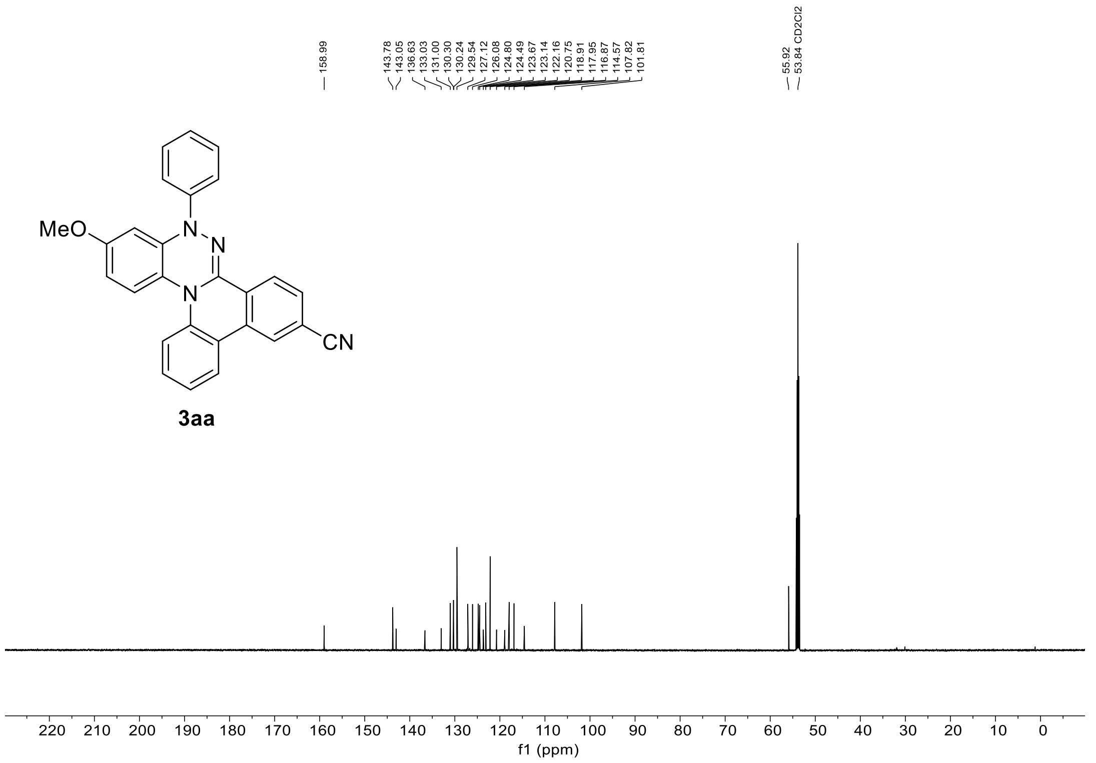

12-Methoxy-10-phenyl-10H-benzo[5,6][1,2,4]triazino[2,3-f]phenanthridine-3-carbonitrile (**3ab**)

^{1}H-NMR (599 MHz, CD_2Cl_2)

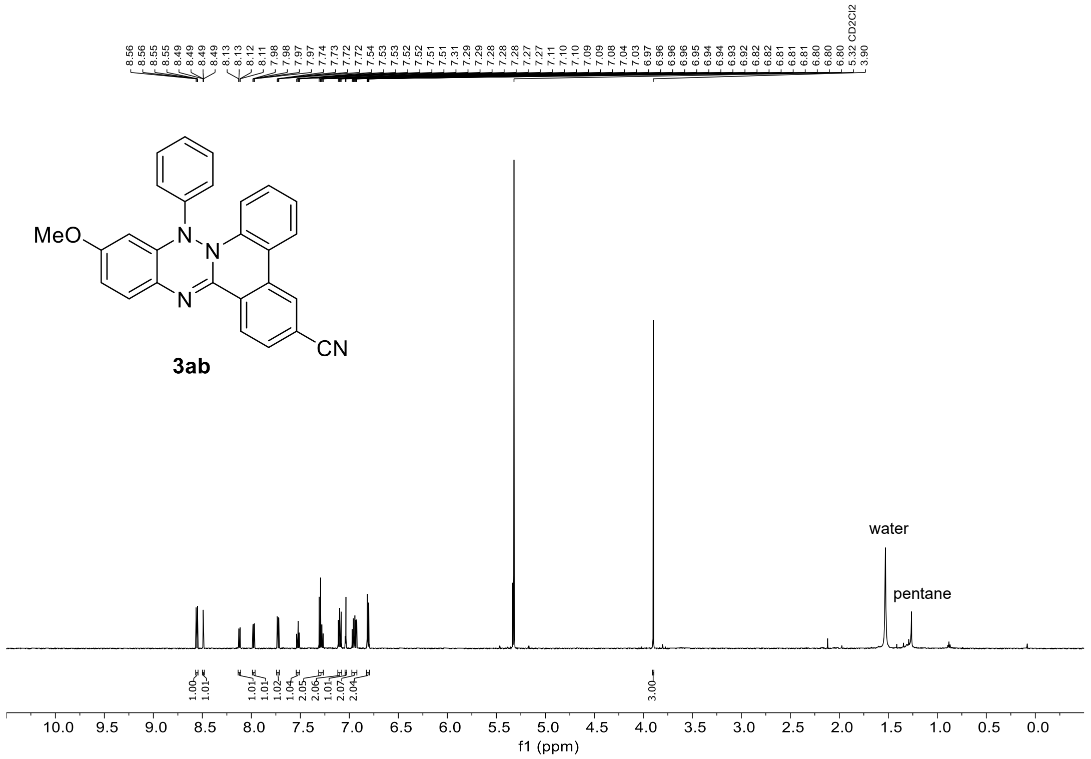

^{13}C-NMR (151 MHz, CD_2Cl_2)

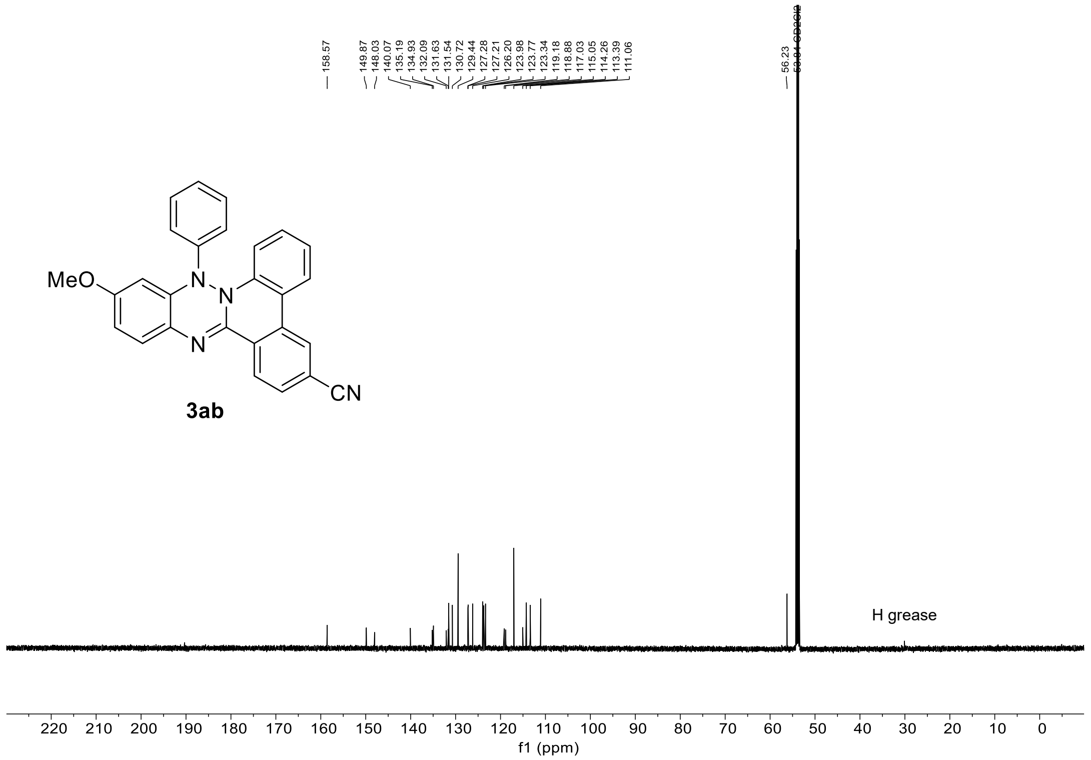

6-Phenyl-6H-benzo[5,6][1,2,4]triazino[4,3-f]phenanthridine (**3ba**)

^{1}H-NMR (599 MHz, CD_2Cl_2)

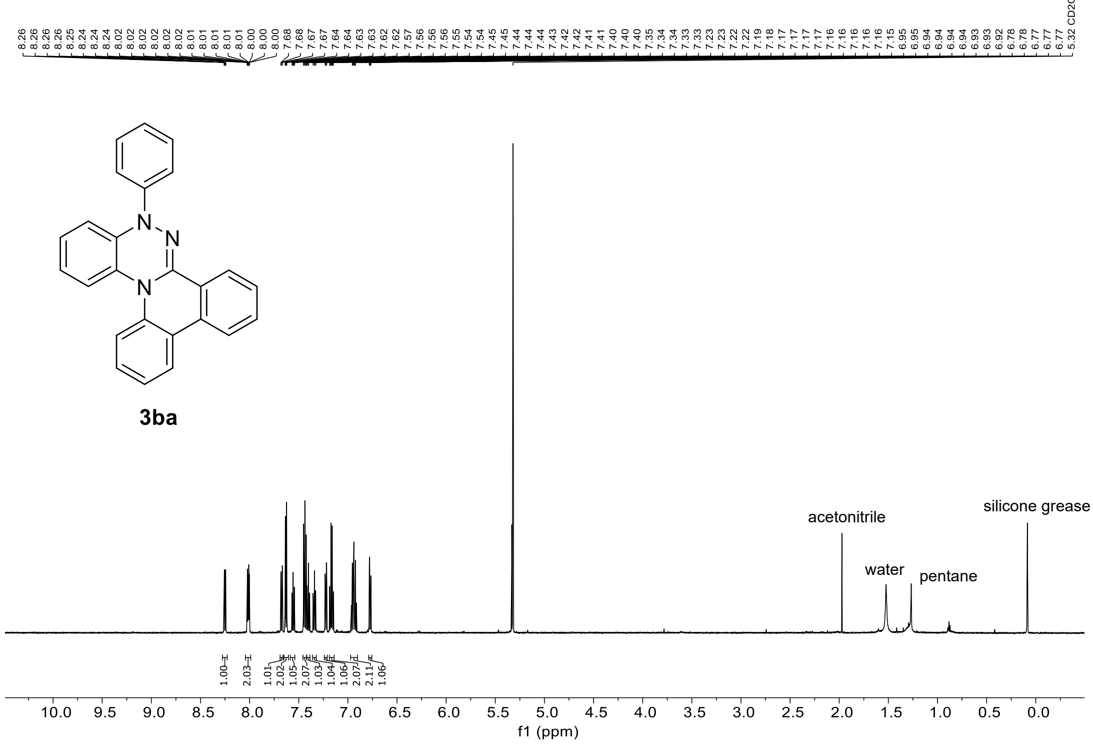

^{13}C-NMR (151 MHz, CD_2Cl_2)

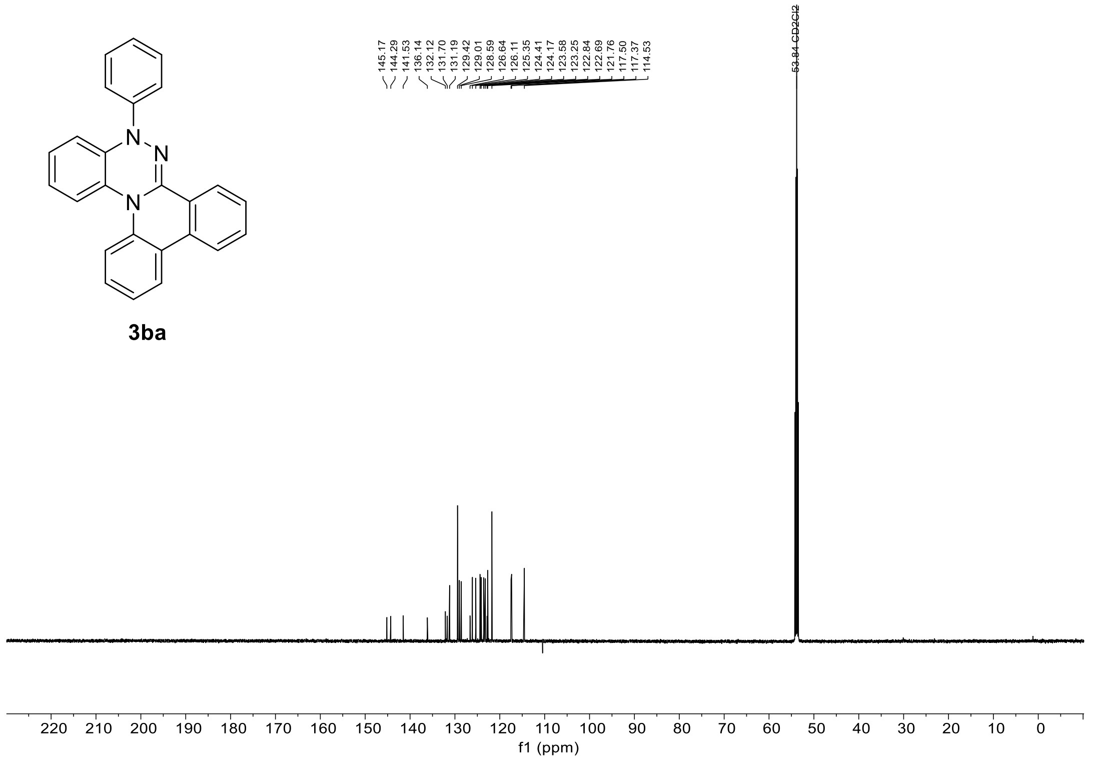

10-Phenyl-10H-benzo[5,6][1,2,4]triazino[2,3-f]phenanthridine (**3bb**)

^{1}H-NMR (599 MHz, CD_2Cl_2)

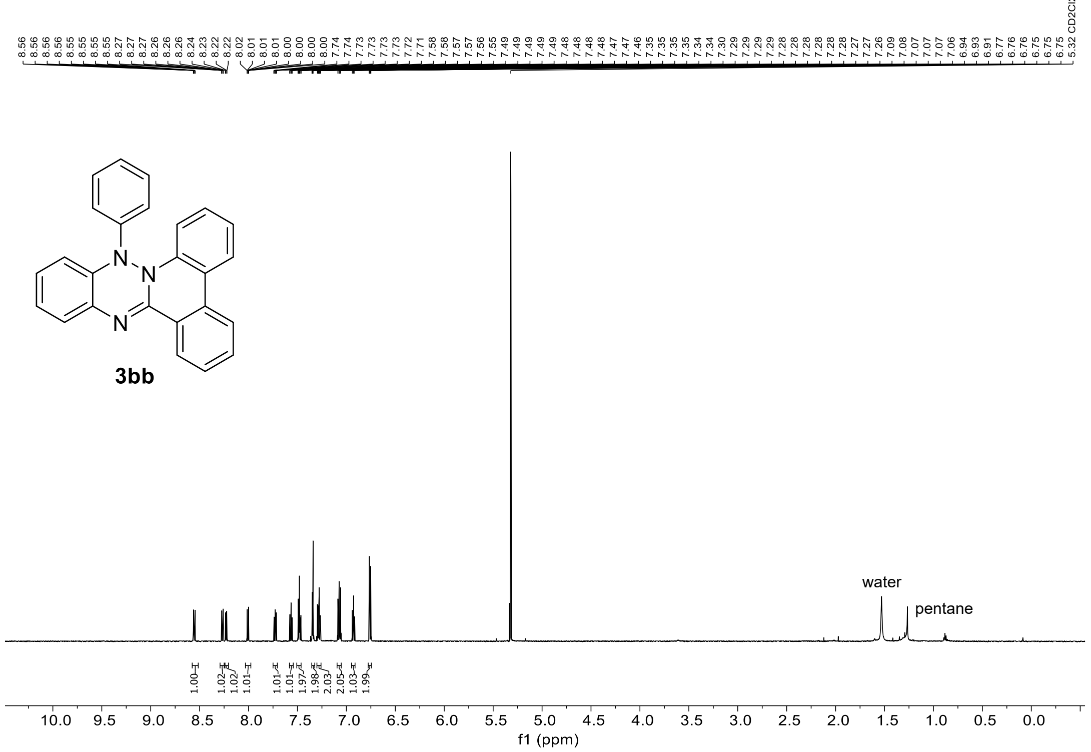

^{13}C-NMR (151 MHz, CD_2Cl_2)

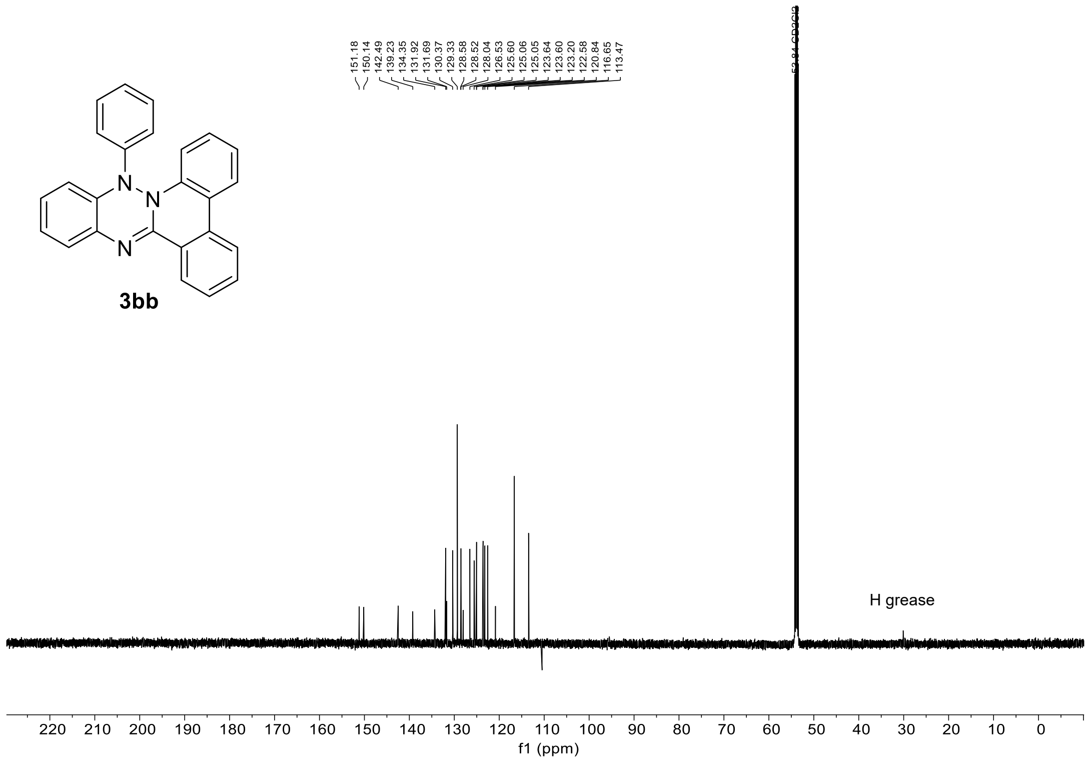

8-(Diphenylamino)-6-phenyl-6H-benzo[5,6][1,2,4]triazino[4,3-f]phenanthridine-2-carbonitrile (**3ca**)

^{1}H-NMR (599 MHz, CD_2Cl_2)

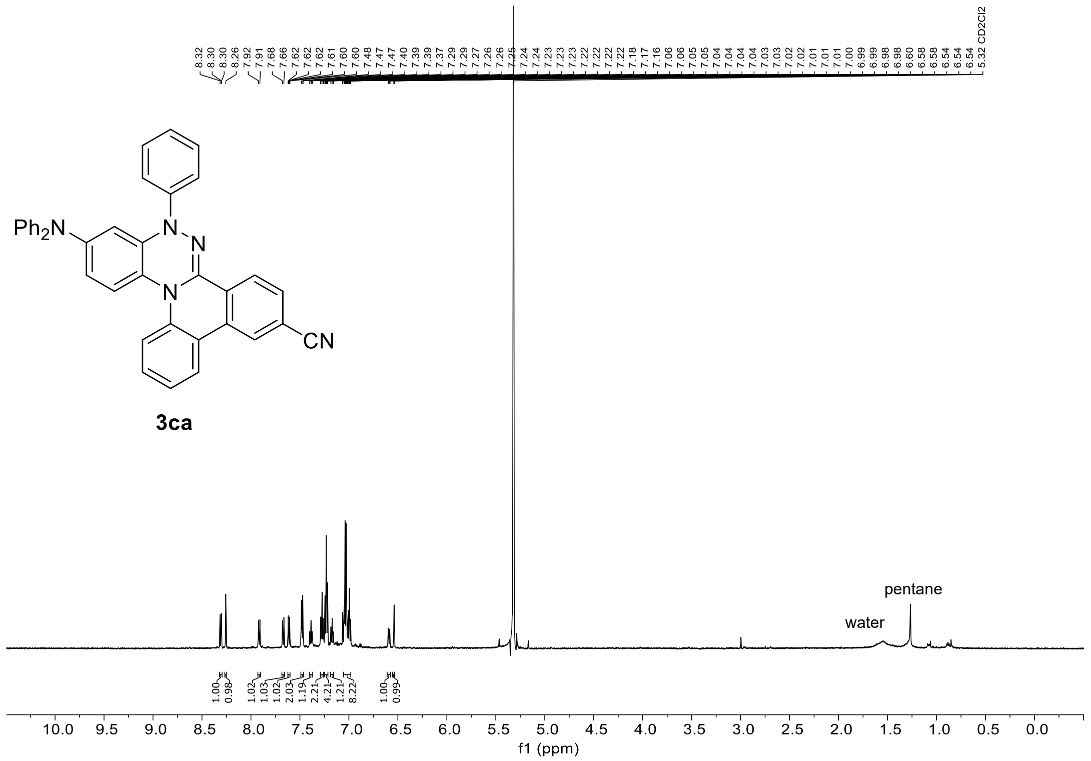

^{13}C-NMR (151 MHz, CD_2Cl_2)

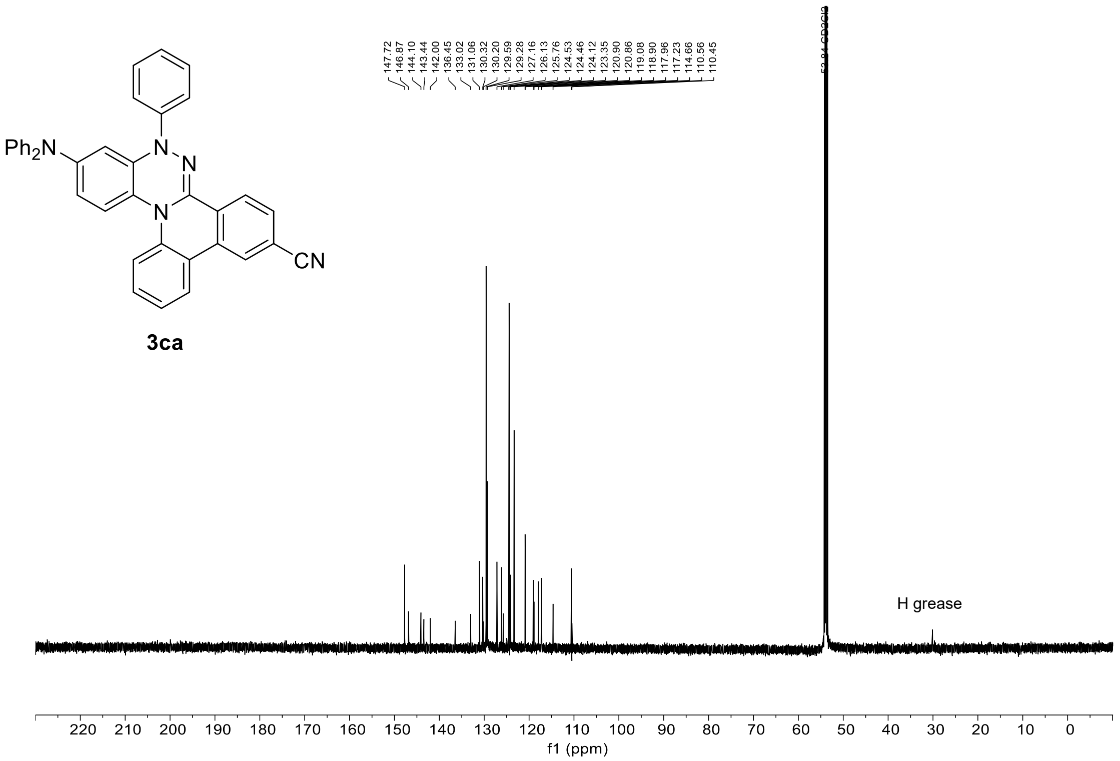

12-(Diphenylamino)-10-phenyl-10H-benzo[5,6][1,2,4]triazino[2,3-f]phenanthridine-3-carbonitrile (**3cb**)

^{1}H-NMR (599 MHz, CD_2Cl_2)

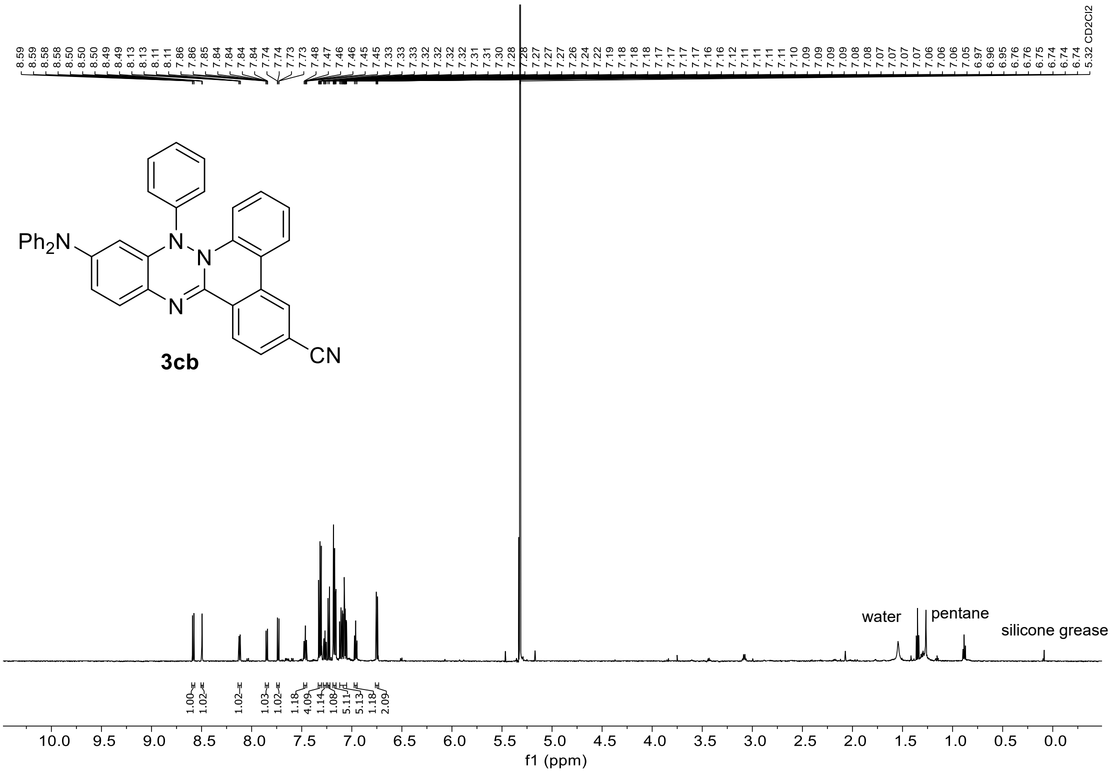

^{13}C-NMR (151 MHz, CD_2Cl_2)

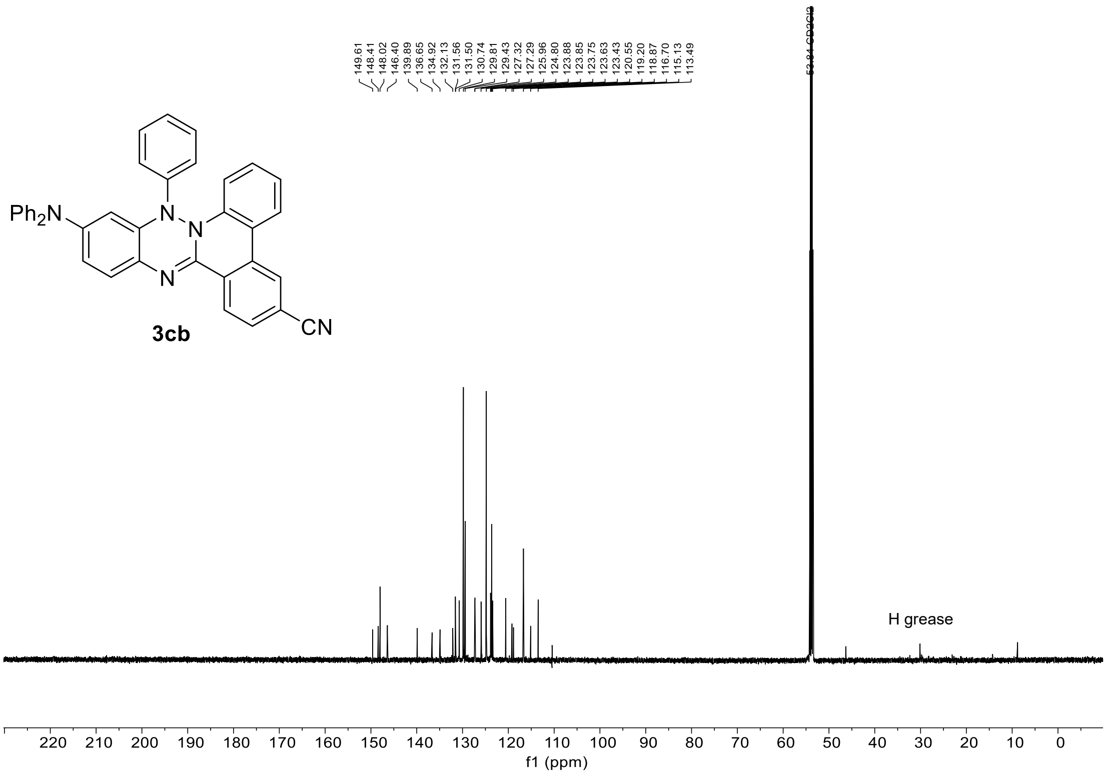

8-(Diphenylamino)-7,9-dimethyl-6-phenyl-6H-benzo[5,6][1,2,4]triazino[4,3-f]phenanthridine-2-carbonitrile (**3da**)

^{1}H-NMR (599 MHz, CD_2Cl_2)

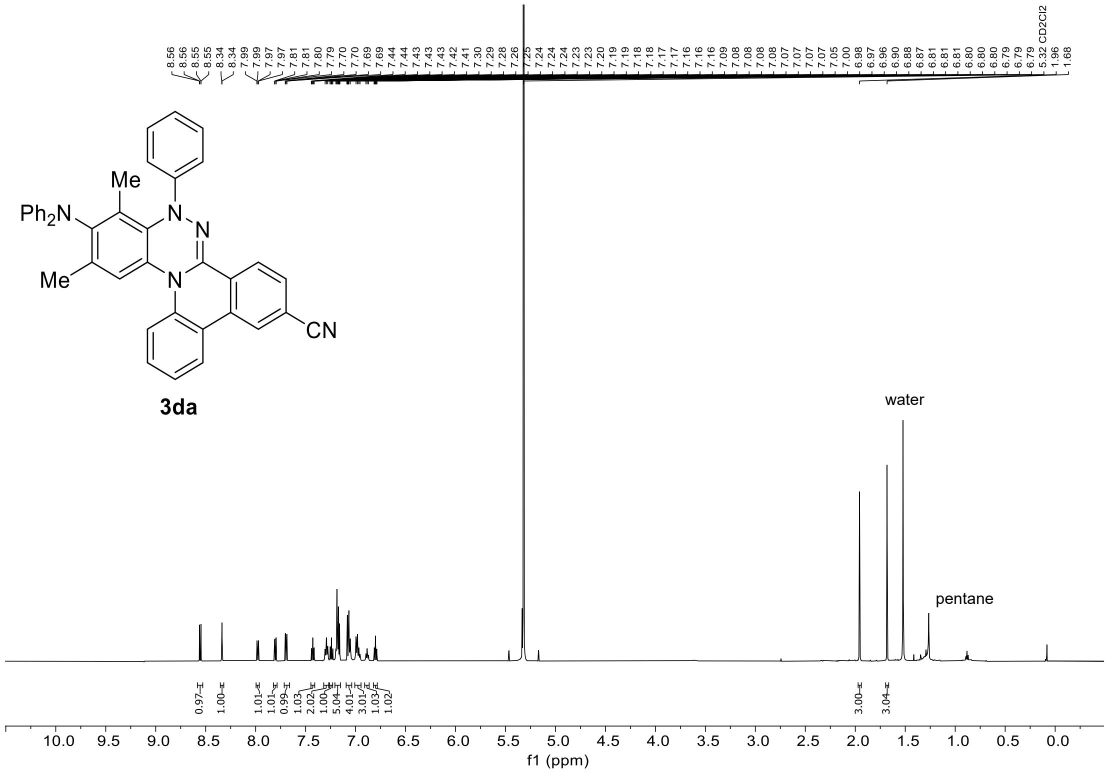

^{13}C-NMR (151 MHz, CD_2Cl_2)

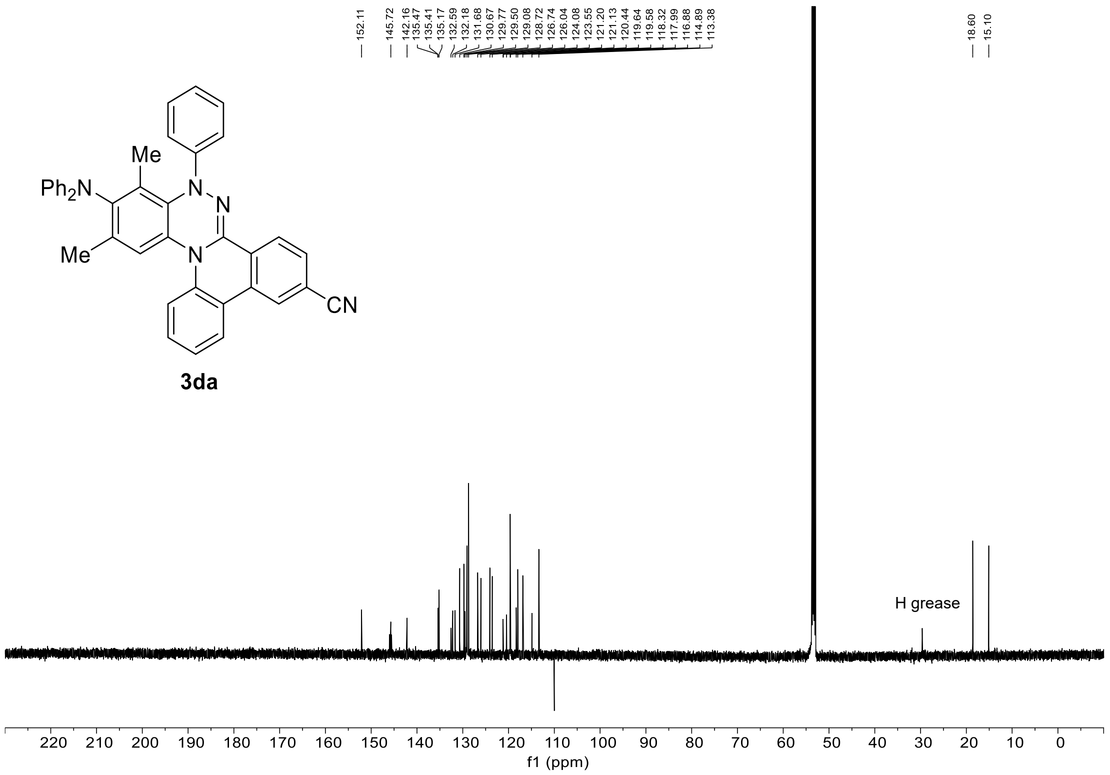

4-(7-(Diphenylamino)-6,8-dimethyl-1,2-diphenyl-1,2-dihydrobenzo[e][1,2,4]triazin-3-yl)benzonitrile (**3db**)

^{1}H-NMR (599 MHz, CD_2Cl_2)

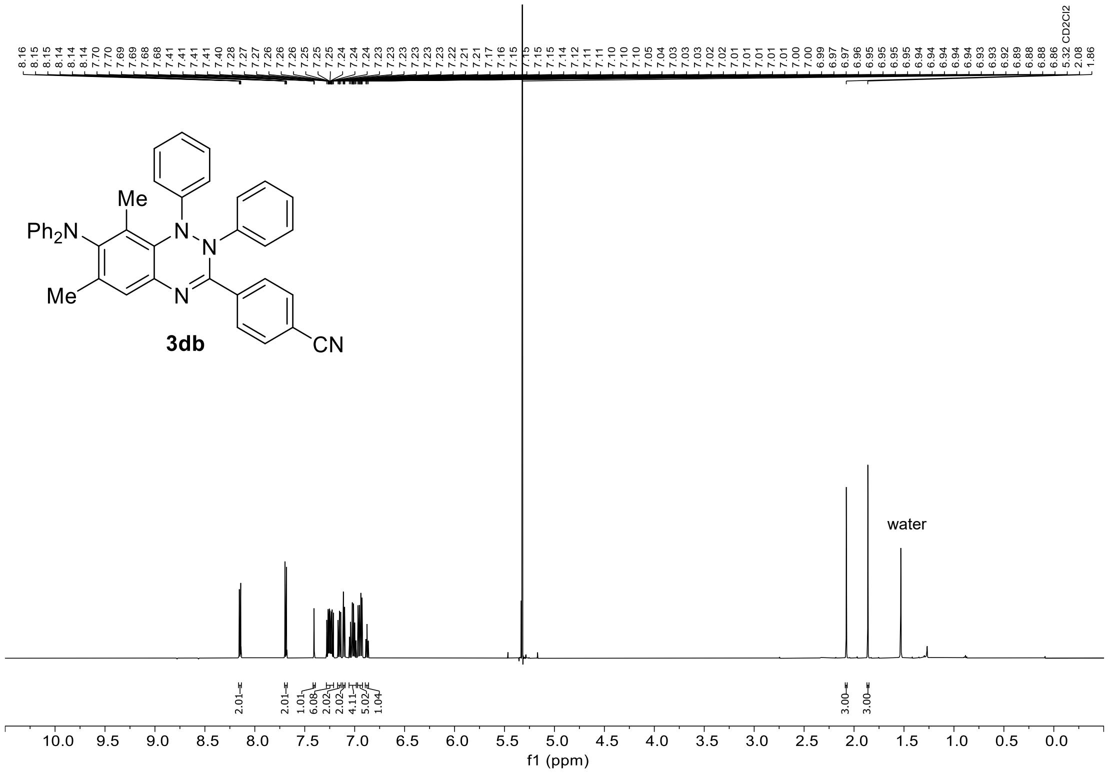

^{13}C-NMR (151 MHz, CD_2Cl_2)

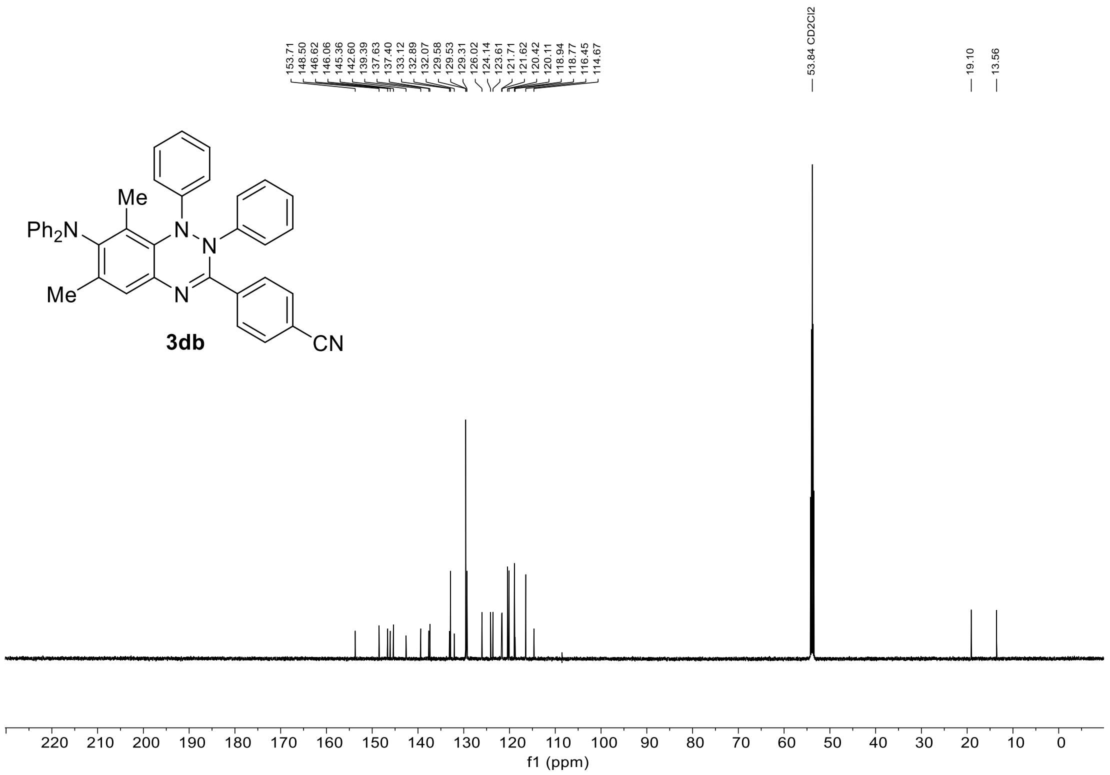

2-Bromo-6-phenyl-6H-benzo[5,6][1,2,4]triazino[4,3-f]phenanthridine (**3ea**)

^{1}H-NMR (599 MHz, CD_2Cl_2)

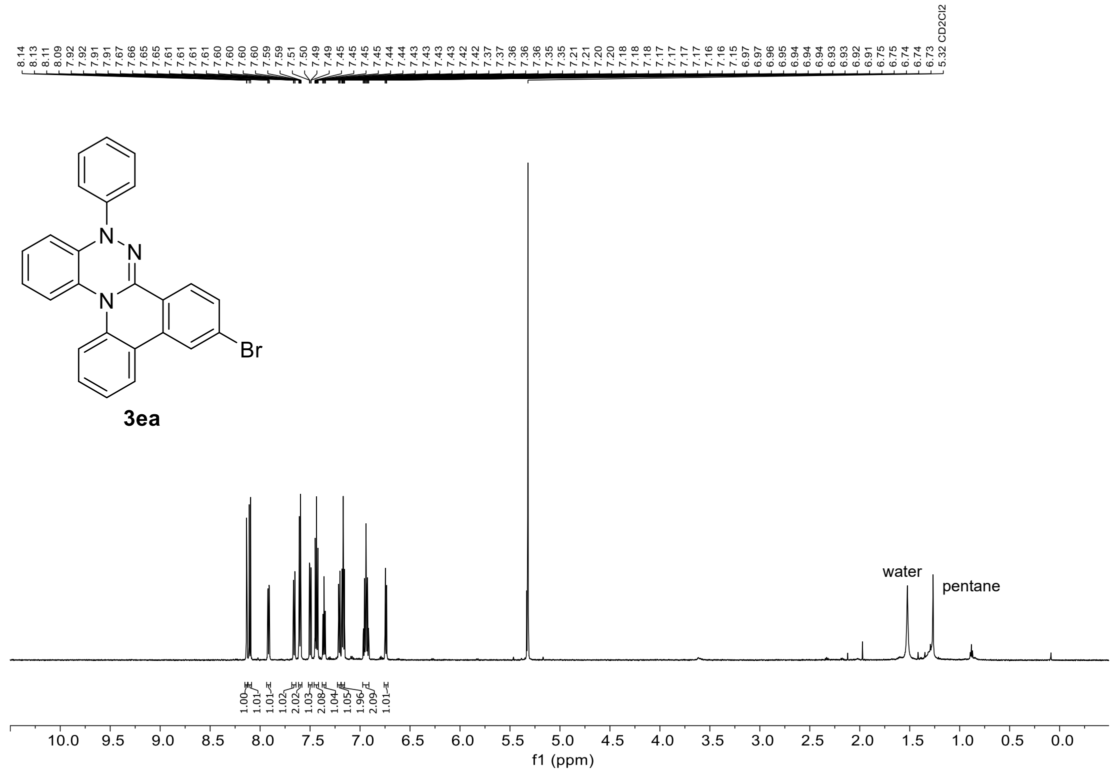

^{13}C-NMR (151 MHz, CD_2Cl_2)

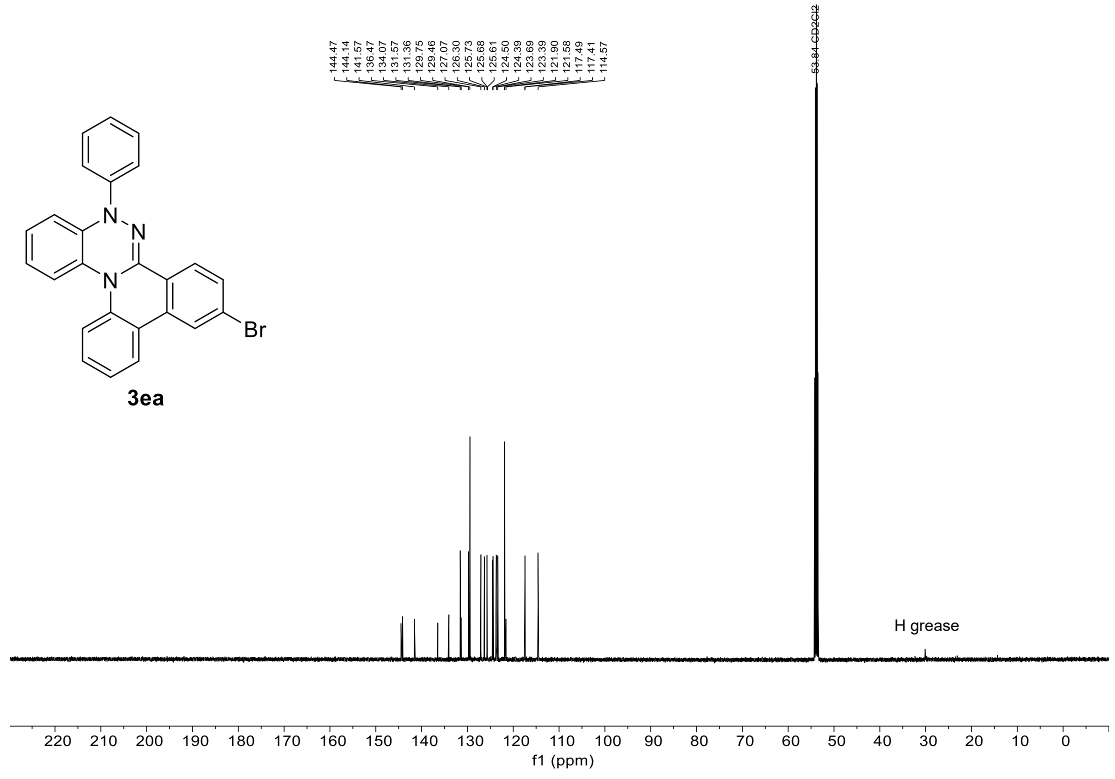

3-Bromo-10-phenyl-10H-benzo[5,6][1,2,4]triazino[2,3-f]phenanthridine (**3eb**)

^{1}H-NMR (599 MHz, CD_2Cl_2)

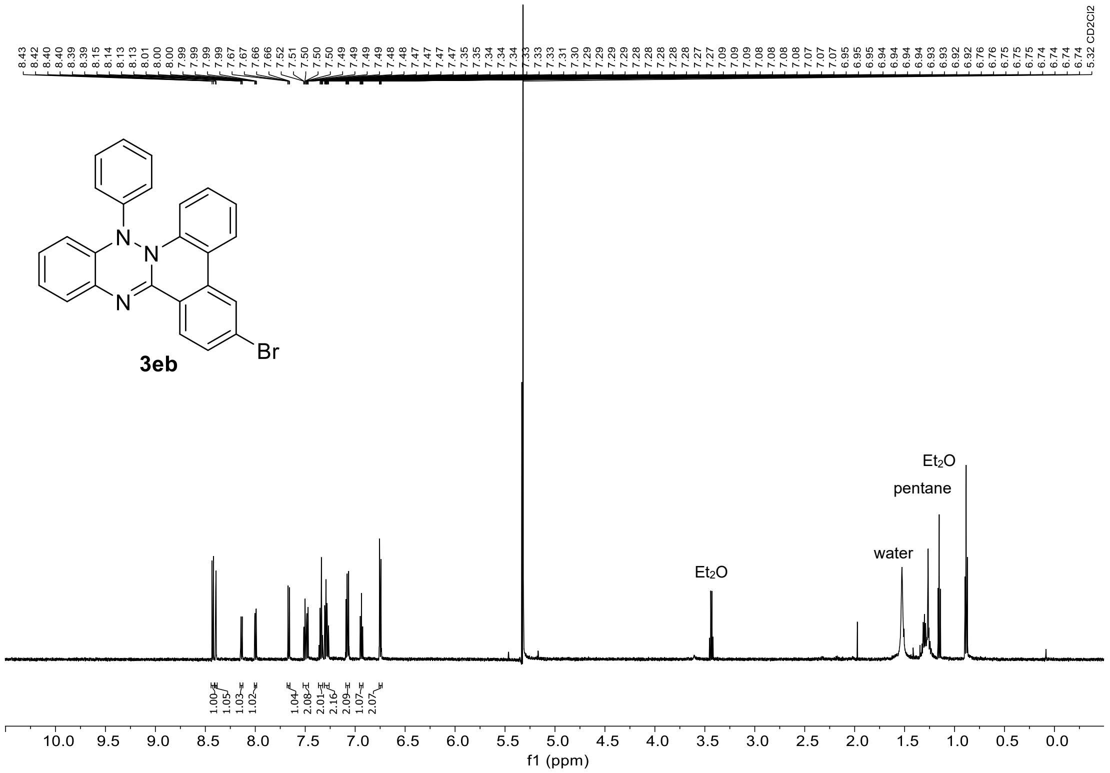

^{13}C-NMR (151 MHz, CD_2Cl_2)

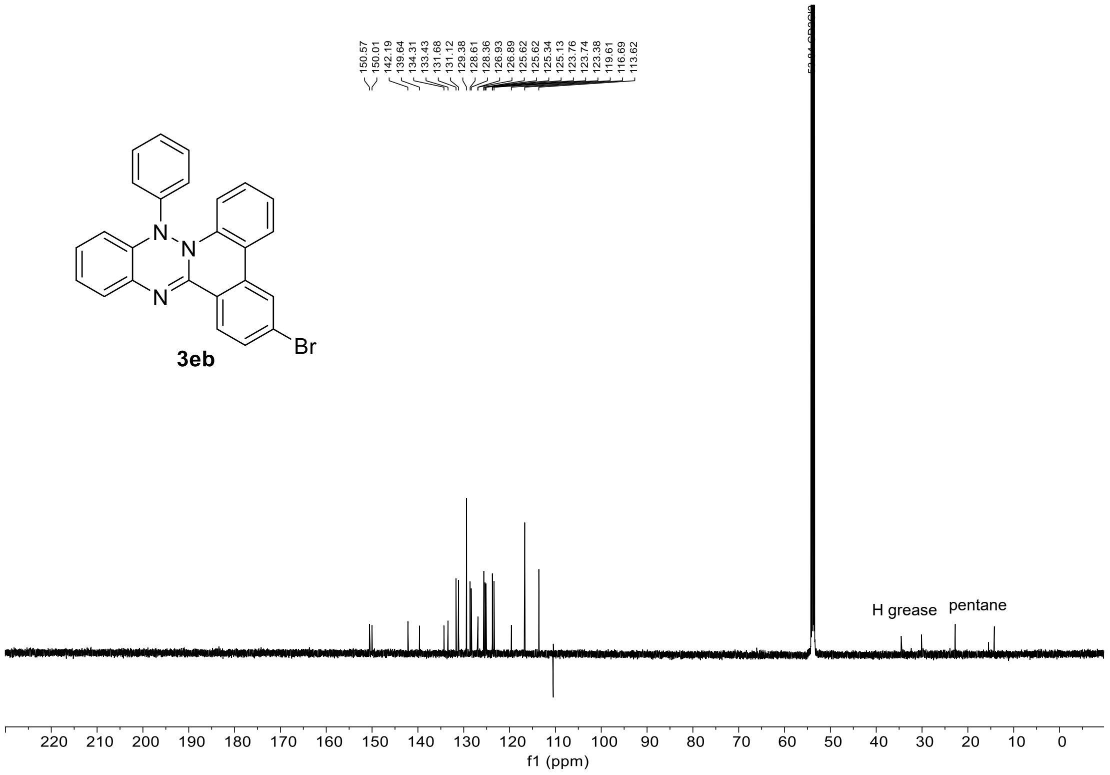

2-Methoxy-6-phenyl-6H-benzo[5,6][1,2,4]triazino[4,3-f]phenanthridine (**3fa**)

^{1}H-NMR (500 MHz, CD_2Cl_2, 183 K)

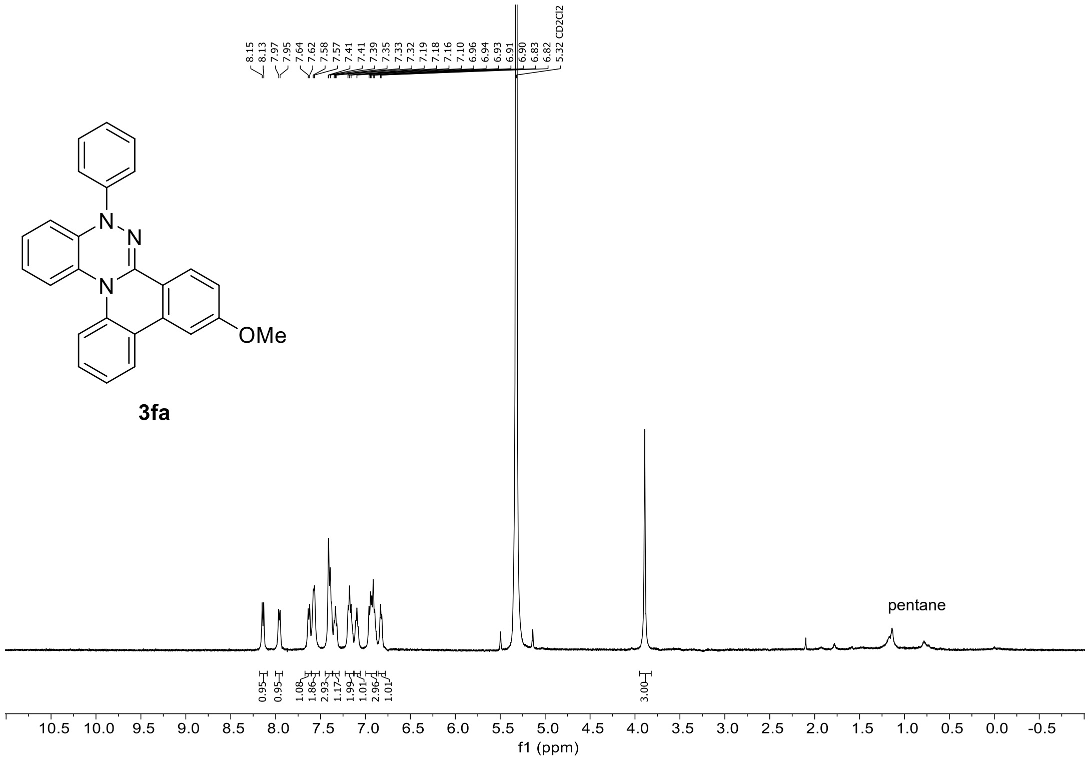

^{13}C-NMR (126 MHz, CD_2Cl_2, 183 K)

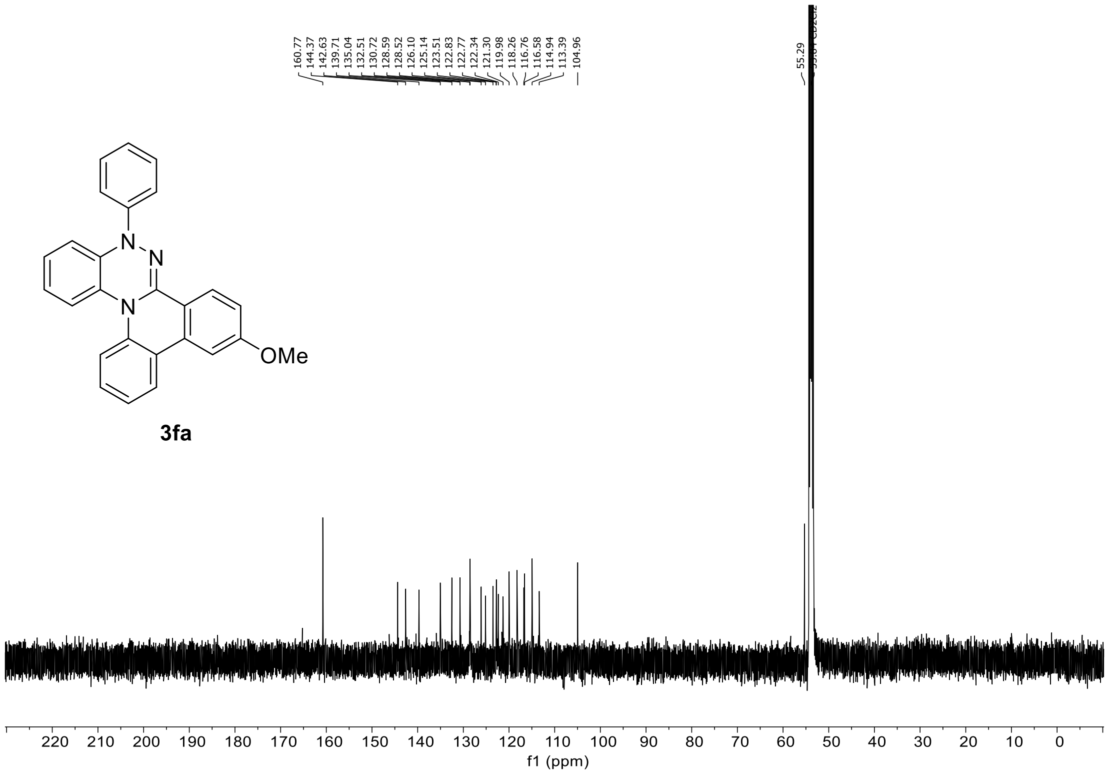

3-Methoxy-10-phenyl-10H-benzo[5,6][1,2,4]triazino[2,3-f]phenanthridine (**3fb**)

^{1}H-NMR (599 MHz, CD_2Cl_2)

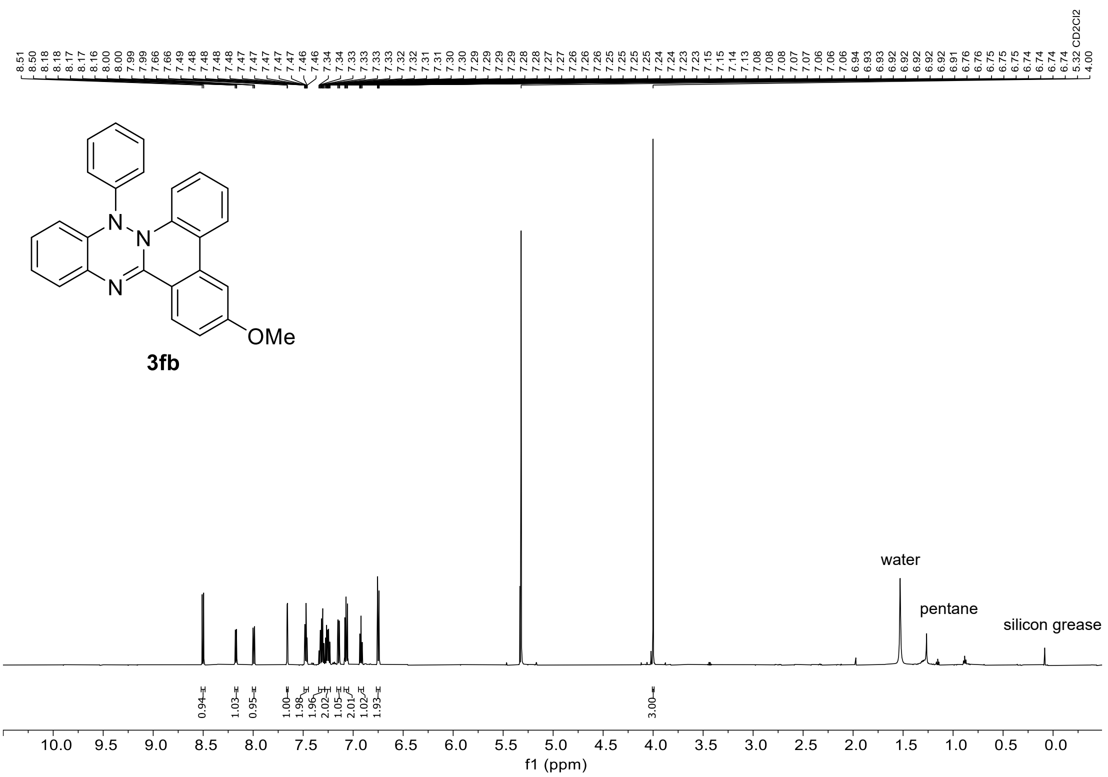

^{13}C-NMR (151 MHz, CD_2Cl_2)

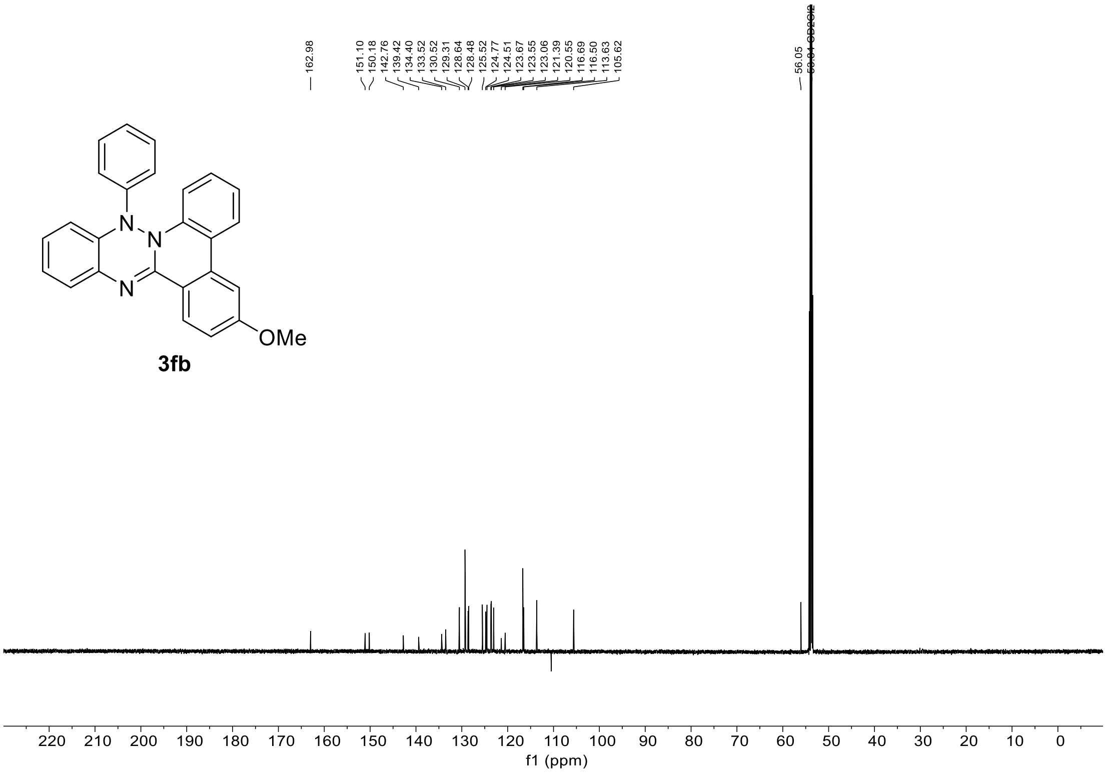

4-(7-Methyl-3,4-diphenylbenzo[e][1,2,4]triazin-1(4H)-yl)benzonitrile (**3ga**)

^{1}H-NMR (599 MHz, CD_2Cl_2)

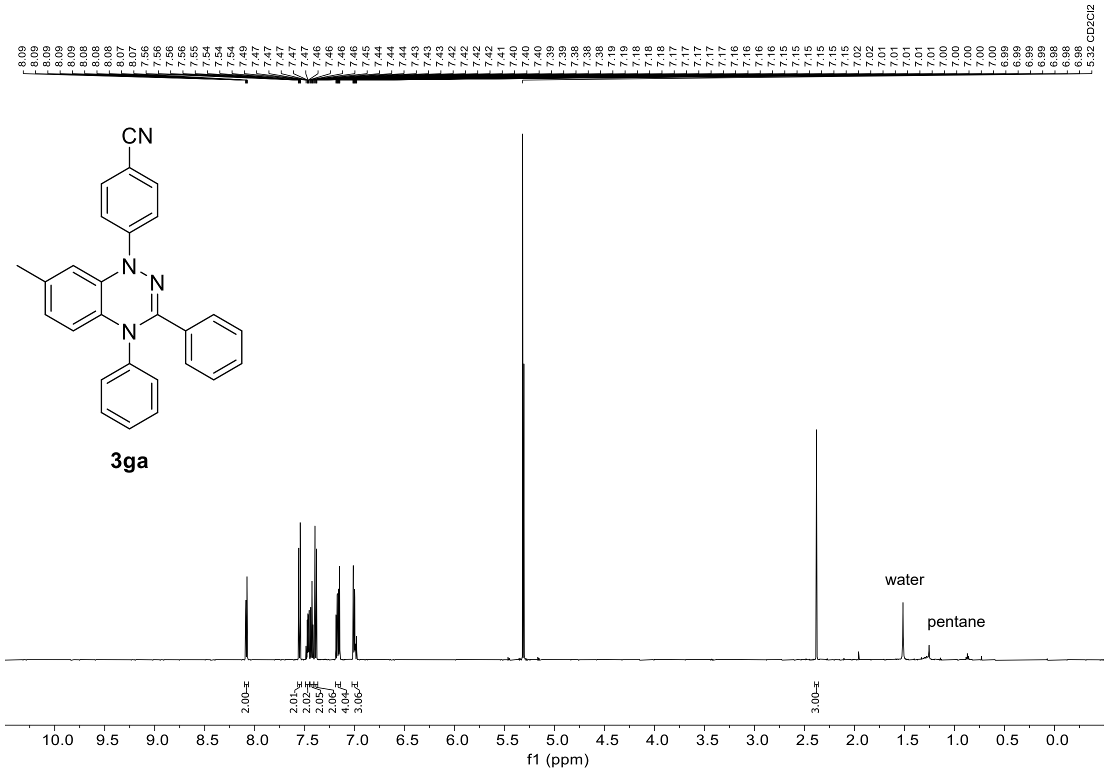

^{13}C-NMR (151 MHz, CD_2Cl_2)

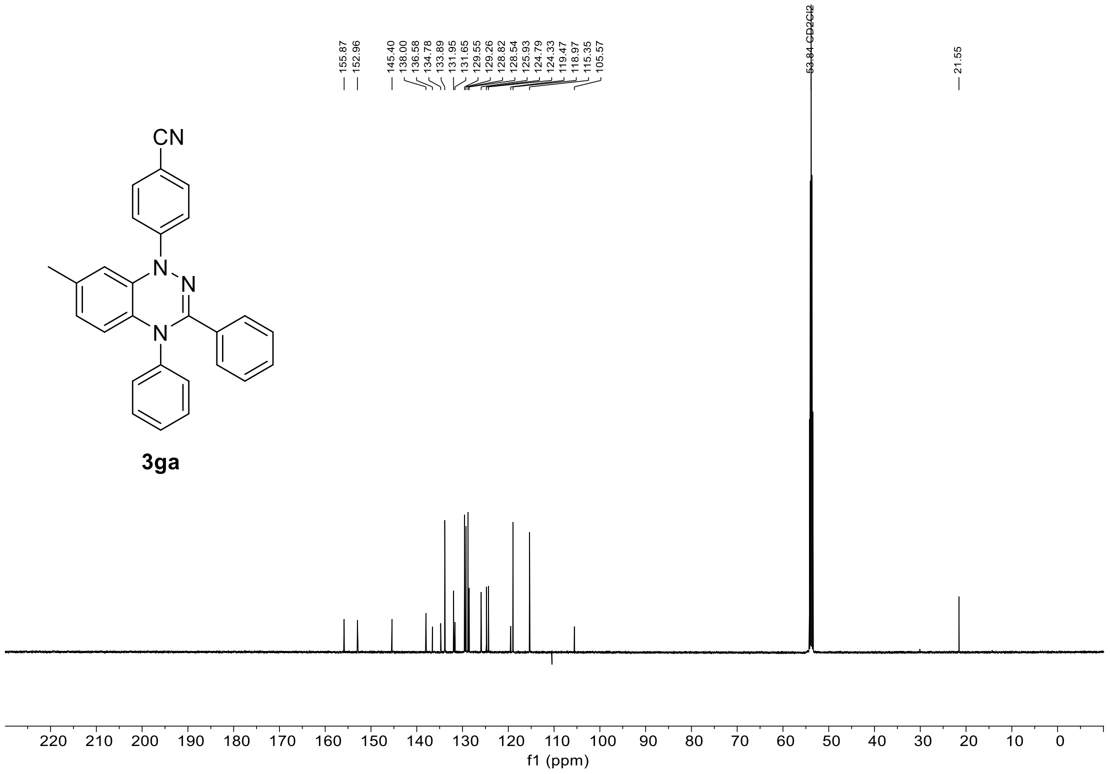

4-(7-Methyl-2,3-diphenylbenzo[e][1,2,4]triazin-1(2H)-yl)benzonitrile (**3gb**)

^{1}H-NMR (599 MHz, CD_2Cl_2)

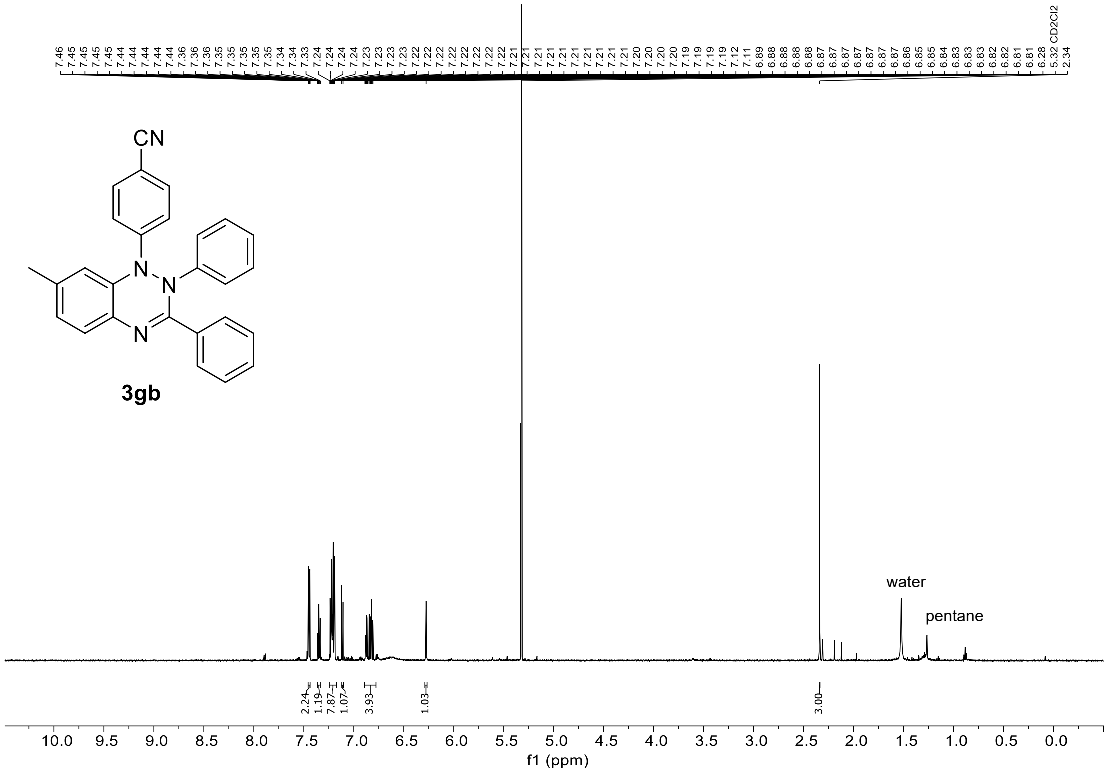

^{13}C-NMR (151 MHz, CD_2Cl_2)

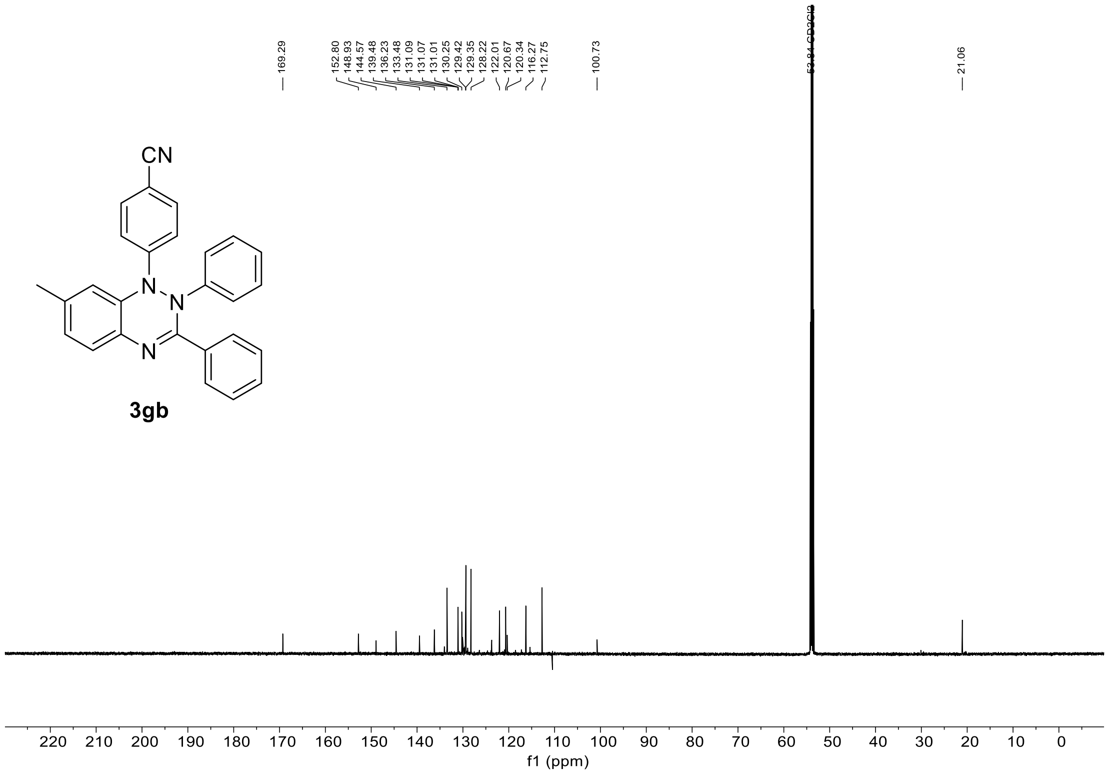

2,4-Diisopropyl-2H-[1,2,4,5]tetrazino[1,6-f]phenanthridin-3(4H)-one (**4aa**)

^{1}H-NMR (599 MHz, CD_2Cl_2)

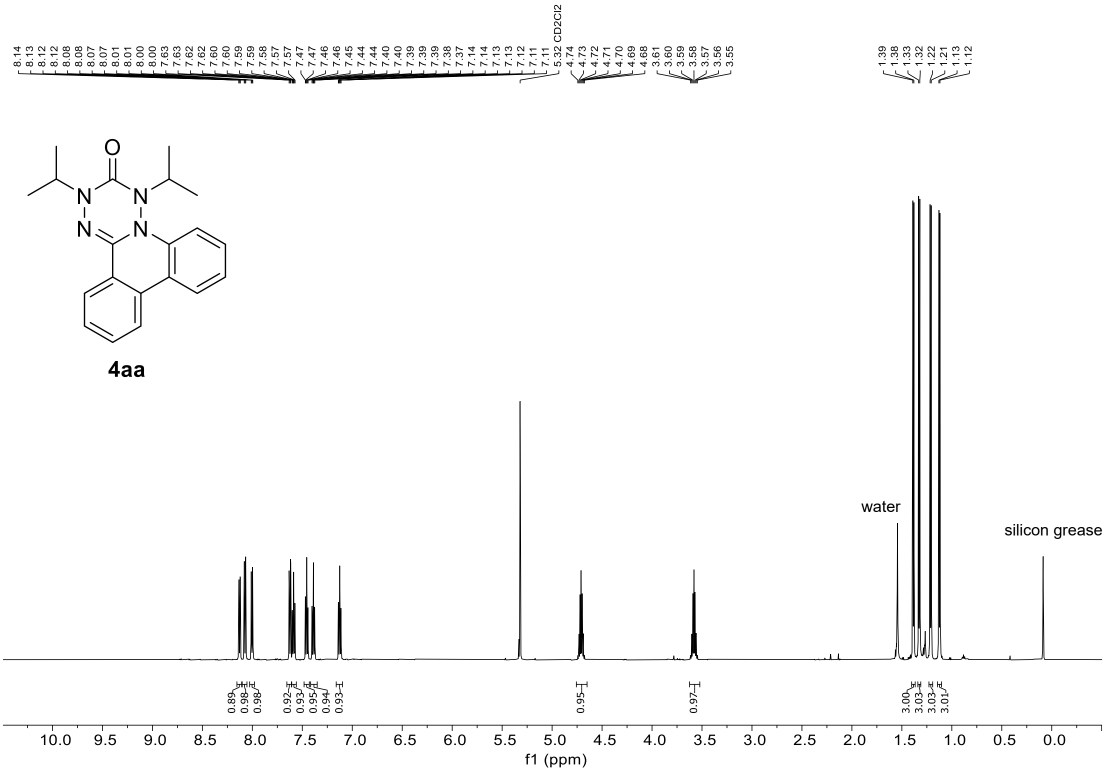

^{13}C-NMR (151 MHz, CD_2Cl_2)

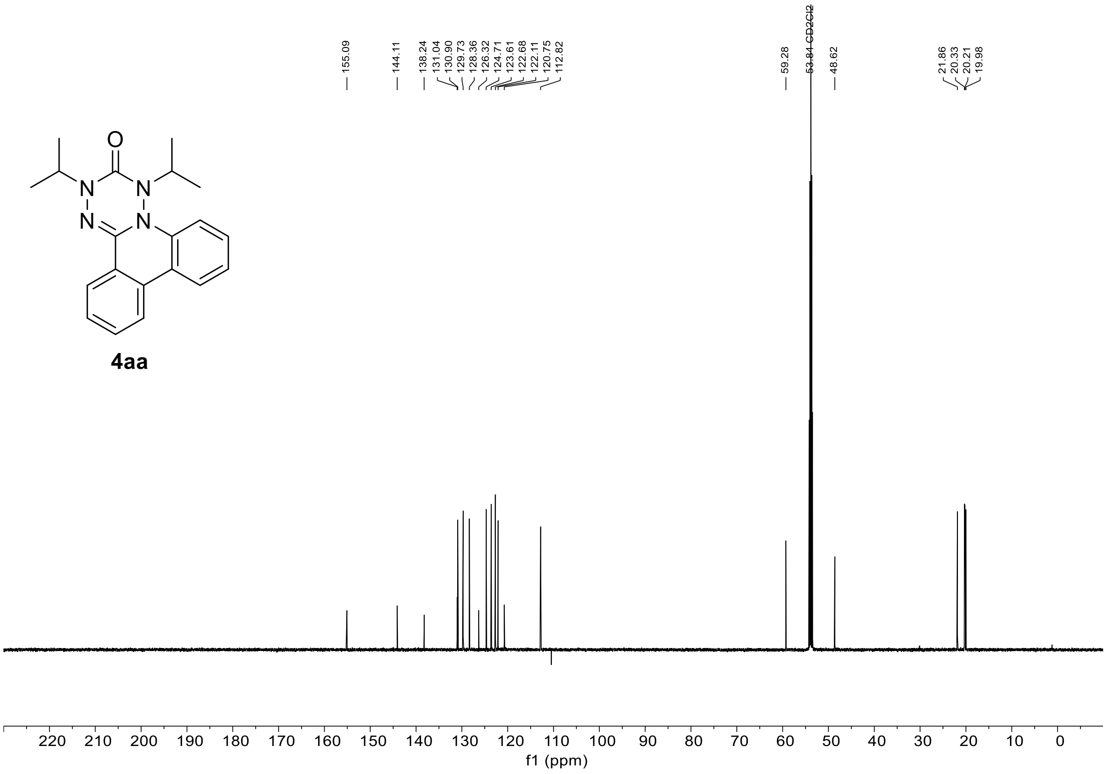

2,4-Diphenyl-2H-[1,2,4,5]tetrazino[1,6-f]phenanthridin-3(4H)-one (**4ca**)

^{1}H-NMR (600 MHz, CD_2Cl_2)

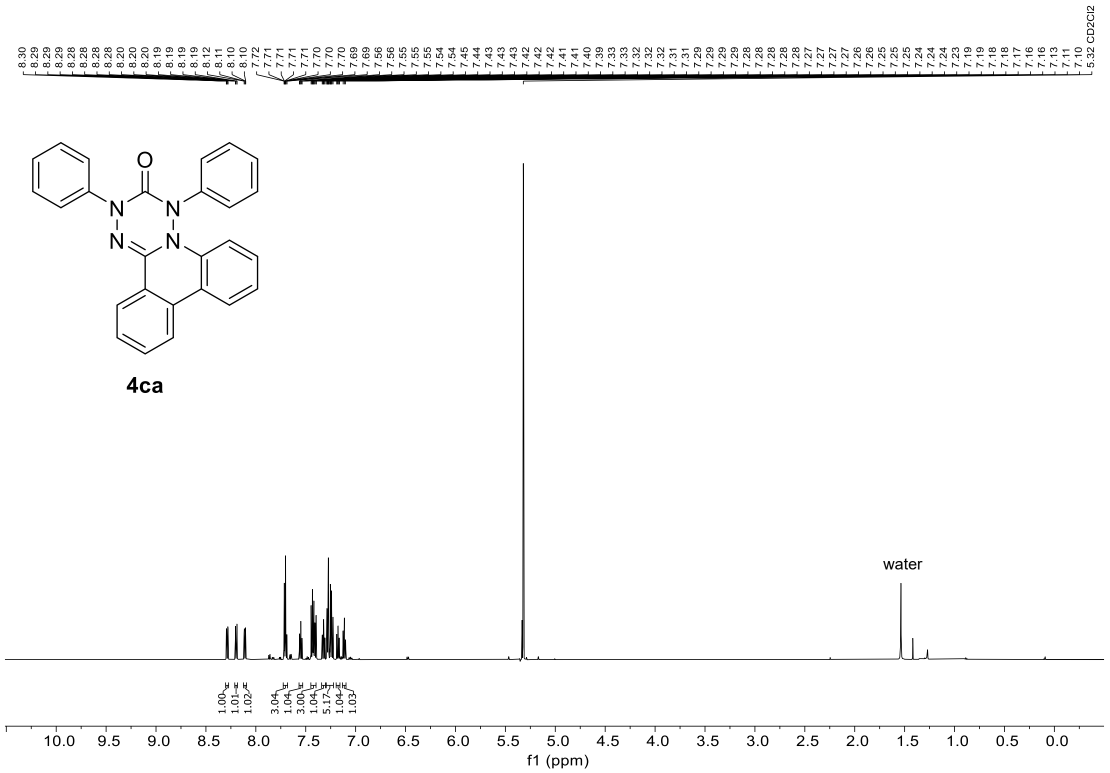

^{13}C-NMR (126 MHz, CD_2Cl_2)

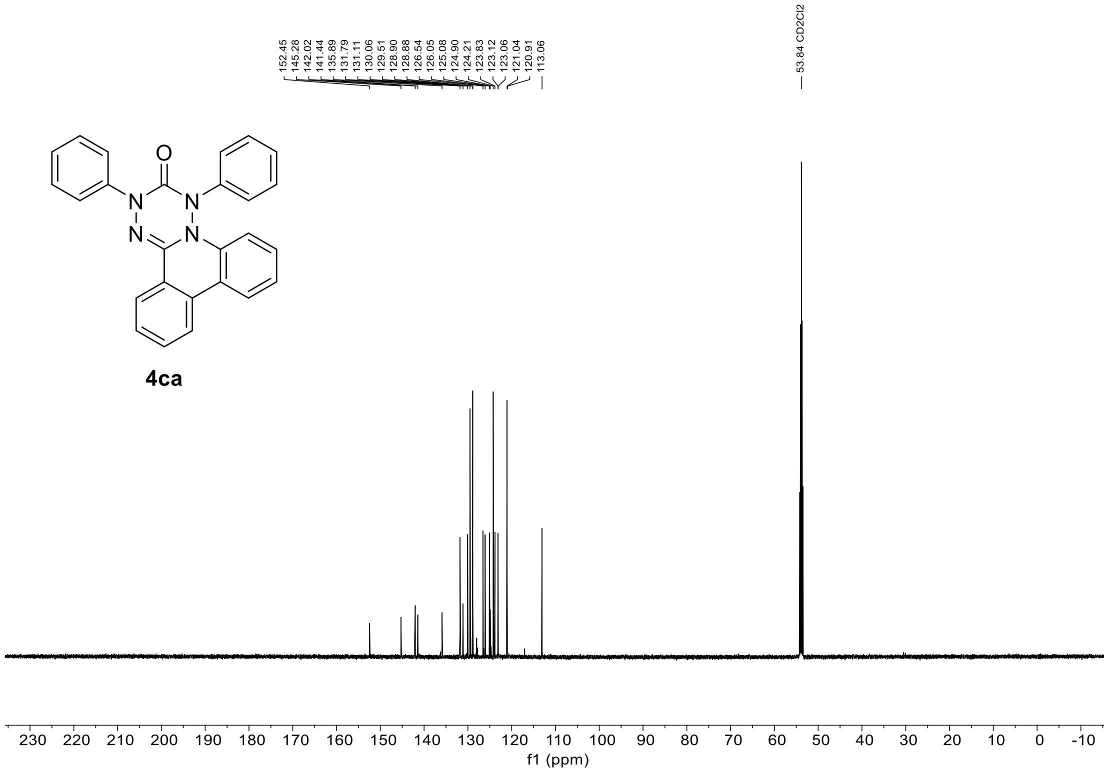

1,2,4,6-Tetraphenyl-1,4-dihydro-1,2,4,5-tetrazin-3(2H)-one (**4cb**)

^{1}H-NMR (599 MHz, CD_2Cl_2)

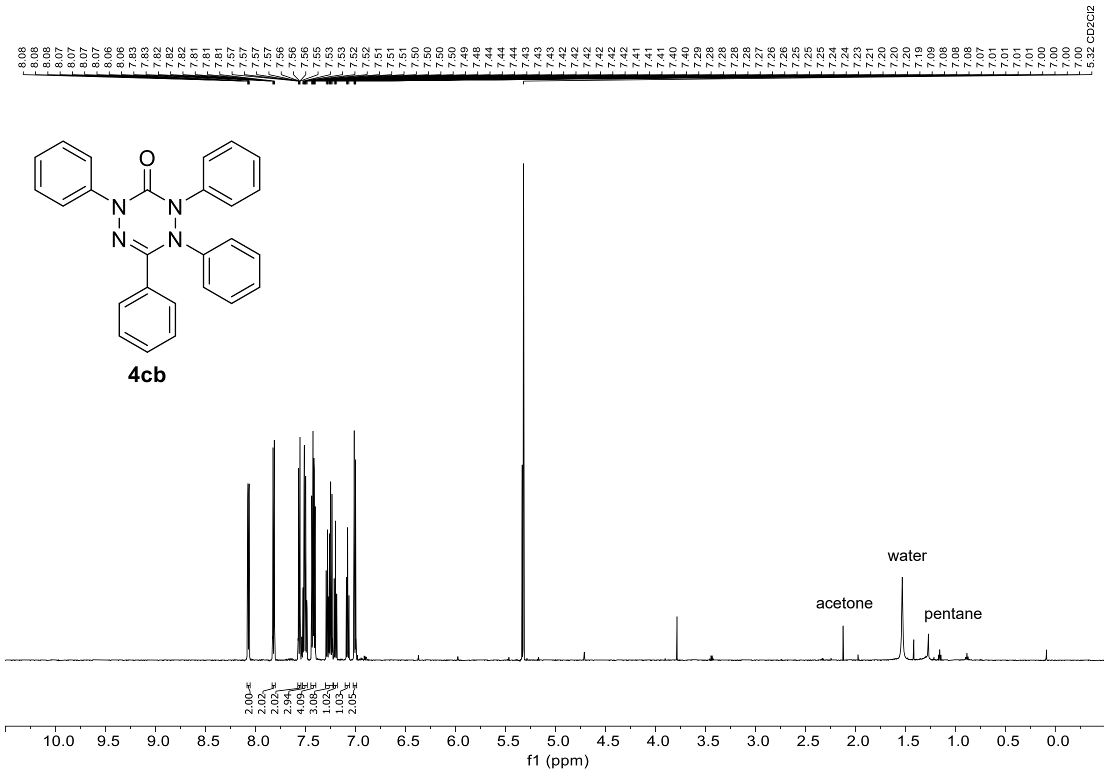

^{13}C-NMR (151 MHz, CD_2Cl_2)

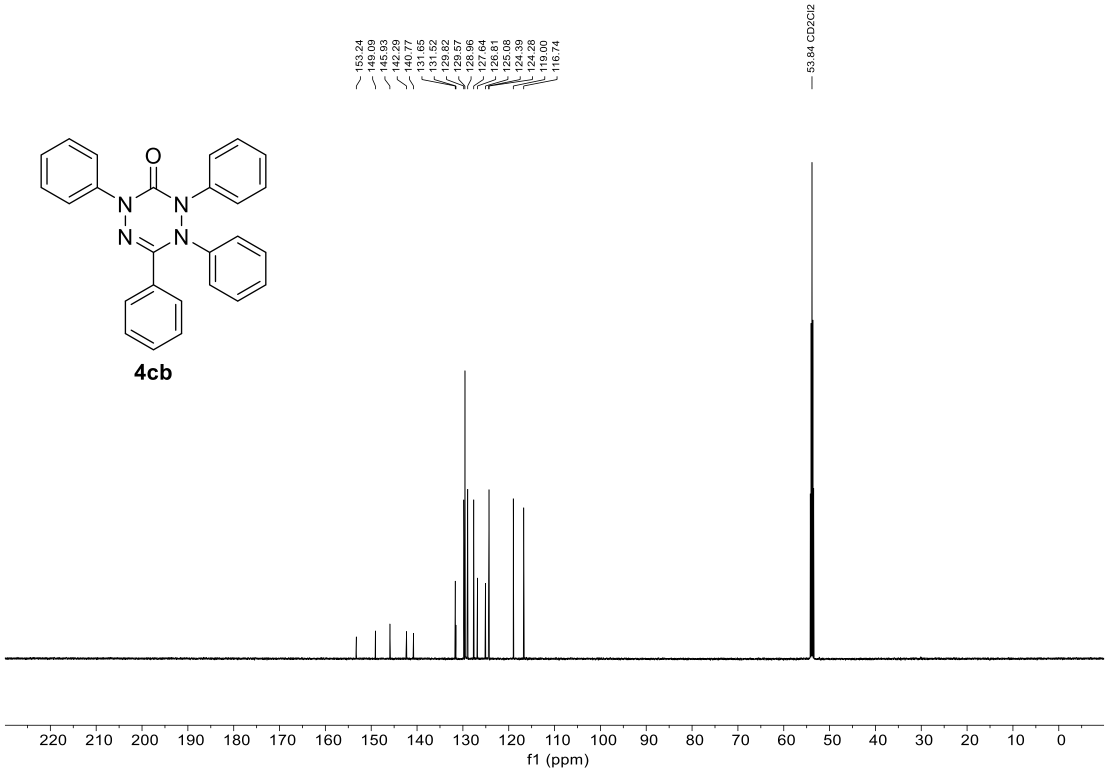

8,15-Dimethoxy-6-phenyl-6H-benzo[5,6][1,2,4]triazino[4,3-f]phenanthridine-2-carbonitrile (**5aa**)

^{1}H-NMR (599 MHz, CD_2Cl_2)

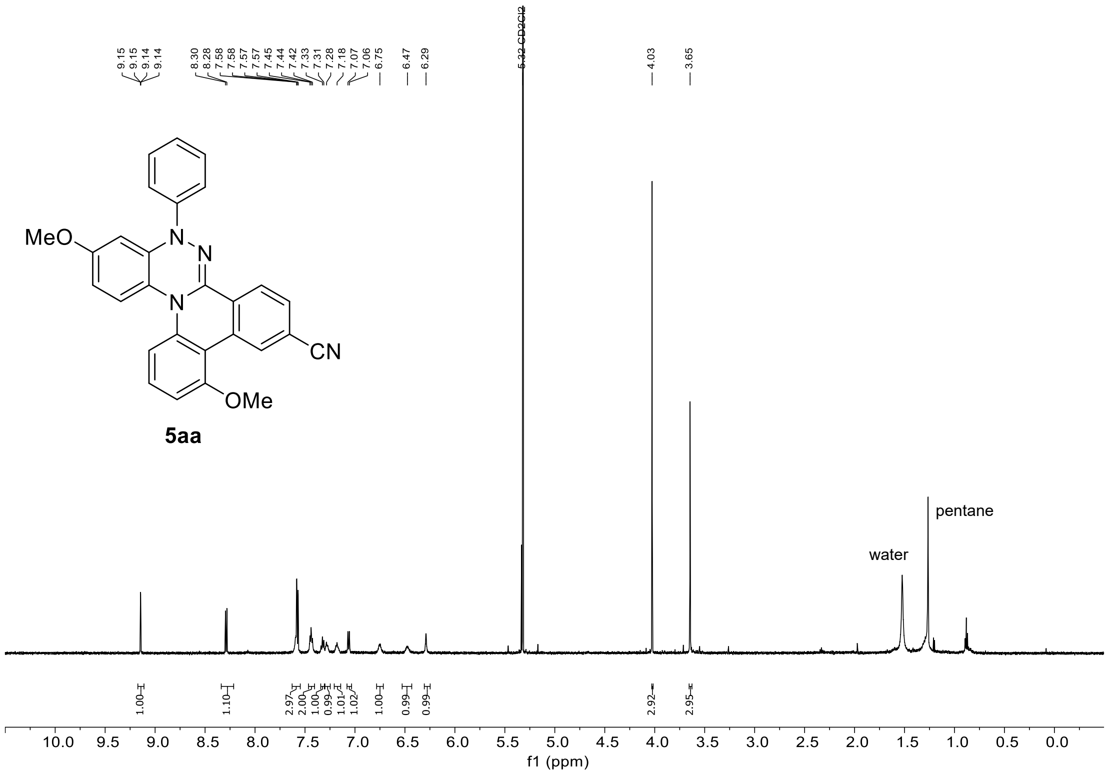

^{13}C-NMR (151 MHz, CD_2Cl_2)

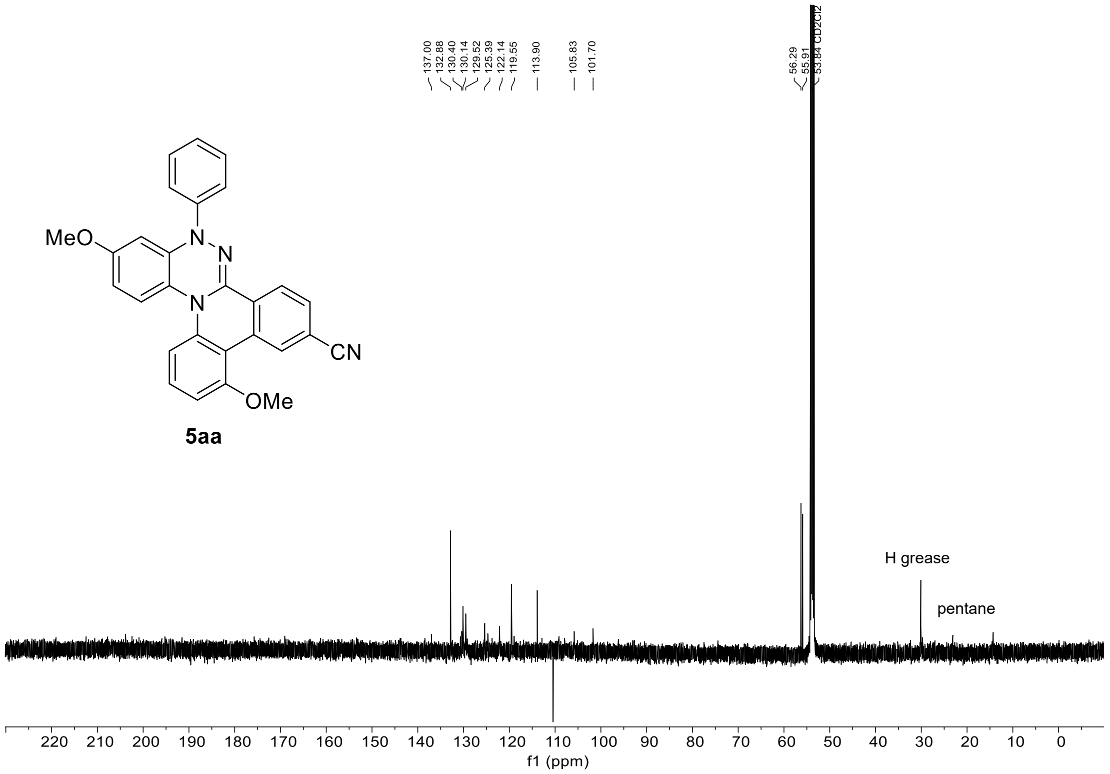

5,12-Dimethoxy-10-phenyl-10H-benzo[5,6][1,2,4]triazino[2,3-f]phenanthridine-3-carbonitrile (**5ab**)

^{1}H-NMR (599 MHz, CD_2Cl_2)

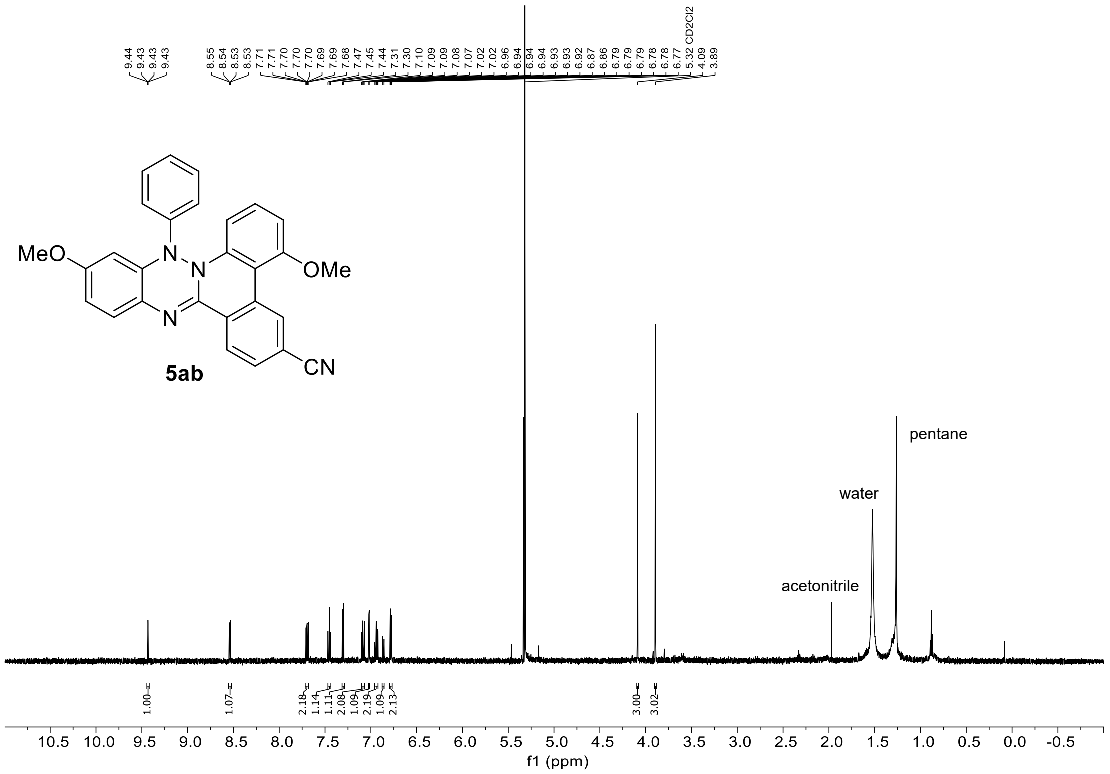

^{13}C-NMR (151 MHz, CD_2Cl_2)

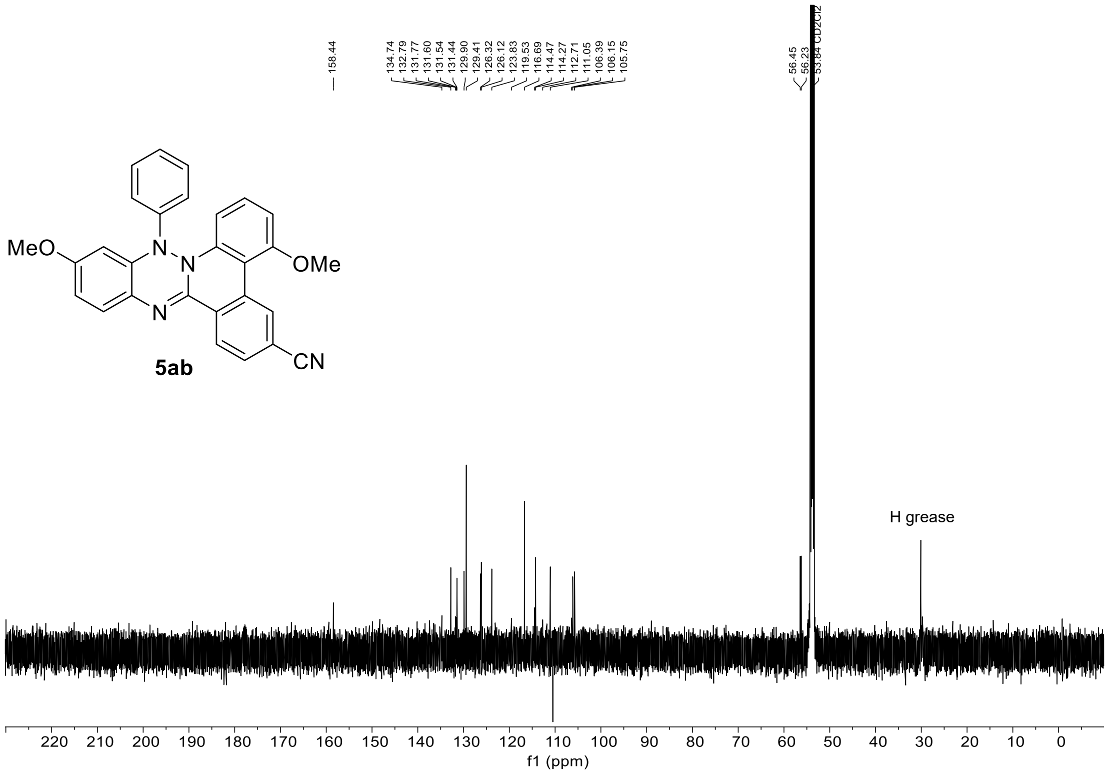

15-Fluoro-8-methoxy-6-phenyl-6H-benzo[5,6][1,2,4]triazino[4,3-f]phenanthridine-2-carbonitrile (**5ba**)

^{1}H-NMR (599 MHz, CD_2Cl_2)

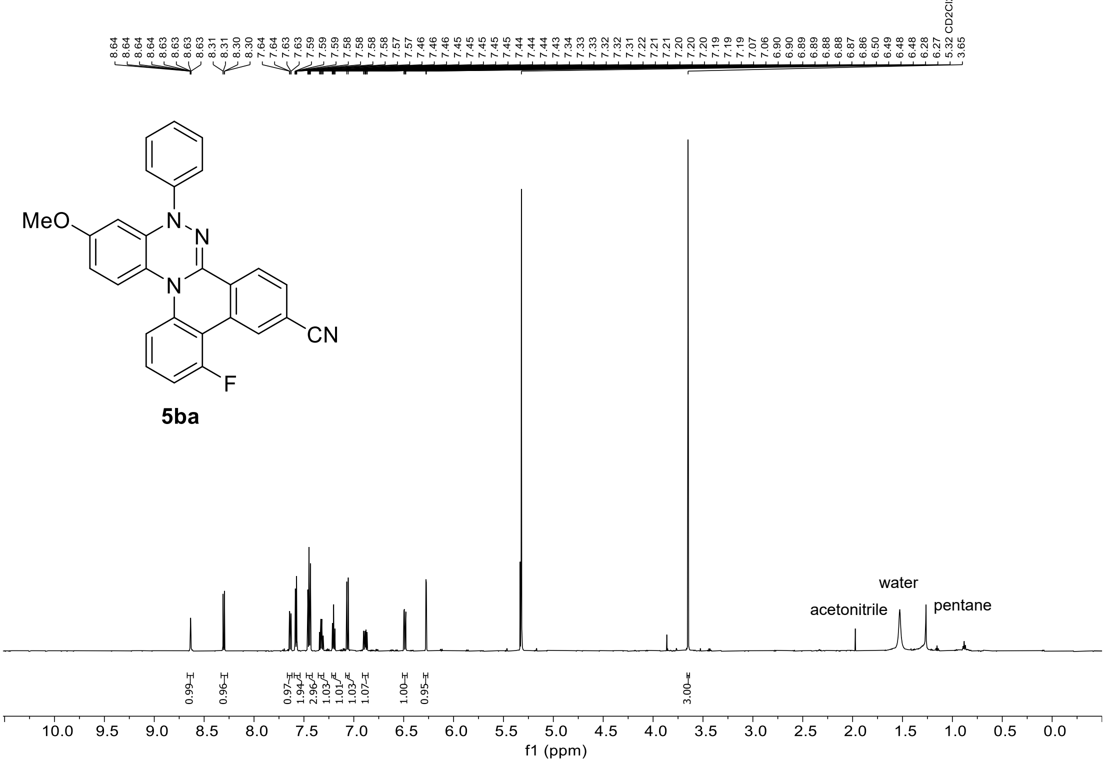

^{13}C-NMR (151 MHz, CD_2Cl_2)

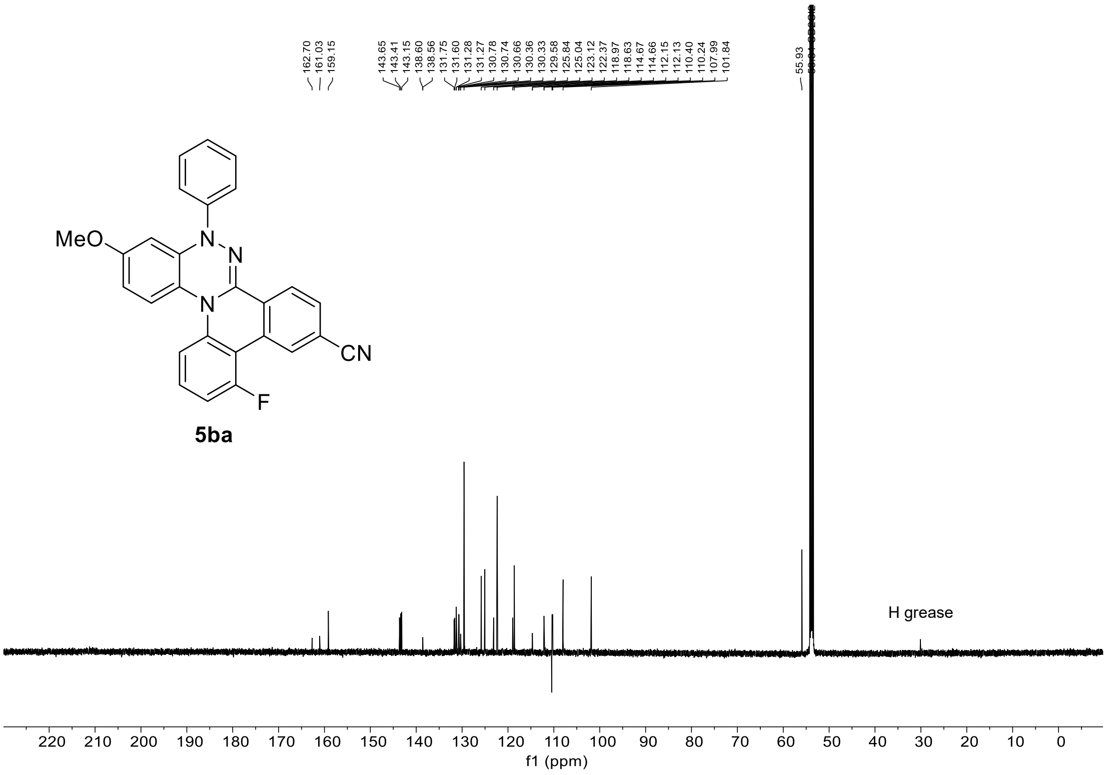

^{19}F-NMR {^{13}C} (563 MHz, $CDCl_3$)

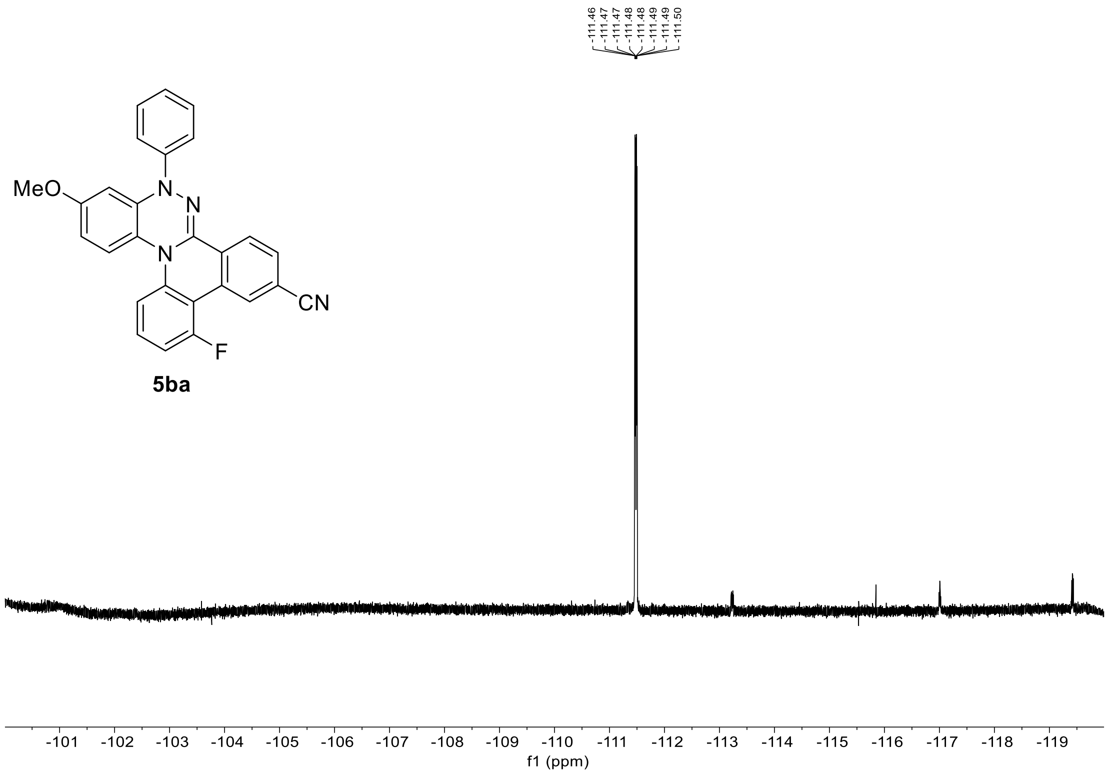

5-Fluoro-12-methoxy-10-phenyl-10H-benzo[5,6][1,2,4]triazino[2,3-f]phenanthridine-3-carbonitrile (**5bb**)

^{1}H-NMR (599 MHz, CD_2Cl_2)

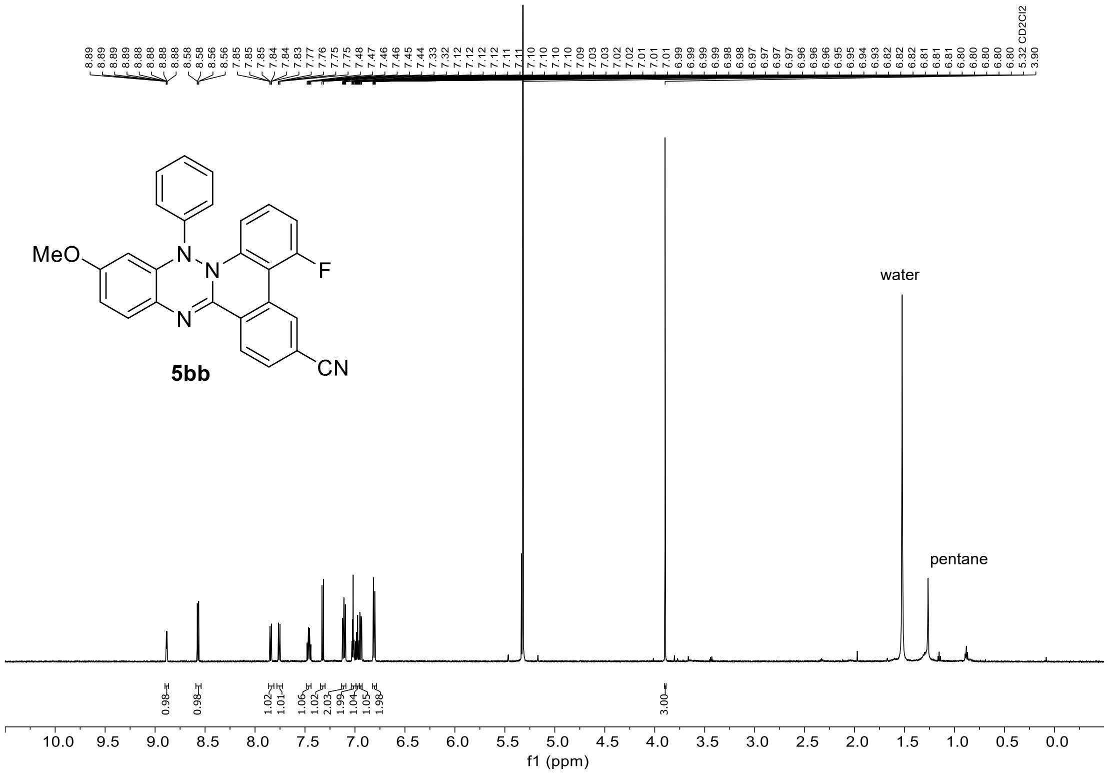

^{13}C-NMR (151 MHz, CD_2Cl_2)

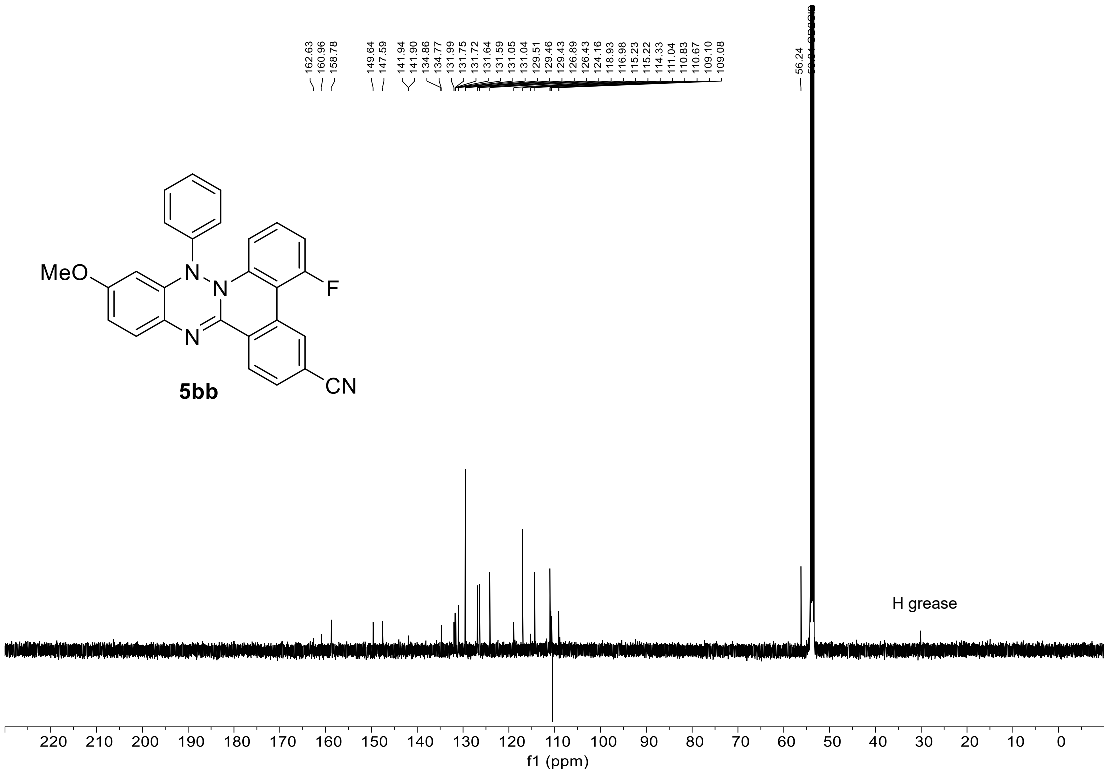

^{19}F-NMR {^{13}C} (563 MHz, CD_2Cl_2)

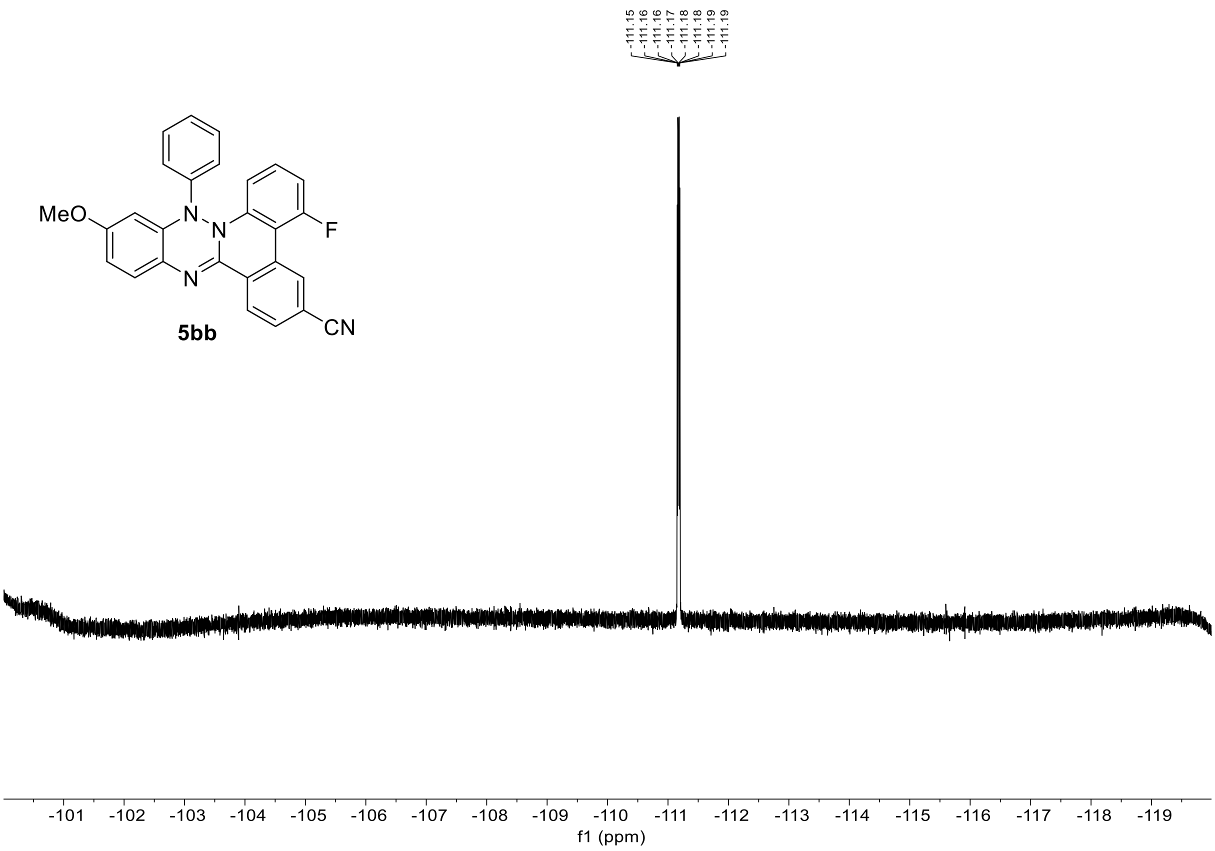

3-Methoxy-5-phenyl-5H-benzo[b]benzo[5,6][1,2,4]triazino[4,3-f]phenanthridine-9-carbonitrile (**5ca**)

^{1}H-NMR (599 MHz, CD_2Cl_2)

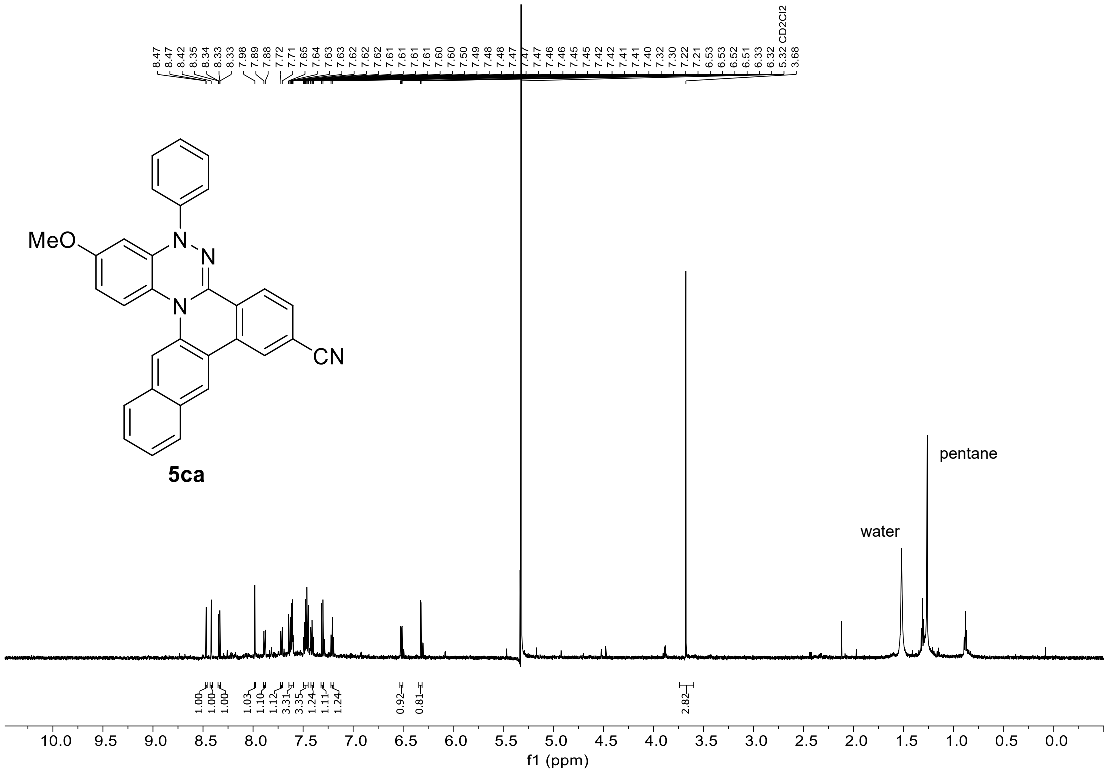

^{13}C-NMR (151 MHz, CD_2Cl_2)

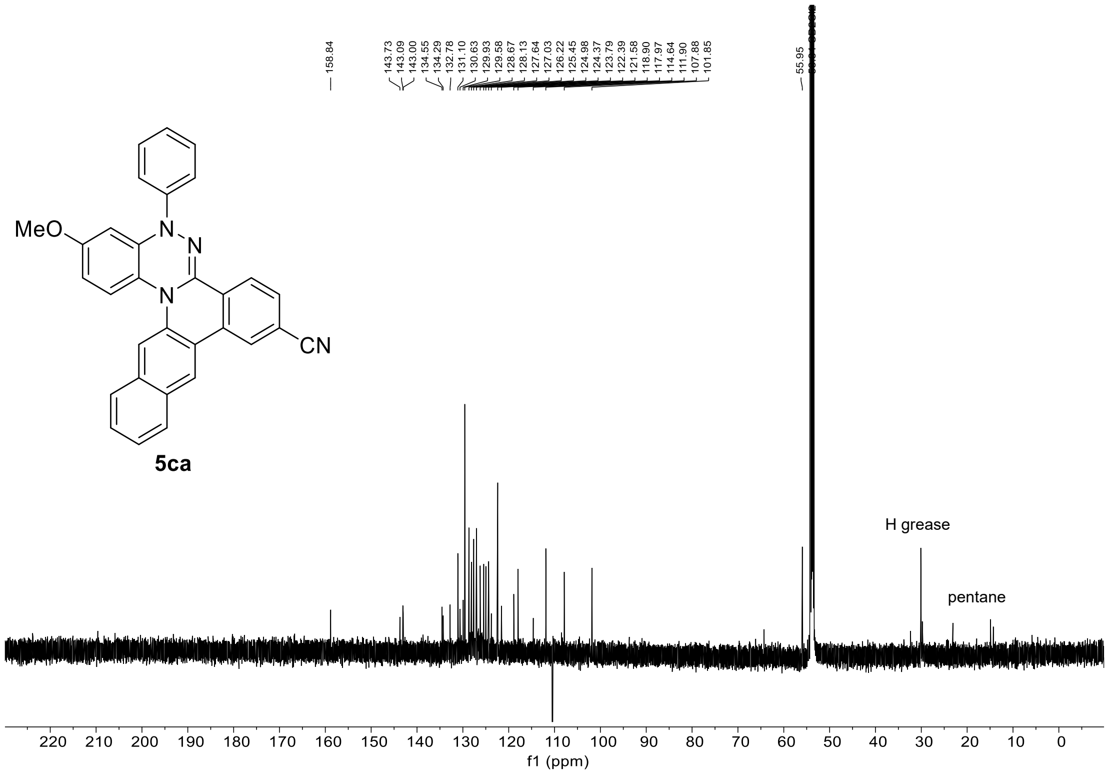

2-Methoxy-17-phenyl-17H-benzo[b]benzo[5,6][1,2,4]triazino[2,3-f]phenanthridine-8-carbonitrile (**5cb**)

^{1}H-NMR (599 MHz, CD_2Cl_2)

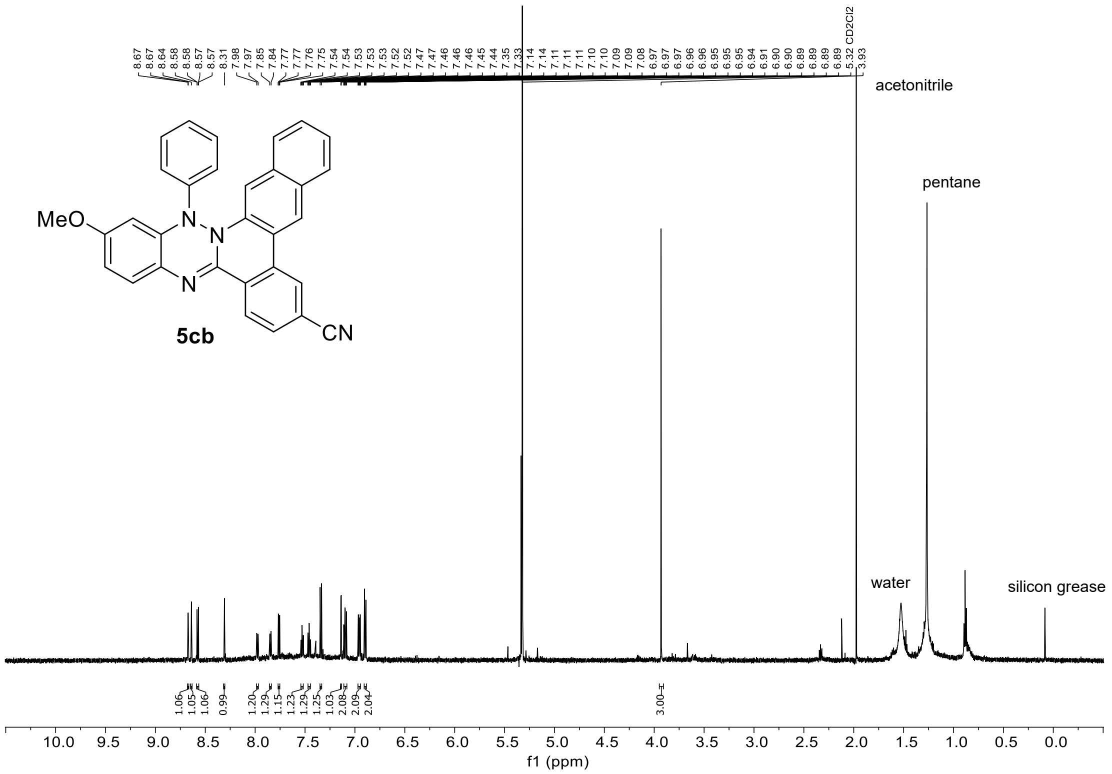

^{13}C-NMR (151 MHz, CD_2Cl_2)

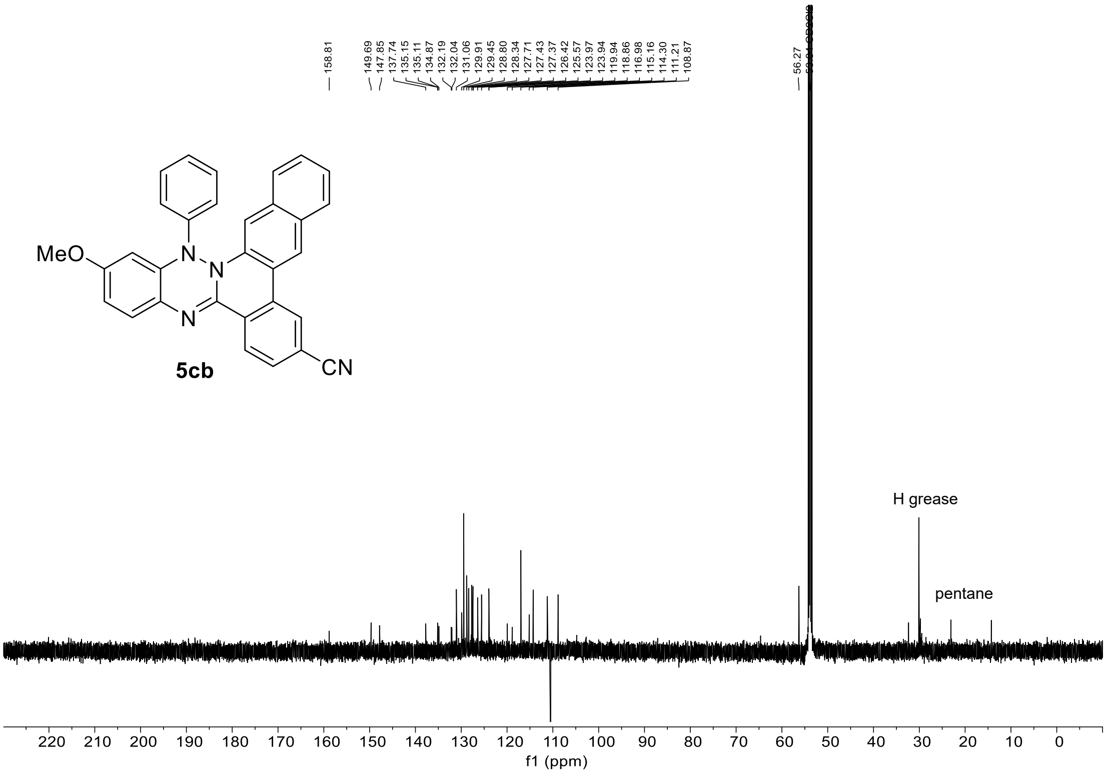

11. References

[1] M. Christmann, *Org. Lett*. **2025**, *27*, 4–7.

[2] D. Matuschek, S. Eusterwiemann, L. Stegemann, C. Doerenkamp, B. Wibbeling, C. G. Daniliuc, N. L. Doltsinis, C. A. Strassert, H. Eckert, A. Studer, *Chem. Sci.* **2015**, *6*, 4712–4716.

[3] J. Exner, I. Maisuls, A. Massolle, S. Klabunde, M. R. Hansen, C. A. Strassert, J. Neugebauer, H. Eckert, A. Studer, *Phys. Chem. Chem. Phys.* **2021**, *23*, 2999–3007.

[4] D. Bhattacharya, M. Scherübl, C. G. Daniliuc, A. Studer, *Chem. Sci.* **2024**, *15*, 13712–13716.

[5] W.-H. Soe, C. Manzano, N. Renaud, P. de Mendoza, A. De Sarkar, F. Ample, M. Hliwa, A. M. Echavarren, N. Chandrasekhar, C. Joachim, *ACS Nano* **2011**, *5*, 1436–1440.

[6] L. Zhang, Y. Geng, Z. Jin, *J Org Chem* **2016**, *81*, 3542–3552.

[7] F. L. Buguis, R. R. Maar, V. N. Staroverov, J. B. Gilroy, *Chem. Eur. J.* **2021**, *27*, 2854–2860.

[8] T. Shi, Z. Wu, T. Jia, C. Zhang, L. Zeng, R. Zhuang, J. Zhang, S. Liu, J. Shao, H. Zhu, *Chem. Commun.* **2021**, *57*, 8460–8463.

[9] H. Xiaoguang, L. Xuying, L. Huaqing, W. Xian, *Benzotriazine Single Free Radical Compound and Preparation Method Thereof*, **2022**, CN202111587846A.

[10] M. S. Santos, A. Nortcliffe, W. Lewis, T. D. Bradshaw, C. J. Moody, *Chem. Eur. J.* **2018**, *24*, 8325–8330.

[11] E. C. Paré, D. J. R. Brook, A. Brieger, M. Badik, M. Schinke, *Org. Biomol. Chem.* **2005**, *3*, 4258–4261.

[12] J. B. Gilroy, S. D. J. McKinnon, P. Kennepohl, M. S. Zsombor, M. J. Ferguson, L. K. Thompson, R. G. Hicks, *J. Org. Chem.* **2007**, *72*, 8062–8069.

[13] S. Eusterwiemann, H. Eckert, C. A. Strassert, N. L. Doltsinis, C. G. Daniliuc, C. C. Doerenkamp, S. Klabunde, L. Stegemann, D. Matuschek, A. Studer, *Chimia* **2016**, *70*, 172.

[14] J. B. Gilroy, S. D. J. McKinnon, B. D. Koivisto, R. G. Hicks, *Org. Lett.* **2007**, *9*, 4837–4840.

[15] Sheldrick, G. M. SHELXT–Integrated Space-Group and Crystal-Structure Determination. *Acta.Cryst.* **2015**, *A71*, 3–8.

[16] Sheldrick, G. M. Crystal Structure Refinement with SHELXL. *Acta. Cryst.* **2015**, *C71*, 3–8.

[17] Bruker AXS (**2024**) *APEX6 Version 2024.9-0*, *SAINT Version 8.41* and *SADABS Bruker AXS area detector scaling and absorption correction Version 2016/2*, Bruker AXS Inc., Madison, Wisconsin, USA.

[18] Bruker AXS (**1998**) *XP – Interactive molecular graphics, Version 5.1,* Bruker AXS Inc., Madison, Wisconsin, USA.

[19] M. J. Frisch, G. W. Trucks, H. B. Schlegel, G. E. Scuseria, M. A. Robb, J. R. Cheeseman, G. Scalmani, V. Barone, G. A. Petersson, H. Nakatsuji, X. Li, M. Caricato, A. V. Marenich, J. Bloino, B. G. Janesko, R. Gomperts, B. Mennucci, H. P. Hratchian, J. V. Ortiz, A. F. Izmaylov, J. L. Sonnenberg, D. WilliamsYoung, F. Ding, F. Lipparini, F. Egidi, J. Goings, B. Peng, A. Petrone, T. Henderson, D. Ranasinghe, V. G. Zakrzewski, J. Gao, N. Rega, G. Zheng, W. Liang, M. Hada, M. Ehara, K. Toyota, R. Fukuda, J. Hasegawa, M. Ishida, T. Nakajima, Y. Honda, O. Kitao, H. Nakai, T. Vreven, K. Throssell, J. A. Montgomery, Jr., J. E. Peralta, F. Ogliaro, M. J. Bearpark, J. J. Heyd, E. N. Brothers, K. N. Kudin, V. N. Staroverov, T. A. Keith, R. Kobayashi, J. Normand, K. Raghavachari, A. P. Rendell, J. C. Burant, S. S. Iyengar, J. Tomasi, M. Cossi, J. M. Millam, M. Klene, C. Adamo, R. Cammi, J. W. Ochterski, R. L. Martin, K. Morokuma, O. Farkas, J. B. Foresman, and D. J. Fox, Gaussian 16, Revision B.01, Gaussian, Inc., Wallingford CT, 2016.

[20] D. Geuenich, K. Hess, F. Köhler, R. Herges, *Chem. Rev.* **2005**, *105*, 3758–3772.

[21] P. von R. Schleyer, C. Maerker, A. Dransfeld, H. Jiao, N. J. R. van Eikema Hommes, *J. Am. Chem. Soc.* **1996**, *118*, 6317–6318.

[22] Z. Chen, C. S. Wannere, C. Corminboeuf, R. Puchta, P. von R. Schleyer, *Chem. Rev.* **2005**, *105*, 3842–3888.

[23] H. Fallah-Bagher-Shaidaei, C. S. Wannere, C. Corminboeuf, R. Puchta, P. v. R. Schleyer, *Org. Lett.* **2006**, *8*, 863–866.
